# Supplementary material for: Importance of vitamin D in acute and critically ill children with subgroup analyses of sepsis and respiratory tract infections: a systematic review and meta-analysis
Source: BMJ Open. 2019 May 22;9(5):e027666. doi: 10.1136/bmjopen-2018-027666 (PMC6538078; doi:10.1136/bmjopen-2018-027666)

Supplementary Material

Table of Contents

Additional Table 1 PRISMA Checklist 2009 3
Additional Table 2A Search terms used in OVID 5
Additional Table 2B Search terms used in PubMed 6
Additional Table 3A Newcastle Ottawa study quality scoring system (cohort studies) 7
Additional Table 3B Newcastle Ottawa study quality scoring system (case-control studies) 8
Additional Table 3C Newcastle Ottawa study quality scoring system (cross-sectional studies) 9
Additional Table 4A Circulating 25(OH)D threshold levels used in the selected studies 10
Additional Table 4B Circulating 25(OH)D threshold levels used in the selected studies for prevalence in sepsis 13
Additional Table 5 Studies with thresholds other than < 50 nmol/L 14
Additional Table 6 Excluded studies 15
Additional Table 7 Characteristics of the 52 included studies 16
Additional Table 8 Objectives and outcomes of included studies 22
Additional Table 9 Assay used in each study to measure Vitamin D levels 26
Additional Table 10 Funding and ethical approval of included studies 27
Additional Table 11 Age groups of children in each study 30
Additional Table 12 Prevalence of vitamin D deficiency in each study of acute and critically ill children (sorted from highest to lowest) 31
Additional Table 13 Characteristics of studies used in the meta-analysis of prevalence 33
Additional Table 14 Sensitivity analyses for prevalence of vitamin D deficiency in acute and critically ill children 35
Additional Table 15 Multivariate meta-regression model for prevalence 36
Additional Table 16 Characteristics of studies included in the meta-analysis for prevalence in individuals with sepsis 37
Additional Table 17 Sensitivity analyses for prevalence of vitamin D deficiency in acute and critically ill children with sepsis 38
Additional Table 18 Sensitivity analyses for mortality. Pooled odds ratios for risk of mortality in deficient versus not deficient children 39
Additional Figures 40

Additional Figure 1 Funnel plot of studies of prevalence of vitamin D deficiency in acute and critically ill children. 40

Additional Figure 2. Pooled prevalence estimate for vitamin D deficiency in acute and critically ill children (by country group). 41

Additional Figure 3. Pooled prevalence estimates for vitamin D deficiency in acute and critically ill children (neonates versus all other age groups). 42

Additional Figure 4 Bubble plots of univariate meta-regressions. 43

Additional Figure 5 Funnel plot for prevalence of vitamin D deficiency in acute and critically ill children with sepsis. 44

Additional Figure 6 Pooled prevalence estimate for vitamin D deficiency in acute and critically ill children with sepsis (subgroup analysis by study design). 45

Additional Figure 7 Funnel plot of risk of mortality in vitamin D deficient versus vitamin D non-deficient acute and critically ill children. 46

Additional Figure 8 Pooled odds ratio (OR) and 95% CI for risk of mortality in vitamin D deficient versus vitamin D non-deficient acute and critically ill children (fixed effects model). 47

Additional Table 1 PRISMA Checklist 2009

Section/topic	#	Checklist item	Reported on page #
**TITLE**			
Title	1	Identify the report as a systematic review, meta-analysis, or both.	1
**ABSTRACT**			
Structured summary	2	Provide a structured summary including, as applicable: background; objectives; data sources; study eligibility criteria, participants, and interventions; study appraisal and synthesis methods; results; limitations; conclusions and implications of key findings; systematic review registration number.	2-3
**INTRODUCTION**			
Rationale	3	Describe the rationale for the review in the context of what is already known.	4-5
Objectives	4	Provide an explicit statement of questions being addressed with reference to participants, interventions, comparisons, outcomes, and study design (PICOS).	6-8
**METHODS**			
Protocol and registration	5	Indicate if a review protocol exists, if and where it can be accessed (e.g., Web address), and, if available, provide registration information including registration number.	9
Eligibility criteria	6	Specify study characteristics (e.g., PICOS, length of follow-up) and report characteristics (e.g., years considered, language, publication status) used as criteria for eligibility, giving rationale.	5-7
Information sources	7	Describe all information sources (e.g., databases with dates of coverage, contact with study authors to identify additional studies) in the search and date last searched.	6-7
Search	8	Present full electronic search strategy for at least one database, including any limits used, such that it could be repeated.	6 and supplementary material
Study selection	9	State the process for selecting studies (i.e., screening, eligibility, included in systematic review, and, if applicable, included in the meta-analysis).	6-7
Data collection process	10	Describe method of data extraction from reports (e.g., piloted forms, independently, in duplicate) and any processes for obtaining and confirming data from investigators.	7
Data items	11	List and define all variables for which data were sought (e.g., PICOS, funding sources) and any assumptions and simplifications made.	7-8
Risk of bias in individual studies	12	Describe methods used for assessing risk of bias of individual studies (including specification of whether this was done at the study or outcome level), and how this information is to be used in any data synthesis.	8
Summary measures	13	State the principal summary measures (e.g., risk ratio, difference in means).	8
Synthesis of results	14	Describe the methods of handling data and combining results of studies, if done, including measures of consistency (e.g., I^2) for each meta-analysis.	8-10

Additional Table 1 PRISMA Checklist 2009

Section/topic	#	Checklist item	Reported on page #
Risk of bias across studies	15	Specify any assessment of risk of bias that may affect the cumulative evidence (e.g., publication bias, selective reporting within studies).	8
Additional analyses	16	Describe methods of additional analyses (e.g., sensitivity or subgroup analyses, meta-regression), if done, indicating which were pre-specified.	9-10 and 15
**RESULTS**			
Study selection	17	Give numbers of studies screened, assessed for eligibility, and included in the review, with reasons for exclusions at each stage, ideally with a flow diagram.	10
Study characteristics	18	For each study, present characteristics for which data were extracted (e.g., study size, PICOS, follow-up period) and provide the citations.	14-19 (supplementary material)
Risk of bias within studies	19	Present data on risk of bias of each study and, if available, any outcome level assessment (see item 12).	5-7 (supplementary material)
Results of individual studies	20	For all outcomes considered (benefits or harms), present, for each study: (a) simple summary data for each intervention group (b) effect estimates and confidence intervals, ideally with a forest plot.	13,15,18 and supplementary material
Synthesis of results	21	Present results of each meta-analysis done, including confidence intervals and measures of consistency.	13 and supplementary material pages 32-36
Risk of bias across studies	22	Present results of any assessment of risk of bias across studies (see Item 15).	5-7 (supplementary material)
Additional analysis	23	Give results of additional analyses, if done (e.g., sensitivity or subgroup analyses, meta-regression [see Item 16]).	supplementary material pages 32-36
**DISCUSSION**			
Summary of evidence	24	Summarize the main findings including the strength of evidence for each main outcome; consider their relevance to key groups (e.g., healthcare providers, users, and policy makers).	19-21
Limitations	25	Discuss limitations at study and outcome level (e.g., risk of bias), and at review-level (e.g., incomplete retrieval of identified research, reporting bias).	20-22
Conclusions	26	Provide a general interpretation of the results in the context of other evidence, and implications for future research.	22
**FUNDING**			
Funding	27	Describe sources of funding for the systematic review and other support (e.g., supply of data); role of funders for the systematic review.	22

From: Moher D, Liberati A, Tetzlaff J, Altman DG, The PRISMA Group (2009). Preferred Reporting Items for Systematic Reviews and Meta-Analyses: The PRISMA Statement. PLoS Med 6(6): e1000097. doi:10.1371/journal.pmed1000097 For more information, visit: www.prisma-statement.org.

Additional Table 2A Search terms used in OVID	
1.	Vitamin D
2.	Sepsis or septic shock
3.	Vitamin D or cholecalciferol* or ergocalciferol or 25-hydroxyvitamin D or calcidiol or calcitriol
4.	Intensive care unit* or critical care
5.	Multiple organ dysfunction syndrome or multiple organ failure
6.	(1 and 2) or 5
7.	((1 or 3) and 2 or 4
8.	(1 or 3) and 2
9.	(1 or 3) and 4
10.	(1 or 3) and 5
11.	Sepsis
12.	multi* organ dysfunction syndrome or multiple organ failure
13.	multi* organ dysfunction syndrome or multi* organ failure
14.	(2 or 8) and 3
15.	critical* ill or acute condition* or intensive care unit
16.	child* or pediatric*
17.	vitamin D or cholecalciferol or ergocalciferol or 25-hydroxyvitamin D or calcidiol or calcitriol
18.	15 and 16 and 17
19.	Vitamin D blood levels or 25-hydroxyvitamin
20.	5 and 3 and 15 and 16
21.	2 and 15 and 16 and 17
22.	16 and 2
23.	16 and 2 and 3
24.	Pediatric*
25.	Pediatric* and 5 and 2
26.	24 and 3 and 15

Additional Table 2B Search terms used in PubMed	
**Term searched**	
1.	Vitamin D.mp.
2.	(sepsis or septic shock).mp.
3.	(vitamin D or cholecalciferol* or ergocalciferol or 25-hydroxyvitamin D or calcidiol or calcitriol).mp.
4.	(intensive care unit* or critical care).mp.
5.	(multi* organ dysfunction syndrome or multiple organ failure).mp.
6.	(critical* ill or acute condition* or intensive care unit).mp.
7.	(toddler or infant or child* or neonate* or baby or teenager or pediatric* or paediatric*).mp.
8.	3 and 6 and 2 and 7

Additional Table 3A Newcastle Ottawa study quality scoring system (cohort studies)

Study	Selection				Comparability	Outcome			Number of stars (out of 9 total)
	Representativeness of the exposed cohort	Selection of the non-exposed cohort	Ascertainment of exposure	Demonstration that outcome of interest was not present at the start of the study	Comparability of cohorts on the basis of the design or analysis (maximum 2 stars)	Assessment of outcome	Was follow-up long enough for outcome to occur (≥ 28 days after admission to the ICU)	Adequacy of follow up of cohorts	
Ebenezer 2016	1	1	1	1	0	1	0	1	6
Sankar 2016	1	1	1	1	2	1	0	1	8
Rippel 2012	1	1	1	1	1	1	1	1	8
Madden 2012	1	1	1	1	2	1	0	1	8
McNally 2012	1	1	1	0	1	1	1	1	7
Dayal 2014	1	1	1	1	0	1	0	1	6
Ayulo 2014	1	1	1	1	2	1	0	0	7
Bustos 2016	1	1	1	0	2	1	0	1	7
Prasad 2015	1	1	1	0	2	1	0	1	7
Onwuneme 2015 (2)	1	1	1	1	2	1	0	1	8
Inamo 2011	1	1	1	0	1	1	0	1	6
Shah 2016	1	1	1	1	0	1	0	1	6
Lopez 2016	1	1	1	1	2	1	0	1	8
Garcia-Soler 2017	1	1	1	1	2	1	0	0	7
Sankar 2017	1	1	1	1	2	1	0	1	8
Asilioglu 2017	1	1	1	1	1	1	0	1	7
Halwany 2017	1	1	1	1	1	1	0	0	6
Hurwitz 2017	1	1	1	0	1	1	0	1	6
Banajeh 2009	1	1	1	0	1	1	1	1	7
Badawi 2017	1	1	1	1	1	1	1	1	8
Rey 2014	1	1	1	1	2	0	0	1	7
Ponnarmeni 2016	1	0	1	1	1	1	0	1	6
Onwuneme 2015 (1)	1	1	1	1	1	1	0	1	8
El-Gamasy 2017	1	0	1	1	2	1	0	1	7
Dhandai 2018	1	1	1	1	1	1	0	1	7
Hebbar 2014	1	0	1	1	1	1	0	1	6
Korwutthikulrangsri 2015	1	1	1	1	2	1	0	0	7
Elmoneim 2016	1	1	1	1	0	1	0	1	7
Vo 2018	1	1	1	1	2	1	0	1	8
Mathias 2017	1	1	1	1	0	1	0	1	6

The Newcastle-Ottawa Scale (NOS) for assessing the quality of non-randomised studies in meta-analyses. Available at: http://www.ohri.ca/programs/clinical_epidemiology/oxford.asp.

Additional Table 3B Newcastle Ottawa study quality scoring system (case-control studies)

Study	SELECTION				COMPARABILITY	EXPOSURE			Number of stars (out of 9 total)
	Is the case definition adequate?	Representativeness of the cases	Selection of controls	Definition of controls	Comparability of cases and controls on the basis of the design or analysis (maximum 2 stars)	Ascertainment of exposure	Same method of ascertainment for cases and controls (yes or no or 0 star if no mention)	Non-response rate	
Wayse 2004	1	1	0	0	1	1	1	1	6
Karatekin 2009	1	1	0	0	1	1	1	1	6
Roth 2009	1	1	0	1	1	1	1	0	7
Roth 2010	1	1	1	1	2	1	1	0	8
Cetinkaya 2015	1	1	0	0	0	1	1	1	6
Cizmeci 2015	1	1	0	0	2	1	1	1	7
Narang 2016	1	1	0	0	1	1	1	1	6
Dinlen 2016	1	1	0	0	1	1	1	1	6
Ahmed 2015	1	1	0	1	2	1	1	1	8
Cayir 2014	1	1	0	0	2	1	1	1	7
Say 2017	1	1	0	0	2	1	1	0	6
Basha 2014	1	1	0	1	2	1	1	0	7
Jia 2017	1	1	0	1	1	1	1	1	7
Jat 2016	1	1	0	1	2	1	1	1	8
Moreno-Solis 2015	1	1	0	1	1	1	0	1	6
Sakka 2014	1	1	0	1	2	1	1	1	8
Khakshour 2015	1	1	0	0	1	1	1	0	5
Li 2018	1	1	1	1	2	1	1	0	8

The Newcastle-Ottawa Scale (NOS) for assessing the quality of non-randomised studies in meta-analyses. Available at: http://www.ohri.ca/programs/clinical_epidemiology/oxford.asp.

Additional Table 3C Newcastle Ottawa study quality scoring system (cross-sectional studies)

	SELECTION (Maximum 5 stars)				COMPARABILITY (Maximum 2 stars)	OUTCOME (Maximum 3 stars)		
**Study**	**Representativeness of the sample**	**Sample size**	**Non-respondents**	**Ascertainment of the exposure (risk factor)**	**Subjects in different outcome groups are comparable, based on the study design or analysis. Confounding factors are controlled.**	**Assessment of the outcome (max=2 stars)**	**Statistical test (max=1 star)**	**Number of stars (out of 10 total)**
Yaghmaie 2017	1	0	0	0	1	1	1	4
Alvarez 2016	1	0	0	1	1	2	1	6
Alonso 2015	1	0	0	1	1	1	1	5
Binks 2014	1	0	0	1	1	2	1	6

The Newcastle-Ottawa Scale (NOS) for assessing the quality of non-randomised studies in meta-analyses. Available at: http://www.ohri.ca/programs/clinical_epidemiology/oxford.asp.

Additional Table 4A Circulating 25(OH)D threshold levels used in the selected studies

Study	Number of cases	25(OH)D categories (as given)	Number of cases in each category
Asilioglu 2017	250	Deficiency: <20 ng/mL	120
		Sufficiency: >=20 ng/mL	85
Halwany 2017	102	Deficiency: <20 ng/ml	38
		Insufficiency: 20-29.9 ng/ml	39
		"Normal" levels: 30 ng/ml	25
Hurwitz 2017	90	Deficiency: <20 ng/mL	11
Garcia-Soler 2017	340	Deficiency: <20 ng/mL	149
		Insufficiency: 20-30 ng/mL	128
		25(OH)D levels: 30-40 ng/mL	41
		Optimal levels >40 ng/mL	22
Badawi 2017	88	Deficiency: < 50nmol/l	39
		Severe deficiency: <30 nmol/l	30
Jia 2017	110	Severe deficiency: <10 ng/ml	36
		Deficiency: 10-20 ng/ml	56
		Insufficiency: 21-30 ng/ml	17
		Sufficiency: >30 ng/ml	1
Yaghmaie 2017	82	Deficiency: <30 ng/ml	53
		Sufficiency: >=30 ng/ml	29
Say 2017	100	Severe deficiency (group 1) <5 ng/mL.	63
		Insufficiency (group2): 5 to 15 ng/mL	24
		Sufficiency (group 3) ≥15 ng/mL	13
El-Gamasy 2017	80	Serious deficiency: < 30 nmol/L	26
		Insufficiency: 30-75 nmol/L	27
		Adequate levels >75 nmol/L	27
Sankar 2017	43	Severe deficiency: serum 25 (OH) D <10 ng/mL	31
Shah 2016	154	25(OH)D < 20 ng/mL	128
		25(OH)D ≥20 ng/mL	26
Ponnarmeni 2016	124	Deficiency: < 50nmol/L	63
		Insufficiency: 50-75 nmol/L	31
		<75, insufficient and deficient	94
		Sufficiency: >75 nmol/L	30
Sankar 2016	101	Deficiency: ≤ 20 ng/mL	75
		Severe deficiency: <15 ng/mL	62
		'No deficiency': > 20 ng/mL	26
Bustos 2016	90	Deficiency: <20 ng/ml	39
		Normal levels: >20 ng/ml	51
Ebenezer 2016	52	Deficiency: < 20ng/mL	21
		Insufficiency: 20–30 ng/ml	12
		'Normal levels: ≥ 20 ng/L	31
Elmoneim 2016	21	Deficiency: < 20 ng/mL	17
		Insufficiency: 20-30 ng/mL	4
		Normal levels: > 30 ng/mL	9
Jat 2016	50	Deficiency: <20 ng/ml	42
		Insufficiency: 20-30 ng/ml	2
		Sufficiency: >=30 ng/ml	1
Narang 2016	50	"Severe deficiency": <20ng/ml	28
Dinlen 2016	30	Deficiency: <=15 ng/mL	26
		Severe deficiency: <=5 ng/mL	3
Lopez 2016	347	25-OHD levels: <10 ng/mL	19
		25-OHD levels: 10-20 ng/mL	65
		25-OHD levels: 20-30 ng/mL	134
Alvarez 2016	50	25(OH) D levels: <20 ng/mL	29
		25(OH) D levels: 20-30 ng/mL	37
Onwuneme 2015 (1)	120	25(OH)D levels: <50nmol/L	71
		25(OH)D levels: ≥50nmol/L	49
Onwuneme 2015 (2)	94	25(OH)D levels: <20 ng/mL	86
		25(OH)D levels: < 30 nmol/L	60
		25(OH)D levels: >=30 nmol/L	34
Prasad 2015	80	Deficiency: < 20 ng/ml	67
		Sufficiency: ≥ 20 ng/mL	13
Moreno-Solis 2015	48	Deficiency: <20 ng/ml	9
		Insufficiency: 21–29 ng/ml	16
		Sufficiency: ≥30 ng/ml	23

Alonso 2015	288	Deficiency: <20 ng/ml	45
		<10 ng/ml	6
Korwutthikulrangsri 2015	32	Deficiency: < 20 ng/ml	25
		Insufficiency: 20-29.9 ng/ml	7
		Sufficiency: ≥ 30 ng/mL	0
Khakshour 2015	37	Deficiency: <20 ng/mL	9
Cizmeci 2015	40	Deficiency: ≤20 ng/ml	28
		Insufficiency: 21–29 ng/ml	7
		Normal levels: ≥30 ng/ml	5
Cetinkaya 2015	50	Severe deficiency: <10 ng ml^{-1}	42
		Insufficiency: 11 to 32 ng ml^{-1}	8
Ayulo 2014	216	Deficient: < 15 ng/ml	61
		Insufficient: 15-29 ng/mL	102
		Sufficient: ≥ 30 ng/mL	53
Dayal 2014	92	Deficiency: < 50 nmol/L	23
		Insufficiency: 50–75 nmol/L	41
		Sufficiency: > 75 nmol/L	28
		25(OH) D levels: < 75 nmol/L	64
		‘Non-deficiency’: > 50 nmol/L	69
Hebbar 2014	61	Deficiency: ≤ 10 ng/mL	10
		Insufficiency: 10 to 20 ng/mL	27
		Sufficiency: ≥ 20 ng/mL	24
Rey 2014	156	Deficiency: < 20 ng/mL	46
		25(OH)D levels: ≥ 20 ng/mL	110
Ahmed 2014	50	Deficiency: <50 nmol/L	15
Basha 2014	81	Deficiency: <50 nmol/L	53
		Insufficiency: <75 nmol/L	14
		Normal level: >75 nmol/L	14
Sakka 2014	96	Severe deficiency: 0–5 ng/mL	29
		Deficiency: 5–15 ng/mL	49
		Insufficiency: 15– 20 ng/mL	11
		Sufficiency: 20–100 ng/mL	7
Cayir 2014	88	Normal levels: 20 ng/mL	38
		Insufficiency: 15-20ng/mL	18
		Deficiency: <15ng/mL	32
Binks 2014	74	25(OH) D levels: < 50 nmol/L	11
Madden 2012	511	25(OH) D levels: < 10 ng/mL	36
		25(OH) D levels: 10-19.9 ng/mL	169
		Deficiency: < 20 ng/ml	205
		Insufficiency: <30 ng/mL	364
Rippel 2012	316	Deficiency: < 50 nmol/L	109
		‘Normal levels’: ≥50 nmol/L	207
Madden 2012	511	25(OH)D levels: < 10 ng/mL	36
		25(OH)D levels: 10-19.9 ng/mL	169
		Deficiency: < 20 ng/mL	205
		Insufficiency: <30 ng/mL	364
McNally 2012	326	Deficiency: < 50 nmol/L	225
		25(OH)D levels: 50 to 75 nmol/L	75
		‘Not deficient’: > 50 nmol/L	101
Inamo 2011	28	25(OH)D levels: < 10 ng/mL	4
		25(OH)D levels: < 15 ng/mL	8
		25(OH)D levels: < 25 ng/mL	12
		25(OH)D levels: < 40 ng/mL	28
Roth 2010	25	25(OH)D levels: < 40 nmol/L	21
Banajeh 2009	79	Deficiency: <30 nmol/L	29
Karatekin 2009	25	Serum 25(OH)D <10 (deficiency)	19
		Serum 25(OH)D 11 to 20 (deficiency)	4
		Serum 25(OH)D 21 to 32 (insufficiency)	1
		Serum 25(OH)D 32 to 100 (sufficiency)	1
Roth 2009	64	< 40 nmol/L	3
Wayse 2004	80	Plasma 25(OH)D3 > 22.5 nmol/L	26
		Plasma 25(OH)D3 > 50 nmol/L	4
Mathias 2017	41	25(OH)D < 20 ng/ml	28

		25(OH)D < 30 ng/ml	36
Dhandai 2018	60	Deficiency: < 20 ng/ml	38
		Insufficiency: <29 ng/ml	17
		Optimum: 30-50 ng/ml	5
Vo 2018	1016	25(OH)D < 20 ng/ml	298
		20-29.9 ng/ml	352
		>=30 ng/ml	366
Li 2018	797	Severe deficiency: < 10 ng/mL	159
		Deficiency: 10–20 ng/mL	286
		Insufficiency: 20– 30 ng/mL	223
		Sufficiency: > 30 ng/mL	127

25(OH)D in nmol/L = 25(OH)D in ng/mL multiplied by 2.496

Additional Table 4B Circulating 25(OH)D threshold levels used in the selected studies for prevalence in sepsis

Study	Number of cases	25(OH)D categories (as provided by each study)	Number of cases
Asilioglu 2017	30 sepsis	Deficiency: <20 ng/mL	20
		Sufficiency: >=20 ng/mL	10
Say 2017	100 neonatal sepsis	Severe deficiency (group 1) <5 ng/mL.	63
		Insufficiency (group2): 5 to 15 ng/mL	24
		Sufficiency (group 3) ≥15 ng/mL	13
El-Gamasy 2017	46 sepsis	Serious deficiency: < 30 nmol/L	20
		Insufficiency: 30-75 nmol/L	18
		Adequate levels >75 nmol/L	8
Sankar 2017	43 septic shock	Severe deficiency: serum 25 (OH) D <10 ng/mL	31
Shah 2016	100 sepsis	25(OH)D <20 ng/mL	84
		25(OH)D >=20 ng/mL	26
Ponnarmeni 2016	124 sepsis	Deficiency: <50nmol/L	63
		Insufficiency: 50-75 nmol/L	31
		<75, insufficient + deficient	94
		Sufficiency: >75 nmol/L	30
Bustos 2016	10 sepsis intraabdominal	Deficiency: < 20 ng/ml	7
		Normal levels: > 20 ng/mL	3
Ebenezer 2016	16 shock	25(OH)D <20 ng/mL	8
		25(OH)D >=20 ng/mL	8
Onwuneme 2015 (1)	35 culture positive sepsis	25(OH)D <50 ng/mL	32
		25(OH)D >=50 ng/mL	3
Onwuneme 2015 (2)	46 culture positive sepsis and late-onset sepsis	Deficiency: < 30 nmol/L	32
		Sufficiency: ≥ 30 nmol/L	14
Prasad 2015	11 positive blood culture	25(OH)D <20 ng/mL	9
		25(OH)D >=20 ng/mL	2
Korwutthikulrangsri 2015	17 shock and septicaemia	25(OH)D <20 ng/mL	14
		25(OH)D >=20 ng/mL	3
Cizmez 2015	40 suspected sepsis	Deficiency: ≤20 ng/ml	28
		Insufficiency: 21–29 ng/ml	7
		Normal levels: ≥30 ng/ml	5
Dayal 2014	9 nosocomial sepsis	25(OH)D <20 ng/mL	4
		25(OH)D >=20 ng/mL	5
Hebbar 2014	30 shock and/or Sepsis	25(OH)D <20 ng/mL	17
		25(OH)D >=20 ng/mL	13
McNally2012	48 septic	25(OH) D levels: <50 nmol/L	33
		25(OH) D levels: ≥50 nmol/L	15
Mathias 2017	41 sepsis, severe sepsis or septic shock	25(OH)D < 20 ng/ml	28
		25(OH)D < 30 ng/ml	36
Dhandai 2018	60 sepsis	Deficiency: < 20 ng/ml	38
		Insufficiency: <29 ng/ml	17
		Optimum: 30-50 ng/ml	5

Additional Table 5 Studies with thresholds other than < 50 nmol/L

Study	Threshold used by study
Roth 2009	< 40 nmol/L
Roth 2010	< 40 nmol/L
Say 2017	<=15 ng/mL (37.4 nmol/L)
Inamo 2011	<=15 ng/mL (37.4 nmol/L)
Ayulo 2014	<=15 ng/mL (37.4 nmol/L)
Dinlen 2016	<=15 ng/mL (37.4 nmol/L)
Onwuneme 2015 (2)	< 30 nmol/L
Yaghmaie 2017	< 30 ng/mL (74.88 nmol/L)
El-Gamasy 2017	< 30 nmol/L
Banajeh 2009	< 30 nmol/L
Sankar 2017	<= 10 ng/mL (24.9 nmol/L)
Cetinkaya 2015	<= 10 ng/mL (24.9 nmol/L)

25(OH) D values nmol/L = ng/mL * 2.496

Additional Table 6 Excluded studies

Study	Design	Sample size	Characteristics of pediatric population	Country	Reasons for exclusion of paper
Seliem 2016	Hospital-based case-control	Cases, n= 30<br>Controls, n= 30	Cases: group 1, full-term neonates with EOS and their mothers.<br>Controls, group 2: 30 full-term healthy neonates and their mothers with no clinical or laboratory evidence of sepsis.	Egypt	Requested data without reply or data not available
Gamal 2017	Case-control	Cases, n= 50<br>Control, n= 30	Cases: neonates with early onset neonatal sepsis that occurred at <72 hours<br>Controls: Age and sex healthy neonates with no prenatal risk factor for early neonatal sepsis enrolled in the study as a control group.	Egypt	Requested data without reply or data not available
Aydemir 2014	Hospital-based case control	Cases, n=40<br>Controls, n= 20	Cases: children with sepsis between 1 and 16 years old<br>Controls: children without sepsis	Turkey	Requested data without reply or data not available
Garg 2016	Hospital-based case-control	Cases, n= 40<br>Controls, n= 40	Cases: children from 6 months to 5 years of age admitted or attending OPD in department of Pediatrics.<br>Controls: children receiving care at the Hospital's ambulatory, emergency or in-patient units and presenting with non-respiratory complaints also not having any clinical indication of vitamin D deficiency.	India	Requested data without reply or data not available

Additional Table 7 Characteristics of the 52 included studies

Study	Design	Number of cases (controls, where appropriate)	Characteristics of population	Country and setting	Vitamin D thresholds as defined by the study	Quality score (NOS)
Asilioglu 2017	Historical cohort (single centre)	250	Cases: aged 1 month to ≤18 years	Turkey, PICU	Deficiency: <20 ng/mL	7
Halwany 2017	Cohort (single centre)	102	Cases: children aged >1 month to ≤ 5 years	Egypt, PICU	Deficiency: <20 ng/ml<br>Insufficiency: 20-29.9 ng/ml<br>"Normal" levels: 30 ng/ml	6
Hurwitz 2017	Prospective cohort (single centre)	90	Cases: aged <5 years hospitalized with LRTI and RSV and/or hMPV	USA, PICU	Deficiency: <20 ng/mL	6
Garcia-Soler 2017	Cohort (single centre)	340	Cases: aged 6 months to 17 years<br>Critically ill with various conditions	Spain, PICU	Deficient: <20 ng/mL<br>Insufficiency: 20-30 ng/mL<br>25(OH)D levels: 30-40 ng/mL<br>Optimal levels >40 ng/mL	7
Badawi 2017	Cohort (single centre)	88	Cases: 1 month to 12 years	Egypt, PICU	Deficiency: < 50nmol/l,<br>Severe deficiency: <30 nmol/l	8
Jia 2017	Hospital-based case-control (single centre)	110 (110)	Cases: infants (< 1 year of age) with ALRTI<br>Controls: Healthy from similar areas attending the hospital for vaccination during study period	China, PICU	Severe deficiency: <10 ng/ml<br>Deficiency: 10-20 ng/ml<br>Insufficiency: 21-30 ng/ml<br>Sufficiency: >30 ng/ml	7
Yaghmaie 2017	Cross sectional (single centre)	82	Cases: hospitalized in PICU	Iran, PICU	Deficiency: <30 ng/ml	4
Say 2017	Case-control (single centre)	100 (13)	Cases: premature infants less than 37 weeks diagnosed with early or late -onset neonatal sepsis<br>Controls: From same population of neonates with sepsis but not vitamin D deficient	Turkey, NICU	Severe deficiency (group 1) <5 ng/mL.<br>Insufficiency (group2): 5 to 15 ng/mL<br>Sufficiency (group 3) ≥15 ng/mL	6

El-Gamasy 2017	Cohort (single centre)	80 (20)	Cases: 3 months to 12 years hospitalized with acute kidney injury<br>Controls: completely healthy subjects	Egypt, PICU	Seriously deficient: < 30 nmol/L<br>Insufficient: 30-75 nmol/L<br>Adequate >75 nmol/L	7
Sankar 2017	Cohort (single centre)	43	Cases: <=17 years of age	India, PICU	Severe vitamin D deficiency: serum 25 (OH) D <10 ng/mL	8
Shah 2016	Cohort (single centre)	154	Cases: aged between 1 month and 15 years	India, PICU	Deficiency: <20 μg/mL	6
Ponnarmeni 2016	Cohort (single centre)	124 (338)	Cases: aged 1–12 years admitted with a diagnosis of sepsis<br>Controls: from previous prospective study, apparently healthy children of upper socioeconomic status who attended the out-patient department for immunization or with minor ailments	India, PICU	Deficiency: < 50 nmol/L<br>Insufficiency: 50-75 nmol/L<br>Sufficiency: > 75 nmol/L	6
Sankar 2016	Cohort (single centre)	101	Cases: aged 1 month to 17 years	India, PICU	Deficiency: ≤ 20 ng/ml<br>Severe deficiency: <15 ng/mL	8
Bustos 2016	Cohort (single centre)	90	Cases: critically ill, greater than 37 weeks and less than 15 years of age	Chile, PICU	Deficiency: < 20 ng/ml<br>Normal levels: > 20 ng/mL	7
Ebenezer 2016	Cohort (single centre)	52	Cases: <18 years of age; medical and surgical diagnoses	India, PICU	Deficiency: < 20 ng/ml<br>Insufficiency: 20–30 ng/ml<br>Normal levels: ≥ 20 ng/L	6
Elmoneim 2016	Cohort (single centre)	30	Cases: aged less than 14 years<br>Controls: with "normal" vitamin D levels	Saudi Arabia, PICU	Deficiency: < 20ng/ml<br>Insufficiency: 20-30 ng/mL<br>Normal: > 30 ng/mL	7
Jat 2016	Hospital-based case-control (single centre)	50 (50)	Cases: 1 month to 12 years of age admitted with pneumonia<br>Controls: admitted for reasons other than respiratory symptoms, required blood sampling	India, PICU	Deficiency: < 20 ng/ml<br>Insufficiency: 20-30 ng/ml Sufficiency: >= 30 ng/ml	8
Narang 2016	Hospital-based case-control (single centre)	50 (50)	Cases: 2 months to 5 years of age admitted as in-patients or seen in the outpatient department with ALRI<br>Controls: healthy, same age group, attending outpatients' service for immunization or admitted for minor conditions other than ALRI	India, PICU	Severe deficiency: < 20ng/ml	6

Dinlen 2016	Hospital-based case-control (single centre)	30 (30)	Cases: term neonates with ALRI<br>Controls: healthy neonates, same age as the study group.	Turkey, NICU	Deficient: <= 15 ng/mL<br>Severe deficiency: <= 5 ng/mL	6
Lopez 2016	Prospective cohort (multi centre)	347	Cases: 0 to >48 months admitted to hospital with ALRI prospectively recruited through the GENDRES (GENetic,vitamin D and RESpiratory infections research network)	Spain, PICU	25-OHD levels: <10 ng/mL<br>25-OHD levels: 10-20 ng/mL<br>25-OHD levels: 20-30 ng/mL	8
Alvarez 2016	Cross-sectional (single centre)	50	Cases: patients aged 0 to 18 years	USA, PICU	25(OH)D < 20 ng/mL<br>25(OH)D < 30 ng/mL	6
Onwuneme 2015 (1)	Cohort (single centre)	120 (30)	Cases: with suspected sepsis (<12 years old)<br>Controls: paediatric controls admitted for elective day case surgery during the same study period and were not suspected of having sepsis	Ireland , PICU	25(OH) D levels: <50 nmol/L<br>25(OH) D levels: ≥50 nmol/L	8
Onwuneme 2015 (2)	Cohort (single centre)	94	Cases: preterm infants <32 weeks gestation	Ireland, NICU	Deficiency: < 30 nmol/L<br>Sufficiency: ≥ 30 nmol/L	8
Prasad 2015	Cohort (single centre)	80	Cases: 2 months to 12 years old	India, PICU	Deficiency: < 20 ng/ml<br>Sufficient: ≥ 20 ng/mL	7
Moreno-Solis 2015	Hospital-based case-control (single centre)	48 (30)	Cases: aged 1–11 months with acute bronchiolitis<br>Controls: healthy, <12 months, admitted to the outpatient clinic without respiratory symptoms or history of hospitalization for bronchiolitis or wheezing	Spain, PICU	Deficiency: < 20 ng/ml<br>Insufficiency: 21–29 ng/ml Sufficiency: ≥ 30 ng/ml	6
Alonso 2015	Cross sectional (single centre)	288	Cases: aged 1 month to 13 years	Spain, PICU	Deficient: < 20 ng/ml and < 10 ng/ml	5
Korwutthikulrangsri 2015	Cohort (single centre)	32 (36)	Cases: requiring PICU admission<br>Controls: Healthy, enrolled during the same period of time and served as the control group (age in months)	Thailand, PICU	Deficiency: < 20 ng/mLl<br>Insufficiency: 20-29.9 ng/ml<br>Sufficiency: ≥ 30 ng/mL	7
Khakshour 2015	Case-control	37 (53)	Cases: below 5 years of age and suffering from respiratory infections<br>Controls: those who were not suffering from respiratory infections	Iran, PICU	Deficiency: < 20 ng/mL	5

Cizmeci 2015	Case-control (single centre)	40 (43)	Cases: infants with suspected early-onset neonatal sepsis.<br>Controls: For each newborn of group 1, one healthy infant selected as a control	Turkey, NICU	Deficiency: ≤ 20 ng/ml<br>Insufficiency: 21–29 ng/ml<br>Normal levels: ≥ 30 ng/ml	7
Cetinkaya 2015	Hospital-based case-control (single centre)	50 (50)	Cases: term infants with clinically suspected (probable) early infection (early-onset sepsis) within the first 3 postnatal days of life and were >37 weeks of gestational age<br>Controls: healthy infants with no signs of clinical/laboratory infection	Turkey, NICU	Severe deficiency: <10 $ng\ ml^{-1}$<br>Insufficiency: 11 to 32 $ng\ ml^{-1}$<br>Adequacy: 32 to 100 $ng\ ml^{-1}$	6
Ayulo 2014	Cohort (single centre)	216	Cases: between the ages of 1 and 21 years	Spain, PICU	Deficient: < 15 ng/ml<br>Insufficient: 15-29 ng/mL<br>Sufficient: ≥ 30 ng/mL	7
Dayal 2014	Cohort (single centre)	92	Cases: Children aged 3 months to 12 years	India, PICU	Deficiency: < 50 nmol/L<br>Insufficiency: 50–75 nmol/L<br>Sufficiency: > 75 nmol/L<br>25(OH) D levels: < 75 nmol/L	6
Hebbar 2014	Cohort (single centre)	61 (46)	Cases: children 0 to 18 years<br>Controls: patients recruited among children in the magnetic resonance imaging suite.	USA, PICU	Deficient: ≤ 10 ng/ml<br>Insufficient: 10 to 20 ng/ml<br>Sufficient: ≥ 20 ng/mL	6
Rey 2014	Cohort (single centre)	156 (289)	Cases: heterogeneous group of critically ill children aged <16 years<br>Control group for comparison: population of healthy children	Spain, PICU	Deficient: < 20 ng/ml	7
Ahmed 2014	Hospital-based case-control (single centre)	50 (50)	Cases: aged 2–60 months hospitalized with ALRI<br>Controls: age-matched with cases within 1 or 2 months, attending well-child clinics or general clinics without evidence of respiratory infection or admitted to the hospital for elective surgery	Africa, PICU	Deficiency: <50 nmol/L	8
Basha 2014	Hospital-based case-control (single centre)	81 (89)	Cases: under 5 years old with severe pneumonia selected from the inpatient departments and emergency units of the hospital<br>Controls: healthy, selected from the outpatient surgical clinics of the hospital during their visit for umbilical or inguinal hernia repair and not suffering	Egypt, PICU	Deficiency: < 50 nmol/L<br>Insufficiency: < 75 nmol/L<br>Sufficiency: > 75 nmol/L	7

			from upper or lower respiratory infections			
Sakka 2014	Population based case-control (single centre)	96 (96)	Cases: <2 years old with ALRI, 48 diagnosed with pneumonia and 48 with bronchiolitis<br>Controls: age and sex matched with no respiratory symptoms or signs from the Health office	Egypt, PICU	Severe deficiency: 0–5 ng/mL Deficiency: 5–15 ng/mL Insufficiency: 15– 20 ng/mL Sufficiency: 20–100 ng/mL	8
Cayir 2014	Hospital-based case-control (single centre)	88 (81)	Cases: 1 to 13 years diagnosed with acute otitis media<br>Controls: Healthy same age range	Turkey, PICU	Normal levels: 20 ng/mL<br>Insufficiency: 15-20ng/mL<br>Deficiency: <15ng/mL	7
Binks 2014	Cross-sectional	74	Cases: aged <3 years admitted with acute lower respiratory infections (ALRIs) or other conditions	Australia, PICU	25(OH) D levels: < 50 nmol/L	6
Madden 2012	Cohort (single centre)	511	Cases: less than 21 years old	USA, PICU	25(OH) D levels: < 10 ng/mL<br>25(OH) D levels: 10-19.9 ng/mL<br>Deficiency: < 20 ng/ml<br>Insufficiency: < 30 ng/mL	8
Rippel 2012	Cohort (single centre)	316	Cases: children aged 16.5 (3.1–75.2) months	Australia, PICU	Deficiency: < 50 nmol/L<br>“Normal” levels: ≥ 50 nmol/L	8
McNally 2012	Cohort (multi-centre)	326	Cases: Newborn to 17 years of age	Canada, PICUs	Deficiency: < 50 nmol/L<br>25(OH) D levels:<br>50 to 75 nmol/L	7
Inamo 2011	Cohort (single centre)	28	Cases: between 1 and 48 months hospitalized with ALRI, 26 diagnosed with bronchiolitis and two as having pneumonia	Japan, PICU	Deficiency: ≤ 15ng/ml<br>Severe deficiency:<br>≤ 5ng/mL	6
Roth 2010	Hospital-based case-control (single centre)	25 (25)	Cases: 1–18 months hospitalized with ALRI<br>Controls: selected by population-based sampling. aged 1–23 months, and matched to cases on age (±2 months) and sex	Bangladesh, PICU	Deficiency: < 40 nmol/L	8
Banajeh 2009	Prospective cohort (single centre)	79	Cases: 2–59 months with WHO-defined very severe community acquired (VSP) pneumonia	Iran, PICU	Deficiency: < 30 nmol/L	7
Karatekin 2009	Hospital-based case-	25 (15)	Cases: newborns with acute respiratory infections	Turkey, NICU	Deficiency: < 20 ng/ml	6

	control, (single centre)		Controls: healthy, age matched from outpatients' service where they went for immunization.			
Roth 2009	Hospital-based case-control (single centre)	64 (65)	Cases: aged 1-25 months admitted with ALRI<br>Controls: aged 1-25 months undergoing elective surgery, no history of hospitalization for ALRI	Canada, PICU	Deficiency: < 40 nmol/L	7
Wayse 2004	Hospital-based case-control, (single centre)	80 (70)	Cases: < 5 years with severe ALRI<br>Controls: healthy, attending outpatients service for immunization	India, PICU	Deficiency: Plasma 25(OH)D3 < 50 nmol/L	6
Mathias 2017	Prospective cohort (single centre)	41	Cases: less than 18 years admitted with diagnosis of sepsis, severe sepsis or septic shock.	USA, PICU	25(OH)D levels < 20 ng/ml<br>25(OH)D levels <30 ng/ml	6
Dhandai 2018	Cohort (multi-centre)	60 (60)	Cases: neonates admitted with late-onset sepsis (LOS)<br>Controls: neonates admitted during same period with clinically significant physiological hyperbilirubinaemia (without sepsis)	India, NICU	Deficiency: < 20 ng/ml<br>Insufficiency: <29 ng/ml<br>Optimum: 30-50 ng/ml	7
Vo 2018	Prospective cohort (multi-centre)	1016	Cases: Infants less than twelve months old hospitalized for bronchiolitis	USA, PICU	25(OH)D < 20 ng/ml<br>25(OH)D: 20-29.9 ng/ml<br>25(OH)D >= 30 ng/ml	8
Li 2018	Case-control (single-centre)	797 (785)	Cases: children with pneumonia or pneumonia-induced sepsis group	China, PICU	Severe deficiency: < 10 ng/mL<br>Deficiency: 10–20 ng/mL<br>Insufficiency: 20– 30 ng/mL<br>Sufficiency: > 30 ng/mL	8

LRTI = lower respiratory tract infection; PICU = paediatric intensive care unit; RSV = respiratory syncytial virus; ALRI = acute lower respiratory infection; WHO = World Health Organization; VSP = very severe community acquired pneumonia; NICU = neonatal intensive care unit.

Additional Table 8 Objectives and outcomes of included studies

Study	Objectives/aims of study	Main outcome(s) and conclusion(s)
Madden 2012	Prevalence of vitamin D deficiency in critically ill children and factors influencing admission 25-hydroxyvitamin D (25(OH)D) levels	Identified high prevalence of vitamin D deficiency and insufficiency in critically ill children. Inverse association severity of illness on admission and 25(OH) levels
Lopez 2016	Role of Vitamin D in Children hospitalized with Lower Tract Acute Respiratory Infections	25-hydroxyvitamin D levels of study population below normal range. Correlation of higher disease severity with lower levels of 25-hydroxyvitamin D.
Garcia-Soler 2017	Prevalence and risks factors of vitamin D deficiency, as well as its relationship with length of PICU stay morbidity and mortality in a PICU.	Vitamin D deficiency is frequent in paediatric critical patients. It also has an association with higher severity scores, season of year and parental educational attainment.<br>Vitamin D levels associated with various laboratory parameters of SIRS. Vitamin D deficiency associated with increased risk of morbidity and mortality. Inconclusive findings on its association with PICU length of stay were inconclusive
McNally 2012	Prevalence of vitamin D deficiency, risk factors and potential association with clinically relevant outcomes in critically ill children in Canada.	Most of critically ill children vitamin D deficient at PICU admission. Lower 25(OH) D levels associated with increased catecholamine requirements, fluid bolus administration, hypocalcemia, and longer PICU admission.
Rippel 2012	Prevalence of hypovitaminosis D and association with outcome in critically ill children requiring admitted in intensive care.	Hypovitaminosis D is frequent in critically ill children in PICU, especially in infants and children with heart disease. Hypovitaminosis D associated with hypocalcemia in non-cardiac population, and increased need for calcium replacement in the cardiac population.<br>No association between vitamin D status and survival or PICU length of stay. Strong association with early postoperative inotropic needs in the cardiac population.
Alonso 2015	Investigate relationship of serum 25-hydroxyvitamin D concentrations with serum parathyroid hormone (PTH) levels, body mass index (BMI), and environmental factors in a population of Caucasian children living at latitude 43°N.	Results doubt the assumption that a serum 25OH D threshold indicates vitamin D deficiency in children.
Asilioglu 2017	Measure occurrence of VDD in critically ill children. Assess determinants of vitamin D status and compare vitamin D deficient and sufficient cases in respect of severity of illness.	Hypovitaminosis D occurrence high in critically ill children and associated with higher vasopressor requirement. Not associated with other markers of illness severity including mortality.
Ayulo 2014	Prevalence of vitamin D deficiency among children in PICU	Vitamin D deficiency common. No significant correlation between disease severity and vitamin D levels levels of vitamin D. Mortality associated with vitamin D levels
Rey 2014	Identify prevalence of 25 hydroxivitamin D or 25(OH) vitamin D deficiency on pediatric intensive care unit (PICU) admission, and if associated with increased prediction of mortality risk scores.	Hypovitaminosis D incidence high in PICU patients. Hypovitaminosis D not associated with higher prediction of risk mortality scores.
Shah 2016	Determine prevalence of vitamin D deficiency in critically ill children its association with illness severity, parathyroid response and clinical outcomes.	High prevalence of vitamin D deficiency. Parathyroid gland response secondary vitamin D deficiency or hypocalcemia impaired in critically ill.
Ponnarmeni 2016	Vitamin D deficiency in critically ill children with sepsis admitted to PICU and its association with: mortality, length of stay, illness severity, requirement for ventilation and catecholamines	High prevalence of vitamin D deficiency<br>No significant association between vitamin D deficiency and other outcomes such as mortality
Onwuneme 2015 (1)	Assess vitamin D status, and its determinants, in chidren with suspected sepsis admitted to PICU. Also investigated association between vitamin D status and clinical outcomes.	Children admitted to the PICU with suspected sepsis lower 25OH D compared to controls. Inadequate 25 OH D levels associated with confirmed sepsis and poor outcomes.
Jia 2017	Association of vitamin D with ALRTI in Chinese infants	Lowered plasma level of 25-OH Vitamin D makes children susceptible to ALRTI.
Halwany 2017	Frequency of vitamin D deficiency in critically ill pediatric intensive care unit [PICU] patients and relation to state of serum 25(OH) D to disease severity.	High prevalence of vitamin D deficiency in critically ill children.<br>Negative correlation of Vitamin D level with PELOD score. Recommend screening of critically ill children for vitamin D deficiency to restore their serum levels.

Sankar 2016	Prevalence of vitamin D deficiency on admission and examine association with length of ICU stay	High prevalence of vitamin D deficiency. Vitamin D deficient children with longer ICU stay duration or mortality
Say 2017	Evaluate effects of low vitamin D levels in cord blood on neonatal sepsis in preterm infants.	No significant relationship between the cord blood vitamin D levels and the risk of neonatal sepsis in premature infants.
Sakka 2014	Determine the relation between vitamin D deficiency, anemia and the severity of ALRTIs in hospitalized children.	Vitamin D deficiency, low BMI, low hemoglobin level, rachitic signs were risk factors for the severity of ALRTIs.
Onwuneme2015 (2)	Investigate the association between serum 25-hydroxyvitamin D (25OHD) levels and outcomes in preterm infants (<32 weeks gestation).	High prevalence of low 25OHD. Association between vitamin D status and acute respiratory morbidity in preterm infants after birth. In none of the following outcomes was the difference statistically significant: surfactant use, inotrope requirement, RDS, pneumothorax, pulmonary hemorrhage, chronic lung disease, sepsis, retinopathy of prematurity, intraventricular hemorrhage, periventricular leucomalacia, patent ductus arteriosus, and mortality
Dayal 2014	Prevalence of vitamin D deficiency<br>Association of serum vitamin D levels with duration of stay in hospital, mortality and requirement of ventilation	Reduced serum vitamin D levels in children
Bustos 2016	Determine prevalence of vitamin D deficiency and its association with other clinically relevant outcomes in children admitted to Paediatric Intensive Care Unit	Vitamin D deficiency was prevalent in critically ill children and associated with adverse clinical outcomes.
Hurwitz 2017	Measure retinol binding protein and vitamin D in children aged <5 years hospitalized with lower respiratory tract infection and respiratory syncytial virus and/or human meta pneumovirus detections	Low vitamin levels in 50% of the children and associated with significantly elevated risk of the need for intensive care unit admission and invasive mechanical ventilation.
Cayir 2014	Investigate the relationship between Vitamin D deficiency and acute otitis media infection	Serum 25-hydroxy vitamin D levels significantly lower in children with acute otitis media compared to the controls. Vitamin D deficiency plays a role in otitis media infection.
Badawi 2017	Investigated if VDD is related to higher severity scores and organ dysfunction. Primary objective of study was to estimate the prevalence of VDD in a group of critically ill children, and secondary objectives was to correlate vitamin D status with pediatric logistic organ dysfunction (PELOD) and pediatric risk ofmortality III (PRISM III) scores.	VDD prevalence was reported in about half of the critically ill patients, and it was observed to be related to multiple organ dysfunctions and rapid clinical deterioration.
Yaghmaie 2017	Investigate relation of vitamin D deficiency with potential demographic and clinical factors.	Vitamin D deficiency among paediatric intensive care unit patients similar to western countries, also with similar age and BMI distribution. Significant relation observed between age and serum level of vitamin D.
Basha 2014	Aimed to evaluate vitamin D status as a risk factor for severe pneumonia in Egyptian hospitalized children under 5 years	Significant association between vitamin D deficiency and severe pneumonia in Egyptian children below 5 years
Prasad 2015	Prevalence of vitamin D deficiency in critically ill children and association with illness severity& other outcomes	High prevalence of vitamin D deficiency in critically ill children and association with PRISM III scores in a developing country.<br>Groups did not differ in terms of PICU stay, duration of hospital stay, culture positivity, biochemical parameters (serum calcium, serum phosphate), need of ventilation or steroids, presence of coagulopathy and mortality.
Wayse 2004	If vitamin D deficiency in Indian children under 5 years old of age is risk factor for severe acute lower respiratory infection (ALRI).	Subclinical vitamin D deficiency & nonexclusive breastfeeding in four first months of life found to be significant risk factors for severe ALRI in Indian children.
El-Gamasy 2017	Assess serum 25 (OH)D level in critically ill paediatric patients with AKI at PED of Tanta University Emergency Hospital (TUEH) within the first 24 hours of admission and evaluate its correlation with duration of hospital stay and mortality outcome.	Vitamin D deficiency associated with higher incidence of sepsis and mortality.

Banajeh 2009	Determine if rickets and VDD predict the outcomes in very severe pneumonia (VSP).	In WHO-defined VSP, nutritional rickets was strongly associated with a reduced successful treatment outcome, and VDD was a significant and independent predictor of reduced circulating PMNs and persistent hypoxemia.
Binks 2014	Aimed to determine prevalence of vitamin D insufficiency among children hospitalised with ALRI in the Northern Territory.	Vitamin D insufficiency was observed in about one-third of these hospitalised children. Children hospitalised with an ALRI less likely to have vitamin D insufficiency compared with children hospitalised for other conditions (predominantly gastroenteritis).
Roth 2009	Test the hypothesis that vitamin D status is associated with the risk of ALRI in Canadian children (1 month to 2 years old)	Among children aged 1 month to 2 years, vitamin D status not associated with ALRI requiring hospitalization
Hebbar 2014	Prevalence of vitamin D insufficiency and relationship between vitamin D levels infection and innate immunity	High prevalence of vitamin D deficiency<br>Serum vitamin D levels - no correlation with illness severity score
Ebenezer 2016	Determine vitamin D status and association with outcomes	Vitamin D deficiency common among pediatric patients<br>No association between vitamin D status and admission and mortality such as higher mortality and/or longer PICU stay<br>Deficiency associated with mechanical ventilation severity of illness, vasopressor need
Narang 2016	Identify an effective nutritional agent that reduces the need for antibiotics, duration of pneumonia and length of hospitalization would be highly cost-beneficial.	Severe vitamin D deficiency (<20 ng/ml), nonexclusive breastfeeding in the first six months of life, inadequate sun exposure found to be significant risk factors for ALRTI in Indian children.
Alvarez 2016	Study aimed to investigate relationship between vitamin D status and plasma markers of lutathione (GSH) and cysteine (Cys) redox and immunity in critically ill children	Vitamin D sufficiency was associated with more reduced plasma hCySS, indicative of lower oxidative stress, in critically ill children. Plasma GSH, GSSG, and glutamine, however, were lower in the vitamin D sufficient group. Vitamin D role in maintaining redox status during pediatric critical illness requires further study.
Cetinkaya 2015	Evaluate the effect of vitamin D levels on early-onset sepsis (EOS) in term infants	Lower maternal and neonatal 25-OHD levels associated with EOS. Suggest that adequate vitamin D supplementation during pregnancy may be helpful to prevent EOS in term neonates.
Ahmed 2014	Examine relationship between vitamin D status and hospitalization for ALRTI in Nigerian children.	ALRTI not associated with vitamin D status, but associated with less exposure to sunlight. Exposure to sunlight & vitamin D supplementation contributed to vitamin D status in this population.
Jat 2016	Evaluate Vitamin D levels and its correlation with severity and outcome of pneumonia in children.	Majority (86.4%) of children were vitamin D deficient. Vitamin D levels were found different in cases and controls and were not related to severity and outcome of pneumonia.
Sankar 2017	Evaluate association of severe vitamin D deficiency with clinically important outcomes in children with septic shock.	Prevalence of severe vitamin D deficiency is high in children with septic shock admitted to pediatric intensive care unit. Severe vitamin D deficiency at admission seems to be associated with lower rates of shock reversal at 24 hours of ICU stay.
Cizmeci 2015	Investigate if neonates with early-onset neonatal sepsis (EONS) had lower levels of vitamin D	Cord-blood 25(OH) D levels of neonates with EONS significantly lower than that of the healthy controls, and a low level of cord-blood vitamin D was found to be associated with an increased risk of EONS. Further studies are warranted to confirm this association.
Khakshour 2015	Clarify the association between vitamin D deficiency and acute respiratory infection in children below age 5 years.	The group of children with respiratory disorders, 9 (42.9%) exhibited vitamin D deficiency. No meaningful statistical relation vitamin D deficiency with acute respiratory infections (p>0.05)
Korwutthikulrangsri 2015	Determine vitamin D status in critically ill children and its relationship with adrenal function	Higher prevalence of vitamin D deficiency in critically ill children in comparison to controls. Patients vitamin D deficiency had higher median (IQR) PRISM III score and higher proportion of mortality than those with serum 25-OHD of equal or more than 12 ng/mL.
Elmoneim 2016	Association of the level of vitamin D on admission & length of stay in the PICU, or duration of mechanical ventilation.	High prevalence of vitamin D deficiency among PICU patients & significant association with increased LOS and need for mechanical ventilation. Not significant association with mortality rate.

Dinlen 2016	Determine the association between serum 25-hydroxy vitamin D [25(OH)D] levels and acute respiratory tract infections (ALRTI) in newborns	Lower blood 25(OH) D levels might be associated with increased risk of ALRTI in term newborn babies. Appropriate vitamin D supplementation during pregnancy and early childhood may enhance newborns' respiratory health.
Moreno-Solis 2015	Examine prevalence of hypovitaminosis D in infants with acute bronchiolitis compared with control subjects and to evaluate the relationship between serum 25-hydroxyvitamin D (25(OH) D) and the severity of bronchiolitis.	Prevalence of hypovitaminosis D is high in Spanish infants with bronchiolitis. The severity of acute bronchiolitis increases with a decline in serum 25 (OH) D level.
Inamo 2011	Relationship between serum vitamin D concentrations and severity of ALRI in hospitalized children in Japan.	Significantly more children with ALRI that required supplementary oxygen and ventilator management were vitamin D deficient. Findings suggest: immunomodulatory properties of vitamin D may influence the severity of ALRI.
Karatekin 2009	Association of serum 25-hydroxy vitamin D concentrations in newborns with acute lower respiratory infection (ALRI) and without clinical signs of rickets, and their mothers	Newborns with subclinical vitamin D deficiency may have an increased risk of suffering from ALRI. Strong positive correlation between newborns' and mothers' 25(OH) D concentrations indicates that adequate vitamin D supplementation of mothers should be emphasized during pregnancy especially in winter months.
Roth 2010	Investigate association between vitamin D status and ALRI	Vitamin D status associated with ALRI in early childhood.<br>Randomized trials needed to establish if interventions to improve vitamin D status could reduce burden of ALRI in early childhood.
Mathias 2017	Determination of association of 25(OH)D with cathelicidin and DBP (D binding protein) in children with sepsis	No association between vitamin D and cathelicidin or DBP (D binding protein) levels
Dhandai 2018	Assess vitamin D deficiency as possible risk factor for late-onset sepsis in term and late preterm neonates	Vitamin D deficient neonates are at greater risk for late onset sepsis compared to those with sufficient vitamin D levels
Vo 2018	Investigate the association between circulating 25-hydroxyvitamin D status upon admission and disease severity in infants hospitalized for bronchiolitis. Also, to determine if the association differs by the form of 25(OH)D (total, bioavailable or free 25 (OH) D.	Infants with total 25 (OH) D < 20 ng/ml had higher risk of intensive care and longer hospital length-of-stay
Li 2018	Assess vitamin D nutritional status of children of 3 days to 14 years and investigated the relationship between community-acquired pneumonia and serum 25(OH)D level	Children with low serum 25 (OH) D levels may be at higher risk of receiving mechanical ventilation and presenting with multiple organ dysfunction. Vitamin D supplementation could be beneficial for the treatment and prevention of CAP

Additional Table 9 Assay used in each study to measure Vitamin D levels

Assay	Paper	Total Number of studies
Radioimmunoassay	Madden 2012; Inamo 2011; Karatekin 2009; Roth 2009; Roth 2010; Sakka 2014; Moreno-Solis 2015; Khashour 2015	8
Competitive binding enzyme linked immunoassay	Basha 2014	1
Binding protein assay	Onwuneme 2015 (2)	1
Liquid chromatography-mass spectrometry	McNally 2012; Korwutthikulrangsri 2015; Binks 2014; Cetinkaya 2015; Cizmeci 2015; Asilioglu 2017; Onwuneme (1)	7
Chemiluminescence immunoassay or chemiluminescent tracer	Alonso 2015; Rey 2014; Sankar 2016; Shah 2016; Prasad 2015; Ahmed 2015; Lopez 2016; Alvarez 2016; Say 2017; Sankar 2017; Jat 2017; Mathias 2017; Li 2018	13
ELISA	Ponnarmeni 2016; Hebbar 2014; Elmoneim 2016; Narang 2016; Jia 2017; El-Gamasy 2017; Halwany 2017; Banajeh 2009; Badawi 2017	9
ELFA (enzyme linked fluorescent assay)	Bustos 2014	1
Electrochemiluminescence Immunoassay (ECLIA)	Dayal 2014, Ebenezer 2016, Garcia Soler 2017; Cayir 2014;	4
Clinical Laboratory Improvement Amendments–approved Vitamin D assay (Elecsys; Roche Diagnostics, Indianapolis, Indiana)	Hurwitz 2017	1
Commercial immunoassay lit (I RIA Diasorin UK)	Wayse 2004, Ayulo 2014,	2
Immunoassay analyzer	Rippel 2012	1
APPLIED 3200 Biosystem	Dinlen 2016	1
Not reported	Yaghmaie 2017; Dhandai 2018	2
Abbott Architect assay (Abbott, Waukegan, Illinois)	Vo 2018	1

Additional Table 10 Funding and ethical approval of included studies

Study	Funding	Approval of study and ethics
Madden 2012	Reported	Children's Hospital Boston institutional review board. Informed consent obtained from family.
Lopez 2016	Reported	Approved by the Ethical Committee of Clinical Investigation of Galicia (CEIC ref 010/015) and all of the regional ethics committees of the participant centres informed consent forms were obtained from either a parent or legal guardian for each subject before study inclusion.
Garcia-Soler 2017	Not reported	Study protocol approved by regional research ethics committee. Obtained informed consent from family of patients. Data recorded anonymously (encrypted electronic database).<br>Project adhered to the principles of Declaration of Helsinki and standards for good clinical practice.
McNally 2012	Reported	Research ethics board approval for vitamin D sub study obtained from 6 centers, representing 337 of the original 389 study participants.
Rippel 2012	Not Reported	Approved by Ethics Committee of The Royal Children's Hospital Melbourne
Alonso 2015	Reported	Regional Ethics Committee of the Principality of Asturias
Asilioglu 2017	Not reported	Approval of study by the Local Ethics Committee of Ondokuz Mayıs University (Samsun, Turkey).
Ayulo 2014	Not reported	Institutional Review Board: Montefiore Medical Centre
Rey 2014	Reported	Hospital Ethics Committee
Shah 2016	Reported	Ethical approval obtained from Institutional ethics committee. Parents of children satisfying criteria gave written informed consent for participation of their child in the study.
Ponnarmeni 2016	Not reported	The institution's ethics committee. Informed consent obtained
Onwuneme 2015 (1)	Reported	Approval by ethics committees of: Children's University Hospital, Temple Street and Our Lady's Children's Hospital, Dublin, Ireland. Participants were informed and provided written consent before recruitment.
Jia 2017	Not reported	Approved by Human Ethical Committee of Affiliated Hospital of Yan'an University, Yan'an. Informed consent was taken from mothers and/or parent of infants.
Halwany 2017	Not reported	Approval by the Medical Ethics Committee of the Faculty of Medicine, Alexandria University
Sankar 2016	Not reported	Institutional Ethics committee. Informed consent obtained
Say 2017	Not reported	Approval by the local Ethics Committee and informed parental consent was obtained for all infants.
Sakka 2014	Not reported	Not reported
Onwuneme2015 (2)	Reported	Ethics Committee of National Maternity Hospital. Informed written consent obtained from parents before recruitment
Dayal 2014	Not reported	Ethics Committee of the Institute. Informed consent obtained.
Bustos 2016	Reported	Comité Ético Científico del Servicio de Salud de Concepción
Hurwitz 2017	Reported	Informed consent obtained and study protocol approved by the Institutional Review Boards at the University of Tennessee, St Jude Research Hospital, and the Centers for Disease Control and Prevention.
Cayir 2014	Not reported	Approval from the institutional ethics committee and consent from the parents of all children in the study.
Badawi 2017	Not reported	Children's Cairo University institutional review board approved study. Informed consent obtained from parents
Yaghmaie 2017	Not reported	Study carried out in accordance with the Declaration of Helsinki, and the ethics committee of the Tehran University of Medical Sciences approved the protocols of the study. Records of patients' were kept confidential. Patients' consent provided

		for blood sampling and vitamin D serum level was assessed from patients' files, so no invasive method or extra blood sampling was done.
Basha 2014	Not reported	Oral consent from the mothers.
Prasad 2015	Not reported	Institutional review board approved the protocol. Written informed consent obtained from parents or guardians.
Wayse 2004	Reported	Information not provided
El-Gamasy 2017	Not reported	Study approved from the Ethical Committee of the Faculty of Medicine, Tanta University.<br>Informed verbal or written parental consents from all subjects involved in the study.
Banajeh 2009	Reported	Protocol approved and described in previous paper
Binks 2014	Reported	Testing performed after approval by the Human Research Ethics Committee of the Northern Territory Department of Health and Menzies School of Health Research
Roth 2009	Reported	Caregiver of each participant provided written informed consent and completed a questionnaire<br>Study approved by the Human Research Ethics Board of the University of Alberta Health Sciences Faculties
Hebbar 2014	Reported	Institutional Review Boards of Emory University and Children's Healthcare Atlanta<br>Informed consent obtained
Ebenezer 2016	Reported	Institutional Review Board (IRB), Informed consent from parents
Narang 2016	Not reported	Not reported
Alvarez 2016	Reported	Study approved by both the Emory University and Children's Healthcare of Atlanta Institutional Review Boards, and informed consent was obtained from patients' guardians prior to any study procedures
Cetinkaya 2015	Not reported	Study protocol approved by the local Ethics Committee. Informed parental consent was obtained for all infants
Ahmed 2014	Not reported	Informed written or oral consent obtained from the parents, and the study was approved by the National Hospital Abuja Ethics Committee
Jat 2016	Not reported	Ethics committee approval was taken before commencing the study. Parent's informed consent was taken before enrolling children into study.
Sankar 2017	No funding	Study approved by the institutional ethics committee.
Cizmeci 2015	Reported	Acquisition of cord-blood was approved by the local ethics committee of Fatih University Medical School.
Khakshour 2015	Not reported	Obtained informed consent from parents, data collection done using demographic questionnaire<br>and serum level of 25-dehydroxycalcciferol was measured. Samples taken by a trained nurse at admission based on physician's orders.
Korwutthikulrangsri 2015	Not reported	Ramathibodi Hospital Ethics Committee. Written informed consent from legal guardians of all participants
Elmoneim 2016	Reported	MCH hospital Ethics Committee approved study protocol
Dinlen 2016	Not reported	Study approved by the Local Ethics Committee. All parents fully informed about this investigation as well as its aim. Written consent was obtained from all parents.
Moreno-Solis 2015	Reported	Written informed consent obtained from parents or legal guardian of all enrolled children. Protocol of study approved by the Ethics Committee before the beginning of this study.
Inamo 2011	Not reported	Ethics Committee of Nihon University Nerima-Hikarigaoka Hospital. Informed consent obtained from the parents of all patients before inclusion in the study.
Karatekin 2009	Not reported	Study approved by the Institution's Ethics Committee, and informed consent was obtained from the study participants.
Roth 2010	Reported	Approved by The Johns Hopkins Bloomberg School of Public Health Institutional Review Board and the ethics committee of the Bangladesh Institute for Child Health at the Dhaka Shishu Hospital, Bangladesh. Signed permission prior to enrolment from parents/guardians.

Mathias 2017	Reported	The institution's Human Investigation Committee approved the study after a full board review and informed consent obtained
Dhandai 2018	Not reported	The institute's ethics committee approved the study and informed written consent had been given by their parents or guardians
Vo 2018	Reported	The institutional review boards at all participating sites approved the protocol and informed consent obtained from the infants' parents/legal guardians
Li 2018	Not reported	Protocols for the study and written consent approved by the ethics committee of the Capital Institute of Pediatrics at Beijing, China

Additional Table 11 Age groups of children in each study	
**First author, date**	**Age group**
Wayse 2004	<5 years
Karatekin 2009	Neonates
Roth 2009	<2 years
Roth 2010	1–18 months
Inamo 2011	<4 years
Madden 2012	<21 years
Rippel 2012	16.5 (3.1 to 75.2) months
McNally 2012	Newborn to 17 years
Ayulo 2014	1 to 21 years
Dayal 2014	3 months to 12 years
Hebbar 2014	0 to 18 years
Rey 2014	<16 years
Cetinkaya 2015	>37 weeks
Onwuneme (1) 2015	<12 years
Onwuneme (2) 2015	<32 weeks gestation
Prasad 2015	2 months to 12 years
Alonso 2015	1 month to 13 years
Korwutthikulrangsri 2015	79 (61) cases; 92 (40) controls months
Cizmeci 2015	Neonates
Shah 2016	1 month to 15 years
Ponnarmeni 2016	1 to 12 years
Sankar 2016	1 month to 17 years
Bustos 2016	>37 weeks and <15 years
Ebenezer 2016	<18 years
Elmoneim 2016	<14 years
Narang 2016	2 months to 5 years
Dinlen 2016	Neonates
Lopez 2016	0 to >48 months
Alvarez 2016	0 to 18 years
Garcia-Soler 2017	6 months to 17 years
Sankar 2017	<17 years
Ahmed 2015	2 to 60 months
Cayir 2014	1 to 13 years
Say 2017	<37 weeks
Asilioglu 2017	<=18 years
Basha 2014	<5 years
Jia 2017	<1 year
Jat 2017	1 month to 12 years
Yaghmaie 2017	Age range not stated
El-Gamasy 2017	3 months to 12 years
Binks 2014	<3 years
Halwany 2017	>1 month to <= 5 years
Badawi 2017	1 month to 12 years
Moreno-Solis 2015	1 to 11 months
Sakka 2014	<2 years
Hurwitz 2017	<5 years
Banajeh 2009	2 to 59 months
Khakshour 2015	< 5 years
Mathias 2017	<= 18 years
Dhandai 2018	Neonates
Vo 2018	< 12 months
Li 2018	3 days to 14 years

Additional Table 12 Prevalence of vitamin D deficiency in each study of acute and critically ill children (sorted from highest to lowest)

Study	Prevalence of vitamin D deficiency (%)	Number of vitamin D deficient children	Total number of children
Wayse 2004	95.00	76	80
Sakka 2014	92.70	89	96
Karatekin 2009	92.00	23	25
Onwuneme2015 (2)	91.48	86	94
Say 2017	87.00	87	100
Dinlen 2016	86.70	26	30
Cetinkaya 2015	84.00	42	50
Jat 2016	84.00	42	50
Roth 2010	84.00	21	25
Prasad 2015	83.75	67	80
Jia 2017	83.64	92	110
Shah 2016	83.11	128	154
Korwutthikulrangsri 2015	78.12	25	32
Sankar 2016	74.26	75	101
Sankar 2017	72.09	31	43
Cizmeci 2015	70.00	28	40
Mathias 2017	70.00	28	41
McNally 2012	69.02	225	326
Basha 2014	65.43	53	81
Yaghmaie 2017	64.63	53	82
Dhandai 2018	63.30	38	60
Hebbar 2014	60.66	37	61
Onwuneme 2015 (1)	59.16	71	120
Alvarez 2016	58.00	29	50
Cayir 2014	56.82	50	88
Elmoneim 2016	56.67	17	30
Narang 2016	56.00	28	50
Li 2018	55.83	445	797
Ponnarmeni 2016	50.81	63	124
Asilioglu 2017	48.00	120	250
Badawi 2017	44.30	39	88
Garcia-Soler 2017	43.82	149	340

Bustos 2016	43.30	39	90
Ebenezer 2016	40.38	21	52
Madden 2012	40.12	205	511
Halwany 2017	37.25	38	102
Banajeh 2009	36.71	29	79
Rippel 2012	34.49	109	316
El-Gamasy 2017	32.50	26	80
Ahmed 2014	30.00	15	50
Rey 2014	29.49	46	156
Vo 2018	29.33	298	1,016
Inamo 2011	28.50	8	28
Dayal 2014	25.00	23	92
Khakshour 2015	24.32	9	37
Lopez 2016	24.20	84	347
Alonso 2015	15.63	45	288
Ayulo 2014	15.63	61	216
Binks 2014	14.86	11	74
Moreno-Solis 2015	13.33	9	48
Hurwitz 2017	12.22	11	90
Roth 2009	4.69	3	64

Additional Table 13 Characteristics of studies used in the meta-analysis of prevalence

Study	Total number of patients	Total number of vitamin D deficient patients	Country, setting	Age range	Design	Quality score (NOS)
Vo 2018	1,016	298	USA, PICU	< 12 months	cohort	8
Li 2018	797	445	China, PICU	3 days to 14 years	case-control	8
Madden 2012	511	205	Boston US, PICU	< 21 years	cohort	8
Lopez 2016	347	84	Spain, hospitalised	0 to >48 months	cohort	8
Garcia-Soler 2017	340	149	Spain, PICU	6 months to 17 years	cohort	7
McNally 2012	326	225	Canada, PICU	newborn to 17 years of age	cohort	7
Rippel 2012	316	109	Australia, PICU	16.5 (3.1–75.2) months	cohort	8
Alonso 2015	288	45	Spain, PICU	1 month to 13 years	cross sectional	5
Asilioglu 2017	250	120	Turkey, PICUs	<= 18 years	cohort	6
Ayulo 2014	216	61	Spain, PICU	1 month to 13 years	cross sectional	5
Rey 2014	156	46	Spain, PICU	<16 years	cohort	7
Shah 2016	154	128	India,PICU	1 month-15 years	cohort	6
Ponnarmeni 2016	124	63	India, PICU	aged 1–12 years	cohort	6
Onwuneme 2015 (1)	120	71	Ireland, PICU	<12 years old	cohort	8
Jia 2017	110	92	China, PICU	infants <1 years old	case-control	7
Halwany 2017	102	38	Alexandria, PICU	>1 month to ≤ 5 years	cohort	6
Sankar 2016	101	75	India, PICU	1 month to 17 years	cohort	8
Say 2017	100	87	Turkey, NICU	gestational age <37 weeks	case-control	6
Sakka 2014	96	89	Egypt, hospitalized	infants (<2 years old)	case-control	8
Onwuneme 2015 (2)	94	86	Ireland, NICU	preterm infants at <32 weeks gestation	cohort	8
Dayal 2014	92	23	India, PICU	3 months to 12 years	cohort	6
Bustos 2016	90	39	Chile, PICU	>37 weeks and <15 years	cohort	7
Hurwitz 2017	90	11	USA, hospitalised	<5 years old	cohort	6
Cayir 2014	88	50	Turkey, PICU	1 to 13 years	case-control	7
Badawi 2017	88	39	Cairo, Egypt PICU	1 month to 12 years	cohort	7
Yaghmaie 2017	82	53	Iran, PICU	children undefined	cross sectional	4
Basha 2014	81	53	Cairo Egypt, PICU	<5 years old	case-control	7
Prasad 2015	80	67	India, PICU	2 months-12 years	cohort	7
Wayse 2004	80	76	Indapur India, PICU	<5 years	case-control	6
El-Gamasy 2017	80	26	Egypt, PICU	3 months to 12 years	cohort	7
Banajeh 2009	79	29	Iran, hospitalised	aged 2–59 months	cohort	7
Binks 2014	74	11	Australia, PICU	<3 years old	cross sectional	6
Roth 2009	64	3	Canada, PICU	aged 1-25 months	case-control	7
Hebbar 2014	61	37	Atlanta, PICU	0 to 18 years	cohort	6
Dhandai 2018	60	38	India, NICU	neonates	cohort	7

Ebenezer 2016	52	21	India, PICU	<18 years	cohort	6
Narang 2016	50	28	Punjab, India, PICU	2 months to 5 years	case-control	6
Alvarez 2016	50	29	Atlanta, PICU	0 to 18 years	cross sectional	6
Cetinkaya 2015	50	42	Instabul/Turkey, NICU	neonates	case-control	6
Ahmed 2014	50	15	Nigeria, PICU	2–60 months	case-control	8
Jat 2016	50	42	India, PICU	1 month to 12 years	case-control	8
Sankar 2017	43	31	India, PICU	<=17 years	cohort	8
Mathias 2017	41	28	USA, PICU	<= 18 years	cohort	6
Cizmeci 2015	40	28	Instabul/Turkey, NICU	neonates	case-control	7
Khakshour 2015	37	9	Iran, hospitalized	<5 years	case-control	5
Korwutthikulrangsri 2015	32	25	Bangkok, PICU	months not more specific range	cohort	7
Elmoneim 2016	30	17	Saudi Arabia, PICU	<14 years	cohort	7
Dinlen 2016	30	26	Ankara Turkey, NICU	neonates	case-control	6
Moreno-Solis 2015	48	9	Spain, PICU	infants 1–11 months	case-control	6
Inamo 2011	28	8	Tokyo Japan, PICU	1-48 months	cohort	6
Karatekin 2009	25	23	Istanbul Turkey, NICU	neonates	case-control	6
Roth 2010	25	21	Bangladesh	1-18 months	case-control	8

Studies arranged from largest to smallest total sample size. NOS = Newcastle Ottawa Score

Additional Table 14 Sensitivity analyses for prevalence of vitamin D deficiency in acute and critically ill children

Patient category	Number of studies (Total number of individuals; number of vitamin D deficient individuals)	Pooled proportion % (95% CI) Random effects model	95% PI	Pooled proportion % (95% CI) Fixed effects model	Heterogeneity (I^2) % (95% CI)	Q value, d.f. p-value for heterogeneity	Median % (IQR) of vitamin D deficiency
Acute and critically ill children, excluding studies that used other thresholds	40 (6,543; 3,000)	53.0 (46.4-59.5)	17.7-85.5	45.1 (43.8-46.5)	95.5 (94.5-96.2)	859.8, 39, < 0.0001	55.9 (33.4-55.9)
Sample size >= 82 (large)	26 (6,094; 2,731)	51.5 (43.6-59.4)	16.5-85.2	44.0 (42.6-45.3)	96.8 (96.0-97.4)	773.1, 25, < 0.0001	46.2 (30.7-67.9)
Sample size < 82 (small)	26 (1,340; 742)	58.2 (47.5-68.2)	13.2-92.8	54.8 (51.7-58.0)	90.9 (87.9-93.2)	275.4, 25, < 0.0001	62.0 (33.6-82.3)
Cohort studies	30 (5,119; 2,197)	49.6 (42.7-56.4)	17.6-81.9	42.1 (40.7-43.6)	94.9 (93.6-95.9)	567.0, 29, < 0.0001	44.1 (33.0-67.1)
Case-control	18 (1,821; 1,138)	68.1 (56.5-77.8)	19.3-95.0	60.8 (58.2-63.3)	93.0 (90.4-94.9)	243.9, 17, 0.0001	76.8 (55.9-86.0)
Cross sectional	4 (494; 138)	34.8 (12.8-66.0)	0.10-99.6	30.3 (25.9-35.2)	96.7 (94.0-98.2)	90.4, 3, < 0.0001	36.8 (15.4-59.7)
Studies from India	11 (886; 592)	68.9 (54.9-80.1)	18.3-95.6	64.0 (60.4-67.5)	96.7 (94.0 98.2)	140.2, 10, < 0.0001	72.1 (53.4-83.4)
Studies from Turkey	7 (583; 376)	76.3 (60.9-87.0)	20.9-97.5	61.0 (56.6-65.2)	91.1 (84.2-95.0)	67.5, 6, < 0.0001	84 (63.4-86.8)
Studies that only recruited neonates	7 (399; 330)	83.0 (73.1-90.0)	42.4-97.0	80.7 (76.1-84.5)	76.6 (51.0-88.9)	25.7, 6, 0.0003	86.7 (77.0-89.2)
Studies with children of all other ages except neonates	45 (7,035; 3,143)	49.7 (43.5-55.8)	16.1-83.5	44.2 (43.0-45.5)	95.2 (94.3-96.0)	919.9, 44, < 0.0001	48.0 (29.5-68.3)

CI = confidence intervals; I^2 = heterogeneity; df = degrees of freedom. Vitamin D deficiency defined in our study as <50 nmol/L (20 ng/mL)
I^2 statistic used to estimate heterogeneity between pooled studies: I^2 >= 75% was considered as high heterogeneity, PI= Prediction interval; IQR = interquartile range: Lower (Q1) to Upper (Q3) quartile

Additional Table 15 Multivariate meta-regression model for prevalence

Predictors	k	b-coefficient	se	t-value	p-value	ci.lb	ci.ub	F-value	I^2 (%)	R^2 (%)	QE
Full model: year + clinical setting + quality score + design + country group + total sample size	52							4.57	95.7	32.9	702.6, p < 0.0001
year		-0.015	0.058	-0.256	0.799	-0.132	0.103				
total study sample size		-0.001	0.001	-0.731	0.469	-0.002	0.001				
country group (group 1 or 2 versus group 3)		1.009	0.356	2.831	0.007	0.291	1.726				
clinical setting (NICU versus PICU)		-1.573	0.465	-3.384	0.002	-2.510	-0.637				
design group (cohort vs other i.e. case-control or cross sectional)		0.482	0.324	1.488	0.144	-0.170	1.135				
quality score		0.296	0.170	1.744	0.088	-0.046	0.637				

k = number of outcomes included in the model fitting; se = standard errors of the coefficients; ci.lb = lower bound of the confidence intervals for the coefficients; ci.ub = upper bound of the confidence intervals for the coefficients; QE = test statistic for the test of (residual) heterogeneity; I^2 = residual heterogeneity / unaccounted variability; R^2 (amount of heterogeneity accounted for; PICU = pediatric intensive care units, NICU = neonatal intensive care units

Additional Table 16 Characteristics of studies included in the meta-analysis for prevalence in individuals with sepsis

Study	Total number of patients with sepsis	Total number of vitamin D deficient patients with sepsis	Country, setting	Age	Design	Quality score (NOS)
Shah 2016	160	135	India, PICU	1 month to 16 years	cohort	6
Asilioglu 2017	120	20	Turkey, PICU	1 month to ≤18 years	cohort	7
Say 2017	100	87	Turkey, NICU	preterm infants at <37 weeks gestation	case-control	6
Dhandai 2018	60	38	India, NICU	neonates	cohort	7
Ponnarmeni 2016	62	32	India, PICU	1 to 12 years	cohort	6
McNally 2012	48	33	Canada, PICU	newborn to 17 years	cohort	7
Onwuneme 2015 (2)	46	32	Ireland, NICU	preterm infants at <32 weeks gestation	cohort	8
El-Gamasy 2017	46	20	Egypt, pediatric emergency department	3 months to 12 years	cohort	7
Sankar 2017	43	31	India, PICU	<=17 years	cohort	8
Mathias 2017	41	28	USA, PICU	<=18 years	cohort	6
Cizmeci 2015	40	28	Turkey, NICU	neonates	case-control	7
Onwuneme 2015 (1)	35	32	Ireland, PICU	<12 years old	cohort	8
Hebbar 2014	30	17	Atlanta, PICU	0 to 18 years	cohort	6
Ebenezer 2016	16	8	India, PICU	<18 years	cohort	6
Korwutthikulrangsri 2015	12	4	Bangkok, PICU	moths (<8/9)	cohort	7
Prasad 2015	11	9	India, PICU	2 months to 12 years	cohort	7
Bustos 2016	10	7	Chile, PICU	>37 weeks and < than 15 years	cohort	7
Dayal 2014	9	4	India, tertiary care hospital	3 months to 12 years	cohort	6

Additional Table 17 Sensitivity analyses for prevalence of vitamin D deficiency in acute and critically ill children with sepsis

Patient category	Number of studies (Total number of individuals; number of vitamin D deficient individuals)	Pooled proportion (%, 95% CI) Random effects	95% PI	Pooled proportion (%, 95% CI) Fixed effects	Heterogeneity (I^2) %, 95% CI	Q value, d.f. p-value for heterogeneity	Median % (IQR) of vitamin D deficiency
Excluding studies that used other thresholds	14 (654; 395)	62.0 (47.3-74.7)	13.3-94.5	60.4 (56.0-64.7)	89.7 (84.5-93.2)	126.5, 13, < 0.0001	65.8 (50.4-70.0)
Sample size >= 42 (large)	9 (685; 428)	63.2 (44.6-78.5)	9.4-96.6	62.5 (58.2-66.6)	94.3 (91.1-96.3)	139.2, 8 < 0.0001	68.8 (51.6-72.1)
Sample size < 42 (small)	9 (204; 137)	64.7 (52.5-75.3)	29.5-89.0	64.7 (57.4-71.4)	57.9 (11.8-79.9)	19.0, 8, 0.0148	68.3 (50.0-70.0)
Cohort studies	16 (749; 450)	61.4 (48.6-72.8)	15.5-93,2	60.0 (55.9-63.9)	88.8 (83.5-92.4)	134.2, 15, < 0.0001	65.8 (48.6-70.5)
Case-control	2 (140; 115)	80.0 (58.8-91.8)	NA	81.0 (73.3-86.9)	81.3 (20.5-95.6)	5.35, 1, 0.0207	78.5 (74.2-82.8)
Studies from India	7 (361; 257)	66.0 (51.4-78.1)	21.3-93.3	69.2 (63.9-74.1)	81.1 (61.8-90.6)	31.7, 6 < 0.0001	63.3 (50.8-77.0)
Studies from Turkey	3 (260; 135)	59.2 (13.6-93.1)	0.00-100	51.2 (43.2-59.3)	97.8 (95.8- 98.8)	90.0, 2, < 0.0001	70.0 (43.3-78.5)
Studies that only included neonates	4 (246; 185)	73.7 (60.3-83.8)	15.9-97.7	73.5 (67.3-78.9)	76.0 (34.1-91.3)	12.5, 3, 0.0058	69.8 (68.0-74.3)
Studies that included children of all other ages except neonates	14 (643; 380)	60.7 (45.5-74.0)	11.8-94.7	58.7 (54.2-63.0)	90.1 (85.2-93.4)	131.8, 13 < 0.0001	62.5 (45.8-71.6)

CI = confidence intervals; I^2 = heterogeneity; df = degrees of freedom. Vitamin D deficiency defined in our study as <50 nmol/L (20 ng/mL). I^2 statistic used to estimate heterogeneity between pooled studies: I^2 >= 75% was considered as high heterogeneity; PI= Prediction interval; IQR = interquartile range: Lower (Q1) to Upper (Q3) quartile; NA= Not available

Additional Table 18 Sensitivity analyses for mortality. Pooled odds ratios for risk of mortality in deficient versus not deficient children

Patient category	Number of studies (Total number of individuals)	Pooled OR (95% CI) Vitamin D deficient/ Vitamin D non-deficient p-value		Heterogeneity (I^2) % (95% CI)	Q value, d.f. p-value for heterogeneity	Eggers p-value
		Random effects	Fixed effects			
Excluding studies that used other thresholds	14 (2,030)	1.59 (1.05-2.41) p-value = 0.028	1.52 (1.08-2.13) p-value = 0.016	24.3 (0.0-59.9)	17.18, 13, 0.1910	p-value = 0.120
Studies from India	7 (646)	1.08 (0.70-1.69) p-value = 0.710	1.08 (0.70-1.69) p-value = 0.710	0.0 (0.0-62.4)	4.56, 6, 0.589	Number of studies too small to test for small study effects (k.min=10)

CI = Confidence Intervals; I^2 = I squared statistic used to estimate heterogeneity (inconsistency); df = degrees of freedom, results reported in 1 decimal place; OR= odds ratio. Vitamin D deficiency defined as < 50 nmol/L or 20 ng/ml. We used the I^2 statistic to estimate heterogeneity between pooled studies: I^2 >= 75% was considered as high heterogeneity.

Additional Figures

Additional Figure 1 Funnel plot of studies of prevalence of vitamin D deficiency in acute and critically ill children. Horizontal axis shows logit transformed proportion and the standard error of the logit transformed proportion is plotted on the vertical axis. Each dot represents an individual study and the vertical line represents the pooled proportion from a random-effects meta-analysis. The diagonal lines represent pseudo 95% confidence limits (p=0.01, Egger's test).

Plot A shows the funnel plot before trim and fill method was applied and B after. Solid circles in plot B represent original data and open circles represent imputed filled studies (11 studies added) on the left-hand side of the funnel plot.

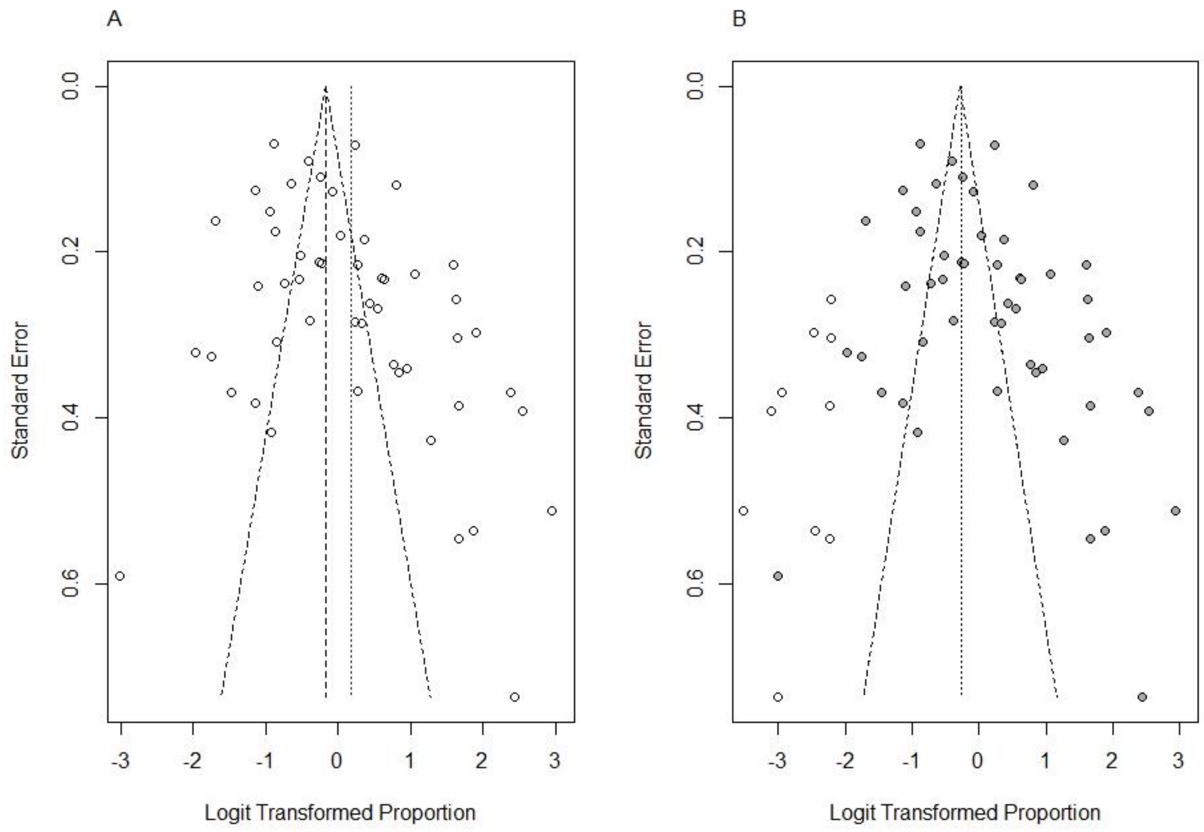

Additional Figure 2. Pooled prevalence estimate for vitamin D deficiency in acute and critically ill children (by country group). Forest plot shows results from the random effects model. Diamonds represent pooled proportion of vitamin D deficiency for the countries in each subgroup (group 1, group2, group 3). The diamond at the bottom shows the overall pooled estimate of all the 52 studies. Each square shows the prevalence estimate of each study and the horizontal line across each square represents the 95% confidence interval (CI) of the prevalence estimate.

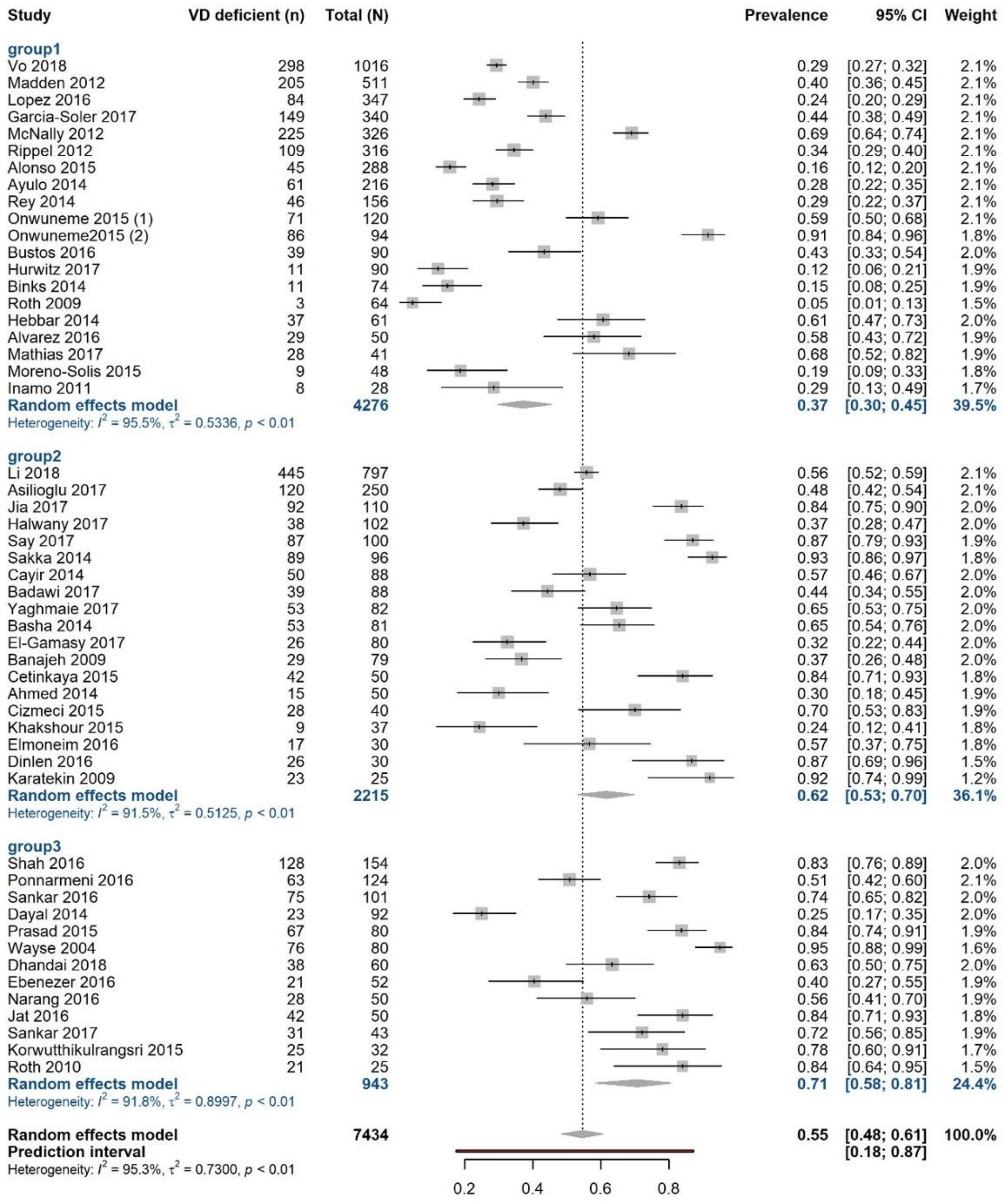

Additional Figure 3. Pooled prevalence estimates for vitamin D deficiency in acute and critically ill children (neonates versus all other age groups). Forest plot shows results from the random effects model. Diamonds represent pooled proportion of vitamin D deficiency for the studies in neonates and all other age groups. The diamond at the bottom shows the overall pooled estimate of all the 52 studies. Each square shows the prevalence estimate of each study and the horizontal line across each square represents the 95% confidence interval (CI) of the prevalence estimate.

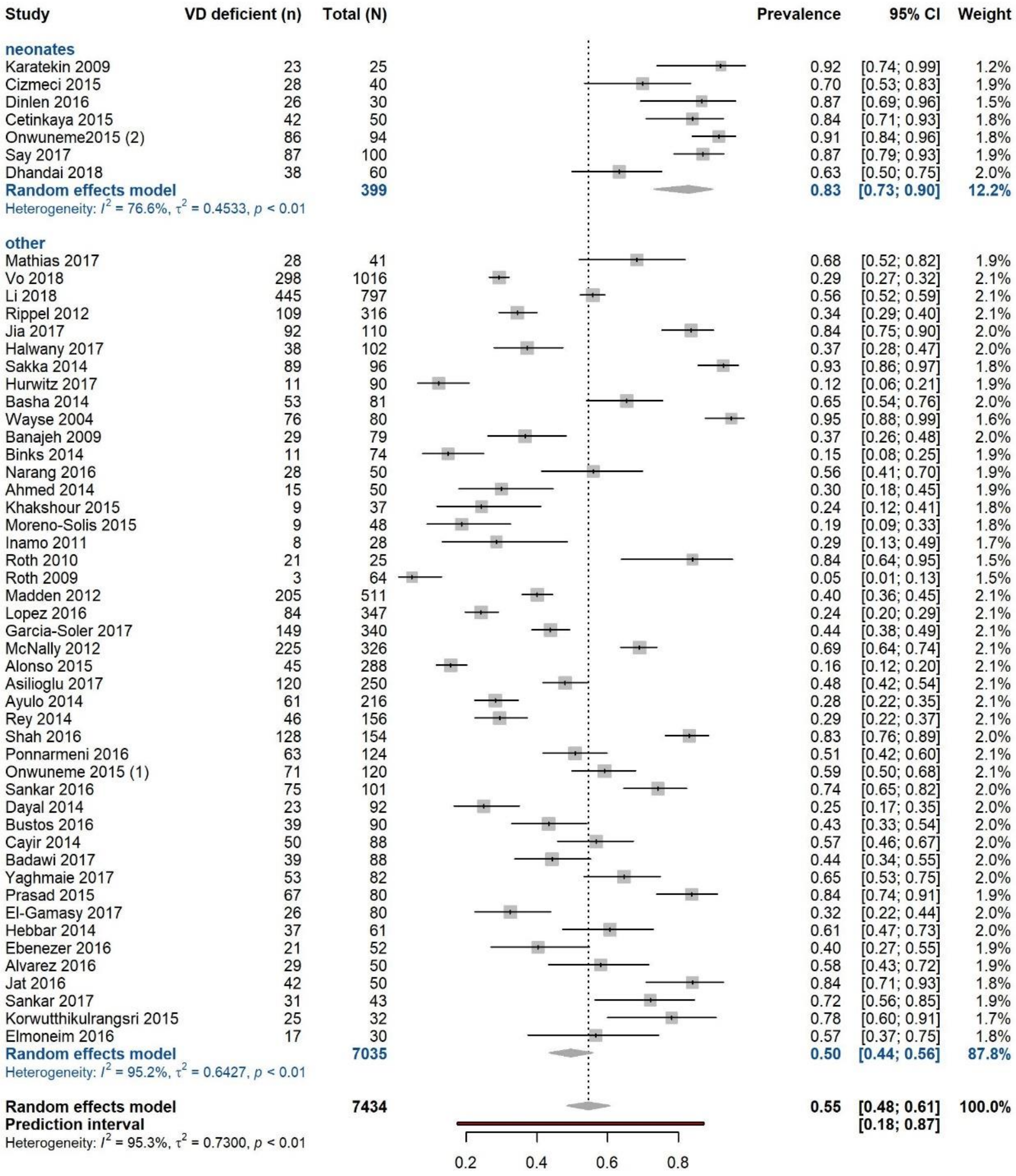

Additional Figure 4 Bubble plots of univariate meta-regressions. Each study is represented by a circle. Predictor variables: A study design, B year of publication, C quality score and D total children are plotted on the x-axis with the effect measure logit transformed proportion shown on the vertical (y-axis).

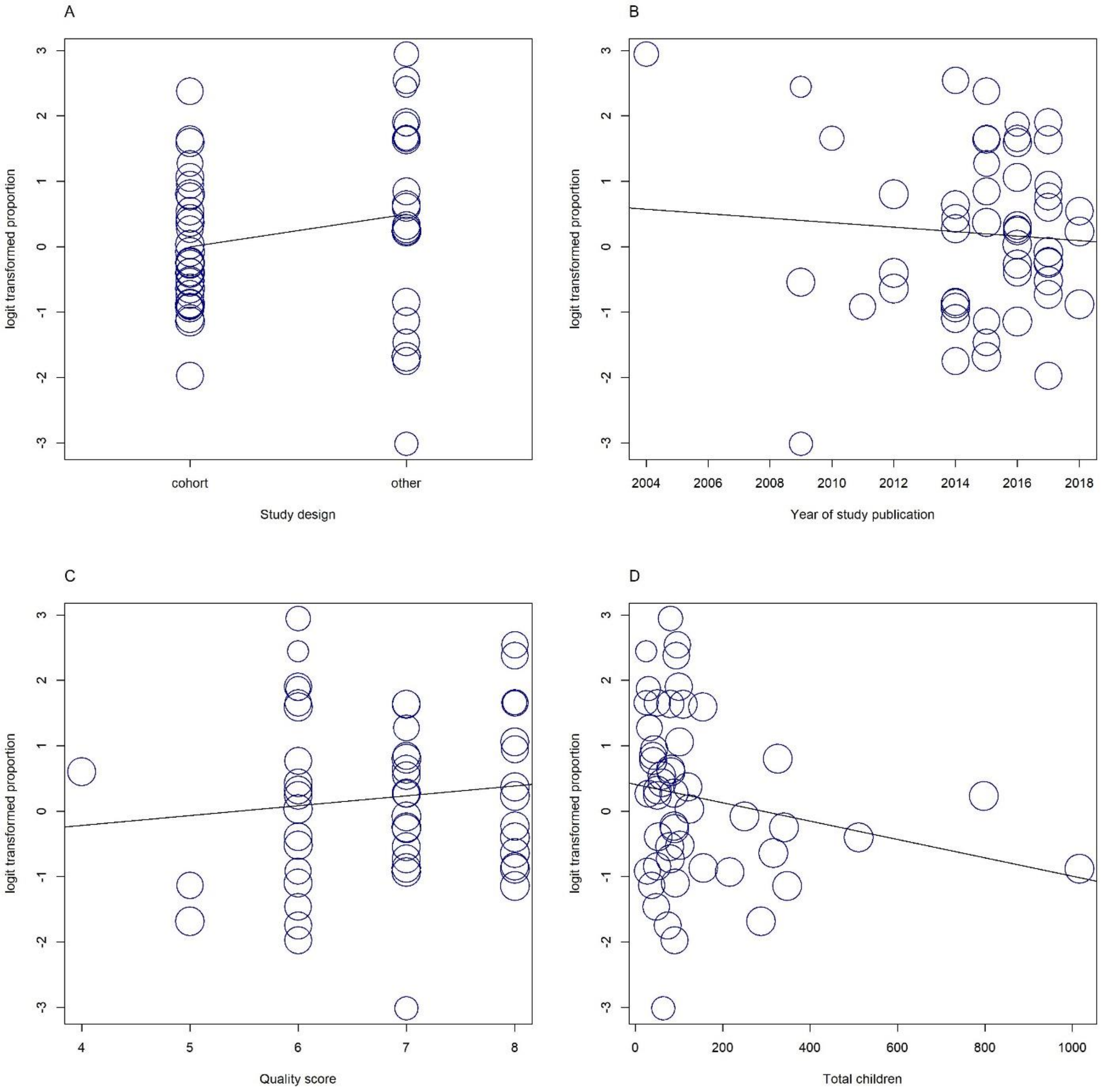

Additional Figure 5 Funnel plot for prevalence of vitamin D deficiency in acute and critically ill children with sepsis. Horizontal axis shows logit transformed proportion and the standard error of the logit transformed proportion is plotted on the vertical axis. Each dot represents an individual study and the vertical line represents the pooled proportion from a random-effects meta-analysis. The diagonal lines represent pseudo 95% confidence limits (p=0.81, Egger's test).

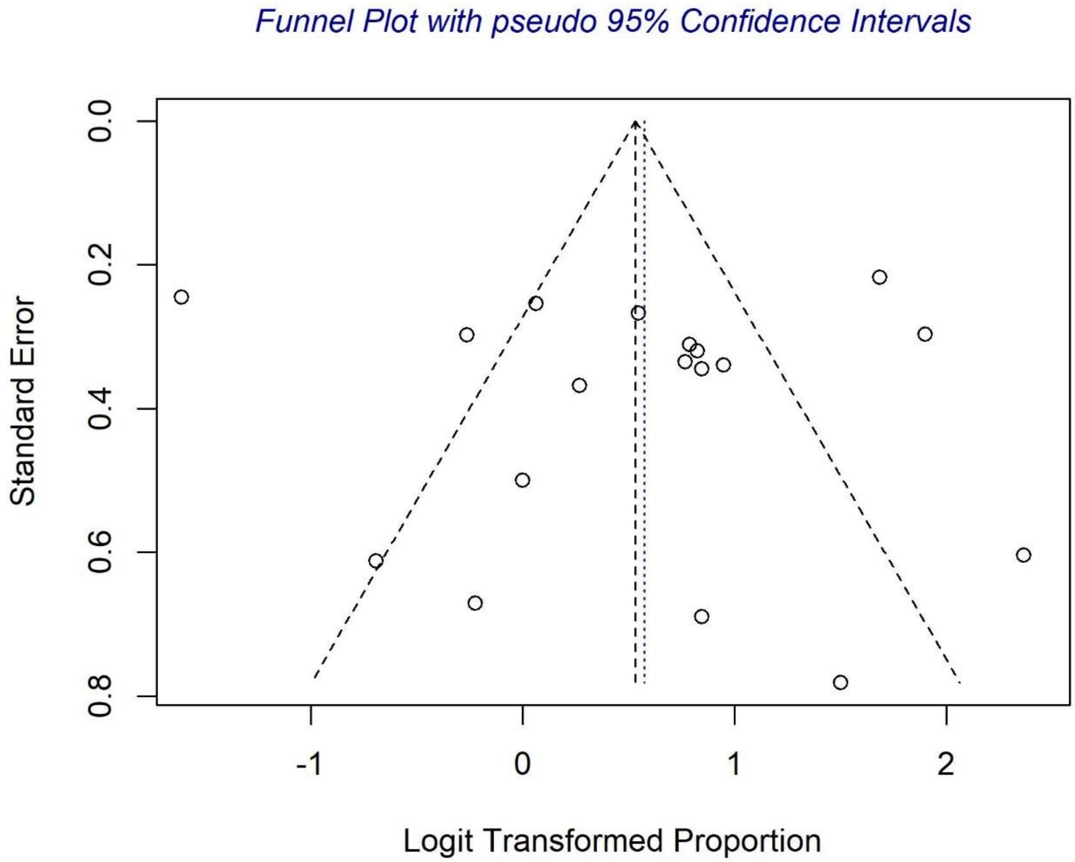

Additional Figure 6 Pooled prevalence estimate for vitamin D deficiency in acute and critically ill children with sepsis (subgroup analysis by study design). Forest plot shows results from the random effects model. Diamonds represent the pooled proportion of vitamin D deficiency for the studies in each subgroup (case-control and cohort). The diamond at the bottom shows the overall pooled estimate of all the 18 studies. Each square shows the prevalence estimate of each study and the horizontal line across each square represents the 95% confidence interval (CI) of the prevalence estimate.

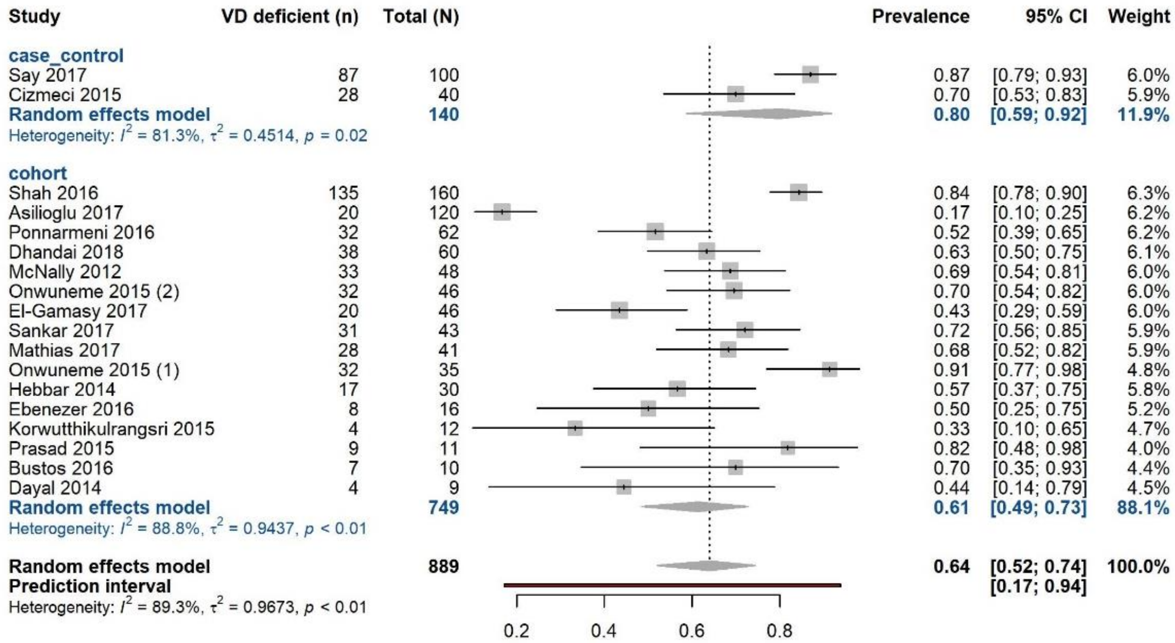

Additional Figure 7 Funnel plot of risk of mortality in vitamin D deficient versus vitamin D non-deficient acute and critically ill children. Horizontal axis shows logit transformed odds ratio and the standard error of the log odds ratio is plotted on the vertical axis. Each dot represents an individual study and the vertical line represents the overall pooled odds ratio from random-effects meta-analysis. The diagonal lines represent pseudo 95% confidence limits (p=0.084, Egger's test).

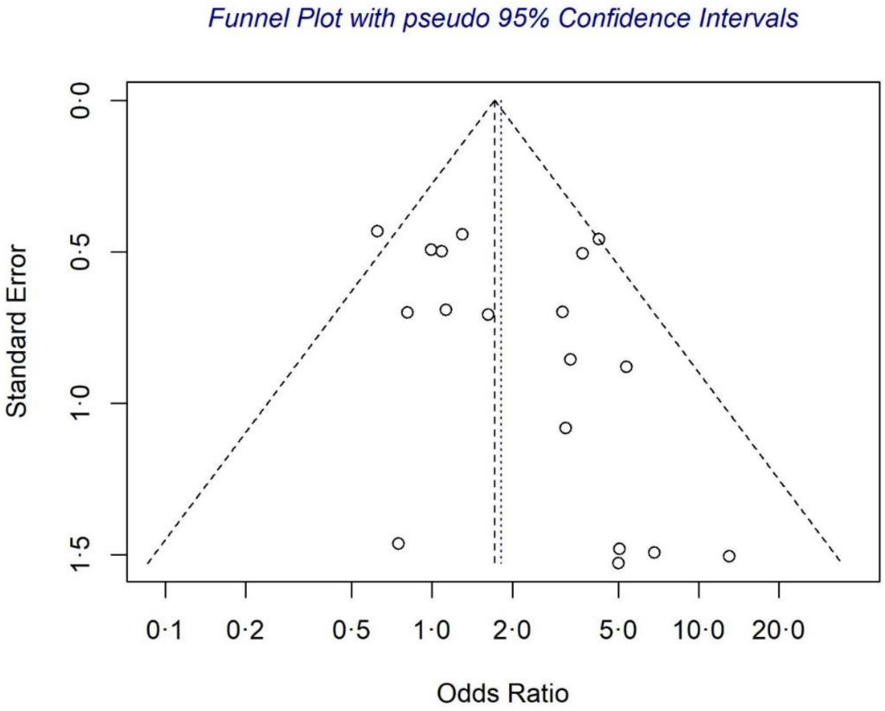

Additional Figure 8 Pooled odds ratio (OR) and 95% CI for risk of mortality in vitamin D deficient versus vitamin D non-deficient acute and critically ill children (fixed effects model). Diamond represents the overall odds ratio (with corresponding 95% Confidence Interval). Each square shows the odds ratio of each study and the horizontal line across each square represents the 95% confidence interval (CI) of the estimate.

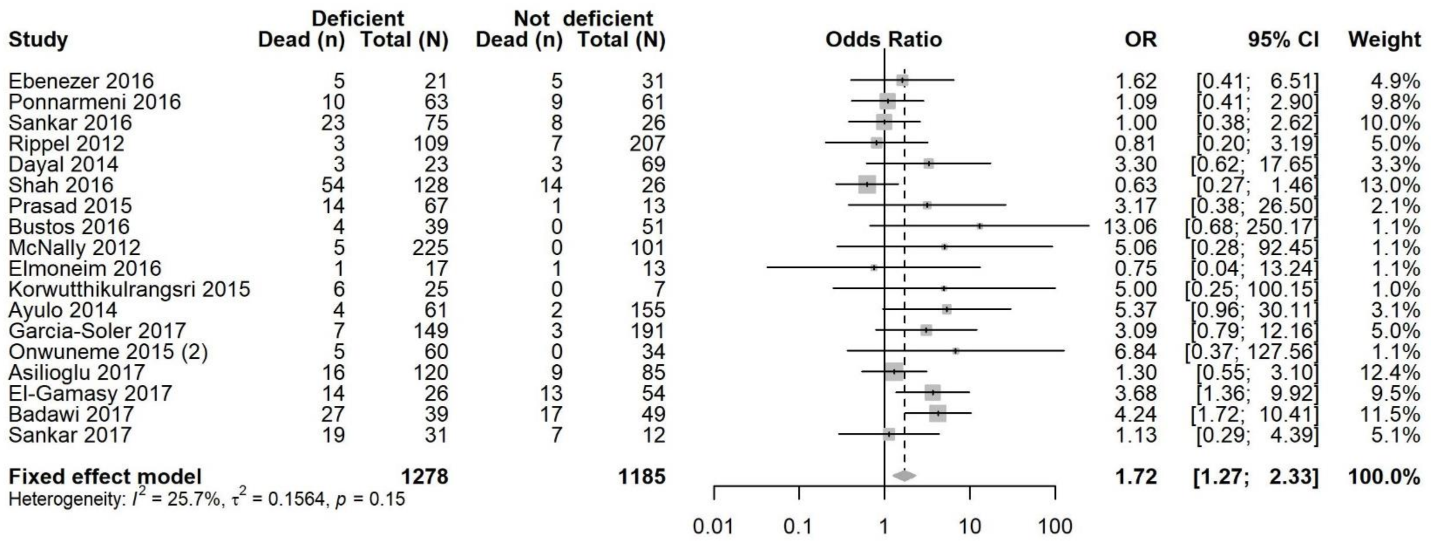

Supplement: Supplementary file 1 [file bmjopen-2018-027666supp001.pdf]
