# Supplementary material for: Importance of vitamin D in acute and critically ill children with subgroup analyses of sepsis and respiratory tract infections: a systematic review and meta-analysis
Source: BMJ Open. 2019 May 22;9(5):e027666. doi: 10.1136/bmjopen-2018-027666 (PMC6538078; doi:10.1136/bmjopen-2018-027666)
Supplement: Author's manuscript [file bmjopen-2018-027666.draft_revisions.pdf]

BMJ Open

BMJ Open is committed to open peer review. As part of this commitment we make the peer review history of every article we publish publicly available.

When an article is published we post the peer reviewers' comments and the authors' responses online. We also post the versions of the paper that were used during peer review. These are the versions that the peer review comments apply to.

The versions of the paper that follow are the versions that were submitted during the peer review process. They are not the versions of record or the final published versions. They should not be cited or distributed as the published version of this manuscript.

BMJ Open is an open access journal and the full, final, typeset and author-corrected version of record of the manuscript is available on our site with no access controls, subscription charges or pay-per-view fees (http://bmjopen.bmj.com).

If you have any questions on BMJ Open's open peer review process please email info.bmjopen@bmj.com

# Importance of vitamin D in critically ill children with subgroup analyses of sepsis and respiratory tract infections: a systematic review and meta-analysis

| | |
|---|---|
| Journal: | *BMJ Open* |
| Manuscript ID | bmjopen-2018-027666 |
| Article Type: | Research |
| Date Submitted by the Author: | 01-Nov-2018 |
| Complete List of Authors: | Cariolou, Margarita; Imperial College London School of Public Health, Epidemiology and Biostatistics<br>Cupp, Meghan; Imperial College London School of Public Health, Epidemiology and Biostatistics<br>Evangelou, Evangelos; Imperial College London School of Public Health, Epidemiology and Biostatistics; University of Ioannina School of Medicine, Department of Hygiene and Epidemiology<br>Tzoulaki, Ioanna; Imperial College London, Epidemiology and Public Health; University of Ioannina School of Medicine, Department of Hygiene and Epidemiology<br>Berlanga-Taylor, Antonio; Imperial College London School of Public Health, Epidemiology and Biostatistics |
| Keywords: | Vitamin D, EPIDEMIOLOGY, meta-analysis, sepsis, PAEDIATRICS, INTENSIVE & CRITICAL CARE |
| | |

SCHOLARONE™
Manuscripts

# Importance of vitamin D in critically ill children with subgroup analyses of sepsis and respiratory tract infections: a systematic review and meta-analysis

Margarita Cariolou[1] MPH, Meghan A. Cupp[1] MPH, Evangelos Evangelou[1,2] PhD, Ioanna Tzoulaki[1,2] PhD and Antonio J. Berlanga-Taylor[1]* DPhil

[1]MRC-PHE Centre for Environment and Health, Department of Epidemiology & Biostatistics, School of Public Health, Faculty of Medicine, Imperial College London, St Mary's Campus, Norfolk Place, UK, LONDON W2 1PG

[2]Department of Hygiene and Epidemiology, University of Ioannina Medical School, Ioannina, Greece

*Correspondence should be addressed to Dr. Antonio J. Berlanga-Taylor (a.berlanga@imperial.ac.uk), MRC-PHE Centre for Environment and Health, Department of Epidemiology & Biostatistics, School of Public Health, Faculty of Medicine, Imperial College London, St Mary's Campus, Norfolk Place, LONDON W2 1PG.

For peer review only

4364 words (excluding title page, abstract, references, figures and tables)

## ABSTRACT

**Objective:** Critical care and sepsis remain high priority concerns in children. Associations between deficiency and mortality, particularly in children with sepsis, remain unclear. We performed a systematic review and meta-analysis of observational studies to address this uncertainty.

**Methods:** We searched PubMed, OVID and Google Scholar to obtain pooled prevalence estimates of vitamin D deficiency and odds ratios for mortality in critically ill children treated in intensive care units, with subgroup analysis for those with sepsis and those with respiratory tract infections.

**Results:** Forty-eight studies were included. Of 5,520 critically ill children, 2,664 (48%) were vitamin D deficient (< 50 nmol/L). Pooled prevalence estimate of vitamin D deficiency was 54.9% (95% CI 48.0-61.6, $I^2$=95.0%, 95% CI 94.0-95.8, p < 0.0001). Prevalence of deficiency in children with sepsis (16 studies, 788 total individuals) was 63.8% (95% CI 49.9-75.7, $I^2$=90.5%, 95% CI 86.2-93.5%, p < 0.0001) and 49.9% (95% CI 37.6-62.2; $I^2$ = 93.9%, 95% CI 92.1-95.3, p < 0.0001) in those with respiratory tract infections (24 studies, 1,683 total individuals). Meta-analysis of mortality (18 studies, 2,463 total individuals) showed increased risk of death in vitamin D deficient critically ill children both with random (OR 1.81, 95% CI 1.24-2.64, p = 0.002) and fixed effects (OR 1.72, 95% CI 1.27-2.33, p= 0.0005) models with low heterogeneity ($I^2$= 25.7%, 95% CI 0.0-58.0, p = 0.153). There were insufficient studies to perform meta-analyses for sepsis and respiratory tract infection related mortality.

**Conclusions**: Circulating vitamin D deficiency is common amongst critically ill children, particularly in those with sepsis. Our results suggest that vitamin D deficiency in critically ill children is associated with increased mortality. Clinical trials, studies with larger sample sizes and




For peer review only - http://bmjopen.bmj.com/site/about/guidelines.xhtml

standardized approaches are needed to further assess associations between circulating vitamin D levels and mortality and other outcomes in the paediatric population.

**Registration** PROSPERO (CRD42016050638)

**Copyright** Open access article under terms of CC BY

**Keywords** paediatric, vitamin D, intensive care, sepsis, meta-analysis, prevalence, mortality, systematic review, respiratory tract infections

## Strengths and limitations of this study

- We comprehensively assessed the magnitude and relevance of vitamin D circulating levels in paediatric acute and critically ill patients using a large number of studies with large total sample size with pre-specified sub-group and sensitivity analyses. We used PRISMA and MOOSE guidelines for reporting.
- We used the currently recommended cut-off of less than 50 nmol/L for vitamin D deficiency.
- We did not find enough studies to perform meta-analyses for mortality from sepsis or respiratory tract infection in relation to vitamin D status.
- We did not identify longitudinal studies with multiple time-point, pre-admission or pre-disease vitamin D measurements.
- Most studies were single centre with heterogeneous patient groups and few controlled for important confounders that influence vitamin D levels such as age, BMI, gender, season of measurements, vitamin D supplementation and comorbidities.



For peer review only - http://bmjopen.bmj.com/site/about/guidelines.xhtml

For peer review only

## INTRODUCTION

Vitamin D is an essential nutrient[1, 2] representing a group of fat soluble secosteroids with key endocrine functions.[3] It is synthesized in the skin upon sunlight exposure[4] while dietary sources, such as oily fish, egg yolk, certain fungi and supplements, are usually secondary sources. Vitamin D is critical in bone metabolism[5] and calcium homeostasis,[6] as well as acting as an important regulator in extra-skeletal metabolic processes,[7] cardiovascular and immune systems.[8] Many observational and laboratory studies have observed the anti-inflammatory properties of vitamin D,[9] including direct regulation of endogenous anti-microbial peptide production.[10]

It is therefore crucial for humans to have sufficient vitamin D levels to maintain bone health and possibly improve response to infection.[6, 11, 12] Infants and children are especially dependent on vitamin D to achieve healthy bone development and growth.[13, 14] Well-known functional outcomes of adequate vitamin D levels in children include rickets prevention, higher bone mineral content and reduced bone fracture rates.[5, 14] In otherwise healthy children in the United States, the reported prevalence of vitamin D deficiency (25OHD levels of < 25 nmol/L) ranges from 9 to 18%.[15] The Endocrine Society Clinical Practice Guidelines and the Institute of Medicine (IOM) suggest that vitamin D levels less than 50 nmol/L (20 ng/mL) reflect a deficient state.[4, 16]

Studies in adults reflect a high prevalence of vitamin D deficiency both in general intensive care unit (ICU) and sepsis patients and strongly suggest an association between low vitamin D and poor clinical outcomes, including increased mortality, particularly in those suffering from sepsis.[2, 17] Recent clinical trials of vitamin D supplementation in adults appear promising in both general critical care[18, 19] and sepsis.[20]



For peer review only - http://bmjopen.bmj.com/site/about/guidelines.xhtml

Sepsis remains a challenging clinical entity with high social and economic costs.[21] Each year there are approximately 123,000 sepsis cases and around 37,000 deaths in England alone.[22] Recent reports show an increased prevalence of paediatric sepsis,[23] likely a reflection of an increased population with chronic comorbidities, higher rates of opportunistic infections and multidrug resistant organisms.[24] Respiratory tract infections account for a large proportion of underlying diagnoses in acute and critical care conditions[24, 25] but remain understudied.[26]

The magnitude and relevance of vitamin D deficiency in children receiving acute care is not clear. Several recent studies have addressed these questions with mixed results. We sought to summarise the evidence regarding the implications of vitamin D deficiency and its prevalence in general ICU, respiratory tract infection and sepsis patients in the paediatric population. We carried out a systematic review and meta-analysis of circulating vitamin D levels to assess the prevalence of vitamin D deficiency (≤ 50 nmol/L) and its association with mortality in these conditions.

For peer review only

## METHODS

We planned and conducted our systematic review and meta-analysis according to the PRISMA guidelines[27] (*Additional Table 1*) and since we did not include randomized controlled trials we reported following the Meta-Analysis of Observational Studies in Epidemiology (MOOSE) guidelines.[28]

### Search strategy and selection criteria

Our population of interest consists of paediatric patients with acute conditions and/or treated in ICU or emergency units for acute conditions whose vitamin D status was assessed prior to or

during admission. We included published cross sectional, case-control and cohort studies that measured circulating 25(OH) D levels and either reported prevalence, odds ratios (OR) or data to enable calculation. Studies were excluded if they were reviews, case reports, surveys, commentaries, replies, not original contributions, experimental *in vitro* or if they recruited patients who were not treated in emergency, neonatal intensive care units (NICUs), paediatric intensive care units (PICUs) or for acute conditions. Studies were also excluded if they only enrolled vitamin D deficient patients, investigated healthy populations only or did not measure circulating 25(OH) D levels as an indicator of vitamin D status. When we identified more than one publication utilising the same cohort, we included the publication which shared our review's objective to investigate vitamin D levels and prevalence of deficiency.

For purposes of our review, we classified vitamin D deficiency as being less than 50 nmol/L (equivalent to 20 ng/mL) as suggested by the IOM.[16] Different age categories were used to designate patients as "children" in the studies reviewed. We therefore included all "children" as defined by each treating facility and this included "neonates", "infants", "toddlers", "children" and "adolescents".

We searched PubMed, OVID, Google Scholar and the Cochrane Library from inception up until 5$^{th}$ November 2017, with no language restrictions. Search terms used across these databases included: "critical care", "vitamin D", "pediatric", "child", "neonate", "toddler", "intensive care unit", "sepsis" and "septic shock". Search terms used in OVID and PubMed are listed in the *Additional Tables 2A and 2B*. Literature searches were performed by two investigators independently and included initial screening of titles and abstracts, followed by full text screening. Any disagreements for study eligibility were resolved by discussion between the authors. Reference lists of the selected papers, including reviews, were also checked for relevant titles.



For peer review only - http://bmjopen.bmj.com/site/about/guidelines.xhtml

Abstracts of relevant titles were then assessed for eligibility. A data extraction form was designed a priori.

**Study quality assessment**

The quality of each included study was assessed using the Newcastle-Ottawa Scale (NOS) for cohort, case-control and cross-sectional study designs (*Additional Tables 3A, 3B* and *3C*).[29] We classified studies as low (1-3), medium (4-6) or high quality (7-9) for purposes of sensitivity analysis.

**Prevalence and mortality outcomes**

In the majority of studies (n = 36), prevalence of vitamin D deficiency was extracted as reported with a threshold of ≤ 50 nmol/L. If prevalence was not reported directly, it was calculated using data provided in each study (cases ≤ 50 nmol/L / total number of study participants, (*Additional Table 4A*and *4B*). Extracted or calculated prevalence values were then combined in a meta-analysis. For mortality, we calculated unadjusted odd ratios (OR) as:

OR = (vitamin D deficient patients who died * vitamin D non-deficient patients who did not die)/ (vitamin D deficient patients who did not die * vitamin D non-deficient patients who died)

We had sufficient information to calculate ORs < 50 nmol/L for 36 studies (75%). For the 12 studies with insufficient information, we used the lower cut-off values reported as a conservative approximation (*Additional Table 5*). We converted 25(OH) D values using: nmol/L = ng/mL * 2.496.

**Data analysis**



For peer review only - http://bmjopen.bmj.com/site/about/guidelines.xhtml

We obtained proportions of vitamin D deficiency with 95% confidence intervals (CI) using the Clopper-Pearson method[30] in R. We used a random effects model[31] to account for the variation observed within and between studies due to the different ages and acute conditions in the populations considered. For mortality, we also obtained pooled proportions and pooled ORs with fixed effect model for sensitivity analysis and to avoid false conclusions that could result from small-study effects.[32]

We investigated possible sources of heterogeneity using sensitivity and subgroup analyses. The $I^2$ statistic was used to estimate the percentage of total variation across studies which can be attributed to heterogeneity. A Q value of $< 0.05$ was considered significant and an $I^2$ statistic greater or equal to 75% indicated a high level of variation due to heterogeneity.[33, 34] We used Egger's regression test to present results for publication bias and funnel plot asymmetry[35] and generated funnel plots for visual assessment and screen for evidence of publication bias.

To further assess heterogeneity, we utilised meta-regression to identify predictor variables that could explain variation in study prevalence estimates. We used restricted maximum likelihood (REML) estimations in the model to account for residual heterogeneity[36] and the Knapp-Hartung method to adjust confidence intervals and test statistics. This method estimates between study variance using a t-distribution, rather than a z-distribution, yielding a more conservative inference.[37] We tested the following continuous predictors: year of study publication, total sample size and quality score. Categorical variables included study setting (PICU, NICU), study design (case-control, cross-sectional and cohort) and country group by geographic region and economic development (group 1, group 2, and group 3) and were dummy coded.



For peer review only - http://bmjopen.bmj.com/site/about/guidelines.xhtml

We used R version 3.5.0 and Microsoft Excel 2010 for analyses and data collection. The R packages "meta"[38] and "metafor"[39] were used for analyses. Only results of the random effects model are reported for prevalence due to the expected heterogeneity between populations being considered. Our protocol is registered in PROSPERO (CRD42016050638).

**Role of the funding source**

The study received funding from the UK Medical Research Council. The funders had no role in data collection, analysis, interpretation or writing of the report. All authors had access to the data in the study.

## RESULTS

**Screening and study characteristics**

After title and abstract screening, we identified 2,890 potentially relevant studies (*Figure 1*) and eighty-five full text articles were assessed for eligibility. Rationale for study exclusion included: studies including adults, study populations other than critically ill children or with acute conditions, studies of circulating vitamin D levels and deficiency in healthy children or in children with chronic conditions. Four studies[40-43] were excluded due to insufficient data reporting (*Additional Table 6*). We also excluded three studies[44-46] that used the same cohort of children and included a single study to represent the cohort.[47] Ultimately, 48 studies met criteria for inclusion (*Additional Table 7*).

*Figure 1 Flow chart of study selection process*



For peer review only - http://bmjopen.bmj.com/site/about/guidelines.xhtml

The primary objective of most studies was to determine circulating vitamin D concentration ("status") in children and/or prevalence of vitamin D deficiency. Secondary objectives included investigation of associations between deficiency of circulating vitamin D and various outcomes, such as hospital mortality length of stay, requirement of ventilation and/or illness severity (*Additional Table 8*).

All included studies reported vitamin D measurement assay methods used (*Additional Table 9*) and stated that samples were collected and analysed within the first 24 hours of hospital admission. Studies reported ethical approval and consent for participation from parents or guardians (*Additional Table 10*). Included studies were published between 2004 and 2017, with the majority (n = 39, 81.3%) published between 2014 and 2017 (*Additional Table 7*). In total, 5,520 children were hospitalized in paediatric or neonatal intensive care units or emergency units. Sample sizes of critically ill children ranged from 25[48] to 511.[49] In 16 studies the total number of cases was greater than 100.

Studies originated from 15 countries, with the majority from India[8, 50-58] (n = 10) or Turkey[48, 59-64] (n = 7) (*Additional Table 7*). All were of medium or high quality (NOS score median 6·5, range 4-8). The score range for cohort studies was 6 to 8 (n = 20), for case-control studies 5 to 8 (n = 24) and for cross sectional 4 to 6 (n = 4). Studies used a broad range of ages to classify patients as "children". Six studies (12.5%)[48, 60, 62-65] included only neonates. In two[60, 65] of these six studies, neonates were preterm. The largest age range was seen in the study of Ayulo et al 2014, which included individuals between 1 and 21 years of age (*Additional Table 11*).

All studies included both female and male participants. For mortality, four of the 18 studies (22%) carried out multivariate regression analysis with adjustment for confounders. The remaining



For peer review only - http://bmjopen.bmj.com/site/about/guidelines.xhtml

studies presented results using a variety of methods, including Spearman's correlation analysis, chi-square or Fisher's exact tests or descriptive statistics.

**Prevalence of vitamin D deficiency**

We included 48 studies representing a total of 5,220 critically ill children. Of these, 2,664 (48%) were classified as vitamin D deficient (< 50 nmol/L). Prevalence of deficiency ranged from 5%[66] to 95%[54] (*Additional Table 12*). Sample sizes ranged from 25 to 511, with a median of 82 individuals (*Additional Table 13*). Using a random effects model, the pooled prevalence estimate of vitamin D deficiency was 54.9% (95% CI 48.0-61.6) with a high proportion of variation attributed to heterogeneity ($I^2 = 95.0\%$, 95% CI 94.0-95.8, $p < 0.0001$) (*Figure 2*) and evidence of funnel plot asymmetry (p = 0.015, Egger's test) (*Table 1* and *Additional Figure 1*).

**Table 1 Pooled estimates of vitamin D deficiency in critically ill children and critically ill children with sepsis and those with respiratory tract infections**

| Patient category | Number of studies (Total number of individuals; number of deficient individuals) | Pooled proportion % (95% CI) | | Heterogeneity ($I^2$) % (95% CI) | Q value, d.f. p-value | Eggers p-value |
|---|---|---|---|---|---|---|
| | | Random effects | Fixed effects | | | |
| Critically ill children (includes those with sepsis) | 48 (5,520; 2,664) | 54.9 (48.0-61.6) | 46.8 (45.4-48.3) | 95.0 (94.0-95.8) | 931.46, 57, < 0.0001 | 0.015 |
| Critically ill children (only those with sepsis) | 16 (788; 499) | 63.8 (49.9-75.7) | 62.6 (58.6-66.5) | 90.5 (86.2-93.5) | 157.99, 15, < 0.0001 | 0.828 |
| Only those admitted with respiratory tract infections | 24 (1,683; 778) | 49.9 (37.6-62.2) | 43.2 (40.4-46.1) | 93.9 (92.1-95.3) | 378.7, 23, < 0.0001 | 0.217 |

CI = confidence intervals; $I^2$ = heterogeneity; df = degrees of freedom. Vitamin D deficiency defined in our study as < 50 nmol/L (20 ng/mL). $I^2$ statistic used to estimate heterogeneity between pooled studies: $I^2 \geq 75\%$ was considered high heterogeneity.



For peer review only - http://bmjopen.bmj.com/site/about/guidelines.xhtml

*Figure 2 Pooled prevalence estimate for vitamin D deficiency in critically ill children by study design.*

**Sensitivity analysis for prevalence**

We did not detect material differences in prevalence after exclusion of the 12 studies which did not directly report prevalence < 50 nmol/L (53.1%, 95% CI 45.6-60.4; $I^2$ = 95.1%, 95% CI 93.9-96.0, p < 0.0001) (*Additional Table 13*).

When examining results by sample size (defining "large" as ≥ 100 and "small" as < 100), we found that the 16[8, 47, 49-51, 59, 60, 67-75] studies with larger sample size included 3,561 total individuals and gave a prevalence estimate of 50.8% (95% CI 40.5-61.1; $I^2$ = 96.9%, 95% CI 95.9-97.6, p < 0.0001). The remaining 32 studies with "smaller" sample sizes included 1,959 total children and estimated pooled prevalence as 57.2% (95% 47.3-66.7; $I^2$= 92.7, 95% CI 90.7-94.3, p < 0.0001) (*Additional Table 14*).

We also conducted analysis by study design. Cohort studies (n = 20) yielded a prevalence estimate of 48.4% (95% CI 39.7-57.3; $I^2$ = 95.5%, 95% CI 94.1-96.5, p < 0.0001). In case-control studies (n = 24) the estimate was 64.1% (95% CI 53.2-73.6; $I^2$ = 92.8%, 95% CI 90.5-94.6, p < 0.0001) and in cross-sectional (n = 4) 34.8% (95% CI 12.8-66.0; $I^2$ = 96.7%, 95% CI 94.0-98.2, p <0.0001) (*Additional Table 14*, *Figure 2*).

We assessed whether studies' country of origin influenced results. Studies in India gave an estimate of 69.5% (95% CI 53.0-81.5; $I^2$ = 93.6%, 95% CI 90.2-95.8, p < 0.0001). Similarly, we found higher pooled prevalence estimates for studies from Turkey (76.3%, 95% CI 60.9-87.0; $I^2$ = 91.1%, 95% CI 84.2-95.0, p < 0.0001). We also grouped studies by geography and economic



For peer review only - http://bmjopen.bmj.com/site/about/guidelines.xhtml

development. Group 1: USA, Chile, Australia, Canada, Ireland, Japan, Spain; group 2: South Africa, China, Egypt, Iran, Turkey, Saudi Arabia; and group 3: Bangladesh, Thailand, and India. Prevalence was 36.1% (95% CI 27.8-45.4) for group 1 (n = 18), 62.7% (95% CI 52.2-72.2) for group 2 (n = 18) and 71.4% (95% CI 57.9-82.0) for group 3 (n = 12) *(Additional Figure 2)*. Variation attributable to heterogeneity was still high in the three subgroups ($I^2 > 90\%$).

Given the broad age range in included studies, we combined studies with only neonates[48, 60, 62-65] and observed a prevalence estimate of 85.6% (95% CI 78.5-90.6) with moderate variation attributable to heterogeneity ($I^2 = 54.3\%$, 95% CI 0.0-81.7, p value = 0.05). In all other studies (n = 42) that included children of broad age ranges, estimated prevalence was lower at 49.7% (95% CI 42.9-56.5; $I^2 = 94.7\%$, 95% CI 93.6-95.6, p value < 0.0001) (*Additional Table 14, Additional Figure 3*).

**Post-hoc investigation to determine sources of heterogeneity**

To investigate the substantial heterogeneity observed in prevalence estimates, we incorporated study-specific characteristics (year of publication, total study sample size, quality score, study design, country group and clinical setting) as covariates in a random effects meta-regression model. We identified clinical setting and country groups as significant predictors *(Figure 3)*. We found that the model fitted with all available covariates can explain 37.52% of $I^2$ with F = 5.1119, p = 0.0005 *(Additional Table 15)*. We also conducted univariate meta-regressions for each of the six predictors *(Additional Figure 4)*.

*Figure 3 Bubble plots of univariate meta-regressions.*



For peer review only - http://bmjopen.bmj.com/site/about/guidelines.xhtml

**Prevalence of vitamin D deficiency in critically ill children with sepsis and in those with respiratory tract infections**

A total of 788 (median 42, range 9 -160) patients had a diagnosis of sepsis, of which 499 (63.3%) were vitamin D deficient. Nine of the sixteen studies including septic patients were cohort (56.3%) and seven (43.8%) case-control *(Additional Table 16).* Most studies originated from India (n = 6) Turkey (n = 3) or Ireland (n = 2) and 15 were published between 2014 and 2017. Thirteen studies took place in a PICU and the remaining[60, 63, 65] in NICUs. We found that all studies were of medium to high quality (median NOS score 6.5, range 5 – 8). Pooled prevalence of vitamin D deficiency was 63.8% (95% CI 49.9-75.7) *(Figure 4)*. Variation attributable to heterogeneity was high ($I^2$ = 90.5%, 95% CI 86.2-93.5%, p < 0.0001). Funnel plot was symmetric suggesting no publication bias (p = 0.828, Egger's test) *(Additional Figure 5).*

*Figure 4 Pooled prevalence estimate for vitamin D deficiency in critically ill children with sepsis.*

We also analysed studies of patients admitted for respiratory tract infections (n = 24) such as acute lower respiratory tract infection (ALRTI), pneumonia and bronchiolitis. Of these 1,683 total individuals (median 49), 778 (46.2%) were vitamin D deficient. These studies were of high to medium quality (median NOS score 6.5, range 6 - 8). Most originated from India (n = 6) and Spain (n = 4). We found a prevalence estimate of 49.9% (95% CI 37.6-62.2; $I^2$ = 93.9%, 95% CI 92.1-95.3, p < 0.0001), with no evidence of publication bias (p = 0.217, Egger's test) (*Table 1)*. Two of these studies[50, 76] also investigated sepsis.

**Sensitivity analysis for prevalence in children with sepsis**



For peer review only - http://bmjopen.bmj.com/site/about/guidelines.xhtml

Exclusion of the studies[58, 60, 65, 77] utilising thresholds other than < 50 nmol/L for deficiency yielded a similar estimate of prevalence at 61.4% (95% CI 43.5-76.6; $I^2$ = 91.2% 86.5-94.2, p < 0.0001) *(Additional Table 17).*

We examined pooled prevalence estimates according to sample size (< 40 versus ≥ 40). Studies with a small sample size (n = 7; 123 total individuals) showed a prevalence estimate of 63.2% (95% CI 45.0-78.2) with moderate variation attributable to heterogeneity ($I^2$ = 66.2%, 95% CI 24.5-84.9, p = 0.0068). For the remaining nine studies (sample sizes ≥ 40, 665 total individuals) the estimate was 63.9% (95% CI 44.9 - 79.4) with high variation attributable to heterogeneity ($I^2$ = 94.3%, 95% CI 91.2-96.3, p < 0.0001).

There was no material change in prevalence estimates when analysed according to study design. The nine cohort studies (463 total individuals) gave an estimate of 62.6% (95% CI 40.7-80.4) with high variation attributable to heterogeneity ($I^2$ = 92.8%, 95% CI 88.6-95.5, p < 0.0001). Case-control studies (n = 7; 325 total individuals) showed a prevalence of 65.2% (95% CI 47.3-79.7; $I^2$ = 87.0%, 95% CI 75.5-93.1, p < 0.0001) *(Additional Table 17, Additional Figure 6).*

Studies from India (n = 6) gave a prevalence estimate of 66.4% (95% CI 48.3-80.7; $I^2$ = 83.6%, 95% CI 65.7-92.2, < 0.0001). The three studies from Turkey assessing septic patients gave a pooled estimate of 59.2% (95% CI 13.6-93.1; $I^2$ = 97.8%, 95% CI 95.8- 98.8, p < 0.0001) *(Additional Table 17).*

The prevalence estimate in the three studies[60, 63, 65] including neonates with sepsis was 76.9% (95% CI 61.9-87.3, $I^2$ = 74.7%, 95% CI 15.9-92.4, p-value 0.019). The thirteen studies with children of different ages, excluding neonates, gave a pooled estimate of 60.1% (95% CI 43.7-74.5; $I^2$ = 90.8%, 95% CI 86.1-93.9, p value < 0.0001) *(Additional Table 17).*



For peer review only - http://bmjopen.bmj.com/site/about/guidelines.xhtml

**Mortality in critically ill children**

We identified 18 studies[8, 47, 50-53, 55, 58, 59, 65, 68, 69, 71, 76-80] assessing vitamin D status and mortality. These studies included a total of 2,463 individuals, from which 220 deaths (17.2%) were observed in 1,278 (51.9%) individuals with vitamin D deficiency and 99 deaths (8.4%) were observed in 1,185 individuals without deficiency (48.1%).

All 18 studies took place in a PICU apart from one[65] in a NICU. Sixteen of these studies (89%) were published between 2014 and 2017. Fourteen were cohort (77.8%) and four case-controls (22.2%). Almost half (n = 7) of the studies originated from India. Quality scores ranged from 5 to 8 with a median of 6.

Using a random effects model, we found that vitamin D deficiency in critically ill children significantly increased the risk of death (OR 1.81, 95% CI 1.24-2.64, p-value = 0.002) with low, non-significant heterogeneity ($I^2$ = 25.7%, 95% CI 0.0-58.0, p = 0.153) (*Table 1, Figure 5*). We did not identify evidence of publication bias (p = 0.084, Egger's test) (*Additional Figure 7*).

*Figure 5 Pooled odds ratio (OR) of risk of mortality in vitamin D deficient versus vitamin D non-deficient critically ill children.*

**Sensitivity analysis for mortality in critically ill children**

We obtained similar results through the fixed effects model (OR 1.72, 95% CI 1.27-2.33, p = 0.0005) (*Table 1, Additional Figure 8*). When excluding studies with thresholds other than < 50 nmol/L indicating deficiency, we found the association between vitamin D deficiency and increased risk of mortality still significant but lower, both with the random (OR 1.59, 95% CI 1.05-



For peer review only - http://bmjopen.bmj.com/site/about/guidelines.xhtml

2.41, p = 0.028; $I^2$ = 24.3%, 95% CI 0.0-59.9, p = 0.191) and fixed effect models (OR 1.52, 95% CI 1.08-2.13, p = 0.016) with no indication of publication bias (p = 0.12, Egger's test) *(Additional Table 18).*

A significant association was also observed in analysis of the 14 cohort studies, both with the random (OR 1.80, 95% CI 1.15-2.81, p = 0.01) and fixed effects model (OR 1.65, 95% CI 1.17-2.34, p-value = 0.004) with low variation attributable to heterogeneity ($I^2$ = 31.3%, 95% CI 0.0-63.7). Pooling the four case-control studies together we obtained a significant positive association with the fixed (OR 1.97, 95% CI 1.02-3.82, p = 0.044) effects model but non-significant with the random effects model (OR 1.97, 95% CI 0.88-4.42, p = 0.098). The association was positive but not-significant when pooling the seven studies from India with the random effects model (OR 1.08, 95% CI 0.70-1.69, p-value = 0.710; $I^2$ = 0.0% 0.0-62.4, p = 0.589) and similar with fixed effects (OR 1.08, 95% CI 0.70-1.69, p = 0.710) *(Additional Table 18)*.

**Mortality in patients with sepsis and respiratory tract infections**

We were unable to identify a sufficient number of studies assessing vitamin D and mortality for meta-analysis in individuals with sepsis. Three studies[8, 58, 60] measured vitamin D levels in paediatric patients with sepsis. One study[8] assessed mortality and did not find a significant association in children from 1 to 12 years with sepsis (n=124). None of the studies with children admitted for respiratory tract infections looked at the association of vitamin D deficient versus vitamin D not deficient children with mortality.



For peer review only - http://bmjopen.bmj.com/site/about/guidelines.xhtml

## DISCUSSION

Vitamin D deficiency is highly prevalent worldwide, even in countries with abundant sunshine. Studies demonstrated high prevalence of vitamin D deficiency in otherwise healthy children from high-income countries (9 to 24%) but also from middle and low-income countries in Indian subcontinent (36 to 90%).[8]

We identified 48 studies representing a total of 5,520 children treated in ICU or emergency units for acute conditions who had blood vitamin D levels measured close to or upon admission. Our analysis shows that prevalence of vitamin D deficiency is high (range 5%[66] to 95%[54]) across ICU and emergency units in the paediatric population, particularly in individuals with sepsis. Importantly, our analysis showed a significantly increased risk of mortality in critically ill children with vitamin D deficiency. We carried out several analyses for sensitivity including fixed effects models, by study design, country group, age and sample size and found consistent results. A recently published meta-analysis[81] also investigated prevalence of vitamin D deficiency in critically ill children and its association with risk of mortality and showed similar results to ours.

Sub-group analyses in patients with sepsis or respiratory tract infections demonstrated a high prevalence of vitamin D deficiency, consistent with the increased risk of bacterial or nosocomial infection in vitamin D deficient individuals identified elsewhere.[81]

Although sepsis is a leading cause of paediatric mortality and morbidity worldwide,[82] we found few studies assessing the relationship between vitamin D status and mortality in this population. We were unable to identify sufficient studies including patients with sepsis to perform a meta-analysis of vitamin D status and mortality. Sepsis remains an area of unmet need with high social and financial costs.[24] Diagnostic criteria,[83] a lack of adequate biomarkers[84] and targeted treatment



For peer review only - http://bmjopen.bmj.com/site/about/guidelines.xhtml

remain important challenges in research on sepsis. We did not find studies that assessed the risk of mortality in relation to vitamin D deficiency in children admitted for respiratory tract infections either.

Strengths of our review include the large number of studies and large total sample size, allowing a high-powered investigation to identify meaningful associations. For our systematic review and meta-analysis, we followed pre-specified eligibility criteria and used the PRISMA[27] and MOOSE guidelines[28] for reporting. We carried out sensitivity analyses with few material differences in results. However, we note that the relationship between vitamin D deficiency and mortality was sensitive to study design and studies from India, probably due to the smaller number of individuals in those analyses. Only the prevalence analysis with neonates indicated lower variation attributable to heterogeneity ($I^2$ = 54.3%) along with a higher prevalence estimate (86%) compared to other analyses. As expected, heterogeneity across studies is high overall, particularly for prevalence estimates. We utilised meta-regression to investigate this substantial heterogeneity around prevalence estimates. From the six variables in our multi-variable model, only clinical setting and country groups were found to be significant predictors of pooled prevalence estimates of vitamin D deficiency and the full model could explain 37.52% of $I^2$. Studies in NICU yielded higher prevalence estimates compared to studies in PICU. Studies from group 3 countries were also associated with higher prevalence estimates compared to studies from countries of group 1 and 2. Other variables, mainly individual patient characteristics such as age and ethnicity, were not directly available to us and may account for significant heterogeneity. Future research should also investigate biological heterogeneity in order to strengthen the evidence and produce generalisable results.



For peer review only - http://bmjopen.bmj.com/site/about/guidelines.xhtml

Our systematic review did not identify longitudinal studies with multiple time-point, pre-disease or pre-admission vitamin D measurements. The majority of studies were single centre with heterogeneous patient groups and relatively small sample sizes. Few studies accounted for important confounders that influence vitamin D levels such as age, gender, BMI, season of measurements, vitamin D supplementation and comorbidities. The relationship observed between vitamin D deficiency and mortality could be due to reverse causation and future studies will need to control for these covariates and other confounders.

Although included studies were generally of good quality, sample sizes varied considerably and were typically small. Over half of studies included less than 100 cases and only 10 studies (19.6%) had a total sample size of more than 200 individuals. In addition, studies used a variety of definitions and age ranges to designate individuals as children. Our analysis only included mortality as a clinical outcome. A further general limitation is the difference in thresholds for vitamin D deficiency, particularly in the levels which are considered normal for infants and young children. Our assessment used the currently recommended threshold for deficiency ($\leq$ 50 nmol/L)[16] and used a conservative estimate for studies which used different criteria.

Vitamin D remains an attractive biomarker and potential therapeutic agent in acute and critical care patients. Carefully designed and adequately powered studies are needed to determine the importance and therapeutic value of vitamin D in the general and septic paediatric critical care population.

**Availability of data and materials**

Data and computational code used for processing and analysis are available at https://github.com/margarc/VitaminD_children



For peer review only - http://bmjopen.bmj.com/site/about/guidelines.xhtml

## Author contributions

AJBT conceived the study. AJBT and IT designed the study. MC collected data and performed the analysis with input from MAC, IT, ABJT and EE. MC and AJBT wrote the manuscript with contributions from all authors.

## Declaration of interests

The authors declare no conflicts of interest.

**Funding** Medical Research Council UK

## Acknowledgements

AJBT was supported by the Medical Research Council (UK MED-BIO Programme Fellowship, MR/L01632X/1).

**Ethics committee approval:** Not applicable.

## Main Figures

**Figure 1 Flow chart of study selection process**

**Figure 2 Pooled prevalence estimate for vitamin D deficiency in critically ill children by study design**. Forest plot shows results from the random effects model. Each diamond represents the pooled proportion of vitamin D deficiency for each of the subgroups (case-control, cohort, cross-sectional study designs). The diamond at the bottom represents the overall pooled proportion of all the 48 studies together. Each square shows the prevalence estimate of each study and the horizontal line across each square represents the 95% confidence interval (CI) of the prevalence estimate.

**Figure 3 Bubble plots of univariate meta-regressions. Each study is represented by a circle.** Predictor variables; A clinical setting and B country groups are shown on the x-axis and the effect measure logit transformed proportion shown on the vertical (y-axis). NICU = Neonatal Intensive Care Unit; PICU = Pediatric Intensive Care Unit; grp = country group; country group 1 = USA, Chile, Australia, Canada, Ireland, Japan, Spain; country group 2 = South Africa, China, Egypt, Iran, Turkey, Saudi Arabia; and country group 3 = Bangladesh, Thailand, and India



For peer review only - http://bmjopen.bmj.com/site/about/guidelines.xhtml

**Figure 4 Pooled prevalence estimate for vitamin D deficiency in critically ill children with sepsis.** Forest plot shows result from the random effects model. The diamond represents the overall pooled proportion of vitamin D deficiency from the meta-analysis of the 16 studies. Each square shows the prevalence estimate of each study and the horizontal line across each square represents the 95% confidence interval (CI) of the prevalence estimate.

**Figure 5 Pooled odds ratio (OR) of risk of mortality in vitamin D deficient versus vitamin D non-deficient critically ill children.** Forest plot shows result from the random effects model. Diamond represents the overall OR (with corresponding 95% Confidence Interval). Each square shows the odds ratio of each study and the horizontal line across each square represents the 95% confidence interval (CI) of the estimate.

## Supplementary Material

**Additional Tables**

Additional Table 1 PRISMA Checklist 2009

Additional Table 2A Search terms used in OVID

Additional Table 2B Search terms used in PubMed

Additional Table 3A Newcastle Ottawa study quality scoring system (cohort studies)

Additional Table 3B Newcastle Ottawa study quality scoring system (case-control studies)

Additional Table 3C Newcastle Ottawa study quality scoring system (cross sectional studies)

Additional Table 4A Circulating 25(OH) D threshold levels used in the selected studies

Additional Table 4B Circulating 25(OH) D threshold levels used in the selected studies for prevalence in sepsis

Additional Table 5 Studies with thresholds other than <50 nmol/L

Additional Table 6 Excluded studies

Additional Table 7 Characteristics of the 48 included studies

Additional Table 8 Objectives and outcomes of included studies

Additional Table 9 Assay used in each study to measure Vitamin D levels

Additional Table 10 Funding and ethical approval of included studies

Additional Table 11 Age groups of children in each study

Additional Table 12 Prevalence of vitamin D deficiency in each study of critically ill children (sorted from highest to lowest)

Additional Table 13 Characteristics of studies used in the meta-analysis for prevalence

Additional Table 14 Sensitivity analyses for prevalence of vitamin D deficiency in all critically ill children

Additional Table 15 Multivariate meta-regression model for prevalence



For peer review only - http://bmjopen.bmj.com/site/about/guidelines.xhtml

Additional Table 16 Characteristics of studies included in the meta-analysis for prevalence in individuals with sepsis

Additional Table 17 Sensitivity analyses for prevalence of vitamin D deficiency in critically ill children with sepsis

Additional Table 18 Sensitivity analyses for mortality

## Additional Figures

Additional Figure 1 Funnel plot of studies of prevalence of vitamin D deficiency in critically ill children

Additional Figure 2 Pooled prevalence estimate for vitamin D deficiency in critically ill children (subgroup analysis by country group)

Additional Figure 3 Pooled prevalence estimate for vitamin D deficiency in critically ill children (subgroup analysis of neonates versus all other age groups)

Additional Figure 4 Bubble plots of univariate meta-regressions.

Additional Figure 5 Funnel plot for prevalence of vitamin D deficiency in critically ill children with sepsis

Additional Figure 6 Pooled prevalence estimate for vitamin D deficiency in critically ill children with sepsis (subgroup analysis by study design)

Additional Figure 7 Funnel plot of risk of mortality in vitamin D deficient versus vitamin D non-deficient critically ill children

Additional Figure 8 Pooled odds ratio and 95% CI for risk of mortality in vitamin D deficient versus vitamin D non-deficient critically ill children (fixed effects model)

For peer review only

## References

1. Shuler FD, Wingate MK, Moore GH, Giangarra C. Sports health benefits of vitamin d. Sports health. 2012;4(6):496-501.
2. Verceles AC, Weiler B, Koldobskiy D, Goldberg AP, Netzer G, Sorkin JD. Association Between Vitamin D Status and Weaning From Prolonged Mechanical Ventilation in Survivors of Critical Illness. Respiratory care. 2015;60(7):1033-9.
3. Braegger C, Campoy C, Colomb V, Decsi T, Domellof M, Fewtrell M, et al. Vitamin D in the healthy European paediatric population. Journal of pediatric gastroenterology and nutrition. 2013;56(6):692-701.
4. Holick MF, Binkley NC, Bischoff-Ferrari HA, Gordon CM, Hanley DA, Heaney RP, et al. Evaluation, treatment, and prevention of vitamin D deficiency: an Endocrine Society clinical practice guideline. The Journal of clinical endocrinology and metabolism. 2011;96(7):1911-30.
5. Holick MF. Vitamin D deficiency. The New England journal of medicine. 2007;357(3):266-81.
6. Aranow C. Vitamin D and the immune system. Journal of investigative medicine : the official publication of the American Federation for Clinical Research. 2011;59(6):881-6.
7. Zhang YP, Wan YD, Sun TW, Kan QC, Wang LX. Association between vitamin D deficiency and mortality in critically ill adult patients: a meta-analysis of cohort studies. Critical care (London, England). 2014;18(6):684.
8. Ponnarmeni S, Kumar Angurana S, Singhi S, Bansal A, Dayal D, Kaur R, et al. Vitamin D deficiency in critically ill children with sepsis. Paediatrics and international child health. 2016;36(1):15-21.
9. Kulie T, Groff A, Redmer J, Hounshell J, Schrager S. Vitamin D: an evidence-based review. Journal of the American Board of Family Medicine : JABFM. 2009;22(6):698-706.
10. Hewison M. Antibacterial effects of vitamin D. Nature reviews Endocrinology. 2011;7(6):337-45.
11. Kempker JA, West KG, Kempker RR, Siwamogsatham O, Alvarez JA, Tangpricha V, et al. Vitamin D status and the risk for hospital-acquired infections in critically ill adults: a prospective cohort study. PloS one. 2015;10(4):e0122136.
12. Martineau AR, Jolliffe DA, Hooper RL, Greenberg L, Aloia JF, Bergman P, et al. Vitamin D supplementation to prevent acute respiratory tract infections: systematic review and meta-analysis of individual participant data. BMJ (Clinical research ed). 2017;356:i6583.
13. Absoud M, Cummins C, Lim MJ, Wassmer E, Shaw N. Prevalence and predictors of vitamin D insufficiency in children: a Great Britain population based study. PloS one. 2011;6(7):e22179.
14. Greer FR. Defining vitamin D deficiency in children: beyond 25-OH vitamin D serum concentrations. Pediatrics. 2009;124(5):1471-3.
15. Abou-Zahr R, Kandil SB. A pediatric critical care perspective on vitamin D. Pediatric research. 2015;77(1-2):164-7.
16. Ross AC, Manson JE, Abrams SA, Aloia JF, Brannon PM, Clinton SK, et al. The 2011 report on dietary reference intakes for calcium and vitamin D from the Institute of Medicine: what clinicians need to know. The Journal of clinical endocrinology and metabolism. 2011;96(1):53-8.
17. Ala-Kokko TI, Mutt SJ, Nisula S, Koskenkari J, Liisanantti J, Ohtonen P, et al. Vitamin D deficiency at admission is not associated with 90-day mortality in patients with severe sepsis or septic shock: Observational FINNAKI cohort study. Annals of medicine. 2016;48(1-2):67-75.
18. Putzu A, Belletti A, Cassina T, Clivio S, Monti G, Zangrillo A, et al. Vitamin D and outcomes in adult critically ill patients. A systematic review and meta-analysis of randomized trials. Journal of critical care. 2017;38:109-14.

For peer review only

19. Han JE, Jones JL, Tangpricha V, Brown MA, Brown LAS, Hao L, et al. High Dose Vitamin D Administration in Ventilated Intensive Care Unit Patients: A Pilot Double Blind Randomized Controlled Trial. Journal of clinical & translational endocrinology. 2016;4:59-65.
20. Quraishi SA, De Pascale G, Needleman JS, Nakazawa H, Kaneki M, Bajwa EK, et al. Effect of Cholecalciferol Supplementation on Vitamin D Status and Cathelicidin Levels in Sepsis: A Randomized, Placebo-Controlled Trial. Critical care medicine. 2015;43(9):1928-37.
21. Singer M, Deutschman CS, Seymour CW, Shankar-Hari M, Annane D, Bauer M, et al. The Third International Consensus Definitions for Sepsis and Septic Shock (Sepsis-3). Jama. 2016;315(8):801-10.
22. NHS-England. Improving outcomes for patients with sepsis: a cross-system action plan 2015 [Available from: https://www.england.nhs.uk/ourwork/part-rel/sepsis/.
23. Plunkett A, Tong J. Sepsis in children. BMJ (Clinical research ed). 2015;350:h3017.
24. Weiss SL, Fitzgerald JC, Pappachan J, Wheeler D, Jaramillo-Bustamante JC, Salloo A, et al. Global epidemiology of pediatric severe sepsis: the sepsis prevalence, outcomes, and therapies study. American journal of respiratory and critical care medicine. 2015;191(10):1147-57.
25. Mayr FB, Yende S, Angus DC. Epidemiology of severe sepsis. Virulence. 2014;5(1):4-11.
26. De Pascale G, Ranzani OT, Nseir S, Chastre J, Welte T, Antonelli M, et al. Intensive care unit patients with lower respiratory tract nosocomial infections: the ENIRRIs project. ERJ open research. 2017;3(4).
27. Moher D, Liberati A, Tetzlaff J, Altman DG. Preferred reporting items for systematic reviews and meta-analyses: the PRISMA statement. Journal of clinical epidemiology. 2009;62(10):1006-12.
28. Stroup DF, Berlin JA, Morton SC, Olkin I, Williamson GD, Rennie D, et al. Meta-analysis of observational studies in epidemiology: a proposal for reporting. Meta-analysis Of Observational Studies in Epidemiology (MOOSE) group. Jama. 2000;283(15):2008-12.
29. Wells GA SB OCD, Peterson J, Welch V, Losos M,Tugwell P. The Newcastle-Ottawa Scale (NOS) for assessing the quality of nonrandomised studies in meta-analyses [Available from: http://www.ohri.ca/programs/clinical_epidemiology/oxford.asp.
30. Newcombe RG. Two-sided confidence intervals for the single proportion: comparison of seven methods. Statistics in medicine. 1998;17(8):857-72.
31. DerSimonian R, Laird N. Meta-analysis in clinical trials revisited. Contemporary clinical trials. 2015;45(Pt A):139-45.
32. Borenstein M, Hedges LV, Higgins JP, Rothstein HR. A basic introduction to fixed-effect and random-effects models for meta-analysis. Research synthesis methods. 2010;1(2):97-111.
33. Higgins JP, Thompson SG, Deeks JJ, Altman DG. Measuring inconsistency in meta-analyses. BMJ (Clinical research ed). 2003;327(7414):557-60.
34. Higgins JP, Thompson SG. Quantifying heterogeneity in a meta-analysis. Statistics in medicine. 2002;21(11):1539-58.
35. Egger M, Davey Smith G, Schneider M, Minder C. Bias in meta-analysis detected by a simple, graphical test. BMJ (Clinical research ed). 1997;315(7109):629-34.
36. Panityakul T, Bumrungsup C, Knapp G. On estimating residual heterogeneity in random-effects meta-regression: a comparative study. J Stat Theory Appl. 2013;12(3):253.
37. Knapp G, Hartung J. Improved tests for a random effects meta-regression with a single covariate. Statistics in medicine. 2003;22(17):2693-710.
38. Schwarzer G. meta: An R package for meta-analysis. R news. 2007;7(3):40-5.
39. Viechtbauer W. Conducting meta-analyses in R with the metafor package. Journal of statistical software. 2010;36(3).
40. Garg D, Sharma VK, Karnawat B. Association of serum vitamin D with acute lower respiratory infection in Indian children under 5 years: a case control study. International Journal of Contemporary Pediatrics. 2016;3(4):1164-9.



For peer review only - http://bmjopen.bmj.com/site/about/guidelines.xhtml

41. Aydemir G, Cekmez F, Kalkan G, Fidanci MK, Kaya G, Karaoglu A, et al. High serum 25-hydroxyvitamin D levels are associated with pediatric sepsis. The Tohoku journal of experimental medicine. 2014;234(4):295-8.
42. Gamal TS, Madiha AS, Hanan MK, Abdel-Azeem ME, Marian GS. Neonatal and Maternal 25-OH Vitamin D Serum Levels in Neonates with Early-Onset Sepsis. Children (Basel, Switzerland). 2017;4(5).
43. Seliem MS, Abdel Haie OM, Mansour AI, Salama SSME. The relation between vitamin D level and increased risk for early-onset neonatal sepsis in full-term infants. Medical Research Journal. 2016;15(1):16-21.
44. McNally JD, Leis K, Matheson LA, Karuananyake C, Sankaran K, Rosenberg AM. Vitamin D deficiency in young children with severe acute lower respiratory infection. Pediatric pulmonology. 2009;44(10):981-8.
45. McNally JD, Doherty DR, Lawson ML, Al-Dirbashi OY, Chakraborty P, Ramsay T, et al. The relationship between vitamin D status and adrenal insufficiency in critically ill children. The Journal of clinical endocrinology and metabolism. 2013;98(5):E877-81.
46. McNally JD, Iliriani K, Pojsupap S, Sampson M, O'Hearn K, McIntyre L, et al. Rapid normalization of vitamin D levels: a meta-analysis. Pediatrics. 2015;135(1):e152-66.
47. McNally JD, Menon K, Chakraborty P, Fisher L, Williams KA, Al-Dirbashi OY, et al. The association of vitamin D status with pediatric critical illness. Pediatrics. 2012;130(3):429-36.
48. Karatekin G, Kaya A, Salihoglu O, Balci H, Nuhoglu A. Association of subclinical vitamin D deficiency in newborns with acute lower respiratory infection and their mothers. European journal of clinical nutrition. 2009;63(4):473-7.
49. Madden K, Feldman HA, Smith EM, Gordon CM, Keisling SM, Sullivan RM, et al. Vitamin D deficiency in critically ill children. Pediatrics. 2012;130(3):421-8.
50. Shah SK, Kabra SK, Gupta N, Pai G, Lodha R. Vitamin D Deficiency and Parathyroid Response in Critically-ill Children: Association with Illness Severity and Clinical Outcomes. Indian pediatrics. 2016;53(6):479-84.
51. Sankar J, Lotha W, Ismail J, Anubhuti C, Meena RS, Sankar MJ. Vitamin D deficiency and length of pediatric intensive care unit stay: a prospective observational study. Annals of intensive care. 2016;6(1):3.
52. Dayal D, Kumar S, Sachdeva N, Kumar R, Singh M, Singhi S. Fall in Vitamin D Levels during Hospitalization in Children. International journal of pediatrics. 2014;2014:291856.
53. Prasad S, Raj D, Warsi S, Chowdhary S. Vitamin D Deficiency and Critical Illness. Indian journal of pediatrics. 2015;82(11):991-5.
54. Wayse V, Yousafzai A, Mogale K, Filteau S. Association of subclinical vitamin D deficiency with severe acute lower respiratory infection in Indian children under 5 y. European journal of clinical nutrition. 2004;58(4):563-7.
55. Ebenezer K, Job V, Antonisamy B, Dawodu A, Manivachagan MN, Steinhoff M. Serum Vitamin D Status and Outcome among Critically Ill Children Admitted to the Pediatric Intensive Care Unit in South India. Indian journal of pediatrics. 2016;83(2):120-5.
56. Narang GS, Arora S, Kukreja S. Association of Vitamin D Deficiency with Acute Lower Respiratory Infection in Toddlers. Journal of Nepal Paediatric Society. 2016;36(1):14-6.
57. Jat KR, Kaur J, Guglani V. Vitamin D and Pneumonia in Children: A Case Control Study. J Pulm Med Respir Res. 2016;2(004).
58. Sankar J, Ismail J, Das R, Dev N, Chitkara A, Sankar MJ. Effect of Severe Vitamin D Deficiency at Admission on Shock Reversal in Children With Septic Shock. Journal of intensive care medicine. 2017:885066617699802.



For peer review only - http://bmjopen.bmj.com/site/about/guidelines.xhtml

59. Asilioglu N, Cigdem H, Paksu MS. Serum Vitamin D Status and Outcome in Critically Ill Children. Indian journal of critical care medicine : peer-reviewed, official publication of Indian Society of Critical Care Medicine. 2017;21(10):660-4.
60. Say B, Uras N, Sahin S, Degirmencioglu H, Oguz SS, Canpolat FE. Effects of cord blood vitamin D levels on the risk of neonatal sepsis in premature infants. Korean journal of pediatrics. 2017;60(8):248-53.
61. Cayir A, Turan MI, Ozkan O, Cayir Y. Vitamin D levels in children diagnosed with acute otitis media. JPMA The Journal of the Pakistan Medical Association. 2014;64(11):1274-7.
62. Cetinkaya M, Cekmez F, Buyukkale G, Erener-Ercan T, Demir F, Tunc T, et al. Lower vitamin D levels are associated with increased risk of early-onset neonatal sepsis in term infants. Journal of Perinatology. 2015;35(1):39.
63. Cizmeci MN, Kanburoglu MK, Akelma AZ, Ayyildiz A, Kutukoglu I, Malli DD, et al. Cord-blood 25-hydroxyvitamin D levels and risk of early-onset neonatal sepsis: a case-control study from a tertiary care center in Turkey. European journal of pediatrics. 2015;174(6):809-15.
64. Dinlen N, Zenciroglu A, Beken S, Dursun A, Dilli D, Okumus N. Association of vitamin D deficiency with acute lower respiratory tract infections in newborns. The journal of maternal-fetal & neonatal medicine : the official journal of the European Association of Perinatal Medicine, the Federation of Asia and Oceania Perinatal Societies, the International Society of Perinatal Obstet. 2016;29(6):928-32.
65. Onwuneme C, Martin F, McCarthy R, Carroll A, Segurado R, Murphy J, et al. The Association of Vitamin D Status with Acute Respiratory Morbidity in Preterm Infants. The Journal of pediatrics. 2015;166(5):1175-80.e1.
66. Roth DE, Jones AB, Prosser C, Robinson JL, Vohra S. Vitamin D status is not associated with the risk of hospitalization for acute bronchiolitis in early childhood. European journal of clinical nutrition. 2009;63(2):297-9.
67. Cebey-Lopez M, Pardo-Seco J, Gomez-Carballa A, Martinon-Torres N, Rivero-Calle I, Justicia A, et al. Role of Vitamin D in Hospitalized Children With Lower Tract Acute Respiratory Infections. Journal of pediatric gastroenterology and nutrition. 2016;62(3):479-85.
68. García-Soler P, Morales-Martínez A, Rosa-Camacho V, Lillo-Muñoz JA, Milano-Manso G. Vitamin D deficiency and morbimortality in critically ill paediatric patients. Anales de Pediatría (English Edition). 2017;87(2):95-103.
69. Rippel C, South M, Butt WW, Shekerdemian LS. Vitamin D status in critically ill children. Intensive care medicine. 2012;38(12):2055-62.
70. Alonso MA, Pallavicini ZF, Rodriguez J, Avello N, Martinez-Camblor P, Santos F. Can vitamin D status be assessed by serum 25OHD in children? Pediatric nephrology (Berlin, Germany). 2015;30(2):327-32.
71. Ayulo M, Jr., Katyal C, Agarwal C, Sweberg T, Rastogi D, Markowitz M, et al. The prevalence of vitamin D deficiency and its relationship with disease severity in an urban pediatric critical care unit. Endocrine regulations. 2014;48(2):69-76.
72. Rey C, Sanchez-Arango D, Lopez-Herce J, Martinez-Camblor P, Garcia-Hernandez I, Prieto B, et al. Vitamin D deficiency at pediatric intensive care admission. Jornal de pediatria. 2014;90(2):135-42.
73. Onwuneme C, Carroll A, Doherty D, Bruell H, Segurado R, Kilbane M, et al. Inadequate vitamin D levels are associated with culture positive sepsis and poor outcomes in paediatric intensive care. Acta paediatrica (Oslo, Norway : 1992). 2015;104(10):e433-8.
74. Jia K-P, Zhao L-F, Feng N, Ma K, Li Y-X. Lower level of vitamin D3 is associated with susceptibility to acute lower respiratory tract infection (ALRTI) and severity: a hospital based study in Chinese infants. INTERNATIONAL JOURNAL OF CLINICAL AND EXPERIMENTAL MEDICINE. 2017;10(5):7997-8003.
75. Halwany AS, Deghady AAE, Moustafa AAA, Diab AEM. Study of Serum Vitamin D Level among Critically Ill Patients Admitted to Alexandria University Pediatric Intensive Care Unit. 2017.



For peer review only - http://bmjopen.bmj.com/site/about/guidelines.xhtml

76. Bustos R, Rodriguez-Nunez I, Peña RZ, Soto GG. Vitamin D deficiency in children admitted to the paediatric intensive care unit. Revista chilena de pediatria. 2016;87(6):480-6.
77. El-Gamasy M, Eldeeb M, Abdelmageed M. Prognostic Value of Vitamin D Status in Pediatric Patients with Acute Kidney Injury in Tanta University Emergency Hospital, Egypt. J Emerg Intern Med. 2017;1(1):5.
78. Elmoneim A, Rhill R, Rahalli M, Al-Rahalli M, Alrehally A, Alrohily S. Vitamin D level in pediatric intensive care unit (PICU) patients: its relation to severity of illness. Pediat Therapeut. 2016;6(293):2161-0665.1000293.
79. Korwutthikulrangsri M, Mahachoklertwattana P, Lertbunrian R, Chailurkit LO, Poomthavorn P. Vitamin D deficiency and adrenal function in critically ill children. Journal of the Medical Association of Thailand = Chotmaihet thangphaet. 2015;98(4):365-72.
80. Badawi NES, Algebaly HAF, El Sayed R, Zeid ESA. Vitamin D deficiency in critically ill children. Kasr Al Ainy Medical Journal. 2017;23(1):6.
81. McNally JD, Nama N, O'Hearn K, Sampson M, Amrein K, Iliriani K, et al. Vitamin D deficiency in critically ill children: a systematic review and meta-analysis. Critical care (London, England). 2017;21(1):287.
82. Randolph AG, McCulloh RJ. Pediatric sepsis: important considerations for diagnosing and managing severe infections in infants, children, and adolescents. Virulence. 2014;5(1):179-89.
83. Vincent JL. The Clinical Challenge of Sepsis Identification and Monitoring. PLoS medicine. 2016;13(5):e1002022.
84. Mathias B, Mira JC, Larson SD. Pediatric sepsis. Current opinion in pediatrics. 2016;28(3):380-7.

For peer review only



For peer review only - http://bmjopen.bmj.com/site/about/guidelines.xhtml

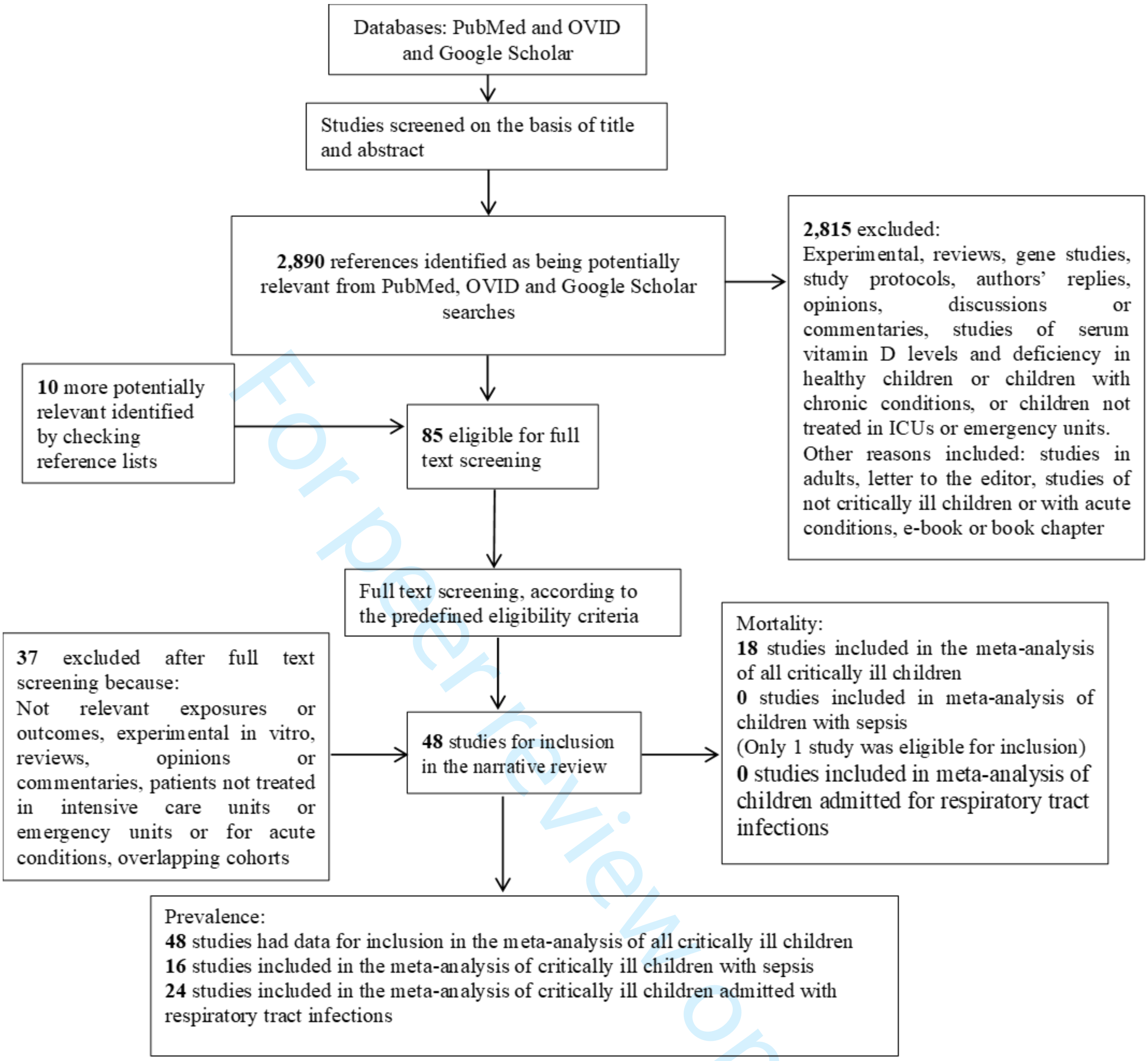


For peer review only

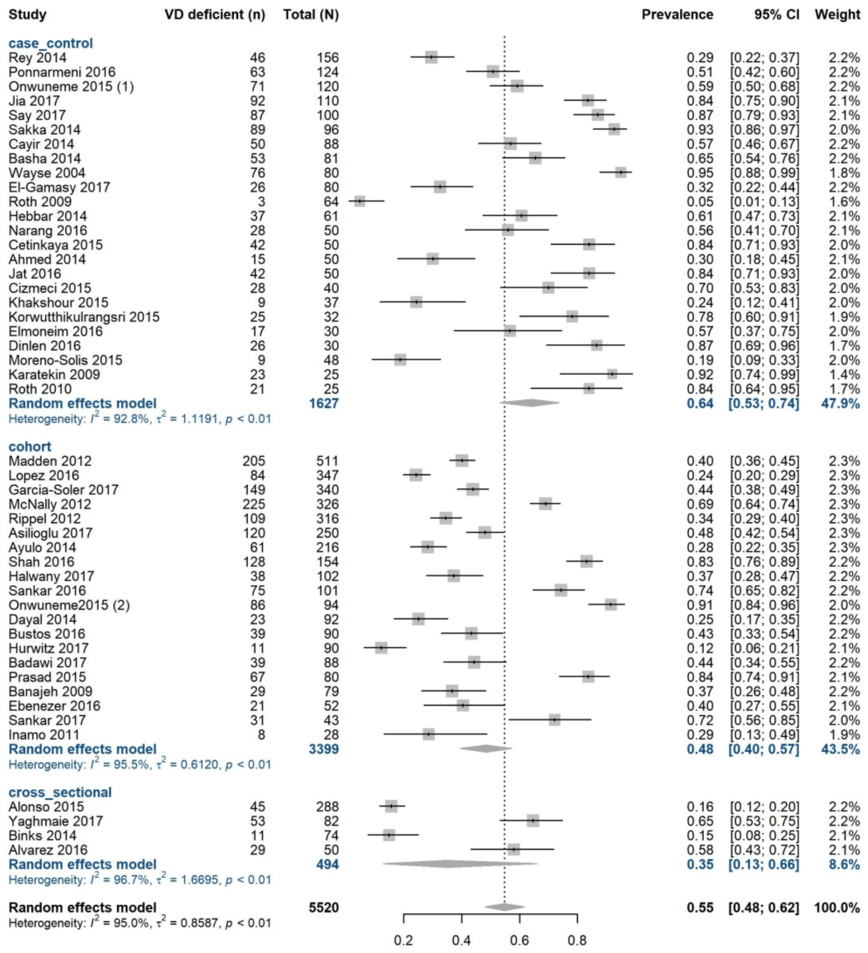


Figure 2 Pooled prevalence estimate for vitamin D deficiency in critically ill children by study design. Forest plot shows results from the random effects model. Each diamond represents the pooled proportion of vitamin D deficiency for each of the subgroups (case-control, cohort, cross-sectional study designs). The diamond at the bottom represents the overall pooled proportion of all the 48 studies together. Each square shows the prevalence estimate of each study and the horizontal line across each square represents the 95% confidence interval (CI) of the prevalence estimate.

203x254mm (300 x 300 DPI)

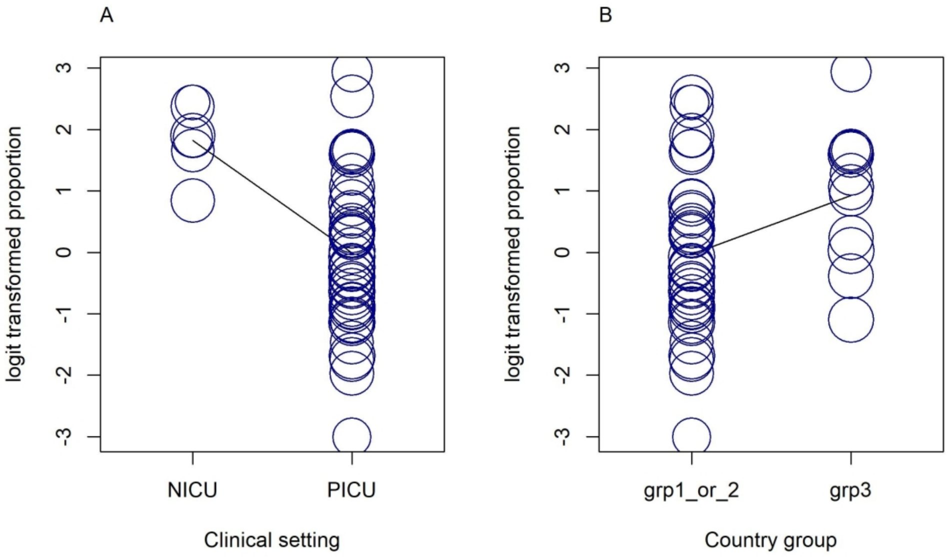


Figure 3 Bubble plots of univariate meta-regressions. Each study is represented by a circle. Predictor variables; A clinical setting and B country groups are shown on the x-axis and the effect measure logit transformed proportion shown on the vertical (y-axis). NICU = Neonatal Intensive Care Unit; PICU = Pediatric Intensive Care Unit; grp = country group; country group 1 = USA, Chile, Australia, Canada, Ireland, Japan, Spain; country group 2 = South Africa, China, Egypt, Iran, Turkey, Saudi Arabia; and country group 3 = Bangladesh, Thailand, and India

203x127mm (300 x 300 DPI)

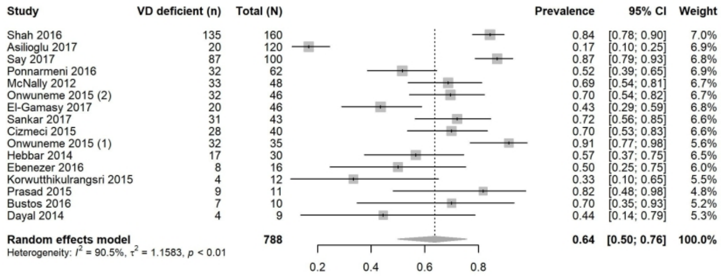


Figure 4 Pooled prevalence estimate for vitamin D deficiency in critically ill children with sepsis. Forest plot shows result from the random effects model. The diamond represents the overall pooled proportion of vitamin D deficiency from the meta-analysis of the 16 studies. Each square shows the prevalence estimate of each study and the horizontal line across each square represents the 95% confidence interval (CI) of the prevalence estimate.

254x152mm (300 x 300 DPI)

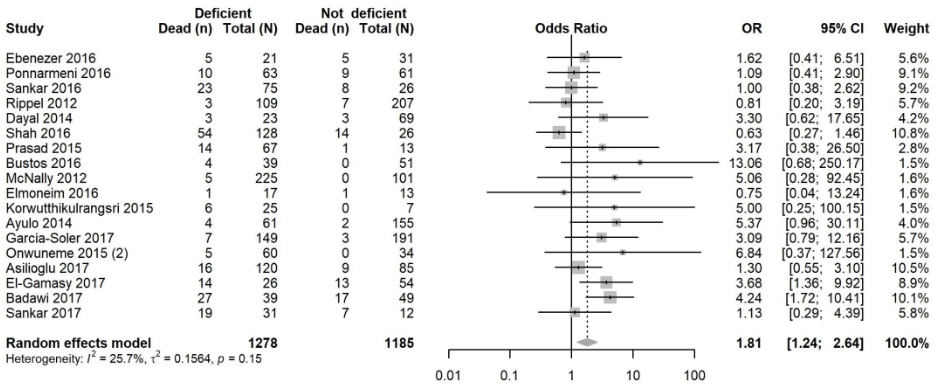


Figure 5 Pooled odds ratio (OR) of risk of mortality in vitamin D deficient versus vitamin D non-deficient critically ill children. Forest plot shows result from the random effects model. Diamond represents the overall OR (with corresponding 95% Confidence Interval). Each square shows the odds ratio of each study and the horizontal line across each square represents the 95% confidence interval (CI) of the estimate.

203x127mm (300 x 300 DPI)

# Supplementary Material

**Additional Table 1 PRISMA Checklist 2009**

| Section/topic | # | Checklist item | Reported on page # |
|---|---|---|---|
| **TITLE** | | | |
| Title | 1 | Identify the report as a systematic review, meta-analysis, or both. | 1 |
| **ABSTRACT** | | | |
| Structured summary | 2 | Provide a structured summary including, as applicable: background; objectives; data sources; study eligibility criteria, participants, and interventions; study appraisal and synthesis methods; results; limitations; conclusions and implications of key findings; systematic review registration number. | 2-3 |
| **INTRODUCTION** | | | |
| Rationale | 3 | Describe the rationale for the review in the context of what is already known. | 5-6 |
| Objectives | 4 | Provide an explicit statement of questions being addressed with reference to participants, interventions, comparisons, outcomes, and study design (PICOS). | 6-8 |
| **METHODS** | | | |
| Protocol and registration | 5 | Indicate if a review protocol exists, if and where it can be accessed (e.g., Web address), and, if available, provide registration information including registration number. | 10 |
| Eligibility criteria | 6 | Specify study characteristics (e.g., PICOS, length of follow-up) and report characteristics (e.g., years considered, language, publication status) used as criteria for eligibility, giving rationale. | 7 |
| Information sources | 7 | Describe all information sources (e.g., databases with dates of coverage, contact with study authors to identify additional studies) in the search and date last searched. | 7-9 |
| Search | 8 | Present full electronic search strategy for at least one database, including any limits used, such that it could be repeated. | 8 and supplementary material |
| Study selection | 9 | State the process for selecting studies (i.e., screening, eligibility, included in systematic review, and, if applicable, included in the meta-analysis). | 7-9 |
| Data collection process | 10 | Describe method of data extraction from reports (e.g., piloted forms, independently, in duplicate) and any processes for obtaining and confirming data from investigators. | 7 |
| Data items | 11 | List and define all variables for which data were sought (e.g., PICOS, funding sources) and any assumptions and simplifications made. | 7-8 |
| Risk of bias in individual studies | 12 | Describe methods used for assessing risk of bias of individual studies (including specification of whether this was done at the study or outcome level), and how this information is to be used in any data synthesis. | 8 |
| Summary measures | 13 | State the principal summary measures (e.g., risk ratio, difference in means). | 8 |
| Synthesis of results | 14 | Describe the methods of handling data and combining results of studies, if done, including measures of consistency (e.g., $I^2$) for each meta-analysis. | 8-10 |



For peer review only - http://bmjopen.bmj.com/site/about/guidelines.xhtml

| Additional Table 1 PRISMA Checklist 2009 | | | |
|---|---|---|---|
| **Section/topic** | **#** | **Checklist item** | **Reported on page #** |
| Risk of bias across studies | 15 | Specify any assessment of risk of bias that may affect the cumulative evidence (e.g., publication bias, selective reporting within studies). | 8 |
| Additional analyses | 16 | Describe methods of additional analyses (e.g., sensitivity or subgroup analyses, meta-regression), if done, indicating which were pre-specified. | 9-10 and 15 |
| **RESULTS** | | | |
| Study selection | 17 | Give numbers of studies screened, assessed for eligibility, and included in the review, with reasons for exclusions at each stage, ideally with a flow diagram. | 10 |
| Study characteristics | 18 | For each study, present characteristics for which data were extracted (e.g., study size, PICOS, follow-up period) and provide the citations. | 14-19 (supplementary material) |
| Risk of bias within studies | 19 | Present data on risk of bias of each study and, if available, any outcome level assessment (see item 12). | 5-7 (supplementary material) |
| Results of individual studies | 20 | For all outcomes considered (benefits or harms), present, for each study: (a) simple summary data for each intervention group (b) effect estimates and confidence intervals, ideally with a forest plot. | 13,15,18 and supplementary material |
| Synthesis of results | 21 | Present results of each meta-analysis done, including confidence intervals and measures of consistency. | 13 and supplementary material pages 32-36 |
| Risk of bias across studies | 22 | Present results of any assessment of risk of bias across studies (see Item 15). | 5-7 (supplementary material) |
| Additional analysis | 23 | Give results of additional analyses, if done (e.g., sensitivity or subgroup analyses, meta-regression [see Item 16]). | supplementary material pages 32-36 |
| **DISCUSSION** | | | |
| Summary of evidence | 24 | Summarize the main findings including the strength of evidence for each main outcome; consider their relevance to key groups (e.g., healthcare providers, users, and policy makers). | 19-21 |
| Limitations | 25 | Discuss limitations at study and outcome level (e.g., risk of bias), and at review-level (e.g., incomplete retrieval of identified research, reporting bias). | 20-22 |
| Conclusions | 26 | Provide a general interpretation of the results in the context of other evidence, and implications for future research. | 22 |
| **FUNDING** | | | |
| Funding | 27 | Describe sources of funding for the systematic review and other support (e.g., supply of data); role of funders for the systematic review. | 22 |

From: Moher D, Liberati A, Tetzlaff J, Altman DG, The PRISMA Group (2009). Preferred Reporting Items for Systematic Reviews and Meta-Analyses: The PRISMA Statement. PLoS Med 6(6): e1000097. doi:10.1371/journal.pmed1000097 For more information, visit: www.prisma-statement.org.



For peer review only - http://bmjopen.bmj.com/site/about/guidelines.xhtml

| Additional Table 2A Search terms used in OVID | |
|---|---|
| 1. | Vitamin D |
| 2. | Sepsis or septic shock |
| 3. | Vitamin D or cholecalciferol* or ergocalciferol or 25-hydroxyvitamin D or calcidiol or calcitriol |
| 4. | Intensive care unit* or critical care |
| 5. | |
| 6. | Multiple organ dysfunction syndrome or multiple organ failure |
| 7. | (1 and 2) or 5 |
| 8. | ((1 or 3) and 2 or 4 |
| 9. | (1 or 3) and 2 |
| 10. | (1 or 3) and 4 |
| 11. | (1 or 3) and 5 |
| 12. | Sepsis |
| 13. | multi* organ dysfunction syndrome or multiple organ failure |
| 14. | multi* organ dysfunction syndrome or multi* organ failure |
| 15. | (2 or 8) and 3 |
| 16. | critical* ill or acute condition* or intensive care unit |
| 17. | child* or pediatric* |
| 18. | vitamin D or cholecalciferol or ergocalciferol or 25-hydroxyvitamin D or calcidiol or calcitriol |
| 19. | 15 and 16 and 17 |
| 20. | Vitamin D blood levels or 25-hydroxyvitamin |
| 21. | 5 and 3 and 15 and 16 |
| 22. | 2 and 15 and 16 and 17 |
| 23. | 16 and 2 |
| 24. | 16 and 2 and 3 |
| 25. | Pediatric* |
| 26. | Pediatric* and 5 and 2 |
| 27. | 24 and 3 and 15 |



For peer review only - http://bmjopen.bmj.com/site/about/guidelines.xhtml

**Additional Table 2B Search terms used in PubMed**

| Term searched | |
|---|---|
| 1. | Vitamin D.mp. |
| 2. | (sepsis or septic shock).mp. |
| 3. | (vitamin D or cholecalciferol* or ergocalciferol or 25-hydroxyvitamin D or calcidiol or calcitriol).mp. |
| 4. | (intensive care unit* or critical care).mp. |
| 5. | (multi* organ dysfunction syndrome or multiple organ failure).mp. |
| 6. | (critical* ill or acute condition* or intensive care unit).mp. |
| 7. | (toddler or infant or child* or neonate* or baby or teenager or pediatric* or paediatric*).mp. |
| 8. | 3 and 6 and 2 and 7 |

For peer review only



For peer review only - http://bmjopen.bmj.com/site/about/guidelines.xhtml

**Additional Table 3A Newcastle Ottawa study quality scoring system (cohort studies)**

| Study | Selection | | | | Comparability | Outcome | | | Number of stars (out of 9 total) |
|---|---|---|---|---|---|---|---|---|---|
| | Representativeness of the exposed cohort | Selection of the non-exposed cohort | Ascertainment of exposure | Demonstration that outcome of interest was not present at the start of the study | Comparability of cohorts on the basis of the design or analysis (maximum 2 stars) | Assessment of outcome | Was follow-up long enough for outcome to occur (≥ 28 days after admission to the ICU) | Adequacy of follow up of cohorts | |
| Ebenezer 2016 | 1 | 1 | 1 | 1 | 0 | 1 | 0 | 1 | 6 |
| Sankar 2016 | 1 | 1 | 1 | 1 | 2 | 1 | 0 | 1 | 8 |
| Rippel 2012 | 1 | 1 | 1 | 1 | 1 | 1 | 1 | 1 | 8 |
| Madden 2012 | 1 | 1 | 1 | 1 | 2 | 1 | 0 | 1 | 8 |
| McNally 2012 | 1 | 1 | 1 | 0 | 1 | 1 | 1 | 1 | 7 |
| Dayal 2014 | 1 | 1 | 1 | 1 | 0 | 1 | 0 | 1 | 6 |
| Ayulo 2014 | 1 | 1 | 1 | 1 | 2 | 1 | 0 | 0 | 7 |
| Bustos 2016 | 1 | 1 | 1 | 0 | 2 | 1 | 0 | 1 | 7 |
| Prasad 2015 | 1 | 1 | 1 | 0 | 2 | 1 | 0 | 1 | 7 |
| Onwuneme 2015 (2) | 1 | 1 | 1 | 1 | 2 | 1 | 0 | 1 | 8 |
| Inamo 2011 | 1 | 1 | 1 | 0 | 1 | 1 | 0 | 1 | 6 |
| Shah 2016 | 1 | 1 | 1 | 1 | 0 | 1 | 0 | 1 | 6 |
| Lopez 2016 | 1 | 1 | 1 | 1 | 2 | 1 | 0 | 1 | 8 |
| Garcia-Soler 2017 | 1 | 1 | 1 | 1 | 2 | 1 | 0 | 0 | 7 |
| Sankar 2017 | 1 | 1 | 1 | 1 | 2 | 1 | 0 | 1 | 8 |
| Asilioglu 2017 | 1 | 1 | 1 | 1 | 1 | 1 | 0 | 1 | 7 |
| Halwany 2017 | 1 | 1 | 1 | 1 | 1 | 1 | 0 | 0 | 6 |
| Hurwitz 2017 | 1 | 1 | 1 | 0 | 1 | 1 | 0 | 1 | 6 |
| Banajeh 2009 | 1 | 1 | 1 | 0 | 1 | 1 | 1 | 1 | 7 |
| Badawi 2017 | 1 | 1 | 1 | 1 | 1 | 1 | 1 | 1 | 8 |

The Newcastle-Ottawa Scale (NOS) for assessing the quality of non-randomised studies in meta-analyses. Available at: http://www.ohri.ca/programs/clinical_epidemiology/oxford.asp.

For peer review only

**Additional Table 3B Newcastle Ottawa study quality scoring system (case-control studies)**

| Study | SELECTION | | | | COMPARABILITY | EXPOSURE | | | Number of stars (out of 9 total) |
|---|---|---|---|---|---|---|---|---|---|
| | Is the case definition adequate? | Representativeness of the cases | Selection of controls | Definition of controls | Comparability of cases and controls on the basis of the design or analysis (maximum 2 stars) | Ascertainment of exposure | Same method of ascertainment for cases and controls (yes or no or 0 star if no mention) | Non-response rate | |
| Wayse 2004 | 1 | 1 | 0 | 0 | 1 | 1 | 1 | 1 | 6 |
| Karatekin 2009 | 1 | 1 | 0 | 0 | 1 | 1 | 1 | 1 | 6 |
| Roth 2009 | 1 | 1 | 0 | 1 | 1 | 1 | 1 | 0 | 7 |
| Roth 2010 | 1 | 1 | 1 | 1 | 2 | 1 | 1 | 0 | 8 |
| Cetinkaya 2015 | 1 | 1 | 0 | 0 | 0 | 1 | 1 | 1 | 6 |
| Onwuneme 2015 (1) | 1 | 1 | 0 | 1 | 2 | 1 | 1 | 1 | 8 |
| Cizmeci 2015 | 1 | 1 | 0 | 0 | 2 | 1 | 1 | 1 | 7 |
| Korwutthikulrangsri 2015 | 1 | 1 | 0 | 0 | 0 | 1 | 1 | 1 | 5 |
| Elmoneim 2016 | 1 | 1 | 0 | 0 | 0 | 1 | 1 | 1 | 5 |
| Narang 2016 | 1 | 1 | 0 | 0 | 1 | 1 | 1 | 1 | 6 |
| Dinlen 2016 | 1 | 1 | 0 | 0 | 1 | 1 | 1 | 1 | 6 |
| Ahmed 2015 | 1 | 1 | 0 | 1 | 2 | 1 | 1 | 1 | 8 |
| Cayir 2014 | 1 | 1 | 0 | 0 | 2 | 1 | 1 | 1 | 7 |
| Say 2017 | 1 | 1 | 0 | 0 | 2 | 1 | 1 | 0 | 6 |
| Basha 2014 | 1 | 1 | 0 | 1 | 2 | 1 | 1 | 0 | 7 |
| Jia 2017 | 1 | 1 | 0 | 1 | 1 | 1 | 1 | 1 | 7 |
| Jat 2016 | 1 | 1 | 0 | 1 | 2 | 1 | 1 | 1 | 8 |
| Moreno-Solis 2015 | 1 | 1 | 0 | 1 | 1 | 1 | 0 | 1 | 6 |
| Sakka 2014 | 1 | 1 | 0 | 1 | 2 | 1 | 1 | 1 | 8 |
| Hebbar 2014 | 1 | 1 | 0 | 0 | 1 | 1 | 1 | 1 | 6 |
| Rey 2014 | 1 | 1 | 1 | 1 | 1 | 1 | 1 | 1 | 8 |
| Ponnarmeni 2016 | 1 | 1 | 0 | 0 | 0 | 1 | 1 | 1 | 6 |
| El-Gamasy 2017 | 1 | 1 | 1 | 0 | 2 | 1 | 1 | 0 | 7 |
| Khakshour 2015 | 1 | 1 | 0 | 0 | 1 | 1 | 1 | 0 | 5 |

The Newcastle-Ottawa Scale (NOS) for assessing the quality of non-randomised studies in meta-analyses. Available at: http://www.ohri.ca/programs/clinical_epidemiology/oxford.asp.



For peer review only - http://bmjopen.bmj.com/site/about/guidelines.xhtml

**Additional Table 3C Newcastle Ottawa study quality scoring system (cross-sectional studies)**

| | SELECTION (Maximum 5 stars) | | | | COMPARABILITY (Maximum 2 stars) | OUTCOME (Maximum 3 stars) | | |
|---|---|---|---|---|---|---|---|---|
| **Study** | **Representativeness of the sample** | **Sample size** | **Non-respondents** | **Ascertainment of the exposure (risk factor)** | **Subjects in different outcome groups are comparable, based on the study design or analysis. Confounding factors are controlled.** | **Assessment of the outcome (max=2 stars)** | **Statistical test (max=1 star)** | **Number of stars (out of 10 total)** |
| Yaghmaie 2017 | 1 | 0 | 0 | 0 | 1 | 1 | 1 | 4 |
| Alvarez 2016 | 1 | 0 | 0 | 1 | 1 | 2 | 1 | 6 |
| Alonso 2015 | 1 | 0 | 0 | 1 | 1 | 1 | 1 | 5 |
| Binks 2014 | 1 | 0 | 0 | 1 | 1 | 2 | 1 | 6 |

The Newcastle-Ottawa Scale (NOS) for assessing the quality of non-randomised studies in meta-analyses. Available at: http://www.ohri.ca/programs/clinical_epidemiology/oxford.asp.



For peer review only - http://bmjopen.bmj.com/site/about/guidelines.xhtml

**Additional Table 4A Circulating 25(OH)D threshold levels used in the selected studies**

| Study | Number of cases | 25(OH)D categories (as given) | Number of cases in each category |
|---|---|---|---|
| Asilioglu 2017 | 250 | Deficiency: <20 ng/mL | 120 |
| | | Sufficiency: >=20 ng/mL | 85 |
| Halwany 2017 | 102 | Deficiency: <20 ng/ml | 38 |
| | | Insufficiency: 20-29.9 ng/ml | 39 |
| | | "Normal" levels: 30 ng/ml | 25 |
| Hurwitz 2017 | 90 | Deficiency: <20 ng/mL | 11 |
| Garcia-Soler 2017 | 340 | Deficiency: <20 ng/mL | 149 |
| | | Insufficiency: 20-30 ng/mL | 128 |
| | | 25(OH)D levels: 30-40 ng/mL | 41 |
| | | Optimal levels >40 ng/mL | 22 |
| Badawi 2017 | 88 | Deficiency: < 50nmol/l | 39 |
| | | Severe deficiency: <30 nmol/l | 30 |
| Jia 2017 | 110 | Severe deficiency: <10 ng/ml | 36 |
| | | Deficiency: 10-20 ng/ml | 56 |
| | | Insufficiency: 21-30 ng/ml | 17 |
| | | Sufficiency: >30 ng/ml | 1 |
| Yaghmaie 2017 | 82 | Deficiency: <30 ng/ml | 53 |
| | | Sufficiency: >=30 ng/ml | 29 |
| Say 2017 | 100 | Severe deficiency (group 1) <5 ng/mL. | 63 |
| | | Insufficiency (group2): 5 to 15 ng/mL | 24 |
| | | Sufficiency (group 3) ≥15 ng/mL | 13 |
| El-Gamasy 2017 | 80 | Serious deficiency: < 30 nmol/L | 26 |
| | | Insufficiency: 30-75 nmol/L | 27 |
| | | Adequate levels >75 nmol/L | 27 |
| Sankar 2017 | 43 | Severe deficiency: serum 25 (OH) D <10 ng/mL | 31 |
| Shah 2016 | 154 | 25(OH)D < 20 ng/mL | 128 |
| | | 25(OH)D ≥20 ng/mL | 26 |
| Ponnarmeni 2016 | 124 | Deficiency: < 50nmol/L | 63 |
| | | Insufficiency: 50-75 nmol/L | 31 |
| | | <75, insufficient and deficient | 94 |
| | | Sufficiency: >75 nmol/L | 30 |
| Sankar 2016 | 101 | Deficiency: ≤ 20 ng/mL | 75 |
| | | Severe deficiency: <15 ng/mL | 62 |
| | | 'No deficiency': > 20 ng/mL | 26 |
| Bustos 2016 | 90 | Deficiency: <20 ng/ml | 39 |
| | | Normal levels: >20 ng/ml | 51 |
| Ebenezer 2016 | 52 | Deficiency: < 20ng/mL | 21 |
| | | Insufficiency: 20–30 ng/ml | 12 |
| | | 'Normal levels: ≥ 20 ng/L | 31 |
| Elmoneim 2016 | 21 | Deficiency: < 20 ng/mL | 17 |
| | | Insufficiency: 20-30 ng/mL | 4 |
| | | Normal levels: > 30 ng/mL | 9 |
| Jat 2016 | 50 | Deficiency: <20 ng/ml | 42 |
| | | Insufficiency: 20-30 ng/ml | 2 |
| | | Sufficiency: >=30 ng/ml | 1 |
| Narang 2016 | 50 | "Severe deficiency": <20ng/ml | 28 |
| Dinlen 2016 | 30 | Deficiency: <=15 ng/mL | 26 |
| | | Severe deficiency: <=5 ng/mL | 3 |
| Lopez 2016 | 347 | 25-OHD levels: <10 ng/mL | 19 |
| | | 25-OHD levels: 10-20 ng/mL | 65 |
| | | 25-OHD levels: 20-30 ng/mL | 134 |
| | | | |
| Alvarez 2016 | 50 | 25(OH) D levels: <20 ng/mL | 29 |
| | | 25(OH) D levels: 20-30 ng/mL | 37 |
| Onwuneme 2015 (1) | 120 | 25(OH)D levels: <50nmol/L | 71 |
| | | 25(OH)D levels: ≥50nmol/L | 49 |
| Onwuneme 2015 (2) | 94 | 25(OH)D levels: <20 ng/mL | 86 |
| | | 25(OH)D levels: < 30 nmol/L | 60 |
| | | 25(OH)D levels: >=30 nmol/L | 34 |
| Prasad 2015 | 80 | Deficiency: < 20 ng/ml | 67 |
| | | Sufficiency: ≥ 20 ng/mL | 13 |
| Moreno-Solis 2015 | 48 | Deficiency: <20 ng/ml | 9 |



For peer review only - http://bmjopen.bmj.com/site/about/guidelines.xhtml

| | | | |
|---|---|---|---|
| | | Insufficiency: 21–29 ng/ml | 16 |
| | | Sufficiency: ≥30 ng/ml | 23 |
| Alonso 2015 | 288 | Deficiency: <20 ng/ml | 45 |
| | | <10 ng/ml | 6 |
| Korwutthikulrangsri 2015 | 32 | Deficiency: < 20 ng/ml | 25 |
| | | Insufficiency: 20-29.9 ng/ml | 7 |
| | | Sufficiency: ≥ 30 ng/mL | 0 |
| Khakshour 2015 | 37 | Deficiency: <20 ng/mL | 9 |
| Cizmeci 2015 | 40 | Deficiency: ≤20 ng/ml | 28 |
| | | Insufficiency: 21–29 ng/ml | 7 |
| | | Normal levels: ≥30 ng/ml | 5 |
| Cetinkaya 2015 | 50 | Severe deficiency: <10 ng $ml^{-1}$ | 42 |
| | | Insufficiency: 11 to 32 ng $ml^{-1}$ | 8 |
| Ayulo 2014 | 216 | Deficient: < 15 ng/ml | 61 |
| | | Insufficient: 15-29 ng/mL | 102 |
| | | Sufficient: ≥ 30 ng/mL | 53 |
| Dayal 2014 | 92 | Deficiency: < 50 nmol/L | 23 |
| | | Insufficiency: 50–75 nmol/L | 41 |
| | | Sufficiency: > 75 nmol/L | 28 |
| | | 25(OH) D levels: < 75 nmol/L | 64 |
| | | 'Non-deficiency': > 50 nmol/L | 69 |
| Hebbar 2014 | 61 | Deficiency: ≤ 10 ng/mL | 10 |
| | | Insufficiency: 10 to 20 ng/mL | 27 |
| | | Sufficiency: ≥ 20 ng/mL | 24 |
| Rey 2014 | 156 | Deficiency: < 20 ng/mL | 46 |
| | | 25(OH)D levels: ≥ 20 ng/mL | 110 |
| Ahmed 2014 | 50 | Deficiency: <50 nmol/L | 15 |
| Basha 2014 | 81 | Deficiency: <50 nmol/L | 53 |
| | | Insufficiency: <75 nmol/L | 14 |
| | | Normal level: >75 nmol/L | 14 |
| Sakka 2014 | 96 | Severe deficiency: 0–5 ng/mL | 29 |
| | | Deficiency: 5–15 ng/mL | 49 |
| | | Insufficiency: 15– 20 ng/mL | 11 |
| | | Sufficiency: 20–100 ng/mL | 7 |
| Cayir 2014 | 88 | Normal levels: 20 ng/mL | 38 |
| | | Insufficiency: 15-20ng/mL | 18 |
| | | Deficiency: <15ng/mL | 32 |
| Binks 2014 | 74 | 25(OH) D levels: < 50 nmol/L | 11 |
| Madden 2012 | 511 | 25(OH) D levels: < 10 ng/mL | 36 |
| | | 25(OH) D levels: 10-19.9 ng/mL | 169 |
| | | Deficiency: < 20 ng/ml | 205 |
| | | Insufficiency: <30 ng/mL | 364 |
| Rippel 2012 | 316 | Deficiency: < 50 nmol/L | 109 |
| | | 'Normal levels': ≥50 nmol/L | 207 |
| Madden 2012 | 511 | 25(OH)D levels: < 10 ng/mL | 36 |
| | | 25(OH)D levels: 10-19.9 ng/mL | 169 |
| | | Deficiency: < 20 ng/mL | 205 |
| | | Insufficiency: <30 ng/mL | 364 |
| McNally 2012 | 326 | Deficiency: < 50 nmol/L | 225 |
| | | 25(OH)D levels: 50 to 75 nmol/L | 75 |
| | | 'Not deficient': > 50 nmol/L | 101 |
| Inamo 2011 | 28 | 25(OH)D levels: < 10 ng/mL | 4 |
| | | 25(OH)D levels: < 15 ng/mL | 8 |
| | | 25(OH)D levels: < 25 ng/mL | 12 |
| | | 25(OH)D levels: < 40 ng/mL | 28 |
| Roth 2010 | 25 | 25(OH)D levels: < 40 nmol/L | 21 |
| Banajeh 2009 | 79 | Deficiency: <30 nmol/L | 29 |
| Karatekin 2009 | 25 | Serum 25(OH)D <10 (deficiency) | 19 |
| | | Serum 25(OH)D 11 to 20 (deficiency) | 4 |
| | | Serum 25(OH)D 21 to 32 (insufficiency) | 1 |



For peer review only

For peer review only - http://bmjopen.bmj.com/site/about/guidelines.xhtml

| | | | |
|---|---|---|---|
| | | Serum 25(OH)D 32 to 100 (sufficiency) | 1 |
| Roth 2009 | 64 | < 40 nmol/L | 3 |
| Wayse 2004 | 80 | Plasma 25(OH)D3 > 22.5 nmol/L | 26 |
| | | Plasma 25(OH)D3 > 50 nmol/L | 4 |

25(OH)D in nmol/L = 25(OH)D in ng/mL multiplied by 2.496

For peer review only

**Additional Table 4B Circulating 25(OH)D threshold levels used in the selected studies for prevalence in sepsis**

| Study | Number of cases | 25(OH)D categories (as provided by each study) | Number of cases |
|---|---|---|---|
| Asilioglu 2017 | 30 sepsis | Deficiency: <20 ng/mL | 20 |
| | | Sufficiency: >=20 ng/mL | 10 |
| Say 2017 | 100 neonatal sepsis | Severe deficiency (group 1) <5 ng/mL. | 63 |
| | | Insufficiency (group2): 5 to 15 ng/mL | 24 |
| | | Sufficiency (group 3) ≥15 ng/mL | 13 |
| El-Gamasy 2017 | 46 sepsis | Serious deficiency: < 30 nmol/L | 20 |
| | | Insufficiency: 30-75 nmol/L | 18 |
| | | Adequate levels >75 nmol/L | 8 |
| Sankar 2017 | 43 septic shock | Severe deficiency: serum 25 (OH) D <10 ng/mL | 31 |
| Shah 2016 | 100 sepsis at admission | 25(OH)D <20 ng/mL | 84 |
| | | 25(OH)D >=20 ng/mL | 26 |
| Ponnarmeni 2016 | 124 sepsis | Deficiency: <50nmol/L | 63 |
| | | Insufficiency: 50-75 nmol/L | 31 |
| | | <75, insufficient + deficient | 94 |
| | | Sufficiency: >75 nmol/L | 30 |
| Bustos 2016 | 10 sepsis intraabdominal | Deficiency: < 20 ng/ml | 7 |
| | | Normal levels: > 20 ng/mL | 3 |
| Ebenezer 2016 | 16 shock | 25(OH)D <20 ng/mL | 8 |
| | | 25(OH)D >=20 ng/mL | 8 |
| Onwuneme 2015 (1) | 35 culture positive sepsis | 25(OH)D <50 ng/mL | 32 |
| | | 25(OH)D >=50 ng/mL | 3 |
| Onwuneme 2015 (2) | 46 culture positive sepsis and late-onset sepsis | Deficiency: < 30 nmol/L | 32 |
| | | Sufficiency: ≥ 30 nmol/L | 14 |
| Prasad 2015 | 11 positive blood culture | 25(OH)D <20 ng/mL | 9 |
| | | 25(OH)D >=20 ng/mL | 2 |
| Korwutthikulrangsri 2015 | 17 shock and septicaemia | 25(OH)D <20 ng/mL | 14 |
| | | 25(OH)D >=20 ng/mL | 3 |
| Cizmez 2015 | 40 suspected sepsis | Deficiency: ≤20 ng/ml | 28 |
| | | Insufficiency: 21–29 ng/ml | 7 |
| | | Normal levels: ≥30 ng/ml | 5 |
| Dayal 2014 | 9 nosocomial sepsis | 25(OH)D <20 ng/mL | 4 |
| | | 25(OH)D >=20 ng/mL | 5 |
| Hebbar 2014 | 30 shock and/or Sepsis | 25(OH)D <20 ng/mL | 17 |
| | | 25(OH)D >=20 ng/mL | 13 |
| McNally2012 | 48 septic | 25(OH) D levels: <50 nmol/L | 33 |
| | | 25(OH) D levels: ≥50 nmol/L | 15 |



For peer review only - http://bmjopen.bmj.com/site/about/guidelines.xhtml

| **Additional Table 5 Studies with thresholds other than < 50 nmol/L** | |
|---|---|
| **Study** | **Threshold used by study** |
| Roth 2009 | < 40 nmol/L |
| Roth 2010 | < 40 nmol/L |
| Say 2017 | <=15 ng/mL (37.4 nmol/L) |
| Inamo 2011 | <=15 ng/mL (37.4 nmol/L) |
| Ayulo 2014 | <=15 ng/mL (37.4 nmol/L) |
| Dinlen 2016 | <=15 ng/mL (37.4 nmol/L) |
| Onwuneme 2015 (2) | < 30 nmol/L |
| Yaghmaie 2017 | < 30 ng/mL (74.88 nmol/L) |
| Gamasay 2017 | < 30 nmol/L |
| Banajeh 2009 | < 30 nmol/L |
| Sankar 2017 | <= 10 ng/mL (24.9 nmol/L) |
| Cetinkaya 2015 | <= 10 ng/mL (24.9 nmol/L) |
| 25(OH) D values nmol/L = ng/mL * 2.496 | |

For peer review only



For peer review only - http://bmjopen.bmj.com/site/about/guidelines.xhtml

**Additional Table 6 Excluded studies**

| Study | Design | Sample size | Characteristics of pediatric population | Country | Reasons for exclusion of paper |
|---|---|---|---|---|---|
| Seliem 2016 | Hospital-based case-control | Cases, n= 30<br>Controls, n= 30 | Cases: group 1, full-term neonates with EOS and their mothers.<br>Controls, group 2: 30 full-term healthy neonates and their mothers with no clinical or laboratory evidence of sepsis. | Egypt | Requested data without reply or data not available |
| Gamal 2017 | Case-control | Cases, n= 50<br>Control, n= 30 | Cases: neonates with early onset neonatal sepsis that occurred at <72 hours<br>Controls: Age and sex healthy neonates with no prenatal risk factor for early neonatal sepsis enrolled in the study as a control group. | Egypt | Requested data without reply or data not available |
| Aydemir 2014 | Hospital-based case control | Cases, n=40<br>Controls, n= 20 | Cases: children with sepsis between 1 and 16 years old<br>Controls: children without sepsis | Turkey | Requested data without reply or data not available |
| Garg 2016 | Hospital-based case-control | Cases, n= 40<br>Controls, n= 40 | Cases: children from 6 months to 5 years of age admitted or attending OPD in department of Pediatrics.<br>Controls: children receiving care at the Hospital's ambulatory, emergency or in-patient units and presenting with non-respiratory complaints also not having any clinical indication of vitamin D deficiency. | India | Requested data without reply or data not available |



For peer review only - http://bmjopen.bmj.com/site/about/guidelines.xhtml

**Additional Table 7 Characteristics of the 48 included studies**

| Study | Design | Number of cases (controls, where appropriate) | Characteristics of population | Country and setting | Vitamin D thresholds as defined by the study | Quality score (NOS) |
|---|---|---|---|---|---|---|
| Asilioglu 2017 | Historical cohort (single centre) | 250 | Cases: aged 1 month to ≤18 years | Turkey, PICU | Deficiency: <20 ng/mL | 7 |
| Halwany 2017 | Cohort (single centre) | 102 | Cases: children aged >1 month to ≤ 5 years | Egypt, PICU | Deficiency: <20 ng/ml<br>Insufficiency: 20-29.9 ng/ml<br>"Normal" levels: 30 ng/ml | 6 |
| Hurwitz 2017 | Prospective cohort (single centre) | 90 | Cases: aged <5 years hospitalized with LRTI and RSV and/or hMPV | USA, PICU | Deficiency: <20 ng/mL | 6 |
| Garcia-Soler 2017 | Cohort (single centre) | 340 | Cases: aged 6 months to 17 years<br>Critically ill with various conditions | Spain, PICU | Deficient: <20 ng/mL<br>Insufficiency: 20-30 ng/mL<br>25(OH)D levels: 30-40 ng/mL<br>Optimal levels >40 ng/mL | 7 |
| Badawi 2017 | Cohort (single centre) | 88 | Cases: 1 month to 12 years | Egypt, PICU | Deficiency: < 50nmol/l,<br>Severe deficiency: <30 nmol/l | 8 |
| Jia 2017 | Hospital-based case-control (single centre) | 110 (110) | Cases: infants (< 1 year of age) with ALRTI<br>Controls: Healthy from similar areas attending the hospital for vaccination during study period | China, PICU | Severe deficiency: <10 ng/ml<br>Deficiency: 10-20 ng/ml<br>Insufficiency: 21-30 ng/ml<br>Sufficiency: >30 ng/ml | 7 |
| Yaghmaie 2017 | Cross sectional (single centre) | 82 | Cases: hospitalized in PICU | Iran, PICU | Deficiency: <30 ng/ml | 4 |
| Say 2017 | Case-control (single centre) | 100 (13) | Cases: premature infants less than 37 weeks diagnosed with early or late -onset neonatal sepsis<br>Controls: From same population of neonates with sepsis but not vitamin D deficient | Turkey, NICU | Severe deficiency (group 1) <5 ng/mL.<br>Insufficiency (group2): 5 to 15 ng/mL<br>Sufficiency (group 3) ≥15 ng/mL | 6 |
| El-Gamasy 2017 | Case-control | 80 (20) | Cases: 3 months to 12 years hospitalized with acute kidney injury | Egypt, PICU | Seriously deficient: < 30 nmol/L | 7 |



For peer review only - http://bmjopen.bmj.com/site/about/guidelines.xhtml

| | | | | | | |
|---|---|---|---|---|---|---|
| | (single centre) | | Controls: completely healthy subjects | | Insufficient: 30-75 nmol/L<br>Adequate >75 nmol/L | |
| Sankar 2017 | Cohort (single centre) | 43 | Cases: <=17 years of age | India, PICU | Severe vitamin D deficiency: serum 25 (OH) D <10 ng/mL | 8 |
| Shah 2016 | Cohort (single centre) | 154 | Cases: aged between 1 month and 15 years | India, PICU | Deficiency: <20 µg/mL | 6 |
| Ponnarmeni 2016 | Case-control (single centre) | 124 (338) | Cases: aged 1–12 years admitted with a diagnosis of sepsis<br>Controls: from previous prospective study, apparently healthy children of upper socioeconomic status who attended the out-patient department for immunization or with minor ailments | India, PICU | Deficiency: < 50 nmol/L<br>Insufficiency: 50-75 nmol/L<br>Sufficiency: > 75 nmol/L | 6 |
| Sankar 2016 | Cohort (single centre) | 101 | Cases: aged 1 month to 17 years | India, PICU | Deficiency: ≤ 20 ng/ml<br>Severe deficiency: <15 ng/mL | 8 |
| Bustos 2016 | Cohort (single centre) | 90 | Cases: critically ill, greater than 37 weeks and less than 15 years of age | Chile, PICU | Deficiency: < 20 ng/ml<br>Normal levels: > 20 ng/mL | 7 |
| Ebenezer 2016 | Cohort (single centre) | 52 | Cases: <18 years of age; medical and surgical diagnoses | India, PICU | Deficiency: < 20 ng/ml<br>Insufficiency: 20–30 ng/ml<br>Normal levels: ≥ 20 ng/L | 6 |
| Elmoneim 2016 | Case-control (single centre) | 30 | Cases: aged less than 14 years<br>Controls: with "normal" vitamin D levels | Saudi Arabia, PICU | Deficiency: < 20ng/ml<br>Insufficiency: 20-30 ng/mL<br>Normal: > 30 ng/mL | 5 |
| Jat 2016 | Hospital-based case-control (single centre) | 50 (50) | Cases: 1 month to 12 years of age admitted with pneumonia<br>Controls: admitted for reasons other than respiratory symptoms, required blood sampling | India, PICU | Deficiency: < 20 ng/ml<br>Insufficiency: 20-30 ng/ml Sufficiency: >= 30 ng/ml | 8 |
| Narang 2016 | Hospital-based case-control (single centre) | 50 (50) | Cases: 2 months to 5 years of age admitted as in-patients or seen in the outpatient department with ALRI<br>Controls: healthy, same age group, attending outpatients' service for immunization or admitted for minor conditions other than ALRI | India, PICU | Severe deficiency: < 20ng/ml | 6 |
| Dinlen 2016 | Hospital-based case-control | 30 (30) | Cases: term neonates with ALRI<br>Controls: healthy neonates, same age as the study group. | Turkey, NICU | Deficient: <= 15 ng/mL | 6 |



For peer review only - http://bmjopen.bmj.com/site/about/guidelines.xhtml

| | | | | | | |
|---|---|---|---|---|---|---|
| | (single centre) | | | | Severe deficiency: <= 5 ng/mL | |
| Lopez 2016 | Prospective cohort (multi centre) | 347 | Cases: 0 to >48 months admitted to hospital with ALRI prospectively recruited through the GENDRES (GENetic,vitamin D and RESpiratory infections research network) | Spain, PICU | 25-OHD levels: <10 ng/mL<br>25-OHD levels: 10-20 ng/mL<br>25-OHD levels: 20-30 ng/mL | 8 |
| Alvarez 2016 | Cross-sectional (single centre) | 50 | Cases: patients aged 0 to 18 years | USA, PICU | 25(OH)D < 20 ng/mL<br>25(OH)D < 30 ng/mL | 6 |
| Onwuneme 2015 (1) | Case-control (single centre) | 120 (30) | Cases: with suspected sepsis (<12 years old)<br>Controls: paediatric controls admitted for elective day case surgery during the same study period and were not suspected of having sepsis | Ireland , PICU | 25(OH) D levels: <50 nmol/L<br>25(OH) D levels: ≥50 nmol/L | 8 |
| Onwuneme 2015 (2) | Cohort (single centre) | 94 | Cases: preterm infants <32 weeks gestation | Ireland, NICU | Deficiency: < 30 nmol/L<br>Sufficiency: ≥ 30 nmol/L | 8 |
| Prasad 2015 | Cohort (single centre) | 80 | Cases: 2 months to 12 years old | India, PICU | Deficiency: < 20 ng/ml<br>Sufficient: ≥ 20 ng/mL | 7 |
| Moreno-Solis 2015 | Hospital-based case-control (single centre) | 48 (30) | Cases: aged 1–11 months with acute bronchiolitis<br>Controls: healthy, <12 months, admitted to the outpatient clinic without respiratory symptoms or history of hospitalization for bronchiolitis or wheezing | Spain, PICU | Deficiency: < 20 ng/ml<br>Insufficiency: 21–29 ng/ml Sufficiency: ≥ 30 ng/ml | 6 |
| Alonso 2015 | Cross sectional (single centre) | 288 | Cases: aged 1 month to 13 years | Spain, PICU | Deficient: < 20 ng/ml and < 10 ng/ml | 5 |
| Korwutthikulrangsri M 2015 | Nested case-control (single centre) | 32 (36) | Cases: requiring PICU admission<br>Controls: Healthy, enrolled during the same period of time and served as the control group (age in months) | Thailand, PICU | Deficiency: < 20 ng/mLl<br>Insufficiency: 20-29.9 ng/ml<br>Sufficiency: ≥ 30 ng/mL | 5 |
| Khakshour 2015 | Case-control | 37 (53) | Cases: below 5 years of age and suffering from respiratory infections<br>Controls: those who were not suffering from respiratory infections | Iran, PICU | Deficiency: < 20 ng/mL | 5 |
| Cizmeci 2015 | Case-control (single centre) | 40 (43) | Cases: infants with suspected early-onset neonatal sepsis.<br>Controls: For each newborn of group 1, one healthy infant selected as a control | Turkey, NICU | Deficiency: ≤ 20 ng/ml<br>Insufficiency: 21–29 ng/ml | 7 |



For peer review only - http://bmjopen.bmj.com/site/about/guidelines.xhtml

| | | | | | | |
|---|---|---|---|---|---|---|
| | | | | | Normal levels: ≥ 30 ng/ml | |
| Cetinkaya 2015 | Hospital-based case-control (single centre) | 50 (50) | Cases: term infants with clinically suspected (probable) early infection (early-onset sepsis) within the first 3 postnatal days of life and were >37 weeks of gestational age<br>Controls: healthy infants with no signs of clinical/laboratory infection | Turkey, NICU | Severe deficiency: <10 ng $ml^{-1}$<br>Insufficiency: 11 to 32 ng $ml^{-1}$<br>Adequacy: 32 to 100 ng $ml^{-1}$ | 6 |
| Ayulo 2014 | Cohort (single centre) | 216 | Cases: between the ages of 1 and 21 years | Spain, PICU | Deficient: < 15 ng/ml<br>Insufficient: 15-29 ng/mL<br>Sufficient: ≥ 30 ng/mL | 7 |
| Dayal 2014 | Cohort (single centre) | 92 | Cases: Children aged 3 months to 12 years | India, PICU | Deficiency: < 50 nmol/L<br>Insufficiency: 50–75 nmol/L<br>Sufficiency: > 75 nmol/L<br>25(OH) D levels: < 75 nmol/L | 6 |
| Hebbar 2014 | Case-control (single centre) | 61 (46) | Cases: children 0 to 18 years<br>Controls: patients recruited among children in the magnetic resonance imaging suite. | USA, PICU | Deficient: ≤ 10 ng/ml<br>Insufficient: 10 to 20 ng/ml<br>Sufficient: ≥ 20 ng/mL | 6 |
| Rey 2014 | Case-control (single centre) | 156 (289) | Cases: heterogeneous group of critically ill children aged <16 years<br>Control group for comparison: population of healthy children | Spain, PICU | Deficient: < 20 ng/ml | 8 |
| Ahmed 2014 | Hospital-based case-control (single centre) | 50 (50) | Cases: aged 2–60 months hospitalized with ALRI<br>Controls: age-matched with cases within 1 or 2 months, attending well-child clinics or general clinics without evidence of respiratory infection or admitted to the hospital for elective surgery | Africa, PICU | Deficiency: <50 nmol/L | 8 |
| Basha 2014 | Hospital-based case-control (single centre) | 81 (89) | Cases: under 5 years old with severe pneumonia selected from the inpatient departments and emergency units of the hospital<br>Controls: healthy, selected from the outpatient surgical clinics of the hospital during their visit for umbilical or inguinal hernia repair and not suffering from upper or lower respiratory infections | Egypt, PICU | Deficiency: < 50 nmol/L<br>Insufficiency: < 75 nmol/L<br>Sufficiency: > 75 nmol/L | 7 |



For peer review only - http://bmjopen.bmj.com/site/about/guidelines.xhtml

| | | | | | | |
|---|---|---|---|---|---|---|
| Sakka 2014 | Population based case-control (single centre) | 96 (96) | Cases: <2 years old with ALRI, 48 diagnosed with pneumonia and 48 with bronchiolitis<br>Controls: age and sex matched with no respiratory symptoms or signs from the Health office | Egypt, PICU | Severe deficiency: 0–5 ng/mL Deficiency: 5–15 ng/mL Insufficiency: 15– 20 ng/mL Sufficiency: 20–100 ng/mL | 8 |
| Cayir 2014 | Hospital-based case-control (single centre) | 88 (81) | Cases: 1 to 13 years diagnosed with acute otitis media<br>Controls: Healthy same age range | Turkey, PICU | Normal levels: 20 ng/mL<br>Insufficiency: 15-20ng/mL<br>Deficiency: <15ng/mL | 7 |
| Binks 2014 | Cross-sectional | 74 | Cases: aged <3 years admitted with acute lower respiratory infections (ALRIs) or other conditions | Australia, PICU | 25(OH) D levels: < 50 nmol/L | 6 |
| Madden 2012 | Cohort (single centre) | 511 | Cases: less than 21 years old | USA, PICU | 25(OH) D levels: < 10 ng/mL<br>25(OH) D levels: 10-19.9 ng/mL<br>Deficiency: < 20 ng/ml<br>Insufficiency: < 30 ng/mL | 8 |
| Rippel 2012 | Cohort (single centre) | 316 | Cases: children aged 16.5 (3.1–75.2) months | Australia, PICU | Deficiency: < 50 nmol/L<br>"Normal" levels: ≥ 50 nmol/L | 8 |
| McNally 2012 | Cohort (multi-centre) | 326 | Cases: Newborn to 17 years of age | Canada, PICUs | Deficiency: < 50 nmol/L<br>25(OH) D levels: 50 to 75 nmol/L | 7 |
| Inamo 2011 | Cohort (single centre) | 28 | Cases: between 1 and 48 months hospitalized with ALRI, 26 diagnosed with bronchiolitis and two as having pneumonia | Japan, PICU | Deficiency: ≤ 15ng/ml<br>Severe deficiency: ≤ 5ng/mL | 6 |
| Roth 2010 | Hospital-based case-control (single centre) | 25 (25) | Cases: 1–18 months hospitalized with ALRI<br>Controls: selected by population-based sampling. aged 1–23 months, and matched to cases on age (±2 months) and sex | Bangladesh, PICU | Deficiency: < 40 nmol/L | 8 |
| Banajeh 2009 | Prospective cohort (single centre) | 79 | Cases: 2–59 months with WHO-defined very severe community acquired (VSP) pneumonia | Iran, PICU | Deficiency: < 30 nmol/L | 7 |
| Karatekin 2009 | Hospital-based case-control, | 25 (15) | Cases: newborns with acute respiratory infections | Turkey, NICU | Deficiency: < 20 ng/ml | 6 |



For peer review only - http://bmjopen.bmj.com/site/about/guidelines.xhtml

| | | | | | | |
|---|---|---|---|---|---|---|
| | (single centre) | | Controls: healthy, age matched from outpatients' service where they went for immunization. | | | |
| Roth 2009 | Hospital-based case-control (single centre) | 64 (65) | Cases: aged 1-25 months admitted with ALRI<br>Controls: aged 1-25 months undergoing elective surgery, no history of hospitalization for ALRI | Canada, PICU | Deficiency: < 40 nmol/L | 7 |
| Wayse 2004 | Hospital-based case-control, (single centre) | 80 (70) | Cases: < 5 years with severe ALRI<br>Controls: healthy, attending outpatients service for immunization | India, PICU | Deficiency: Plasma 25(OH)D3 < 50 nmol/L | 6 |

LRTI = lower respiratory tract infection; PICU = paediatric intensive care unit; RSV = respiratory syncytial virus; ALRI = acute lower respiratory infection; WHO = World Health Organization; VSP = very severe community acquired pneumonia; NICU = neonatal intensive care unit.



For peer review only - http://bmjopen.bmj.com/site/about/guidelines.xhtml

**Additional Table 8 Objectives and outcomes of included studies**

| Study | Objectives/aims of study | Main outcome(s) and conclusion(s) |
|---|---|---|
| Madden 2012 | Prevalence of vitamin D deficiency in critically ill children and factors influencing admission 25-hydroxyvitamin D (25(OH)D) levels | Identified high prevalence of vitamin D deficiency and insufficiency in critically ill children. Inverse association severity of illness on admission and 25(OH) levels |
| Lopez 2016 | Role of Vitamin D in Children hospitalized with Lower Tract Acute Respiratory Infections | 25-hydroxyvitamin D levels of study population below normal range. Correlation of higher disease severity with lower levels of 25-hydroxyvitamin D. |
| Garcia-Soler 2017 | Prevalence and risks factors of vitamin D deficiency, as well as its relationship with length of PICU stay morbidity and mortality in a PICU. | Vitamin D deficiency is frequent in paediatric critical patients. It also has an association with higher severity scores, season of year and parental educational attainment.<br>Vitamin D levels associated with various laboratory parameters of SIRS. Vitamin D deficiency associated with increased risk of morbidity and mortality. Inconclusive findings on its association with PICU length of stay were inconclusive |
| McNally 2012 | Prevalence of vitamin D deficiency, risk factors and potential association with clinically relevant outcomes in critically ill children in Canada. | Most of critically ill children vitamin D deficient at PICU admission. Lower 25(OH) D levels associated with increased catecholamine requirements, fluid bolus administration, hypocalcemia, and longer PICU admission. |
| Rippel 2012 | Prevalence of hypovitaminosis D and association with outcome in critically ill children requiring admitted in intensive care. | Hypovitaminosis D is frequent in critically ill children in PICU, especially in infants and children with heart disease. Hypovitaminosis D associated with hypocalcemia in non-cardiac population, and increased need for calcium replacement in the cardiac population.<br>No association between vitamin D status and survival or PICU length of stay. Strong association with early postoperative inotropic needs in the cardiac population. |
| Alonso 2015 | Investigate relationship of serum 25-hydroxyvitamin D concentrations with serum parathyroid hormone (PTH) levels, body mass index (BMI), and environmental factors in a population of Caucasian children living at latitude 43°N. | Results doubt the assumption that a serum 25OH D threshold indicates vitamin D deficiency in children. |
| Asilioglu 2017 | Measure occurrence of VDD in critically ill children. Assess determinants of vitamin D status and compare vitamin D deficient and sufficient cases in respect of severity of illness. | Hypovitaminosis D occurrence high in critically ill children and associated with higher vasopressor requirement. Not associated with other markers of illness severity including mortality. |
| Ayulo 2014 | Prevalence of vitamin D deficiency among children in PICU | Vitamin D deficiency common. No significant correlation between disease severity and vitamin D levels levels of vitamin D. Mortality associated with vitamin D levels |
| Rey 2014 | Identify prevalence of 25 hydroxivitamin D or 25(OH) vitamin D deficiency on pediatric intensive care unit (PICU) admission, and if associated with increased prediction of mortality risk scores. | Hypovitaminosis D incidence high in PICU patients. Hypovitaminosis D not associated with higher prediction of risk mortality scores. |
| Shah 2016 | Determine prevalence of vitamin D deficiency in critically ill children its association with illness severity, parathyroid response and clinical outcomes. | High prevalence of vitamin D deficiency. Parathyroid gland response secondary vitamin D deficiency or hypocalcemia impaired in critically ill. |
| Ponnarmeni 2016 | Vitamin D deficiency in critically ill children with sepsis admitted to PICU and its association with: mortality, length of stay, illness severity, requirement for ventilation and catecholamines | High prevalence of vitamin D deficiency<br>No significant association between vitamin D deficiency and other outcomes such as mortality |
| Onwuneme 2015 (1) | Assess vitamin D status, and its determinants, in chidren with suspected sepsis admitted to PICU. Also investigated association between vitamin D status and clinical outcomes. | Children admitted to the PICU with suspected sepsis lower 25OH D compared to controls. Inadequate 25 OH D levels associated with confirmed sepsis and poor outcomes. |
| Jia 2017 | Association of vitamin D with ALRTI in Chinese infants | Lowered plasma level of 25-OH Vitamin D makes children susceptible to ALRTI. |
| Halwany 2017 | Frequency of vitamin D deficiency in critically ill pediatric intensive care unit [PICU] patients and relation to state of serum 25(OH) D to disease severity. | High prevalence of vitamin D deficiency in critically ill children.<br>Negative correlation of Vitamin D level with PELOD score. Recommend screening of critically ill children for vitamin D deficiency to restore their serum levels. |



For peer review only - http://bmjopen.bmj.com/site/about/guidelines.xhtml

| | | |
|---|---|---|
| Sankar 2016 | Prevalence of vitamin D deficiency on admission and examine association with length of ICU stay | High prevalence of vitamin D deficiency. Vitamin D deficient children with longer ICU stay duration or mortality |
| Say 2017 | Evaluate effects of low vitamin D levels in cord blood on neonatal sepsis in preterm infants. | No significant relationship between the cord blood vitamin D levels and the risk of neonatal sepsis in premature infants. |
| Sakka 2014 | Determine the relation between vitamin D deficiency, anemia and the severity of ALRTIs in hospitalized children. | Vitamin D deficiency, low BMI, low hemoglobin level, rachitic signs were risk factors for the severity of ALRTIs. |
| Onwuneme2015 (2) | Investigate the association between serum 25-hydroxyvitamin D (25OHD) levels and outcomes in preterm infants (<32 weeks gestation). | High prevalence of low 25OHD. Association between vitamin D status and acute respiratory morbidity in preterm infants after birth. In none of the following outcomes was the difference statistically significant: surfactant use, inotrope requirement, RDS, pneumothorax, pulmonary hemorrhage, chronic lung disease, sepsis, retinopathy of prematurity, intraventricular hemorrhage, periventricular leucomalacia, patent ductus arteriosus, and mortality |
| Dayal 2014 | Prevalence of vitamin D deficiency<br>Association of serum vitamin D levels with duration of stay in hospital, mortality and requirement of ventilation | Reduced serum vitamin D levels in children |
| Bustos 2016 | Determine prevalence of vitamin D deficiency and its association with other clinically relevant outcomes in children admitted to Paediatric Intensive Care Unit | Vitamin D deficiency was prevalent in critically ill children and associated with adverse clinical outcomes. |
| Hurwitz 2017 | Measure retinol binding protein and vitamin D in children aged <5 years hospitalized with lower respiratory tract infection and respiratory syncytial virus and/or human meta pneumovirus detections | Low vitamin levels in 50% of the children and associated with significantly elevated risk of the need for intensive care unit admission and invasive mechanical ventilation. |
| Cayir 2014 | Investigate the relationship between Vitamin D deficiency and acute otitis media infection | Serum 25-hydroxy vitamin D levels significantly lower in children with acute otitis media compared to the controls. Vitamin D deficiency plays a role in otitis media infection. |
| Badawi 2017 | Investigated if VDD is related to higher severity scores and organ dysfunction. Primary objective of study was to estimate the prevalence of VDD in a group of critically ill children, and secondary objectives was to correlate vitamin D status with pediatric logistic organ dysfunction (PELOD) and pediatric risk ofmortality III (PRISM III) scores. | VDD prevalence was reported in about half of the critically ill patients, and it was observed to be related to multiple organ dysfunctions and rapid clinical deterioration. |
| Yaghmaie 2017 | Investigate relation of vitamin D deficiency with potential demographic and clinical factors. | Vitamin D deficiency among paediatric intensive care unit patients similar to western countries, also with similar age and BMI distribution. Significant relation observed between age and serum level of vitamin D. |
| Basha 2014 | Aimed to evaluate vitamin D status as a risk factor for severe pneumonia in Egyptian hospitalized children under 5 years | Significant association between vitamin D deficiency and severe pneumonia in Egyptian children below 5 years |
| Prasad 2015 | Prevalence of vitamin D deficiency in critically ill children and association with illness severity& other outcomes | High prevalence of vitamin D deficiency in critically ill children and association with PRISM III scores in a developing country.<br>Groups did not differ in terms of PICU stay, duration of hospital stay, culture positivity, biochemical parameters (serum calcium, serum phosphate), need of ventilation or steroids, presence of coagulopathy and mortality. |
| Wayse 2004 | If vitamin D deficiency in Indian children under 5 years old of age is risk factor for severe acute lower respiratory infection (ALRI). | Subclinical vitamin D deficiency & nonexclusive breastfeeding in four first months of life found to be significant risk factors for severe ALRI in Indian children. |
| El-Gamasy 2017 | Assess serum 25 (OH)D level in critically ill paediatric patients with AKI at PED of Tanta University Emergency Hospital (TUEH) within the first 24 hours of admission and evaluate its correlation with duration of hospital stay and mortality outcome. | Vitamin D deficiency associated with higher incidence of sepsis and mortality. |



For peer review only - http://bmjopen.bmj.com/site/about/guidelines.xhtml

| | | |
|---|---|---|
| Banajeh 2009 | Determine if rickets and VDD predict the outcomes in very severe pneumonia (VSP). | In WHO-defined VSP, nutritional rickets was strongly associated with a reduced successful treatment outcome, and VDD was a significant and independent predictor of reduced circulating PMNs and persistent hypoxemia. |
| Binks 2014 | Aimed to determine prevalence of vitamin D insufficiency among children hospitalised with ALRI in the Northern Territory. | Vitamin D insufficiency was observed in about one-third of these hospitalised children. Children hospitalised with an ALRI less likely to have vitamin D insufficiency compared with children hospitalised for other conditions (predominantly gastroenteritis). |
| Roth 2009 | Test the hypothesis that vitamin D status is associated with the risk of ALRI in Canadian children (1 month to 2 years old) | Among children aged 1 month to 2 years, vitamin D status not associated with ALRI requiring hospitalization |
| Hebbar 2014 | Prevalence of vitamin D insufficiency and relationship between vitamin D levels infection and innate immunity | High prevalence of vitamin D deficiency<br>Serum vitamin D levels - no correlation with illness severity score |
| Ebenezer 2016 | Determine vitamin D status and association with outcomes | Vitamin D deficiency common among pediatric patients<br>No association between vitamin D status and admission and mortality such as higher mortality and/or longer PICU stay<br>Deficiency associated with mechanical ventilation severity of illness, vasopressor need |
| Narang 2016 | Identify an effective nutritional agent that reduces the need for antibiotics, duration of pneumonia and length of hospitalization would be highly cost-beneficial. | Severe vitamin D deficiency (<20 ng/ml), nonexclusive breastfeeding in the first six months of life, inadequate sun exposure found to be significant risk factors for ALRTI in Indian children. |
| Alvarez 2016 | Study aimed to investigate relationship between vitamin D status and plasma markers of lutathione (GSH) and cysteine (Cys) redox and immunity in critically ill children | Vitamin D sufficiency was associated with more reduced plasma hCySS, indicative of lower oxidative stress, in critically ill children. Plasma GSH, GSSG, and glutamine, however, were lower in the vitamin D sufficient group. Vitamin D role in maintaining redox status during pediatric critical illness requires further study. |
| Cetinkaya 2015 | Evaluate the effect of vitamin D levels on early-onset sepsis (EOS) in term infants | Lower maternal and neonatal 25-OHD levels associated with EOS. Suggest that adequate vitamin D supplementation during pregnancy may be helpful to prevent EOS in term neonates. |
| Ahmed 2014 | Examine relationship between vitamin D status and hospitalization for ALRTI in Nigerian children. | ALRTI not associated with vitamin D status, but associated with less exposure to sunlight. Exposure to sunlight & vitamin D supplementation contributed to vitamin D status in this population. |
| Jat 2016 | Evaluate Vitamin D levels and its correlation with severity and outcome of pneumonia in children. | Majority (86.4%) of children were vitamin D deficient. Vitamin D levels were found different in cases and controls and were not related to severity and outcome of pneumonia. |
| Sankar 2017 | Evaluate association of severe vitamin D deficiency with clinically important outcomes in children with septic shock. | Prevalence of severe vitamin D deficiency is high in children with septic shock admitted to pediatric intensive care unit. Severe vitamin D deficiency at admission seems to be associated with lower rates of shock reversal at 24 hours of ICU stay. |
| Cizmeci 2015 | Investigate if neonates with early-onset neonatal sepsis (EONS) had lower levels of vitamin D | Cord-blood 25(OH) D levels of neonates with EONS significantly lower than that of the healthy controls, and a low level of cord-blood vitamin D was found to be associated with an increased risk of EONS. Further studies are warranted to confirm this association. |
| Khakshour 2015 | Clarify the association between vitamin D deficiency and acute respiratory infection in children below age 5 years. | The group of children with respiratory disorders, 9 (42.9%) exhibited vitamin D deficiency. No meaningful statistical relation vitamin D deficiency with acute respiratory infections (p>0.05) |
| Korwutthikulrangsri 2015 | Determine vitamin D status in critically ill children and its relationship with adrenal function | Higher prevalence of vitamin D deficiency in critically ill children in comparison to controls. Patients vitamin D deficiency had higher median (IQR) PRISM III score and higher proportion of mortality than those with serum 25-OHD of equal or more than 12 ng/mL. |
| Elmoneim 2016 | Association of the level of vitamin D on admission & length of stay in the PICU, or duration of mechanical ventilation. | High prevalence of vitamin D deficiency among PICU patients & significant association with increased LOS and need for mechanical ventilation. Not significant association with mortality rate. |



For peer review only - http://bmjopen.bmj.com/site/about/guidelines.xhtml

| | | |
|---|---|---|
| Dinlen 2016 | Determine the association between serum 25-hydroxy vitamin D [25(OH)D] levels and acute respiratory tract infections (ALRTI) in newborns | Lower blood 25(OH) D levels might be associated with increased risk of ALRTI in term newborn babies. Appropriate vitamin D supplementation during pregnancy and early childhood may enhance newborns' respiratory health. |
| Moreno-Solis 2015 | Examine prevalence of hypovitaminosis D in infants with acute bronchiolitis compared with control subjects and to evaluate the relationship between serum 25-hydroxyvitamin D (25(OH) D) and the severity of bronchiolitis. | Prevalence of hypovitaminosis D is high in Spanish infants with bronchiolitis. The severity of acute bronchiolitis increases with a decline in serum 25 (OH) D level. |
| Inamo 2011 | Relationship between serum vitamin D concentrations and severity of ALRI in hospitalized children in Japan. | Significantly more children with ALRI that required supplementary oxygen and ventilator management were vitamin D deficient. Findings suggest: immunomodulatory properties of vitamin D may influence the severity of ALRI. |
| Karatekin 2009 | Association of serum 25-hydroxy vitamin D concentrations in newborns with acute lower respiratory infection (ALRI) and without clinical signs of rickets, and their mothers | Newborns with subclinical vitamin D deficiency may have an increased risk of suffering from ALRI. Strong positive correlation between newborns' and mothers' 25(OH) D concentrations indicates that adequate vitamin D supplementation of mothers should be emphasized during pregnancy especially in winter months. |
| Roth 2010 | Investigate association between vitamin D status and ALRI | Vitamin D status associated with ALRI in early childhood.<br>Randomized trials needed to establish if interventions to improve vitamin D status could reduce burden of ALRI in early childhood. |

For peer review only



For peer review only - http://bmjopen.bmj.com/site/about/guidelines.xhtml

**Additional Table 9 Assay used in each study to measure Vitamin D levels**

| Assay | Paper | Number of studies |
|---|---|---|
| Radioimmunoassay | Madden 2012; Inamo 2011; Karatekin 2009; Roth 2009; Roth 2010; Sakka 2014; Moreno-Solis 2015; Khashour 2015 | 8 |
| Competitive binding enzyme linked immunoassay | Basha 2014 | 1 |
| Binding protein assay | Onwuneme 2015 (2) | 1 |
| Liquid chromatography-mass spectrometry | McNally 2012; Korwutthikulrangsri 2015; Binks 2014; Cetinkaya 2015; Cizmeci 2015; Asilioglu 2017; Onwuneme (1) | 7 |
| Chemiluminescence immunoassay or chemiluminescent tracer | Alonso 2015; Rey 2014; Sankar 2016; Shah 2016; Prasad 2015; Ahmed 2015; Lopez 2016; Alvarez 2016; Say 2017; Sankar 2017; Jat 2017 | 11 |
| ELISA | Ponnarmeni 2016; Hebbar 2014; Elmoneim 2016; Narang 2016; Jia 2017; El-Gamasy 2017; Halwany 2017; Banajeh 2009; Badawi 2017 | 9 |
| ELFA (enzyme linked fluorescent assay) | Bustos 2014 | 1 |
| Electrochemiluminescence Immunoassay (ECLIA) | Dayal 2014, Ebenezer 2016, Garcia Soler 2017; Cayir 2014; | 4 |
| Clinical Laboratory Improvement Amendments–approved Vitamin D assay (Elecsys; Roche Diagnostics, Indianapolis, Indiana) | Hurwitz 2017 | 1 |
| Commercial immunoassay lit (I RIA Diasorin UK) | Wayse 2004, Ayulo 2014, | 2 |
| Immunoassay analyzer | Rippel 2012 | 1 |
| APPLIED 3200 Biosystem | Dinlen 2016 | 1 |
| Not reported | Yaghmaie 2017 | 1 |

For peer review only

| Additional Table 10 Funding and ethical approval of included studies | | |
|---|---|---|
| **Study** | **Funding** | **Approval of study and ethics** |
| Madden 2012 | Reported | Children's Hospital Boston institutional review board. Informed consent obtained from family. |
| Lopez 2016 | Reported | Approved by the Ethical Committee of Clinical Investigation of Galicia (CEIC ref 010/015) and all of the regional ethics committees of the participant centres informed consent forms were obtained from either a parent or legal guardian for each subject before study inclusion. |
| Garcia-Soler 2017 | Not reported | Study protocol approved by regional research ethics committee. Obtained informed consent from family of patients. Data recorded anonymously (encrypted electronic database).<br>Project adhered to the principles of Declaration of Helsinki and standards for good clinical practice. |
| McNally 2012 | Reported | Research ethics board approval for vitamin D sub study obtained from 6 centers, representing 337 of the original 389 study participants. |
| Rippel 2012 | Not Reported | Approved by Ethics Committee of The Royal Children's Hospital Melbourne |
| Alonso 2015 | Reported | Regional Ethics Committee of the Principality of Asturias |
| Asilioglu 2017 | Not reported | Approval of study by the Local Ethics Committee of Ondokuz Mayıs University (Samsun, Turkey). |
| Ayulo 2014 | Not reported | Institutional Review Board: Montefiore Medical Centre |
| Rey 2014 | Reported | Hospital Ethics Committee |
| Shah 2016 | Reported | Ethical approval obtained from Institutional ethics committee. Parents of children satisfying criteria gave written informed consent for participation of their child in the study. |
| Ponnarmeni 2016 | Not reported | The institution's ethics committee. Informed consent obtained |
| Onwuneme 2015 (1) | Reported | Approval by ethics committees of: Children's University Hospital, Temple Street and Our Lady's Children's Hospital, Dublin, Ireland. Participants were informed and provided written consent before recruitment. |
| Jia 2017 | Not reported | Approved by Human Ethical Committee of Affiliated Hospital of Yan'an University, Yan'an. Informed consent was taken from mothers and/or parent of infants. |
| Halwany 2017 | Not reported | Approval by the Medical Ethics Committee of the Faculty of Medicine, Alexandria University |
| Sankar 2016 | Not reported | Institutional Ethics committee. Informed consent obtained |
| Say 2017 | Not reported | Approval by the local Ethics Committee and informed parental consent was obtained for all infants. |
| Sakka 2014 | Not reported | Not reported |
| Onwuneme2015 (2) | Reported | Ethics Committee of National Maternity Hospital. Informed written consent obtained from parents before recruitment |
| Dayal 2014 | Not reported | Ethics Committee of the Institute. Informed consent obtained. |
| Bustos 2016 | Reported | Comité Ético Científico del Servicio de Salud de Concepción |
| Hurwitz 2017 | Reported | Informed consent obtained and study protocol approved by the Institutional Review Boards at the University of Tennessee, St Jude Research Hospital, and the Centers for Disease Control and Prevention. |
| Cayir 2014 | Not reported | Approval from the institutional ethics committee and consent from the parents of all children in the study. |
| Badawi 2017 | Not reported | Children's Cairo University institutional review board approved study. Informed consent obtained from parents |
| Yaghmaie 2017 | Not reported | Study carried out in accordance with the Declaration of Helsinki, and the ethics committee of the Tehran University of Medical Sciences approved the protocols of the study. Records of patients' were kept confidential. Patients' consent provided for blood sampling and vitamin D serum level was assessed from patients' files, so no invasive method or extra blood sampling was done. |



For peer review only - http://bmjopen.bmj.com/site/about/guidelines.xhtml

| | | |
|---|---|---|
| Basha 2014 | Not reported | Oral consent from the mothers. |
| Prasad 2015 | Not reported | Institutional review board approved the protocol. Written informed consent obtained from parents or guardians. |
| Wayse 2004 | Reported | Information not provided |
| El-Gamasy 2017 | Not reported | Study approved from the Ethical Committee of the Faculty of Medicine, Tanta University. Informed verbal or written parental consents from all subjects involved in the study. |
| Banajeh 2009 | Reported | Protocol approved and described in previous paper |
| Binks 2014 | Reported | Testing performed after approval by the Human Research Ethics Committee of the Northern Territory Department of Health and Menzies School of Health Research |
| Roth 2009 | Reported | Caregiver of each participant provided written informed consent and completed a questionnaire Study approved by the Human Research Ethics Board of the University of Alberta Health Sciences Faculties |
| Hebbar 2014 | Reported | Institutional Review Boards of Emory University and Children's Healthcare Atlanta Informed consent obtained |
| Ebenezer 2016 | Reported | Institutional Review Board (IRB), Informed consent from parents |
| Narang 2016 | Not reported | Not reported |
| Alvarez 2016 | Reported | Study approved by both the Emory University and Children's Healthcare of Atlanta Institutional Review Boards, and informed consent was obtained from patients' guardians prior to any study procedures |
| Cetinkaya 2015 | Not reported | Study protocol approved by the local Ethics Committee. Informed parental consent was obtained for all infants |
| Ahmed 2014 | Not reported | Informed written or oral consent obtained from the parents, and the study was approved by the National Hospital Abuja Ethics Committee |
| Jat 2016 | Not reported | Ethics committee approval was taken before commencing the study. Parent's informed consent was taken before enrolling children into study. |
| Sankar 2017 | No funding | Study approved by the institutional ethics committee. |
| Cizmeci 2015 | Reported | Acquisition of cord-blood was approved by the local ethics committee of Fatih University Medical School. |
| Khakshour 2015 | Not reported | Obtained informed consent from parents, data collection done using demographic questionnaire and serum level of 25-dehydroxycalcciferol was measured. Samples taken by a trained nurse at admission based on physician's orders. |
| Korwutthikulrangsri 2015 | Not reported | Ramathibodi Hospital Ethics Committee. Written informed consent from legal guardians of all participants |
| Elmoneim 2016 | Reported | MCH hospital Ethics Committee approved study protocol |
| Dinlen 2016 | Not reported | Study approved by the Local Ethics Committee. All parents fully informed about this investigation as well as its aim. Written consent was obtained from all parents. |
| Moreno-Solis 2015 | Reported | Written informed consent obtained from parents or legal guardian of all enrolled children. Protocol of study approved by the Ethics Committee before the beginning of this study. |
| Inamo 2011 | Not reported | Ethics Committee of Nihon University Nerima-Hikarigaoka Hospital. Informed consent obtained from the parents of all patients before inclusion in the study. |
| Karatekin 2009 | Not reported | Study approved by the Institution's Ethics Committee, and informed consent was obtained from the study participants. |
| Roth 2010 | Reported | Approved by The Johns Hopkins Bloomberg School of Public Health Institutional Review Board and the ethics committee of the Bangladesh Institute for Child Health at the Dhaka Shishu Hospital, Bangladesh. Signed permission prior to enrolment from parents/guardians. |



For peer review only - http://bmjopen.bmj.com/site/about/guidelines.xhtml

| Additional Table 11 Age groups of children in each study | |
|---|---|
| **First author, date** | **Age group** |
| Wayse 2004 | <5 years |
| Karatekin 2009 | Neonates |
| Roth 2009 | <2 years |
| Roth 2010 | 1–18 months |
| Inamo 2011 | <4 years |
| Madden 2012 | <21 years |
| Rippel 2012 | 16.5 (3.1 to 75.2) months |
| McNally 2012 | Newborn to 17 years |
| Ayulo 2014 | 1 to 21 years |
| Dayal 2014 | 3 months to 21 years |
| Hebbar 2014 | 0 to 18 years |
| Rey 2014 | <16 years |
| Cetinkaya 2015 | >37 weeks |
| Onwuneme (1) 2015 | <12 years |
| Onwuneme (2) 2015 | <32 weeks gestation |
| Prasad 2015 | 2 months to 12 years |
| Alonso 2015 | 1 month to 13 years |
| Korwutthikulrangsri 2015 | 79 (61) cases; 92 (40) controls months |
| Cizmeci 2015 | Neonates |
| Shah 2016 | 1 month to 15 years |
| Ponnarmeni 2016 | 1 to 12 years |
| Sankar 2016 | 1 month to 17 years |
| Bustos 2016 | >37 weeks and <15 years |
| Ebenezer 2016 | <18 years |
| Elmoneim 2016 | <14 years |
| Narang 2016 | 2 months to 5 years |
| Dinlen 2016 | Neonates |
| Lopez 2016 | 0 to >48 months |
| Alvarez 2016 | 0 to 18 years |
| Garcia-Soler 2017 | 6 months to 17 years |
| Sankar 2017 | <17 years |
| Ahmed 2015 | 2 to 60 months |
| Cayir 2014 | 1 to 13 years |
| Say 2017 | <37 weeks |
| Asilioglu 2017 | <=18 years |
| Basha 2014 | <5 years |
| Jia 2017 | <1 year |
| Jat 2017 | 1 month to 12 years |
| Yaghmaie 2017 | Age range not stated |
| El-Gamasay 2017 | 3 months to 12 years |
| Binks 2014 | <3 years |
| Halwany 2017 | >1 month to <= 5 years |
| Badawi 2017 | 1 month to 12 years |
| Moreno-Solis 2015 | 1 to 11 months |
| Sakka 2014 | <2 years |



For peer review only - http://bmjopen.bmj.com/site/about/guidelines.xhtml

| | |
|---|---|
| Hurwitz 2017 | <5 years |
| Banajeh 2009 | 2 to 59 months |
| Khakshour 2015 | < 5 years |

**Additional Table 12 Prevalence of vitamin D deficiency in each study of critically ill children (sorted from highest to lowest)**

| Study | Prevalence of vitamin D deficiency (%) | Number of vitamin D deficient children | Total number of children |
|---|---|---|---|
| Wayse 2004 | 95.00 | 76 | 80 |
| Sakka 2014 | 92.70 | 89 | 96 |
| Karatekin 2009 | 92.00 | 23 | 25 |
| Onwuneme2015 (2) | 91.48 | 86 | 94 |
| Say 2017 | 87.00 | 87 | 100 |
| Dinlen 2016 | 86.70 | 26 | 30 |
| Cetinkaya 2015 | 84.00 | 42 | 50 |
| Jat 2016 | 84.00 | 42 | 50 |
| Roth 2010 | 84.00 | 21 | 25 |
| Prasad 2015 | 83.75 | 67 | 80 |
| Jia 2017 | 83.64 | 92 | 110 |
| Shah 2016 | 83.11 | 128 | 154 |
| Korwutthikulrangsri 2015 | 78.12 | 25 | 32 |
| Sankar 2016 | 74.26 | 75 | 101 |
| Sankar 2017 | 72.09 | 31 | 43 |
| Cizmeci 2015 | 70.00 | 28 | 40 |



For peer review only - http://bmjopen.bmj.com/site/about/guidelines.xhtml

| | | | |
|---|---|---|---|
| McNally 2012 | 69.02 | 225 | 326 |
| Basha 2014 | 65.43 | 53 | 81 |
| Yaghmaie 2017 | 64.63 | 53 | 82 |
| Hebbar 2014 | 60.66 | 37 | 61 |
| Onwuneme 2015 (1) | 59.16 | 71 | 120 |
| Alvarez 2016 | 58.00 | 29 | 50 |
| Cayir 2014 | 56.82 | 50 | 88 |
| Elmoneim 2016 | 56.67 | 17 | 30 |
| Narang 2016 | 56.00 | 28 | 50 |
| Ponnarmeni 2016 | 50.81 | 63 | 124 |
| Asilioglu 2017 | 48.00 | 120 | 250 |
| Badawi 2017 | 44.30 | 39 | 88 |
| Garcia-Soler 2017 | 43.82 | 149 | 340 |
| Bustos 2016 | 43.30 | 39 | 90 |
| Ebenezer 2016 | 40.38 | 21 | 52 |
| Madden 2012 | 40.12 | 205 | 511 |
| Halwany 2017 | 37.25 | 38 | 102 |
| Banajeh 2009 | 36.71 | 29 | 79 |
| Rippel 2012 | 34.49 | 109 | 316 |
| El-Gamasy 2017 | 32.50 | 26 | 80 |
| Ahmed 2014 | 30.00 | 15 | 50 |
| Rey 2014 | 29.49 | 46 | 156 |
| Inamo 2011 | 28.50 | 8 | 28 |
| Dayal 2014 | 25.00 | 23 | 92 |
| Khakshour 2015 | 24.32 | 9 | 37 |
| Lopez 2016 | 24.20 | 84 | 347 |



For peer review only - http://bmjopen.bmj.com/site/about/guidelines.xhtml

| | | | |
|---|---|---|---|
| Alonso 2015 | 15.63 | 45 | 288 |
| Ayulo 2014 | 15.63 | 61 | 216 |
| Binks 2014 | 14.86 | 11 | 74 |
| Moreno-Solis 2015 | 13.33 | 9 | 48 |
| Hurwitz 2017 | 12.22 | 11 | 90 |
| Roth 2009 | 4.69 | 3 | 64 |

**Additional Table 13 Characteristics of studies used in the meta-analysis of prevalence**

| Study | Total number of patients | Total number of vitamin D deficient patients | Country, setting | Age range | Design | Quality score (NOS) |
|---|---|---|---|---|---|---|
| Madden 2012 | 511 | 205 | Boston US, PICU | <21 years | cohort | 8 |
| Lopez 2016 | 347 | 84 | Spain, hospitalised | 0 to >48 months | cohort | 8 |
| Garcia-Soler 2017 | 340 | 149 | Spain, PICU | 6 months to 17 years | cohort | 7 |
| McNally 2012 | 326 | 225 | Canada, PICU | newborn to 17 years of age | cohort | 7 |
| Rippel 2012 | 316 | 109 | Australia, PICU | 16.5 (3.1–75.2) months | cohort | 8 |
| Alonso 2015 | 288 | 45 | Spain, PICU | 1 month to 13 years | cross sectional | 5 |
| Asilioglu 2017 | 250 | 120 | Turkey, PICUs | <= 18 years | cohort | 6 |
| Ayulo 2014 | 216 | 61 | Spain, PICU | 1 month to 13 years | cross sectional | 5 |
| Rey 2014 | 156 | 46 | Spain, PICU | <16 years | case-control | 8 |
| Shah 2016 | 154 | 128 | India,PICU | 1 month-15 years | cohort | 6 |
| Ponnarmeni 2016 | 124 | 63 | India, PICU | aged 1–12 years | case-control | 6 |
| Onwuneme 2015 (1) | 120 | 71 | Ireland, PICU | <12 years old | case-control | 8 |
| Jia 2017 | 110 | 92 | China, PICU | infants <1 years old | case-control | 7 |
| Halwany 2017 | 102 | 38 | Alexandria, PICU | >1 month to ≤ 5 years | cohort | 6 |
| Sankar 2016 | 101 | 75 | India, PICU | 1 month to 17 years | cohort | 8 |



For peer review only - http://bmjopen.bmj.com/site/about/guidelines.xhtml

| | | | | | | |
|---|---|---|---|---|---|---|
| Say 2017 | 100 | 87 | Turkey, NICU | gestational age <37 weeks | case-control | 6 |
| Sakka 2014 | 96 | 89 | Egypt, hospitalized | infants (<2 years old) | case-control | 8 |
| Onwuneme 2015 (2) | 94 | 86 | Ireland, NICU | preterm infants at <32 weeks gestation | cohort | 8 |
| Dayal 2014 | 92 | 23 | India, PICU | 3 months to 12 years | cohort | 6 |
| Bustos 2016 | 90 | 39 | Chile, PICU | >37 weeks and <15 years | cohort | 7 |
| Hurwitz 2017 | 90 | 11 | USA, hospitalised | <5 years old | cohort | 6 |
| Cayir 2014 | 88 | 50 | Turkey, PICU | 1 to 13 years | case-control | 7 |
| Badawi 2017 | 88 | 39 | Cairo, Egypt PICU | 1 month to 12 years | cohort | 7 |
| Yaghmaie 2017 | 82 | 53 | Iran, PICU | children undefined | cross sectional | 4 |
| Basha 2014 | 81 | 53 | Cairo Egypt, PICU | <5 years old | case-control | 7 |
| Prasad 2015 | 80 | 67 | India, PICU | 2 months-12 years | cohort | 7 |
| Wayse 2004 | 80 | 76 | Indapur India, PICU | <5 years | case-control | 6 |
| El-Gamasy 2017 | 80 | 26 | Egypt, PICU | 3 months to 12 years | Case-control | 7 |
| Banajeh 2009 | 79 | 29 | Iran, hospitalised | aged 2–59 months | cohort | 7 |
| Binks 2014 | 74 | 11 | Australia, PICU | <3 years old | cross sectional | 6 |
| Roth 2009 | 64 | 3 | Canada, PICU | aged 1-25 months | case-control | 7 |
| Hebbar 2014 | 61 | 37 | Atlanta, PICU | 0 to 18 years | case-control | 6 |
| Ebenezer 2016 | 52 | 21 | India, PICU | <18 years | cohort | 6 |
| Narang 2016 | 50 | 28 | Punjab, India, PICU | 2 months to 5 years | case-control | 6 |
| Alvarez 2016 | 50 | 29 | Atlanta, PICU | 0 to 18 years | cross sectional | 6 |
| Cetinkaya 2015 | 50 | 42 | Instabul/Turkey, NICU | neonates | case-control | 6 |
| Ahmed 2014 | 50 | 15 | Nigeria, PICU | 2–60 months | case-control | 8 |
| Jat 2016 | 50 | 42 | India, PICU | 1 month to 12 years | case-control | 8 |
| Sankar 2017 | 43 | 31 | India, PICU | <=17 years | cohort | 8 |
| Cizmeci 2015 | 40 | 28 | Instabul/Turkey, NICU | neonates | case-control | 7 |
| Khakshour 2015 | 37 | 9 | Iran, hospitalized | <5 years | Case-control | 5 |
| Korwutthikulrangsri 2015 | 32 | 25 | Bangkok, PICU | months not more specific range | nested case-control | 5 |
| Elmoneim 2016 | 30 | 17 | Saudi Arabia, PICU | <14 years | case-control | 5 |



For peer review only - http://bmjopen.bmj.com/site/about/guidelines.xhtml

For peer review only

| | | | | | | |
|---|---|---|---|---|---|---|
| Dinlen 2016 | 30 | 26 | Ankara Turkey, NICU | neonates | case-control | 6 |
| Moreno-Solis 2015 | 48 | 9 | Spain, PICU | infants 1–11 months | case-control | 6 |
| Inamo 2011 | 28 | 8 | Tokyo Japan, PICU | 1-48 months | cohort | 6 |
| Karatekin 2009 | 25 | 23 | Istanbul Turkey, NICU | neonates | case-control | 6 |
| Roth 2010 | 25 | 21 | Bangladesh | 1-18 months | case-control | 8 |

Studies arranged from largest to smallest total sample size. NOS = Newcastle Ottawa Score

**Additional Table 14 Sensitivity analyses for prevalence of vitamin D deficiency in all critically ill children**

| Patient category | Number of studies (Total number of individuals; number of vitamin D deficient individuals) | Pooled proportion % (95% CI) Random effects model | Pooled proportion % (95% CI) Fixed effects model | Heterogeneity ($I^2$) % (95% CI) | Q value, d.f. p-value |
|---|---|---|---|---|---|
| Critically ill children, excluding studies that used other thresholds | 36 (4,629; 2,191) | 53.1 (45.6-60.4) | 46.2 (44.6-47.8) | 95.1 (93.9-96.0) | 707.2, 35, < 0.0001 |
| Sample size >=100 (large) | 16 (3,561; 1,598) | 50.8 (40.5-61.1) | 44.3 (42.5-46.0) | 96.9 (95.9-97.6) | 481.7 15 < 0.0001 |
| Sample size <100 (small) | 32 (1,959; 1,066) | 57.2 (47.3-66.7) | 52.5 (49.9-55.1) | 92.7 (90.7-94.3) | 424.3 31 < 0.0001 |
| Cohort studies | 20 (3,399; 1,548) | 48.4 (39.7-57.3) | 44.4 (42.6-46.2) | 95.5 (94.1-96.5) | 418.6 19 < 0.0001 |
| Case-control | 24 (1,627; 978) | 64.1 (53.2-73.6) | 57.9 (55.0-60.7) | 92.8 (90.5-94.6) | 320.5 23 < 0.0001 |
| Cross sectional | 4 (494; 138) | 34.8 (12.8-66.0) | 30.3 (25.9-35.2) | 96.7 (94.0-98.2) | 90.4 3 < 0.0001 |
| Studies from India | 10 (826; 554) | 69.5 (53.0-81.5) | 64.1 (60.3-67.8) | 93.6 (90.2-95.8) | 140.2, 9, < 0.0001 |
| Studies from Turkey | 7 (583; 376) | 76.3 (60.9-87.0) | 61.0 (56.6-65.2) | 91.1 (842-95.0) | 67.5, 6, < 0.0001 |
| Studies that only recruited neonates | 6 (292; 339) | 85.6 (78.5-90.6) | 85.1 (80.7-88.7) | 54.3 (0.0-81.7) | 11.0, 5, 0.052 |
| Studies with children of all other ages except neonates | 42 (5,181; 2,372) | 49.7 (42.9-56.5) | 45.1 (43.6-46.6) | 94.7 (93.6-95.6) | 778.7 41 < 0.0001 |

CI = confidence intervals; $I^2$ = heterogeneity; df = degrees of freedom. Vitamin D deficiency defined in our study as <50 nmol/L (20 ng/mL)
$I^2$ statistic used to estimate heterogeneity between pooled studies: $I^2$ >= 75% was considered as high heterogeneity



For peer review only - http://bmjopen.bmj.com/site/about/guidelines.xhtml

**Additional Table 15 Multivariate meta-regression model for prevalence**

| Predictors | k | b-coefficient | se | t-value | p-value | ci.lb | ci.ub | F-value | I^2 (%) | R^2 (%) | QE |
|---|---|---|---|---|---|---|---|---|---|---|---|
| year+ cilinical setting+quality score+design+country group+total sample size | 48 | | | | | | | 5.111 | 95.11 | 37.52 | 642.96, p < 0.0001 |
| year | | -0.0146 | 0.0598 | -0.2442 | 0.8083 | -0.1354 | 0.1062 | | | | |
| total study sample size | | 0.0002 | 0.0017 | 0.1016 | 0.9196 | -0.0033 | 0.0037 | | | | |
| country group | | 1.3197 | 0.3853 | 3.425 | 0.0014** | 0.5415 | 2.0979 | | | | |
| clinical setting | | -2.1096 | 0.5059 | -4.17 | 0.0002*** | -3.1313 | -1.0879 | | | | |
| design group (cohort vs other) | | 0.4242 | 0.3570 | 1.1883 | 0.2415 | -0.2967 | 1.1451 | | | | |
| quality score | | 0.2066 | 0.1493 | 1.3836 | 0.174 | -0.0949 | 0.5081 | | | | |

k = number of outcomes included in the model fitting; se = standard errors of the coefficients; ci.lb = lower bound of the confidence intervals for the coefficients; ci.ub = upper bound of the confidence intervals for the coefficients; QE = test statistic for the test of (residual) heterogeneity; I^2 = residual heterogeneity / unaccounted variability; R^2 (amount of heterogeneity accounted for; PICU = pediatric intensive care units, NICU = neonatal intensive care units; *p < 0.05, **p < 0.01, ***p < 0.001

**Additional Table 16 Characteristics of studies included in the meta-analysis for prevalence in individuals with sepsis**

| Study | Total number of patients with sepsis | Total number of vitamin D deficient patients with sepsis | Country, setting | Age | Design | Quality score (NOS) |
|---|---|---|---|---|---|---|
| Shah 2016 | 160 | 135 | India, PICU | 1 month to 16 years | cohort | 6 |
| Asilioglu 2017 | 120 | 20 | Turkey, PICU | 1 month to ≤18 years | cohort | 7 |
| Say 2017 | 100 | 87 | Turkey, NICU | preterm infants at <37 weeks gestation | case-control | 6 |
| Ponnarmeni 2016 | 62 | 32 | India, PICU | 1 to 12 years | case-control | 6 |
| McNally 2012 | 48 | 33 | Canada, PICU | newborn to 17 years | cohort | 7 |
| Onwuneme 2015 (2) | 46 | 32 | Ireland, NICU | preterm infants at <32 weeks gestation | cohort | 8 |
| El-Gamasy 2017 | 46 | 20 | Egypt, pediatric emergency department | 3 months to 12 years | case-control | 7 |
| Sankar 2017 | 43 | 31 | India, PICU | <=17 years | cohort | 8 |



For peer review only - http://bmjopen.bmj.com/site/about/guidelines.xhtml

| | | | | | | |
|---|---|---|---|---|---|---|
| Cizmez 2015 | 40 | 28 | Turkey, NICU | neonates | case-control | 7 |
| Onwuneme 2015 (1) | 35 | 32 | Ireland, PICU | <12 years old | case-control | 8 |
| Hebbar 2014 | 30 | 17 | Atlanta, PICU | 0 to 18 years | case-control | 6 |
| Ebenezer 2016 | 16 | 8 | India, PICU | <18 years | cohort | 6 |
| Korwutthikulrangsri 2015 | 12 | 4 | Bangkok, PICU | moths (<8/9) | nested-case control | 5 |
| Prasad 2015 | 11 | 9 | India, PICU | 2 months to 12 years | cohort | 7 |
| Bustos 2016 | 10 | 7 | Chile, PICU | >37 weeks and < than 15 years | cohort | 7 |
| Dayal 2014 | 9 | 4 | India, tertiary care hospital | 3 months to 12 years | cohort | 6 |

For peer review only

**Additional Table 17 Sensitivity analyses for prevalence of vitamin D deficiency in critically ill children with sepsis**

| Patient category | Number of studies (Total number of individuals; number of vitamin D deficient individuals) | Pooled proportion (%, 95% CI) Random effects | Pooled proportion (%, 95% CI) Fixed effects | Heterogeneity ($I^2$) %, 95% CI | Q value, d.f. p-value |
|---|---|---|---|---|---|
| Excluding studies that used other thresholds | 12 (553; 329) | 61.4 (43.5-76.6) | 59.2 (54.3- 64.0) | 91.2 (86.5-94.2) | 125.0, 11, < 0.0001 |
| Sample size >=40 (large) | 9 (665; 418) | 63.9 (44.9-79.4) | 62.9 (58.5-67.1) | 94.3 (91.2-96.3) | 140.1, 8, < 0.0001 |
| Sample size <40 (small) | 7 (123; 81) | 63.2 (45.0-78.2) | 61.2 (51.1-70.4) | 66.2 (24.5-84.9) | 17.8, 6, 0.0068 |
| Cohort studies | 9 (463; 279) | 62.6 (40.7-80.4) | 61.8 (56.4-67.0) | 92.8 (88.6-95.5) | 111.5, 8, < 0.0001 |
| Case-control | 7 (325; 220) | 65.2 (47.3-79.7) | 63.7 (57.6-69.3) | 87.0 (75.5-93.1) | 46.3, 6, < 0.0001 |
| Studies from India | 6 (219; 301) | 66.4 (48.3-80.7) | 70.7 (64.8-75.9) | 83.6 (65.7-92.2) | 30.5, 5, < 0.0001 |
| Studies from Turkey | 3 (260; 135) | 59.2 (13.6-93.1) | 51.2 (43.2-59.3) | 97.8 (95.8- 98.8) | 90.0, 2, <0.0001 |
| Studies that only included neonates | 3 (186, 147) | 76.9 (61.9-87.3) | 77.7 (70.8-83.3) | 74.7 (15.9-92.4) | 7.9, 2, 0.019 |
| Studies that included children of all other ages except neonates | 13 (602, 352) | 60.1 (43.7-74.5) | 57.8 (53.1-62.4) | 90.8 (86.1-93.9) | 130.2, 12, < 0.0001 |

CI = confidence intervals; $I^2$ = heterogeneity; df = degrees of freedom. Vitamin D deficiency defined in our study as <50 nmol/L (20 ng/mL). $I^2$ statistic used to estimate heterogeneity between pooled studies: $I^2$ >= 75% was considered as high heterogeneity



For peer review only - http://bmjopen.bmj.com/site/about/guidelines.xhtml

**Additional Table 18 Sensitivity analyses for mortality. Pooled odds ratios for risk of mortality in deficient versus not deficient children**

| Patient category | Number of studies (Total number of individuals) | Pooled OR (95% CI) Vitamin D deficient/ Vitamin D non-deficient p-value | | Heterogeneity ($I^2$) % (95% CI) | Q value, d.f. p-value | Eggers p-value (4d.p) |
|---|---|---|---|---|---|---|
| | | Random effects | Fixed effects | | | |
| Excluding studies that used other thresholds | 14 (2,030) | 1.59 (1.05-2.41) p-value = 0.028 | 1.52 (1.08-2.13) p-value = 0.016 | 24.3 (0.0-59.9) | 17.18, 13, 0.1910 | p-value = 0.12 |
| Cohort studies only | 14 (2,197) | 1.80 (1.15-2.81) p-value = 0.009 | 1.65 (1.17-2.34) p-value = 0.004 | 31.3 (0.0-63.7) | 18.92, 13, 0.1255 | p-value = 0.0423 |
| Studies from India | 7 (646) | 1.08 (0.70-1.69) p-value = 0.710 | 1.08 (0.70-1.69) p-value = 0.710 | 0.0 (0.0-62.4) | 4.56, 6, 0.589 | Number of studies (k=5) too small to test for small study effects (k.min=10) |

CI = Confidence Intervals; $I^2$ = I squared statistic used to estimate heterogeneity (inconsistency); df = degrees of freedom, results reported in 1 decimal place; OR= odds ratio. Vitamin D deficiency defined as < 50 nmol/L or 20 ng/ml. We used the $I^2$ statistic to estimate heterogeneity between pooled studies: $I^2$ >= 75% was considered as high heterogeneity.

For peer review only

## Additional Figures

**Additional Figure 1 Funnel plot of studies of prevalence of vitamin D deficiency in critically ill children.** Horizontal axis shows logit transformed proportion and the standard error of the logit transformed proportion is plotted on the vertical axis. Each dot represents an individual study and the vertical line represents the pooled proportion from a random-effects meta-analysis. The diagonal lines represent pseudo 95% confidence limits (p = 0.015, Egger's test)

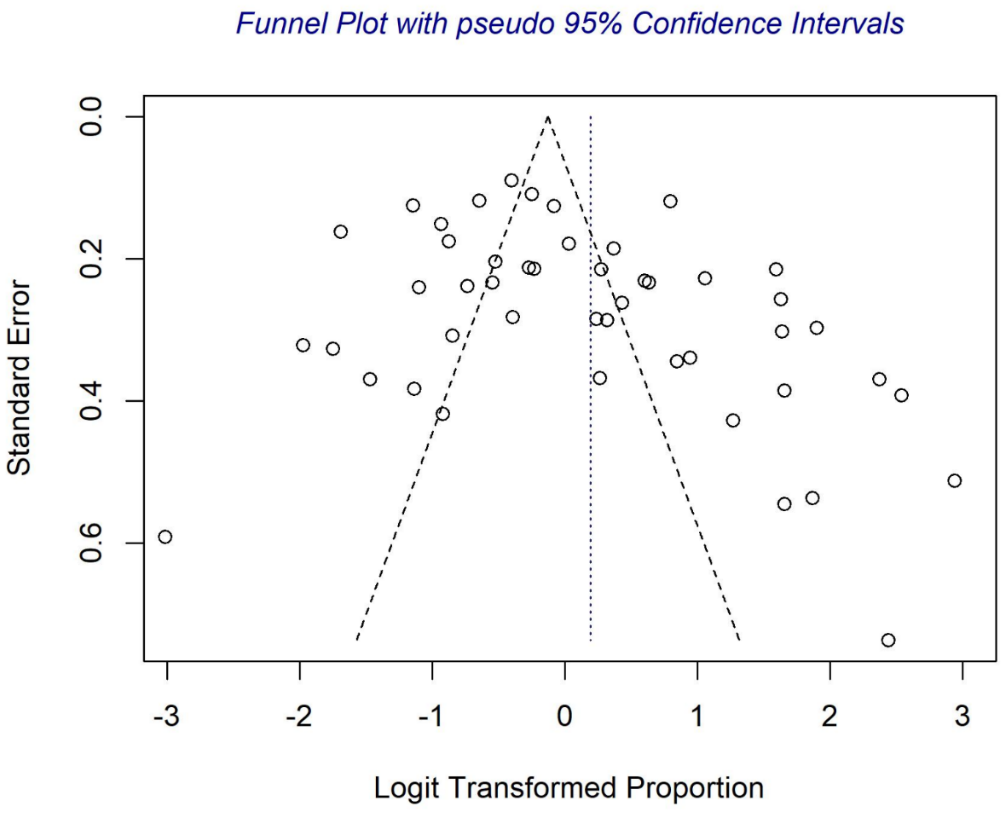




For peer review only - http://bmjopen.bmj.com/site/about/guidelines.xhtml

**Additional Figure 2. Pooled prevalence estimate for vitamin D deficiency in critically ill children (by country group).** Forest plot shows results from the random effects model. Diamonds represent pooled proportion of vitamin D deficiency for the countries in each subgroup (group 1, group2, group 3). The diamond at the bottom shows the overall pooled estimate of all the 48 studies. Each square shows the prevalence estimate of each study and the horizontal line across each square represents the 95% confidence interval (CI) of the prevalence estimate.

| Study | VD deficient (n) | Total (N) | Prevalence | 95% CI | Weight |
|---|---|---|---|---|---|
| **group1** | | | | | |
| Madden 2012 | 205 | 511 | 0.40 | [0.36; 0.45] | 2.3% |
| Lopez 2016 | 84 | 347 | 0.24 | [0.20; 0.29] | 2.3% |
| Garcia-Soler 2017 | 149 | 340 | 0.44 | [0.38; 0.49] | 2.3% |
| McNally 2012 | 225 | 326 | 0.69 | [0.64; 0.74] | 2.3% |
| Rippel 2012 | 109 | 316 | 0.34 | [0.29; 0.40] | 2.3% |
| Alonso 2015 | 45 | 288 | 0.16 | [0.12; 0.20] | 2.2% |
| Ayulo 2014 | 61 | 216 | 0.28 | [0.22; 0.35] | 2.3% |
| Rey 2014 | 46 | 156 | 0.29 | [0.22; 0.37] | 2.2% |
| Onwuneme 2015 (1) | 71 | 120 | 0.59 | [0.50; 0.68] | 2.2% |
| Onwuneme2015 (2) | 86 | 94 | 0.91 | [0.84; 0.96] | 2.0% |
| Bustos 2016 | 39 | 90 | 0.43 | [0.33; 0.54] | 2.2% |
| Hurwitz 2017 | 11 | 90 | 0.12 | [0.06; 0.21] | 2.1% |
| Binks 2014 | 11 | 74 | 0.15 | [0.08; 0.25] | 2.1% |
| Roth 2009 | 3 | 64 | 0.05 | [0.01; 0.13] | 1.6% |
| Hebbar 2014 | 37 | 61 | 0.61 | [0.47; 0.73] | 2.1% |
| Alvarez 2016 | 29 | 50 | 0.58 | [0.43; 0.72] | 2.1% |
| Moreno-Solis 2015 | 9 | 48 | 0.19 | [0.09; 0.33] | 2.0% |
| Inamo 2011 | 8 | 28 | 0.29 | [0.13; 0.49] | 1.9% |
| **Random effects model** | | **3219** | **0.36** | **[0.28; 0.45]** | **38.5%** |
| Heterogeneity: $I^2 = 95.6\%$, $\tau^2 = 0.6310$, $p < 0.01$ | | | | | |
| **group2** | | | | | |
| Asilioglu 2017 | 120 | 250 | 0.48 | [0.42; 0.54] | 2.3% |
| Jia 2017 | 92 | 110 | 0.84 | [0.75; 0.90] | 2.1% |
| Halwany 2017 | 38 | 102 | 0.37 | [0.28; 0.47] | 2.2% |
| Say 2017 | 87 | 100 | 0.87 | [0.79; 0.93] | 2.1% |
| Sakka 2014 | 89 | 96 | 0.93 | [0.86; 0.97] | 2.0% |
| Cayir 2014 | 50 | 88 | 0.57 | [0.46; 0.67] | 2.2% |
| Badawi 2017 | 39 | 88 | 0.44 | [0.34; 0.55] | 2.2% |
| Yaghmaie 2017 | 53 | 82 | 0.65 | [0.53; 0.75] | 2.2% |
| Basha 2014 | 53 | 81 | 0.65 | [0.54; 0.76] | 2.2% |
| El-Gamasy 2017 | 26 | 80 | 0.32 | [0.22; 0.44] | 2.2% |
| Banajeh 2009 | 29 | 79 | 0.37 | [0.26; 0.48] | 2.2% |
| Cetinkaya 2015 | 42 | 50 | 0.84 | [0.71; 0.93] | 2.0% |
| Ahmed 2014 | 15 | 50 | 0.30 | [0.18; 0.45] | 2.1% |
| Cizmeci 2015 | 28 | 40 | 0.70 | [0.53; 0.83] | 2.0% |
| Khakshour 2015 | 9 | 37 | 0.24 | [0.12; 0.41] | 2.0% |
| Elmoneim 2016 | 17 | 30 | 0.57 | [0.37; 0.75] | 2.0% |
| Dinlen 2016 | 26 | 30 | 0.87 | [0.69; 0.96] | 1.7% |
| Karatekin 2009 | 23 | 25 | 0.92 | [0.74; 0.99] | 1.4% |
| **Random effects model** | | **1418** | **0.63** | **[0.52; 0.72]** | **37.0%** |
| Heterogeneity: $I^2 = 92\%$, $\tau^2 = 0.7717$, $p < 0.01$ | | | | | |
| **group3** | | | | | |
| Shah 2016 | 128 | 154 | 0.83 | [0.76; 0.89] | 2.2% |
| Ponnarmeni 2016 | 63 | 124 | 0.51 | [0.42; 0.60] | 2.2% |
| Sankar 2016 | 75 | 101 | 0.74 | [0.65; 0.82] | 2.2% |
| Dayal 2014 | 23 | 92 | 0.25 | [0.17; 0.35] | 2.2% |
| Prasad 2015 | 67 | 80 | 0.84 | [0.74; 0.91] | 2.1% |
| Wayse 2004 | 76 | 80 | 0.95 | [0.88; 0.99] | 1.8% |
| Ebenezer 2016 | 21 | 52 | 0.40 | [0.27; 0.55] | 2.1% |
| Narang 2016 | 28 | 50 | 0.56 | [0.41; 0.70] | 2.1% |
| Jat 2016 | 42 | 50 | 0.84 | [0.71; 0.93] | 2.0% |
| Sankar 2017 | 31 | 43 | 0.72 | [0.56; 0.85] | 2.0% |
| Korwutthikulrangsri 2015 | 25 | 32 | 0.78 | [0.60; 0.91] | 1.9% |
| Roth 2010 | 21 | 25 | 0.84 | [0.64; 0.95] | 1.7% |
| **Random effects model** | | **883** | **0.71** | **[0.58; 0.82]** | **24.5%** |
| Heterogeneity: $I^2 = 92.5\%$, $\tau^2 = 1.0012$, $p < 0.01$ | | | | | |
| **Random effects model** | | **5520** | **0.55** | **[0.48; 0.62]** | **100.0%** |
| Heterogeneity: $I^2 = 95.0\%$, $\tau^2 = 0.8587$, $p < 0.01$ | | | | | |

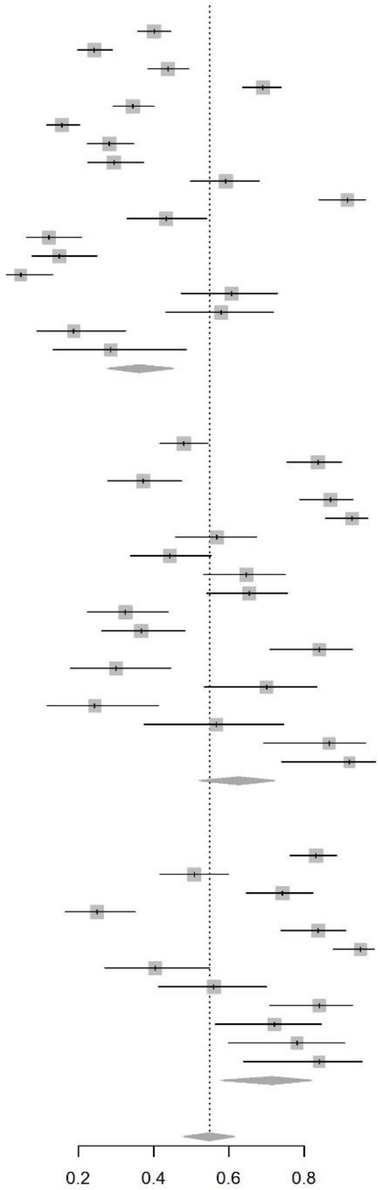




For peer review only - http://bmjopen.bmj.com/site/about/guidelines.xhtml

**Additional Figure 3. Pooled prevalence estimates for vitamin D deficiency in critically ill children (neonates versus all other age groups).** Forest plot shows results from the random effects model. Diamonds represent pooled proportion of vitamin D deficiency for the studies in neonates and all other age groups. The diamond at the bottom shows the overall pooled estimate of all the 48 studies. Each square shows the prevalence estimate of each study and the horizontal line across each square represents the 95% confidence interval (CI) of the prevalence estimate.

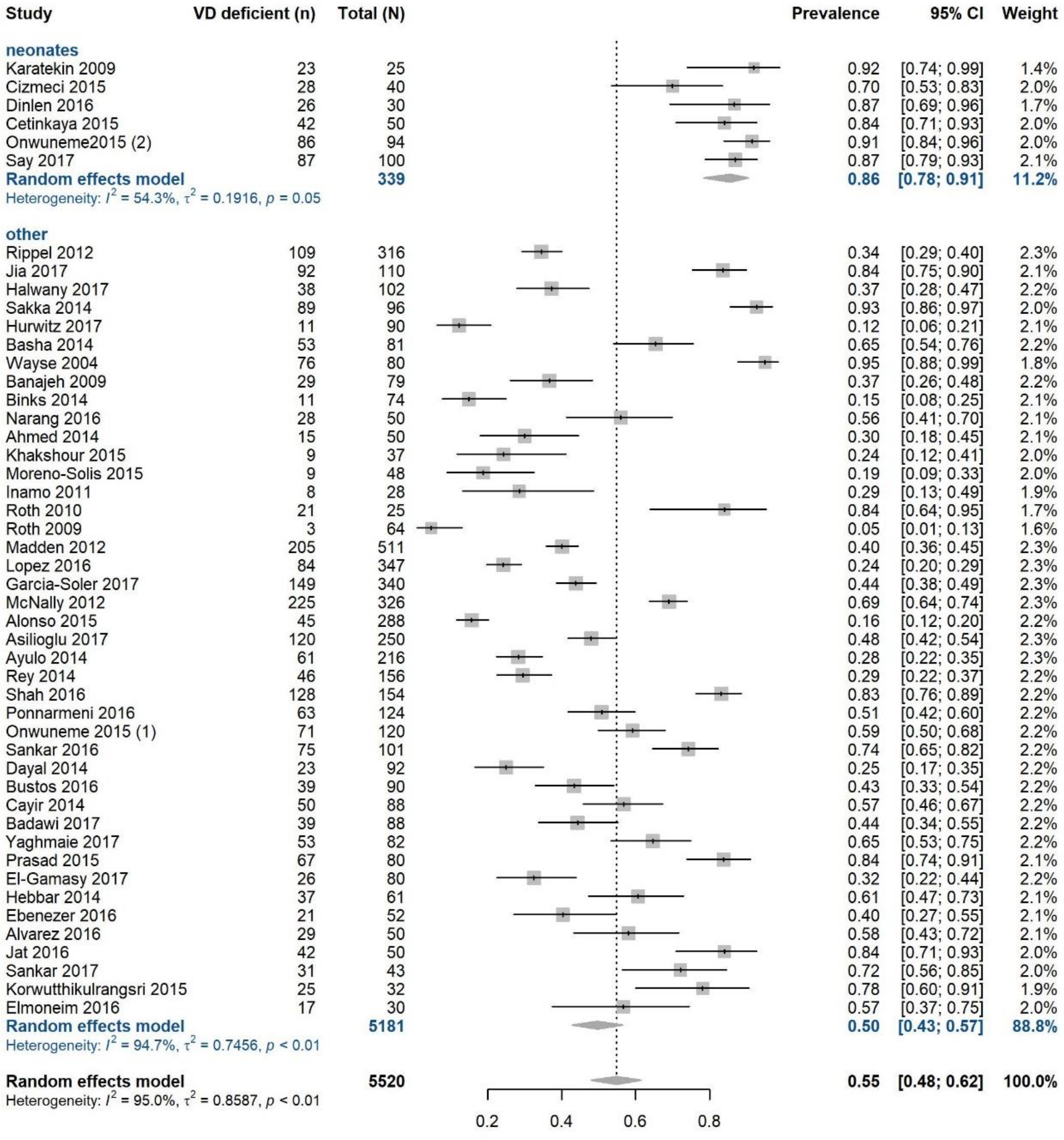

**Additional Figure 4 Bubble plots of univariate meta-regressions.** Each study is represented by a circle. Predictor variables: A study design, B year of publication, C quality score and D total children are plotted on the x-axis with the effect measure "logit transformed proportion" shown on the vertical (y-axis).

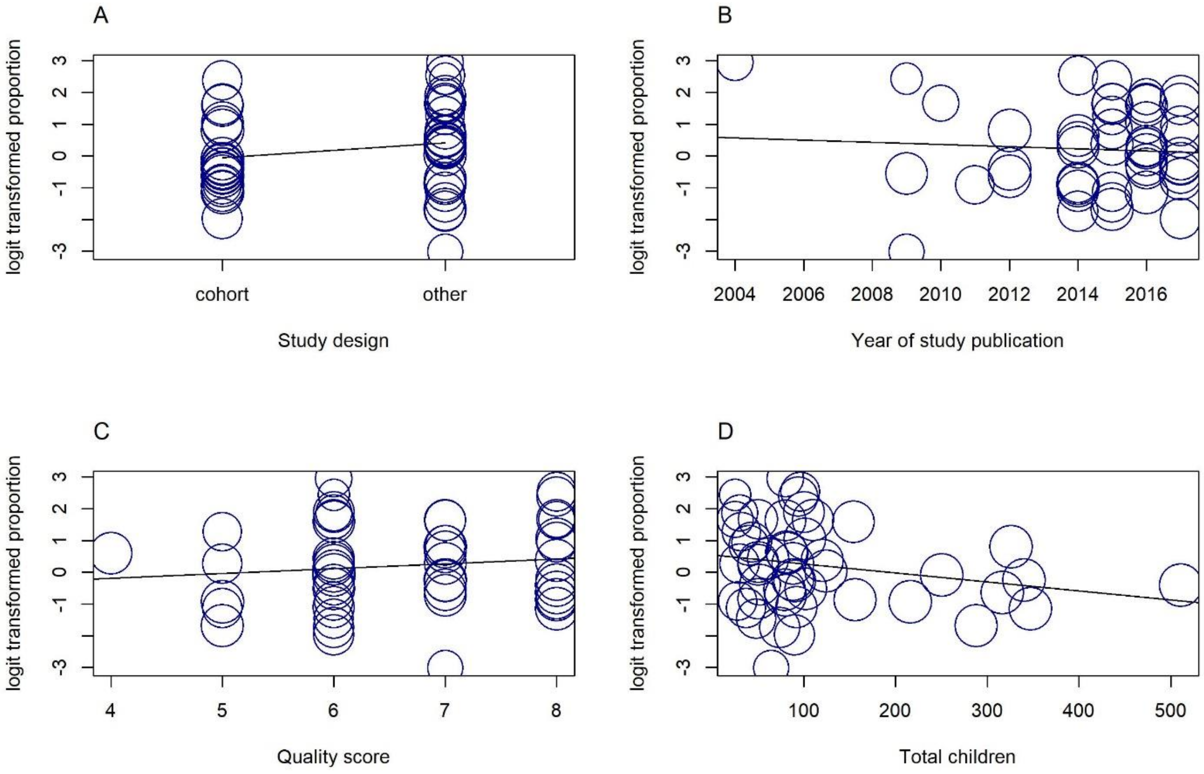




For peer review only - http://bmjopen.bmj.com/site/about/guidelines.xhtml

**Additional Figure 5 Funnel plot for prevalence of vitamin D deficiency in critically ill children with sepsis.** Horizontal axis shows logit transformed proportion and the standard error of the logit transformed proportion is plotted on the vertical axis. Each dot represents an individual study and the vertical line represents the pooled proportion from a random-effects meta-analysis. The diagonal lines represent pseudo 95% confidence limits (p = 0.828, Egger's test).

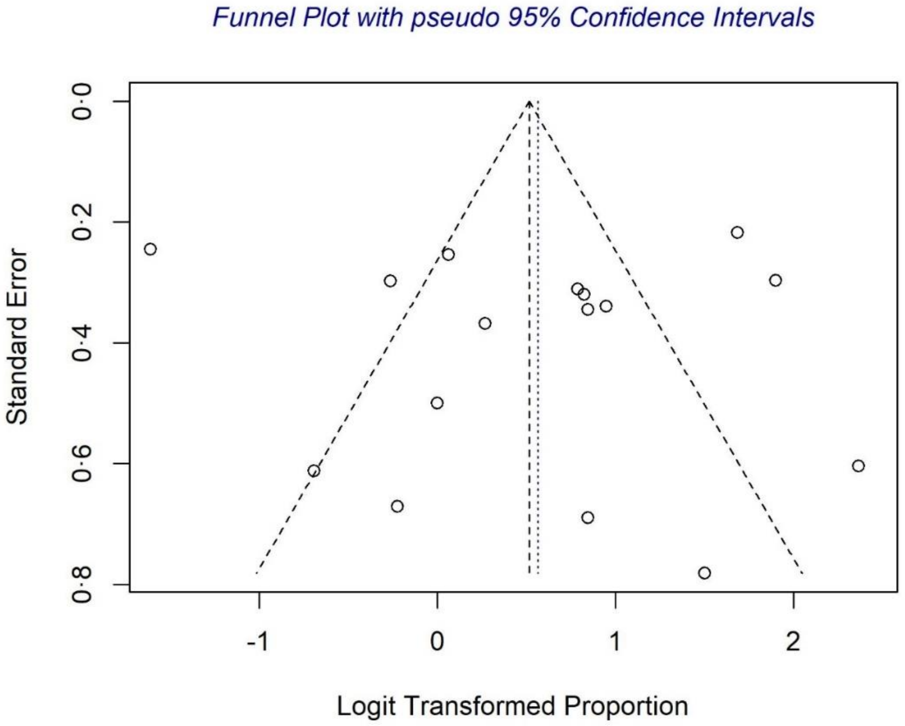


For peer review only

**Additional Figure 6 Pooled prevalence estimate for vitamin D deficiency in critically ill children with sepsis (subgroup analysis by study design).** Forest plot shows results from the random effects model. Diamonds represent the pooled proportion of vitamin D deficiency for the studies in each subgroup (case-control and cohort). The diamond at the bottom shows the overall pooled estimate of all the 16 studies. Each square shows the prevalence estimate of each study and the horizontal line across each square represents the 95% confidence interval (CI) of the prevalence estimate.

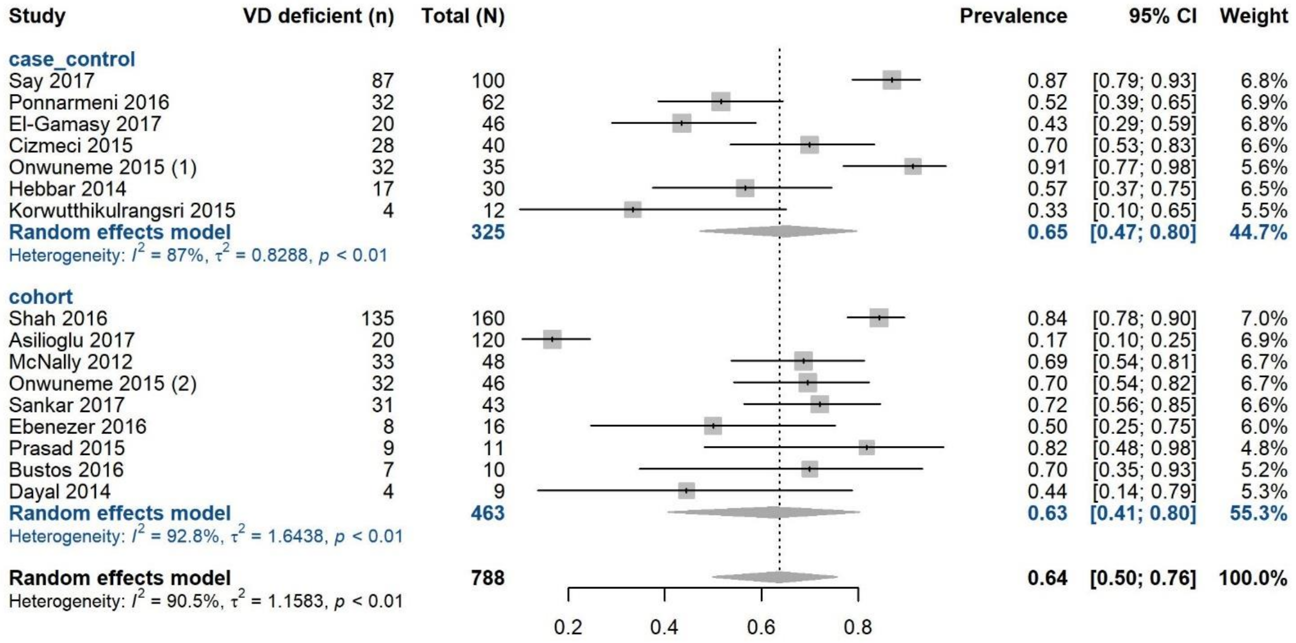




For peer review only - http://bmjopen.bmj.com/site/about/guidelines.xhtml

**Additional Figure 7 Funnel plot of risk of mortality in vitamin D deficient versus vitamin D non-deficient critically ill children**. Horizontal axis shows logit transformed odds ratio and the standard error of the log odds ratio is plotted on the vertical axis. Each dot represents an individual study and the vertical line represents the overall pooled odds ratio from random-effects meta-analysis. The diagonal lines represent pseudo 95% confidence limits (p = 0.084, Egger's test).

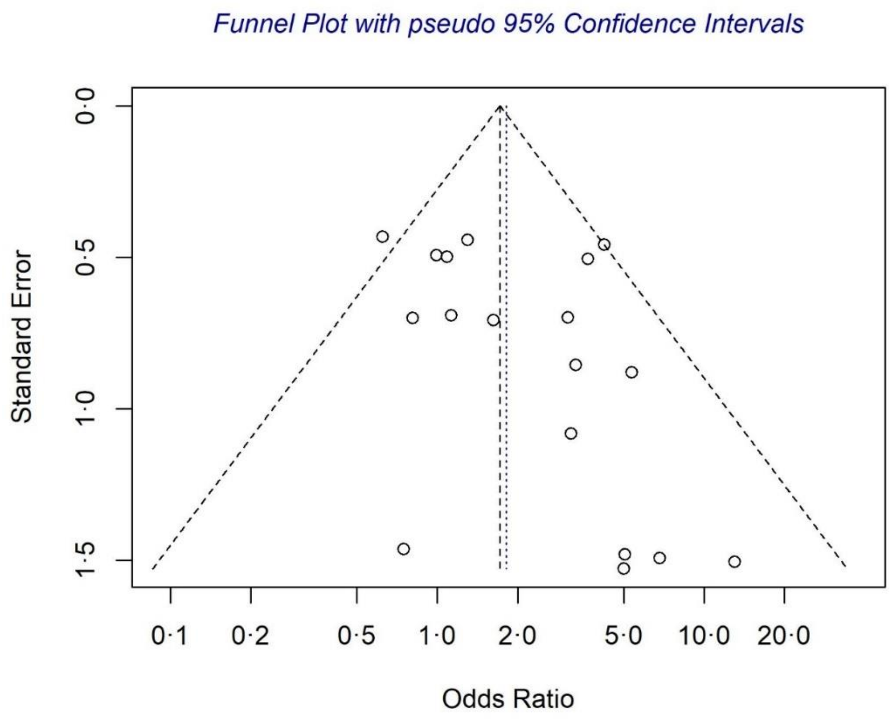


only



For peer review only - http://bmjopen.bmj.com/site/about/guidelines.xhtml

**Additional Figure 8 Pooled odds ratio (OR) and 95% CI for risk of mortality in vitamin D deficient versus vitamin D non-deficient critically ill children (fixed effects model)**. Diamond represents the overall odds ratio (with corresponding 95% Confidence Interval). Each square shows the odds ratio of each study and the horizontal line across each square represents the 95% confidence interval (CI) of the estimate.

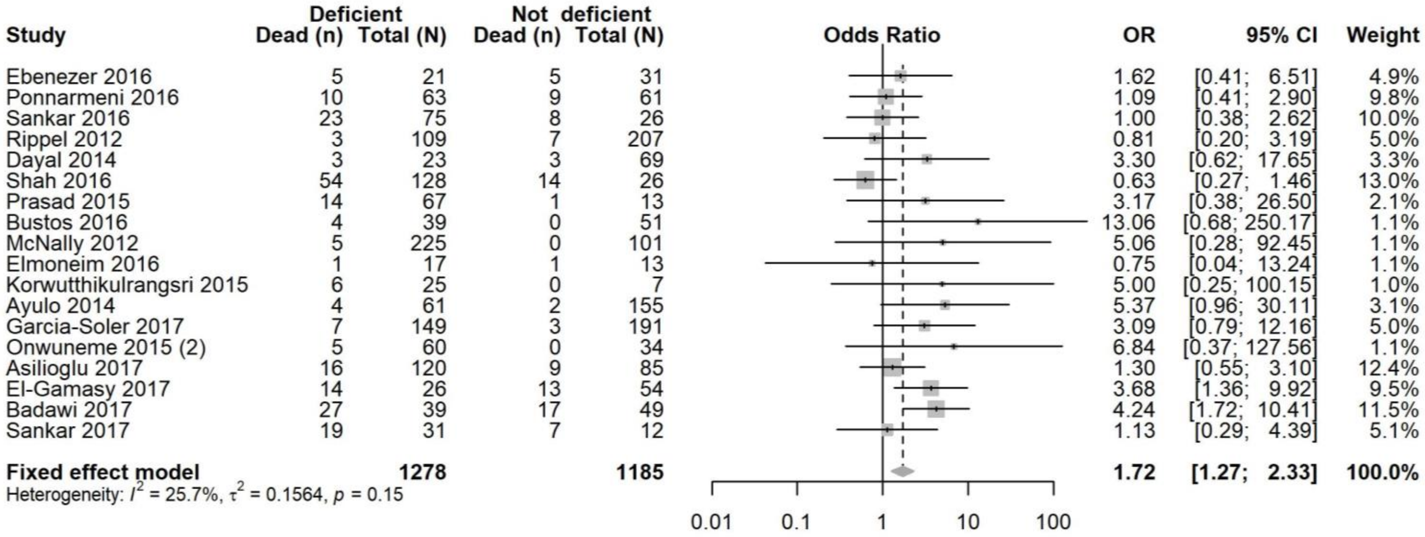


For peer review only

BMJ Open

# Importance of vitamin D in acute and critically ill children with subgroup analyses of sepsis and respiratory tract infections: a systematic review and meta-analysis

| | |
|---|---|
| Journal: | *BMJ Open* |
| Manuscript ID | bmjopen-2018-027666.R1 |
| Article Type: | Research |
| Date Submitted by the Author: | 18-Jan-2019 |
| Complete List of Authors: | Cariolou, Margarita; Imperial College London School of Public Health, Epidemiology and Biostatistics<br>Cupp, Meghan; Imperial College London School of Public Health, Epidemiology and Biostatistics<br>Evangelou, Evangelos; Imperial College London School of Public Health, Epidemiology and Biostatistics; University of Ioannina School of Medicine, Department of Hygiene and Epidemiology<br>Tzoulaki, Ioanna; Imperial College London, Epidemiology and Public Health; University of Ioannina School of Medicine, Department of Hygiene and Epidemiology<br>Berlanga-Taylor, Antonio; Imperial College London School of Public Health, Epidemiology and Biostatistics |
| <b>Primary Subject Heading</b>: | Epidemiology |
| Secondary Subject Heading: | Paediatrics, Research methods |
| Keywords: | Vitamin D, EPIDEMIOLOGY, meta-analysis, sepsis, PAEDIATRICS, INTENSIVE & CRITICAL CARE |
| | |

SCHOLARONE™
Manuscripts

**Importance of vitamin D in acute and critically ill children with subgroup analyses of sepsis and respiratory tract infections: a systematic review and meta-analysis**

Margarita Cariolou[1] MPH, Meghan A. Cupp[1] MPH, Evangelos Evangelou[1,2] PhD, Ioanna Tzoulaki[1,2] PhD and Antonio J. Berlanga-Taylor[1]* DPhil

[1]MRC-PHE Centre for Environment and Health, Department of Epidemiology & Biostatistics, School of Public Health, Faculty of Medicine, Imperial College London, St Mary's Campus, Norfolk Place, UK, LONDON W2 1PG

[2]Department of Hygiene and Epidemiology, University of Ioannina Medical School, Ioannina, Greece

*Correspondence should be addressed to Dr. Antonio J. Berlanga-Taylor (a.berlanga@imperial.ac.uk), MRC-PHE Centre for Environment and Health, Department of Epidemiology & Biostatistics, School of Public Health, Faculty of Medicine, Imperial College London, St Mary's Campus, Norfolk Place, LONDON W2 1PG.



For peer review only - http://bmjopen.bmj.com/site/about/guidelines.xhtml

For peer review only

## ABSTRACT

**Objectives:** To estimate the prevalence of 25-hydroxyvitamin D (25(OH)D) deficiency and investigate its association with mortality in children with acute or critical conditions.

**Design:** Systematic review and meta-analysis of observational studies.

**Data sources:** PubMed, OVID, Google Scholar and the Cochrane Library searched until 21 December 2018.

**Eligibility criteria:** Studies of children hospitalised with acute or critical conditions who had blood 25(OH)D levels measured.

**Data extraction and synthesis:** We obtained pooled prevalence estimates of 25(OH)D deficiency and odds ratios for mortality. We calculated 95% confidence and prediction intervals and investigated heterogeneity and evidence of small-study effects.

**Results:** Fifty-two studies were included. Of 7,434 children, 3,473 (47.0%) were 25(OH)D deficient (<50 nmol/L). The pooled prevalence estimate of 25(OH)D deficiency was 54.6% (95% CI 48.5-60.6, $I^2$=95.3%, p<0.0001). Prevalence was similar after excluding smaller studies (51.5%). In children with sepsis (18 studies, 889 total individuals) prevalence was 64.0% (95% CI 52.0-74.4, $I^2$=89.3%, p<0.0001) and 48.7% (95% CI 38.2-59.3; $I^2$=94.3%, p<0.0001) in those with respiratory tract infections (RTI) (25 studies, 2,699 total individuals). Overall, meta-analysis of mortality (18 studies, 2,463 total individuals) showed increased risk of death in 25(OH)D deficient children (OR 1.81, 95% CI 1.24-2.64, p=0.002, $I^2$=25.7%, p=0.153). A cohort-only sensitivity analysis agreed with the overall observation (n=14, OR 1.80, 95% CI 1.15-2.81, p=0.009, $I^2$=31.3%, p=0.126) but showed small-study effects (Egger's test p=0.042). Four (22.0%) of 18




For peer review only - http://bmjopen.bmj.com/site/about/guidelines.xhtml

studies statistically adjusted for confounders. There were insufficient studies to meta-analyse sepsis and RTI related mortality.

**Conclusions:** Our results suggest that 25(OH)D deficiency in acute and critically ill children is high and associated with increased mortality. Small-study effects, reverse causation and other biases may have confounded results. Larger, carefully designed studies in homogeneous populations with confounder adjustment are needed to clarify the association between 25(OH)D levels with mortality and other outcomes.

**Registration** PROSPERO (CRD42016050638)

**Copyright** Open access article under terms of CC BY

**Keywords** paediatric, vitamin D, intensive care, sepsis, meta-analysis, prevalence, mortality, systematic review, respiratory tract infections

**Strengths and limitations of this study**

- We comprehensively assessed the magnitude and relevance of vitamin D (25(OH)D) circulating levels in paediatric acute and critically ill patients using a large number of studies with large total sample size with pre-specified sub-group and sensitivity analyses.
- We used PRISMA and MOOSE guidelines for reporting.
- We used the currently recommended cut-off of less than 50 nmol/L for vitamin D deficiency.
- We did not find enough studies to perform meta-analyses for mortality from sepsis or respiratory tract infection in relation to vitamin D status.
- We did not identify longitudinal studies with multiple time-point, pre-admission or pre-disease vitamin D measurements.
- Most studies were single centre with heterogeneous patient groups and few controlled for important confounders that influence vitamin D levels such as age, BMI, gender, season of measurements, vitamin D supplementation and comorbidities.



For peer review only - http://bmjopen.bmj.com/site/about/guidelines.xhtml

## INTRODUCTION

Vitamin D is an essential nutrient[1, 2] representing a group of fat soluble secosteroids with key endocrine functions.[3] It is synthesized in the skin upon sunlight exposure[4] while dietary sources, such as oily fish, egg yolk, certain fungi and supplements, are usually secondary sources. Vitamin D is critical in bone metabolism[5] and calcium homeostasis,[6] as well as acting as an important regulator in extra-skeletal metabolic processes,[7] cardiovascular and immune systems.[8] Many observational and laboratory studies have observed the anti-inflammatory properties of vitamin D,[9] including direct regulation of endogenous anti-microbial peptide production.[10]

It is therefore crucial for humans to have sufficient vitamin D levels to maintain bone health and possibly improve response to infection.[6, 11, 12] Infants and children are especially dependent on vitamin D to achieve healthy bone development and growth.[13, 14] Well-known functional outcomes of adequate vitamin D levels in children include rickets prevention, higher bone mineral content and reduced bone fracture rates.[5, 14] In otherwise healthy children in the United States, the reported prevalence of vitamin D deficiency (25OHD levels of < 25 nmol/L) ranges from 9 to 18%.[15] The Endocrine Society Clinical Practice Guidelines and the Institute of Medicine (IOM) suggest that 25(OH)D levels less than 50 nmol/L (20 ng/mL) reflect a deficient state.[4, 16]

Studies in adults reflect a high prevalence of vitamin D deficiency both in general intensive care unit (ICU) and sepsis patients and strongly suggest an association between low vitamin D and poor clinical outcomes, including increased mortality, particularly in those suffering from sepsis.[2, 17] Recent clinical trials of vitamin D supplementation in adults appear promising in both general critical care[18, 19] and sepsis.[20]



For peer review only - http://bmjopen.bmj.com/site/about/guidelines.xhtml

Sepsis remains a challenging clinical entity with high social and economic costs.[21] Each year there are approximately 123,000 sepsis cases and around 37,000 deaths in England alone.[22] Recent reports show an increased prevalence of paediatric sepsis,[23] likely a reflection of an increased population with chronic comorbidities, higher rates of opportunistic infections and multi-drug resistant organisms.[24] Respiratory tract infections account for a large proportion of underlying diagnoses in acute and critical care conditions[24, 25] but remain understudied.[26]

The magnitude, relevance and quality of evidence of vitamin D deficiency in children receiving acute care is not clear. Several recent studies have addressed these questions with mixed results. We sought to summarise the evidence regarding the implications of vitamin D deficiency and its prevalence in general acute care, ICU, respiratory tract infection and sepsis patients in the paediatric population. We carried out a systematic review and meta-analysis of circulating vitamin D levels, as measured by 25(OH)D, to assess the prevalence of vitamin D deficiency (≤ 50 nmol/L) and its association with mortality in these conditions.

## METHODS

We planned and conducted our systematic review and meta-analysis according to the PRISMA guidelines[27] (*Additional Table 1*). We also followed the Meta-Analysis of Observational Studies in Epidemiology (MOOSE) guidelines[28] as no relevant randomized controlled trials have been reported.

### Search strategy and selection criteria



For peer review only - http://bmjopen.bmj.com/site/about/guidelines.xhtml

Our population of interest consists of paediatric patients with acute conditions and/or those treated in ICU or emergency units for acute conditions whose vitamin D status was assessed prior to or during admission. We included published cross sectional, case-control and cohort studies that measured circulating 25(OH)D levels and either reported prevalence, odds ratios (OR) or data to enable calculation of these measures. Studies were excluded if they were reviews, case reports, surveys, commentaries, replies, not original contributions, experimental *in vitro* or if they recruited patients who were not treated in emergency, neonatal intensive care units (NICUs), paediatric intensive care units (PICUs) or for acute conditions. Studies were also excluded if they only enrolled vitamin D deficient patients, investigated healthy populations only or did not measure circulating 25(OH)D levels as an indicator of vitamin D status. When we identified more than one publication utilising the same cohort, we included the publication which shared our review's objective to investigate vitamin D levels and prevalence of deficiency.

For purposes of our review, we classified vitamin D deficiency as being 25(OH)D less than 50 nmol/L (equivalent to 20 ng/mL), as suggested by the IOM.[16] Different age categories were used to designate patients as "children" in the studies reviewed. We therefore included all "children" (neonates up to 21 years) as defined by each treating facility and this included "neonates", "infants", "toddlers", "children" and "adolescents".

We searched PubMed, OVID, Google Scholar and the Cochrane Library from inception up until 21$^{st}$ December 2018, with no language restrictions. Search terms used across these databases included: "critical care", "vitamin D", "pediatric", "child", "neonate", "toddler", "intensive care unit", "sepsis" and "septic shock". Search terms used in OVID and PubMed are listed in the *Additional Tables 2A and 2B*. Literature searches were performed by two investigators independently (MC and AJBT) and included initial screening of titles and abstracts, followed by



For peer review only - http://bmjopen.bmj.com/site/about/guidelines.xhtml

full text screening. Any disagreements for study eligibility were resolved by discussion between the two investigators. Reference lists of the selected papers, including reviews, were also checked for relevant titles. Abstracts of relevant titles were then assessed for eligibility. Corresponding authors were contacted to obtain additional information if necessary. A data extraction form was designed a priori in Excel. Variables extracted from each study included year of publication, country of study, clinical setting, cut-off given to define vitamin D deficiency, total number of children, total number of cases, study design and age range.

**Study quality assessment**

The quality of each included study was assessed using the Newcastle-Ottawa Scale (NOS) for cohort, case-control and cross-sectional study designs (*Additional Tables 3A, 3B* and *3C*).[29] We classified studies as low (1-3), medium (4-6) or high quality (7-9) for purposes of sensitivity analysis.

**Prevalence and mortality outcomes**

In the majority of studies (n = 40), prevalence of vitamin D deficiency was extracted as reported with a threshold of ≤ 50 nmol/L. If prevalence was not reported directly, it was calculated using data provided in each study (cases ≤ 50 nmol/L / total number of study participants) (*Additional Table 4A* and *4B*). Extracted or calculated prevalence values were then combined in a meta-analysis. For mortality, we calculated unadjusted odd ratios (OR) as:

OR = (vitamin D deficient patients who died * vitamin D non-deficient patients who did not die)/ (vitamin D deficient patients who did not die * vitamin D non-deficient patients who died)



For peer review only - http://bmjopen.bmj.com/site/about/guidelines.xhtml

We had sufficient information to calculate ORs < 50 nmol/L for 40 studies (77.0%). For the 12 studies with insufficient information, we used the lower cut-off values reported as a conservative approximation (*Additional Table 5*). We converted 25(OH)D values using: nmol/L = ng/mL * 2.496.

**Data analysis**

We obtained proportions of vitamin D deficiency with 95% confidence intervals (CI) using the Clopper-Pearson method[30] in R. We used a random effects model[31] to account for the variation observed within and between studies due to the different ages and acute conditions in the populations considered. For each meta-analysis we also obtained the 95% prediction interval (PI) to further account for between study heterogeneity. This helps to evaluate how consistent an observed effect would be in a future study that will investigate the same association.[32] We obtained pooled proportions and pooled ORs with fixed effect model for sensitivity analysis or in cases where heterogeneity was low.[33-35] For prevalence we also calculated median and interquartile range (IQR) for comparisons with pooled prevalence estimates.

We investigated possible sources of heterogeneity using sensitivity and subgroup analyses. Cochran's Q was used to assess the heterogeneity and the $I^2$ statistic was used to estimate the percentage of total variation across studies which can be attributed to heterogeneity. Confidence intervals of $I^2$ were calculated to aid interpretation.[36] A Q value of < 0.05 was considered significant and an $I^2$ statistic greater or equal to 75% indicated a high level of variation due to heterogeneity.[37, 38] We used Egger's regression test to present results of small-study effects and funnel plot asymmetry[39] and generated funnel plots for visual assessment and screening. A p-value < 0.05 indicated evidence of small-study effects. With few studies, Egger's test has low power to


For peer review only - http://bmjopen.bmj.com/site/about/guidelines.xhtml

detect such bias, therefore we only estimated small-study effects for analyses with more than ten studies.[40] When small-study effects were detected based on this threshold, we used trim-and-fill methods to add potentially missed studies and re-calculate an adjusted pooled estimate.[41]

To further assess heterogeneity, we utilised meta-regression to identify predictor variables that could explain variation in study prevalence estimates. We used restricted maximum likelihood (REML) estimations in the model to account for residual heterogeneity[42] and the Knapp-Hartung method to adjust confidence intervals and test statistics. This method estimates between study variance using a t-distribution, rather than a z-distribution, yielding a more conservative inference.[43] We tested the following continuous predictors: year of study publication, total sample size and quality score. Categorical variables included study setting (PICU, NICU), study design (case-control, cross-sectional and cohort) and country group by geographic region and economic development (group 1, group 2, and group 3) and were dummy coded.

We used R version 3.5.0 and Microsoft Excel 2010 for analyses and data collection. The R packages "meta"[44] and "metafor"[45] were used for analyses. Only results of the random effects model are reported for prevalence due to the expected heterogeneity between populations being considered. Our protocol is registered in PROSPERO (CRD42016050638).

**Role of the funding source**

The study received funding from the UK Medical Research Council. The funders had no role in data collection, analysis, interpretation or writing of the report. All authors had access to the data in the study.

**Patient involvement**



For peer review only - http://bmjopen.bmj.com/site/about/guidelines.xhtml

No patients were involved in this study. We only used data from previously published studies.

## RESULTS

### Screening and study characteristics

After title and abstract screening, we identified 2,890 potentially relevant studies (*Figure 1*) and eighty-five full text articles were assessed for eligibility. Rationale for study exclusion included: studies including adults, study populations other than critically ill children or with acute conditions, studies of circulating vitamin D levels and deficiency in healthy children or in children with chronic conditions. Four studies[46-49] were excluded due to insufficient data reporting (*Additional Table 6*). We also excluded three studies[50-52] that used the same cohort of children and included a single study to represent the cohort.[53] Ultimately, 52 studies met criteria for inclusion (*Additional Table 7*).

*Figure 1 Flow chart of study selection process*

The primary objective of most included studies was to determine circulating vitamin D concentration ("status") in children and/or prevalence of vitamin D deficiency. Secondary objectives included investigation of associations between deficiency of circulating vitamin D and various outcomes, such as hospital mortality length of stay, requirement of ventilation and/or illness severity (*Additional Table 8*).

All included studies reported vitamin D measurement assay methods used (*Additional Table 9*) and stated that samples were collected and analysed within the first 24 hours of hospital admission. Studies reported ethical approval and consent for participation from parents or guardians (*Additional Table 10*). Included studies were published between 2004 and 2018, with the majority



For peer review only - http://bmjopen.bmj.com/site/about/guidelines.xhtml

(n = 40, 77.0%) published between 2014 and 2017 (*Additional Table 7*). In total, 7,434 children were hospitalized in paediatric or neonatal intensive care units or emergency units or for acute conditions. Sample sizes of critically ill children ranged from 25[54] to 1,016.[55] In 18 studies the total number of cases was greater than 100.

Studies originated from 15 countries, with the majority from India[8, 56-65] (n = 11) or Turkey[54, 66-71] (n = 7) (*Additional Table 7*). All were of medium or high quality (NOS score median 6.5, range 4-8). The score range for cohort studies was 6 to 8 (n = 22), for case-control studies 5 to 8 (n = 26) and for cross sectional 4 to 6 (n = 4). Studies used a broad range of ages to classify patients as "children". Seven studies (13.5%)[54, 65, 67, 69-72] included only neonates. In two[67, 72] of these studies, neonates were preterm. The largest age range was seen in the study of Ayulo et al 2014, which included individuals between 1 and 21 years of age (*Additional Table 11*). Forty-two of the included studies (80.8%) included patients admitted for medical conditions and the other ten[53, 61, 66, 73-78] included both surgical and medical patients. Of the 52 included studies 26 used a control group and had a total number of 2,479 controls of which 773 (31.2%) were vitamin D deficient.

All studies included both female and male participants. For mortality, four of the 18 studies (22.0%) carried out multivariate regression analysis with adjustment for confounders. The remaining studies presented results using a variety of methods, including Spearman's correlation analysis, chi-square or Fisher's exact tests or descriptive statistics.

**Prevalence of vitamin D deficiency**

We included 52 studies representing a total of 7,434 children hospitalised with critical or acute conditions. Of these, 3,473 (47.0%) were classified as vitamin D deficient (< 50 nmol/L).



For peer review only - http://bmjopen.bmj.com/site/about/guidelines.xhtml

Prevalence of deficiency ranged from 5.0%[79] to 95.0%[60] , median (IQR) 56.3% (31.9 to 75.2%) (*Additional Table 12*). Sample sizes ranged from 25 to 1,016, with a median of 82 individuals (*Additional Table 13*). Using a random effects model, the pooled prevalence estimate of vitamin D deficiency was 54.6% (95% CI 48.5-60.6) with a high proportion of variation attributed to heterogeneity ($I^2$ = 95.3%, 95% CI 94.5-96.0, $p < 0.0001$) (*Figure 2*) and evidence of funnel plot asymmetry (p = 0.01, Egger's test) (*Table 1* and *Additional Figure 1*). Trim and fill analysis estimated 11 unpublished studies in the lower left-hand side of the funnel plot (*Additional Figure 1*). The re-calculated adjusted pooled estimate was lower 43.6% (95% CI 37.5-50.0) with significant heterogeneity ($p < 0.0001$).

**Table 1 - Pooled estimates of vitamin D (25(OH)D) deficiency in acute and critically ill children and those with sepsis or respiratory tract infections**

| **Patient category** | **Number of studies (Total number of individuals; number of deficient individuals)** | **Pooled proportion (%, 95% CI)<br><br>Random effects** | **95% PI** | **Pooled proportion (%, 95% CI)<br><br>Fixed effects** | **Heterogeneity ($I^2$) % (95% CI)** | **Q value, d.f. p-value for heterogeneity** | **Eggers p-value** |
|---|---|---|---|---|---|---|---|
| All children (includes those with sepsis and respiratory tract infections) | 52 (7,434; 3,473) | 54.6 (48.5-60.6) | 17.5-87.2 | 45.7 (44.4-46.9) | 95.3 (94.5-96.0) | 1086.6,<br>51,<br>< 0.0001 | 0.01 |
| Critically ill children with sepsis only | 18 (889; 565) | 64.0 (52.0-74.4) | 17.1-93.9 | 63.0 (59.3-66.6) | 89.3 (84.6-92.5) | 158.52<br>17<br>< 0.0001 | 0.81 |
| Critically ill children with respiratory tract infections only | 25 (2,699; 1,076) | 48.7 (38.2-59.3) | 9.96-89.1 | 37.0 (35.0-39.1) | 94.3 (92.7-95.6) | 423.07<br>24<br>< 0.0001 | 0.05 |

CI = confidence intervals; $I^2$ = heterogeneity; df = degrees of freedom. Vitamin D deficiency defined in our study as 25(OH)D < 50 nmol/L (20 ng/mL). $I^2$ statistic used to estimate heterogeneity between pooled studies: $I^2 \geq 75\%$ was considered high heterogeneity; PI = Prediction Interval

*Figure 2 Pooled prevalence estimate for vitamin D deficiency in acute and critically ill children by study design.*

## Sensitivity analysis for prevalence



For peer review only

For peer review only - http://bmjopen.bmj.com/site/about/guidelines.xhtml

We did not detect material differences in prevalence after exclusion of the 12 studies which did not directly report prevalence < 50 nmol/L (53.0%, 95% CI 46.4-59.5; $I^2$ = 95.5%, 95% CI (94.5-96.2, p < 0.0001) (*Additional Table 14*).

When examining results by median sample size (defining "large" as ≥ 82 and "small" as < 82), we found that the 26[8, 53, 55-58, 66-68, 72-74, 77, 78, 80-91] studies with larger sample size included 6,094 total individuals and gave a prevalence estimate of 51.5% (95% CI 43.6-59.4; $I^2$ = 96.8%, 95% CI 96.0-97.4, p < 0.0001) . The remaining 26 studies with "smaller" sample sizes included 1,340 total children and estimated pooled prevalence as 58.2% (95% CI 47.5-68.2; $I^2$= 90.9%, 95% CI 87.9-93.2, p < 0.0001) (*Additional Table 14*).

We also conducted analysis by study design. Cohort studies (n = 22) yielded a prevalence estimate of 48.3% (95% CI 40.2-56.5; $I^2$ = 95.8%, 95% CI 94.6-96.7, p < 0.0001). In case-control studies (n = 26) the estimate was 63.4% (95% CI 54.9-71.2; $I^2$ = 92.2%, 95% CI 89.8-94.1, p < 0.0001) and in cross-sectional (n = 4) 34.8% (95% CI 12.8-66.0; $I^2$ = 96.7%, 95% CI 94.0-98.2, p <0.0001) (*Additional Table 14*, *Figure 2*).

We assessed whether studies' country of origin influenced results. Studies in India gave an estimate of 68.9% (95% CI 54.9-80.1; $I^2$ = 96.7% (95% CI 94.0-98.2, p < 0.0001). Similarly, we found higher pooled prevalence estimates for studies from Turkey (76.3%, 95% CI 60.9-87.0; $I^2$ = 91.1%, 95% CI 84.2-95.0, p < 0.0001). We also grouped studies by geography and economic development. Group 1: USA, Chile, Australia, Canada, Ireland, Japan, Spain; group 2: South Africa, China, Egypt, Iran, Turkey, Saudi Arabia; and group 3: Bangladesh, Thailand, and India. Prevalence was 37.2% (95% CI 29.7-45.5) for group 1 (n = 20), 61.8% (95% CI 53.2-69.7) for



For peer review only - http://bmjopen.bmj.com/site/about/guidelines.xhtml

group 2 (n = 19) and 70.8% (95% CI 58.3-80.7) for group 3 (n = 13) *(Additional Figure 2).* Variation attributable to heterogeneity was still high in the three subgroups ($I^2 > 90.0\%$).

Given the broad age range in included studies, we combined studies with only neonates[54, 65, 67, 69-72] and observed a prevalence estimate of 83.0% (95% CI 73.1-89.8) with less variation attributable to heterogeneity ($I^2$ = 76.6%, 95% CI 51.0-88.9, p = 0.0003). In all other studies (n = 45) that included children of other age ranges, estimated prevalence was lower at 49.7% (95% CI 43.5-55.8; $I^2$ = 95.2%, 95% CI 94.3-96.0, p < 0.0001) (*Additional Table 14, Additional Figure 3*).

**Post-hoc investigation to determine sources of heterogeneity**

To investigate the substantial heterogeneity observed in prevalence estimates, we incorporated study-specific characteristics (year of publication, total study sample size, quality score, study design, country group and clinical setting) as covariates in a random effects meta-regression model. We identified clinical setting and country groups as significant predictors, p < 0.01 *(Figure 3).* We found that the model fitted with all available covariates can explain 29.6% of $I^2$ with F = 4.14, p = 0.002 *(Additional Table 15).* We also conducted univariate meta-regressions for each of the six predictors *(Additional Figure 4).*

*Figure 3 Bubble plots of univariate meta-regressions.*

**Prevalence of vitamin D deficiency in children with sepsis and in those with respiratory tract infections**

A total of 889 (median 42, range 9 -160) patients had a diagnosis of sepsis, of which 565 (63.5%) were vitamin D deficient. Ten of the eighteen studies including septic patients were cohort (55.6%)



For peer review only - http://bmjopen.bmj.com/site/about/guidelines.xhtml

and eight (44.4%) case-control *(Additional Table 16)*. Most studies originated from India (n = 7) Turkey (n = 3) or Ireland (n = 2) and 16 were published between 2014 and 2017. Thirteen studies took place in a PICU and the remaining[65, 67, 70, 72] in NICUs. We found that all studies were of medium to high quality (median NOS score 6.5, range 5 – 8). Pooled prevalence of vitamin D deficiency was 64.0% (95% CI 52.0-74.4) *(Figure 4)* and median (IQR), 68.5% (50.4 to 71.6%). Variation attributable to heterogeneity was high ($I^2$ = 89.3%, 95% CI 84.6-92.5, $p < 0.0001$). Funnel plot was symmetric ($p > 0.05$) suggesting no small-study effects ($p = 0.81$, Egger's test) *(Additional Figure 5)*.

*Figure 4 Pooled prevalence estimate for vitamin D deficiency in children with sepsis.*

We also separately analysed studies of patients admitted for respiratory tract infections (n = 25) such as acute lower respiratory tract infection (ALRTI), pneumonia and bronchiolitis. Of these 2,699 total individuals (median 50), 1,076 (39.9%) were vitamin D deficient. These studies were of high to medium quality (median NOS score 7, range 6 - 8). Most originated from India (n = 6) and Spain (n = 4). We found a prevalence estimate of 48.7% (95% CI 38.2-59.3; $I^2$ = 94.3%, 95% CI 92.7-95.6, $p < 0.0001$) and median (IQR) at 36.7% (24.3 to 83.6%) with marginally non-significant evidence of bias ($p = 0.05$, Egger's test) (*Table 1)*. We therefore applied the trim and fill method and obtained an adjusted pooled estimate of 37.4% (95% CI 27.6-48.4) after four studies were added.

**Sensitivity analysis for prevalence in children with sepsis**

Exclusion of the studies[64, 67, 72, 92] utilising thresholds other than < 50 nmol/L for deficiency yielded a similar estimate of prevalence at 62.0% (95% CI 47.3-74.7; $I^2$ = 89.7%, 95% CI 84.5-93.2, $p < 0.0001$) *(Additional Table 17)*.



For peer review only - http://bmjopen.bmj.com/site/about/guidelines.xhtml

We examined pooled prevalence estimates according to median sample size (< 42 versus ≥ 42). Studies with a smaller sample size (n = 9; 204 total individuals) showed a pooled prevalence estimate of 64.7% (95% CI 52.5-75.3) with moderate variation attributable to heterogeneity ($I^2$ = 57.9%, 95% CI 11.8-79.9, p = 0.015). For the remaining nine studies (sample sizes ≥ 42, 685 total individuals) the estimate was 63.2% (95% CI 44.6-78.5) with high variation attributable to heterogeneity ($I^2$ = 94.3%, 95% CI 91.1-96.3, p < 0.0001).

There was no material change in prevalence estimates when analysed according to study design. The ten cohort studies (504 total individuals) gave an estimate of 63.2% (95% CI 43.7-79.1) with high variation attributable to heterogeneity ($I^2$ = 92.0, 95% CI 87.3-94.9 p < 0.0001). Case-control studies (n = 8; 385 total individuals) showed a pooled prevalence of 64.9% (95% CI 50.1-77.3; $I^2$ = 84.9%, 95% CI 72.0-91.8, p < 0.0001) *(Additional Table 17, Additional Figure 6).*

Studies from India (n = 7) gave a prevalence estimate of 66.0% (95% CI 51.4-78.1); $I^2$ = 81.1%, 95% CI 61.8-90.6, p < 0.0001). The three studies from Turkey assessing septic patients gave a pooled estimate of 59.2% (95% CI 13.6-93.1; $I^2$ = 97.8%, 95% CI 95.8-98.8, p < 0.0001) *(Additional Table 17).*

The pooled prevalence estimate in the four studies[65, 67, 70, 72] including neonates with sepsis was 73.7% (95% CI 60.3-83.8, $I^2$ = 76.0% 34.1-91.3, p = 0.006). The fourteen studies with children of different ages, excluding neonates, gave a pooled estimate of 60.7% (95% CI 45.5-74.0); $I^2$ = 90.1%, 95% CI 85.2-93.4, p < 0.0001) *(Additional Table 17)*. Four of the studies[56, 61, 87, 89] included children admitted with either sepsis or respiratory tract infections.

**Mortality in acute and critically ill children**



For peer review only - http://bmjopen.bmj.com/site/about/guidelines.xhtml

We identified 18 studies[8, 53, 56-59, 61, 64, 66, 72, 74-78, 82, 89, 92] assessing vitamin D status and mortality. These studies included a total of 2,463 individuals, from which 220 deaths (17.2%) were observed in 1,278 (51.9%) individuals with vitamin D deficiency and 99 deaths (8.4%) were observed in 1,185 individuals without deficiency (48.1%).

All 18 studies took place in a PICU apart from one[72], which considered only NICU patients. Sixteen of these studies (89.0%) were published between 2014 and 2017. Fourteen were cohort (77.8%) and four case-controls (22.2%). Almost half (n = 7) of the studies originated from India. Quality scores ranged from 5 to 8 with a median of 6.

Using a random effects model, we found that vitamin D deficiency in critically ill children significantly increased the risk of death (OR 1.81, 95% CI 1.24-2.64, p = 0.002) with low, non-significant heterogeneity ($I^2$ = 25.7%, 95% CI 0.0-58.0, p = 0.153) (*Figure 5*). However, small-study effects cannot be easily excluded (p = 0.084, Egger's test) (*Additional Figure 7*) and the 95% prediction interval (0.71-4.62) included the null value.

*Figure 5 Pooled odds ratio (OR) of risk of mortality in vitamin D deficient versus vitamin D non-deficient acute and critically ill children.*

**Sensitivity analysis for mortality in acute and critically ill children**

We obtained similar results through the fixed effects model (OR 1.72, 95% CI 1.27-2.33, p = 0.0005) (*Additional Figure 8*). When excluding studies with thresholds other than < 50 nmol/L indicating deficiency, we found the association between vitamin D deficiency and increased risk of mortality still significant but lower, both with the random (OR 1.59, 95% CI 1.05-2.41, p = 0.028; $I^2$ = 24.3%, 95% CI 0.00-59.9, p = 0.191) and fixed effect models (OR 1.52, 95% CI 1.08-



For peer review only - http://bmjopen.bmj.com/site/about/guidelines.xhtml

2.13, p = 0.016) without clear indication of small-study effects (p = 0.120, Egger's test) *(Additional Table 18).*

A significant association was also observed in analysis of the 14 cohort studies, both with the random (OR 1.80, 95% CI 1.15-2.81, p = 0.01) and fixed effects model (OR 1.65, 95% CI 1.17-2.34, p = 0.004) with low variation attributable to heterogeneity ($I^2$ = 31.3%, 95% CI 0.0-63.7) but significant small-study effects (p = 0.042). Trim and fill analysis estimated five unpublished studies and a non-significant association (OR 1.57, 95% CI 1.09-2.28, p = 0.131). Pooling the four case-control studies together, we obtained a significant positive association with the fixed (OR 1.97, 95% CI 1.02-3.82, p = 0.044) effects model but non-significant with the random effects model (OR 1.97, 95% CI 0.88-4.42, p = 0.098). The association was positive but not-significant when pooling the seven studies from India with the random effects model (OR 1.08, 95% CI 0.70-1.69, p = 0.710; $I^2$ = 0.0% 0.0-62.4, p = 0.589) and similar with fixed effects (OR 1.08, 95% CI 0.70-1.69, p = 0.710) *(Additional Table 18).*

**Mortality in patients with sepsis and respiratory tract infections**

We were unable to identify a sufficient number of studies assessing vitamin D and mortality for meta-analysis in individuals with sepsis. Three studies[8, 64, 67] measured vitamin D levels in paediatric patients with sepsis. One study[8] assessed mortality and did not find a significant association in children from 1 to 12 years with sepsis (n=124). None of the studies with children admitted for respiratory tract infections looked at the association between vitamin D deficiency and childhood mortality.

## DISCUSSION


For peer review only - http://bmjopen.bmj.com/site/about/guidelines.xhtml

Vitamin D deficiency is highly prevalent worldwide, even in countries with abundant sunshine. Studies have shown high prevalence of vitamin D deficiency in otherwise healthy children from high-income countries (9 to 24%) but also from middle and low-income countries in the Indian subcontinent (36 to 90%).[8]

We identified 52 studies representing a total of 7,434 children treated in ICU or emergency units for acute conditions who had blood 25(OH)D levels measured close to or upon admission. Our analysis shows that prevalence of vitamin D deficiency is generally high but very variable (range 5%[79] to 95%[60]) across ICU and emergency units in the paediatric population, particularly in individuals with sepsis. Importantly, our analysis showed a significantly increased risk of mortality in critically ill children with vitamin D deficiency. We carried out several analyses for sensitivity including fixed effects models, by study design, country group, age and sample size and found generally consistent results. A recently published meta-analysis[93] also investigated prevalence of vitamin D deficiency in critically ill children and its association with risk of mortality and showed similar results to ours. The study did not clearly report heterogeneity and small-study effects however, which we found to be critical limitations that must be addressed.

Subgroup analyses in patients with sepsis or respiratory tract infections demonstrated a high prevalence of vitamin D deficiency, consistent with the increased risk of bacterial or nosocomial infection in vitamin D deficient individuals identified elsewhere.[93]

Although sepsis is a leading cause of paediatric mortality and morbidity worldwide,[94] we found few studies assessing the relationship between vitamin D status and mortality in this population. We were unable to identify sufficient studies including patients with sepsis to perform a meta-analysis of vitamin D status and mortality. Sepsis remains an area of unmet need with high social



For peer review only - http://bmjopen.bmj.com/site/about/guidelines.xhtml

and financial costs.[24] Diagnostic criteria,[95] a lack of adequate biomarkers[96] and targeted treatment remain important challenges in research on sepsis. We did not find studies that assessed the risk of mortality in relation to vitamin D deficiency in children admitted for respiratory tract infections either.

Strengths of our review include the large number of studies and large total sample size, allowing a high-powered investigation to identify meaningful associations. For our systematic review and meta-analysis, we followed pre-specified eligibility criteria and used the PRISMA[27] and MOOSE guidelines[28] for reporting. We carried out multiple sensitivity analyses with few material differences in results. However, we note that the relationship between vitamin D deficiency and mortality was sensitive to study design and studies from India, probably due to the smaller number of individuals in those analyses. As expected for prevalence estimates, heterogeneity across studies was high overall. Only the prevalence analysis with neonates indicated somewhat lower variation attributable to heterogeneity ($I^2$ = 76.6%) along with a higher prevalence estimate (83.0%) compared to other analyses. We utilised meta-regression to investigate this substantial heterogeneity. From the six variables in our multi-variable model, only clinical setting and country groups were found to be significant predictors of pooled prevalence estimates of vitamin D deficiency and the full model could explain 29.6% of heterogeneity ($I^2$). Studies in NICU yielded higher prevalence estimates compared to studies in PICU. Studies from group 3 countries were also associated with higher prevalence estimates compared to studies from countries of group 1 and 2. Other variables, mainly individual patient characteristics such as age and ethnicity, were not directly available to us and may account for significant heterogeneity.

Our systematic review did not identify longitudinal studies with multiple time-point, pre-disease or pre-admission vitamin D measurements. The majority of studies were single centre with



For peer review only - http://bmjopen.bmj.com/site/about/guidelines.xhtml

heterogeneous patient groups and relatively small sample sizes. Few studies accounted for important confounders that influence vitamin D levels such as age, gender, BMI, season of measurements, vitamin D supplementation and comorbidities. The relationship observed between vitamin D deficiency and mortality could be due to reverse causation and future studies will need to control for covariates and other confounders. Low vitamin D levels could also represent a chronically deficient state due to reduced sunlight exposure, because of chronic illness, lifestyle factors or different country latitudes. In addition, we cannot rule out measurement bias such as dilution from intravenous fluids. Our results should be interpreted with caution since our review is based on evidence from observational studies. More research is warranted to strengthen the evidence and investigate whether vitamin D could be causally linked to acute or critical illness and what its contribution might be through various mechanisms such as anti-inflammatory or anti-microbial peptide responses.

Although included studies were generally of good quality, sample sizes varied considerably and were typically small. Half of the studies included less than 100 cases and only 10 (19.2 %) had a total sample size of more than 200 individuals. In addition, studies used a variety of definitions and age ranges to designate individuals as children. Our analysis only included mortality as a clinical outcome. A further general limitation is the difference in thresholds for vitamin D deficiency, particularly in the levels which are considered normal for infants and young children. Our assessment used the currently recommended threshold for deficiency (25(OH)D ≤ 50 nmol/L)[16] and a conservative estimate for studies which used different criteria. Although our review included a large number of studies and individuals, all studies were observational, and results could be subject to small-study effects.



For peer review only - http://bmjopen.bmj.com/site/about/guidelines.xhtml

Vitamin D remains an attractive biomarker and potential therapeutic agent in acute and critical care patients. Our review suggests that high quality focussed studies in each relevant paediatric population are needed first, which could then be followed by trials to establish safety and appropriate treatment regimens in children with acute or critical illness.

**Availability of data and materials**

Data and computational code used for processing and analysis are available at https://github.com/margarc/VitaminD_children

**Author contributions**

AJBT conceived the study. AJBT and IT designed the study. MC collected data and performed the analysis with input from MAC, IT, ABJT and EE. MC and AJBT wrote the manuscript with contributions from all authors.

**Declaration of interests**

The authors declare no conflicts of interest.

**Funding** Medical Research Council UK

**Acknowledgements**

AJBT was supported by the Medical Research Council (UK MED-BIO Programme Fellowship, MR/L01632X/1).

**Ethics committee approval:** Not applicable.

For peer review only



For peer review only - http://bmjopen.bmj.com/site/about/guidelines.xhtml

## Main Figures

**Figure 1 Flow chart of study selection process**

**Figure 2 Pooled prevalence estimate for vitamin D deficiency in acute and critically ill children by study design**. Forest plot shows results from the random effects model. Each diamond represents the pooled proportion of 25(OH)D deficiency for each of the subgroups (case-control, cohort, cross-sectional study designs). The diamond at the bottom represents the overall pooled proportion of all the 52 studies together. Each square shows the prevalence estimate of each study and the horizontal line across each square represents the 95% confidence interval (CI) of the prevalence estimate.

**Figure 3 Bubble plots of univariate meta-regressions. Each study is represented by a circle.** Predictor variables; A clinical setting and B country groups are shown on the x-axis and the effect measure logit transformed proportion shown on the vertical (y-axis). NICU = Neonatal Intensive Care Unit; PICU = Pediatric Intensive Care Unit; grp = country group; country group 1 = USA, Chile, Australia, Canada, Ireland, Japan, Spain; country group 2 = South Africa, China, Egypt, Iran, Turkey, Saudi Arabia; and country group 3 = Bangladesh, Thailand, and India

**Figure 4 Pooled prevalence estimate for vitamin D deficiency in children with sepsis.** Forest plot shows result from the random effects model. The diamond represents the overall pooled proportion of 25(OH)D deficiency from the meta-analysis of the 18 studies. Each square shows the prevalence estimate of each study and the horizontal line across each square represents the 95% confidence interval (CI) of the prevalence estimate.

**Figure 5 Pooled odds ratio (OR) of risk of mortality in vitamin D deficient versus vitamin D non-deficient acute and critically ill children.** Forest plot shows result from the random effects model. Diamond represents the overall OR (with corresponding 95% Confidence Interval). Each square shows the odds ratio of each study and the horizontal line across each square represents the 95% confidence interval (CI) of the estimate.

## Supplementary Material

**Additional Tables**

Additional Table 1 PRISMA Checklist 2009

Additional Table 2A Search terms used in OVID

Additional Table 2B Search terms used in PubMed

Additional Table 3A Newcastle Ottawa study quality scoring system (cohort studies)

Additional Table 3B Newcastle Ottawa study quality scoring system (case-control studies)

Additional Table 3C Newcastle Ottawa study quality scoring system (cross sectional studies)



For peer review only - http://bmjopen.bmj.com/site/about/guidelines.xhtml

Additional Table 4A Circulating 25(OH)D threshold levels used in the selected studies

Additional Table 4B Circulating 25(OH)D threshold levels used in the selected studies for prevalence in sepsis

Additional Table 5 Studies with thresholds other than <50 nmol/L

Additional Table 6 Excluded studies

Additional Table 7 Characteristics of the 52 included studies

Additional Table 8 Objectives and outcomes of included studies

Additional Table 9 Assay used in each study to measure Vitamin D levels

Additional Table 10 Funding and ethical approval of included studies

Additional Table 11 Age groups of children in each study

Additional Table 12 Prevalence of vitamin D deficiency in each study of acute and critically ill children (sorted from highest to lowest)

Additional Table 13 Characteristics of studies used in the meta-analysis for prevalence

Additional Table 14 Sensitivity analyses for prevalence of vitamin D deficiency in acute and critically ill children

Additional Table 15 Multivariate meta-regression model for prevalence

Additional Table 16 Characteristics of studies included in the meta-analysis for prevalence in individuals with sepsis

Additional Table 17 Sensitivity analyses for prevalence of vitamin D deficiency in acute and critically ill children with sepsis

Additional Table 18 Sensitivity analyses for mortality

## Additional Figures

Additional Figure 1 Funnel plot of studies of prevalence of vitamin D deficiency in acute and critically ill children

Additional Figure 2 Pooled prevalence estimate for vitamin D deficiency in acute and critically ill children (subgroup analysis by country group)

Additional Figure 3 Pooled prevalence estimate for vitamin D deficiency in acute and critically ill children (subgroup analysis of neonates versus all other age groups)

Additional Figure 4 Bubble plots of univariate meta-regressions.

Additional Figure 5 Funnel plot for prevalence of vitamin D deficiency in acute and critically ill children with sepsis

Additional Figure 6 Pooled prevalence estimate for vitamin D deficiency in acute and critically ill children with sepsis (subgroup analysis by study design)



For peer review only - http://bmjopen.bmj.com/site/about/guidelines.xhtml

Additional Figure 7 Funnel plot of risk of mortality in vitamin D deficient versus vitamin D non-deficient acute and critically ill children

Additional Figure 8 Pooled odds ratio and 95% CI for risk of mortality in vitamin D deficient versus vitamin D non-deficient acute and critically ill children (fixed effects model)

For peer review only



For peer review only - http://bmjopen.bmj.com/site/about/guidelines.xhtml

## References


1. Shuler FD, Wingate MK, Moore GH*, et al.* Sports health benefits of vitamin d. *Sports health* 2012;4(6):496-501.
2. Verceles AC, Weiler B, Koldobskiy D*, et al.* Association Between Vitamin D Status and Weaning From Prolonged Mechanical Ventilation in Survivors of Critical Illness. *Respir Care* 2015;60(7):1033-9.
3. Braegger C, Campoy C, Colomb V*, et al.* Vitamin D in the healthy European paediatric population. *J Pediatr Gastroenterol Nutr* 2013;56(6):692-701.
4. Holick MF, Binkley NC, Bischoff-Ferrari HA*, et al.* Evaluation, treatment, and prevention of vitamin D deficiency: an Endocrine Society clinical practice guideline. *J Clin Endocrinol Metab* 2011;96(7):1911-30.
5. Holick MF. Vitamin D deficiency. *N Engl J Med* 2007;357(3):266-81.
6. Aranow C. Vitamin D and the immune system. *J Investig Med* 2011;59(6):881-6.
7. Zhang YP, Wan YD, Sun TW*, et al.* Association between vitamin D deficiency and mortality in critically ill adult patients: a meta-analysis of cohort studies. *Crit Care* 2014;18(6):684.
8. Ponnarmeni S, Kumar Angurana S, Singhi S*, et al.* Vitamin D deficiency in critically ill children with sepsis. *Paediatr Int Child Health* 2016;36(1):15-21.
9. Kulie T, Groff A, Redmer J*, et al.* Vitamin D: an evidence-based review. *J Am Board Fam Med* 2009;22(6):698-706.
10. Hewison M. Antibacterial effects of vitamin D. *Nat Rev Endocrinol* 2011;7(6):337-45.
11. Kempker JA, West KG, Kempker RR*, et al.* Vitamin D status and the risk for hospital-acquired infections in critically ill adults: a prospective cohort study. *PLoS One* 2015;10(4):e0122136.
12. Martineau AR, Jolliffe DA, Hooper RL*, et al.* Vitamin D supplementation to prevent acute respiratory tract infections: systematic review and meta-analysis of individual participant data. *BMJ* 2017;356:i6583.
13. Absoud M, Cummins C, Lim MJ*, et al.* Prevalence and predictors of vitamin D insufficiency in children: a Great Britain population based study. *PLoS One* 2011;6(7):e22179.
14. Greer FR. Defining vitamin D deficiency in children: beyond 25-OH vitamin D serum concentrations. *Pediatrics* 2009;124(5):1471-3.
15. Abou-Zahr R, Kandil SB. A pediatric critical care perspective on vitamin D. *Pediatr Res* 2015;77(1-2):164-7.
16. Ross AC, Manson JE, Abrams SA*, et al.* The 2011 report on dietary reference intakes for calcium and vitamin D from the Institute of Medicine: what clinicians need to know. *J Clin Endocrinol Metab* 2011;96(1):53-8.
17. Ala-Kokko TI, Mutt SJ, Nisula S*, et al.* Vitamin D deficiency at admission is not associated with 90-day mortality in patients with severe sepsis or septic shock: Observational FINNAKI cohort study. *Ann Med* 2016;48(1-2):67-75.
18. Putzu A, Belletti A, Cassina T*, et al.* Vitamin D and outcomes in adult critically ill patients. A systematic review and meta-analysis of randomized trials. *J Crit Care* 2017;38:109-14.
19. Han JE, Jones JL, Tangpricha V*, et al.* High Dose Vitamin D Administration in Ventilated Intensive Care Unit Patients: A Pilot Double Blind Randomized Controlled Trial. *J Clin Transl Endocrinol* 2016;4:59-65.
20. Quraishi SA, De Pascale G, Needleman JS*, et al.* Effect of Cholecalciferol Supplementation on Vitamin D Status and Cathelicidin Levels in Sepsis: A Randomized, Placebo-Controlled Trial. *Crit Care Med* 2015;43(9):1928-37.
21. Singer M, Deutschman CS, Seymour CW*, et al.* The Third International Consensus Definitions for Sepsis and Septic Shock (Sepsis-3). *JAMA* 2016;315(8):801-10.




For peer review only - http://bmjopen.bmj.com/site/about/guidelines.xhtml

22. NHS-England. Improving outcomes for patients with sepsis: a cross-system action plan 2015 [Available from: https://www.england.nhs.uk/ourwork/part-rel/sepsis/.
23. Plunkett A, Tong J. Sepsis in children. *BMJ* 2015;350:h3017.
24. Weiss SL, Fitzgerald JC, Pappachan J*, et al.* Global epidemiology of pediatric severe sepsis: the sepsis prevalence, outcomes, and therapies study. *Am J Respir Crit Care Med* 2015;191(10):1147-57.
25. Mayr FB, Yende S, Angus DC. Epidemiology of severe sepsis. *Virulence* 2014;5(1):4-11.
26. De Pascale G, Ranzani OT, Nseir S*, et al.* Intensive care unit patients with lower respiratory tract nosocomial infections: the ENIRRIs project. *ERJ Open Res* 2017;3(4).
27. Moher D, Liberati A, Tetzlaff J*, et al.* Preferred reporting items for systematic reviews and meta-analyses: the PRISMA statement. *PloS Med* 2009;6.
28. Stroup DF, Berlin JA, Morton SC*, et al.* Meta-analysis of observational studies in epidemiology: a proposal for reporting. Meta-analysis Of Observational Studies in Epidemiology (MOOSE) group. *JAMA* 2000;283(15):2008-12.
29. Wells GA SB OCD, Peterson J, Welch V, Losos M,Tugwell P. The Newcastle-Ottawa Scale (NOS) for assessing the quality of nonrandomised studies in meta-analyses [Available from: http://www.ohri.ca/programs/clinical_epidemiology/oxford.asp.
30. Newcombe RG. Two-sided confidence intervals for the single proportion: comparison of seven methods. *Stat Med* 1998;17(8):857-72.
31. DerSimonian R, Laird N. Meta-analysis in clinical trials revisited. *Contemp Clin Trials* 2015;45(Pt A):139-45.
32. Higgins JP, Thompson SG, Spiegelhalter DJ. A re-evaluation of random-effects meta-analysis. *J R Stat Soc Ser A Stat Soc* 2009;172(1):137-59.
33. Borenstein M, Hedges LV, Higgins JP*, et al.* A basic introduction to fixed-effect and random-effects models for meta-analysis. *Res Synth Methods* 2010;1(2):97-111.
34. Haidich A-BJ. Meta-analysis in medical research. *Hippokratia* 2010;14(Suppl 1):29.
35. Lau J, Ioannidis JP, Schmid CH. Quantitative synthesis in systematic reviews. *Ann Intern Med* 1997;127(9):820-6.
36. Ioannidis JP, Patsopoulos NA, Evangelou E. Uncertainty in heterogeneity estimates in meta-analyses. *BMJ* 2007;335(7626):914-6.
37. Higgins JP, Thompson SG, Deeks JJ*, et al.* Measuring inconsistency in meta-analyses. *BMJ* 2003;327(7414):557-60.
38. Higgins JP, Thompson SG. Quantifying heterogeneity in a meta-analysis. *Stat Med* 2002;21(11):1539-58.
39. Egger M, Davey Smith G, Schneider M*, et al.* Bias in meta-analysis detected by a simple, graphical test. *BMJ* 1997;315(7109):629-34.
40. Sterne JA, Sutton AJ, Ioannidis JP*, et al.* Recommendations for examining and interpreting funnel plot asymmetry in meta-analyses of randomised controlled trials. *BMJ* 2011;343:d4002.
41. Duval S, Tweedie R. Trim and fill: A simple funnel-plot-based method of testing and adjusting for publication bias in meta-analysis. *Biometrics* 2000;56(2):455-63.
42. Panityakul T, Bumrungsup C, Knapp G. On estimating residual heterogeneity in random-effects meta-regression: a comparative study. *J Stat Theory Appl* 2013;12(3):253.
43. Knapp G, Hartung J. Improved tests for a random effects meta-regression with a single covariate. *Stat Med* 2003;22(17):2693-710.
44. Schwarzer G. meta: An R package for meta-analysis. *R news* 2007;7(3):40-5.
45. Viechtbauer W. Conducting meta-analyses in R with the metafor package. *J Stat Softw* 2010;36(3).



For peer review only - http://bmjopen.bmj.com/site/about/guidelines.xhtml

46. Garg D, Sharma VK, Karnawat B. Association of serum vitamin D with acute lower respiratory infection in Indian children under 5 years: a case control study. *Int J Contemp Pediatrics* 2016;3(4):1164-9.
47. Aydemir G, Cekmez F, Kalkan G*, et al.* High serum 25-hydroxyvitamin D levels are associated with pediatric sepsis. *Tohoku J Exp Med* 2014;234(4):295-8.
48. Gamal TS, Madiha AS, Hanan MK*, et al.* Neonatal and Maternal 25-OH Vitamin D Serum Levels in Neonates with Early-Onset Sepsis. *Children (Basel)* 2017;4(5).
49. Seliem MS, Abdel Haie OM, Mansour AI*, et al.* The relation between vitamin D level and increased risk for early-onset neonatal sepsis in full-term infants. *Medical Research Journal* 2016;15(1):16-21.
50. McNally JD, Leis K, Matheson LA*, et al.* Vitamin D deficiency in young children with severe acute lower respiratory infection. *Pediatr Pulmonol* 2009;44(10):981-8.
51. McNally JD, Doherty DR, Lawson ML*, et al.* The relationship between vitamin D status and adrenal insufficiency in critically ill children. *J Clin Endocrinol Metab* 2013;98(5):E877-81.
52. McNally JD, Iliriani K, Pojsupap S*, et al.* Rapid normalization of vitamin D levels: a meta-analysis. *Pediatrics* 2015;135(1):e152-66.
53. McNally JD, Menon K, Chakraborty P*, et al.* The association of vitamin D status with pediatric critical illness. *Pediatrics* 2012;130(3):429-36.
54. Karatekin G, Kaya A, Salihoglu O*, et al.* Association of subclinical vitamin D deficiency in newborns with acute lower respiratory infection and their mothers. *Eur J Clin Nutr* 2009;63(4):473-7.
55. Vo P, Koppel C, Espinola JA*, et al.* Vitamin D Status at the Time of Hospitalization for Bronchiolitis and Its Association with Disease Severity. *J Pediatr* 2018;203:416-22.e1.
56. Shah SK, Kabra SK, Gupta N*, et al.* Vitamin D Deficiency and Parathyroid Response in Critically-ill Children: Association with Illness Severity and Clinical Outcomes. *Indian Pediatr* 2016;53(6):479-84.
57. Sankar J, Lotha W, Ismail J*, et al.* Vitamin D deficiency and length of pediatric intensive care unit stay: a prospective observational study. *Ann Intensive Care* 2016;6(1):3.
58. Dayal D, Kumar S, Sachdeva N*, et al.* Fall in Vitamin D Levels during Hospitalization in Children. *Int J Pediatr* 2014;2014:291856.
59. Prasad S, Raj D, Warsi S*, et al.* Vitamin D Deficiency and Critical Illness. *Indian J Pediatr* 2015;82(11):991-5.
60. Wayse V, Yousafzai A, Mogale K*, et al.* Association of subclinical vitamin D deficiency with severe acute lower respiratory infection in Indian children under 5 y. *Eur J Clin Nutr* 2004;58(4):563-7.
61. Ebenezer K, Job V, Antonisamy B*, et al.* Serum Vitamin D Status and Outcome among Critically Ill Children Admitted to the Pediatric Intensive Care Unit in South India. *Indian J Pediatr* 2016;83(2):120-5.
62. Narang GS, Arora S, Kukreja S. Association of Vitamin D Deficiency with Acute Lower Respiratory Infection in Toddlers. *Journal of Nepal Paediatric Society* 2016;36(1):14-6.
63. Jat KR, Kaur J, Guglani V. Vitamin D and Pneumonia in Children: A Case Control Study. *J Pulm Med Respir Res* 2016;2(004).
64. Sankar J, Ismail J, Das R*, et al.* Effect of Severe Vitamin D Deficiency at Admission on Shock Reversal in Children With Septic Shock. *J Intensive Care Med* 2017.
65. Dhandai R, Jajoo M, Singh A*, et al.* Association of vitamin D deficiency with an increased risk of late-onset neonatal sepsis. *Paediatr Int Child Health* 2018;38(3):193-7.
66. Asilioglu N, Cigdem H, Paksu MS. Serum Vitamin D Status and Outcome in Critically Ill Children. *Indian J Crit Care Med* 2017;21(10):660-4.
67. Say B, Uras N, Sahin S*, et al.* Effects of cord blood vitamin D levels on the risk of neonatal sepsis in premature infants. *Korean J Pediatr* 2017;60(8):248-53.
68. Cayir A, Turan MI, Ozkan O*, et al.* Vitamin D levels in children diagnosed with acute otitis media. *J Pak Med Assoc* 2014;64(11):1274-7.



For peer review only - http://bmjopen.bmj.com/site/about/guidelines.xhtml

69. Cetinkaya M, Cekmez F, Buyukkale G, *et al.* Lower vitamin D levels are associated with increased risk of early-onset neonatal sepsis in term infants. *J Perinatol* 2015;35(1):39.
70. Cizmeci MN, Kanburoglu MK, Akelma AZ, *et al.* Cord-blood 25-hydroxyvitamin D levels and risk of early-onset neonatal sepsis: a case-control study from a tertiary care center in Turkey. *Eur J Pediatr* 2015;174(6):809-15.
71. Dinlen N, Zenciroglu A, Beken S, *et al.* Association of vitamin D deficiency with acute lower respiratory tract infections in newborns. *J Matern Fetal Neonatal Med* 2016;29(6):928-32.
72. Onwuneme C, Martin F, McCarthy R, *et al.* The Association of Vitamin D Status with Acute Respiratory Morbidity in Preterm Infants. *J Pediatr* 2015;166(5):1175-80.e1.
73. Madden K, Feldman HA, Smith EM, *et al.* Vitamin D deficiency in critically ill children. *Pediatrics* 2012;130(3):421-8.
74. Rippel C, South M, Butt WW, *et al.* Vitamin D status in critically ill children. *Intensive Care Med* 2012;38(12):2055-62.
75. Korwutthikulrangsri M, Mahachoklertwattana P, Lertbunrian R, *et al.* Vitamin D deficiency and adrenal function in critically ill children. *J Med Assoc Thai* 2015;98(4):365-72.
76. Elmoneim A, Rhill R, Rahalli M, *et al.* Vitamin D level in pediatric intensive care unit (PICU) patients: its relation to severity of illness. *Pediat Therapeut* 2016;6(293):2161-0665.
77. García-Soler P, Morales-Martínez A, Rosa-Camacho V, *et al.* Vitamin D deficiency and morbimortality in critically ill paediatric patients. *Anales de Pediatría (English Edition)* 2017;87(2):95-103.
78. Badawi NES, Algebaly HAF, El Sayed R, *et al.* Vitamin D deficiency in critically ill children. *Kasr Al Ainy Medical Journal* 2017;23(1):6.
79. Roth DE, Jones AB, Prosser C, *et al.* Vitamin D status is not associated with the risk of hospitalization for acute bronchiolitis in early childhood. *Eur J Clin Nutr* 2009;63(2):297-9.
80. Cebey-Lopez M, Pardo-Seco J, Gomez-Carballa A, *et al.* Role of Vitamin D in Hospitalized Children With Lower Tract Acute Respiratory Infections. *J Pediatr Gastroenterol Nutr* 2016;62(3):479-85.
81. Alonso MA, Pallavicini ZF, Rodriguez J, *et al.* Can vitamin D status be assessed by serum 25OHD in children? *Pediatr Nephrol* 2015;30(2):327-32.
82. Ayulo M, Jr., Katyal C, Agarwal C, *et al.* The prevalence of vitamin D deficiency and its relationship with disease severity in an urban pediatric critical care unit. *Endocr Regul* 2014;48(2):69-76.
83. Rey C, Sanchez-Arango D, Lopez-Herce J, *et al.* Vitamin D deficiency at pediatric intensive care admission. *J Pediatr (Rio J)* 2014;90(2):135-42.
84. Onwuneme C, Carroll A, Doherty D, *et al.* Inadequate vitamin D levels are associated with culture positive sepsis and poor outcomes in paediatric intensive care. *Acta Paediatr* 2015;104(10):e433-8.
85. Jia K-P, Zhao L-F, Feng N, *et al.* Lower level of vitamin D3 is associated with susceptibility to acute lower respiratory tract infection (ALRTI) and severity: a hospital based study in Chinese infants. *Int J Clin Exp Med* 2017;10(5):7997-8003.
86. Halwany AS, Deghady AAE, Moustafa AAA, *et al.* Study of Serum Vitamin D Level among Critically Ill Patients Admitted to Alexandria University Pediatric Intensive Care Unit. *JMSCR* 2017;5(4):20776-81.
87. Li W, Cheng X, Guo L, *et al.* Association between serum 25-hydroxyvitamin D concentration and pulmonary infection in children. *Medicine* 2018;97(1):e9060.
88. El Sakka AS, Imam SS, Amer HA, *et al.* Vitamin D deficiency and low hemoglobin level as risk factors for severity of acute lower respiratory tract infections in Egyptian children: A case-control study. *Egyptian Pediatric Association Gazette* 2014;62(1):1-7.
89. Bustos R, Rodriguez-Nunez I, Peña RZ, *et al.* Vitamin D deficiency in children admitted to the paediatric intensive care unit. *Revista chilena de pediatria* 2016;87(6):480-6.



For peer review only - http://bmjopen.bmj.com/site/about/guidelines.xhtml

90. Hurwitz JL, Jones BG, Penkert RR, *et al.* Low Retinol-Binding Protein and Vitamin D Levels Are Associated with Severe Outcomes in Children Hospitalized with Lower Respiratory Tract Infection and Respiratory Syncytial Virus or Human Metapneumovirus Detection. *J Pediatr* 2017;187:323-7.

91. Yaghmaie B, Sayarifard F, Modarresi M, *et al.* Investigation of serum 25 hydroxy vitamin D level in critically ill children and its relation with demographic and clinical risk factors. *Biomed Res* 2017;28(5).

92. El-Gamasy M, Eldeeb M, Abdelmageed M. Prognostic Value of Vitamin D Status in Pediatric Patients with Acute Kidney Injury in Tanta University Emergency Hospital, Egypt. *J Emerg Intern Med* 2017;1(1):5.

93. McNally JD, Nama N, O'Hearn K, *et al.* Vitamin D deficiency in critically ill children: a systematic review and meta-analysis. *Crit Care* 2017;21(1):287.

94. Randolph AG, McCulloh RJ. Pediatric sepsis: important considerations for diagnosing and managing severe infections in infants, children, and adolescents. *Virulence* 2014;5(1):179-89.

95. Vincent JL. The Clinical Challenge of Sepsis Identification and Monitoring. *PLoS Med* 2016;13(5):e1002022.

96. Mathias B, Mira JC, Larson SD. Pediatric sepsis. *Curr Opin Pediatr* 2016;28(3):380-7.

For peer review only



For peer review only - http://bmjopen.bmj.com/site/about/guidelines.xhtml

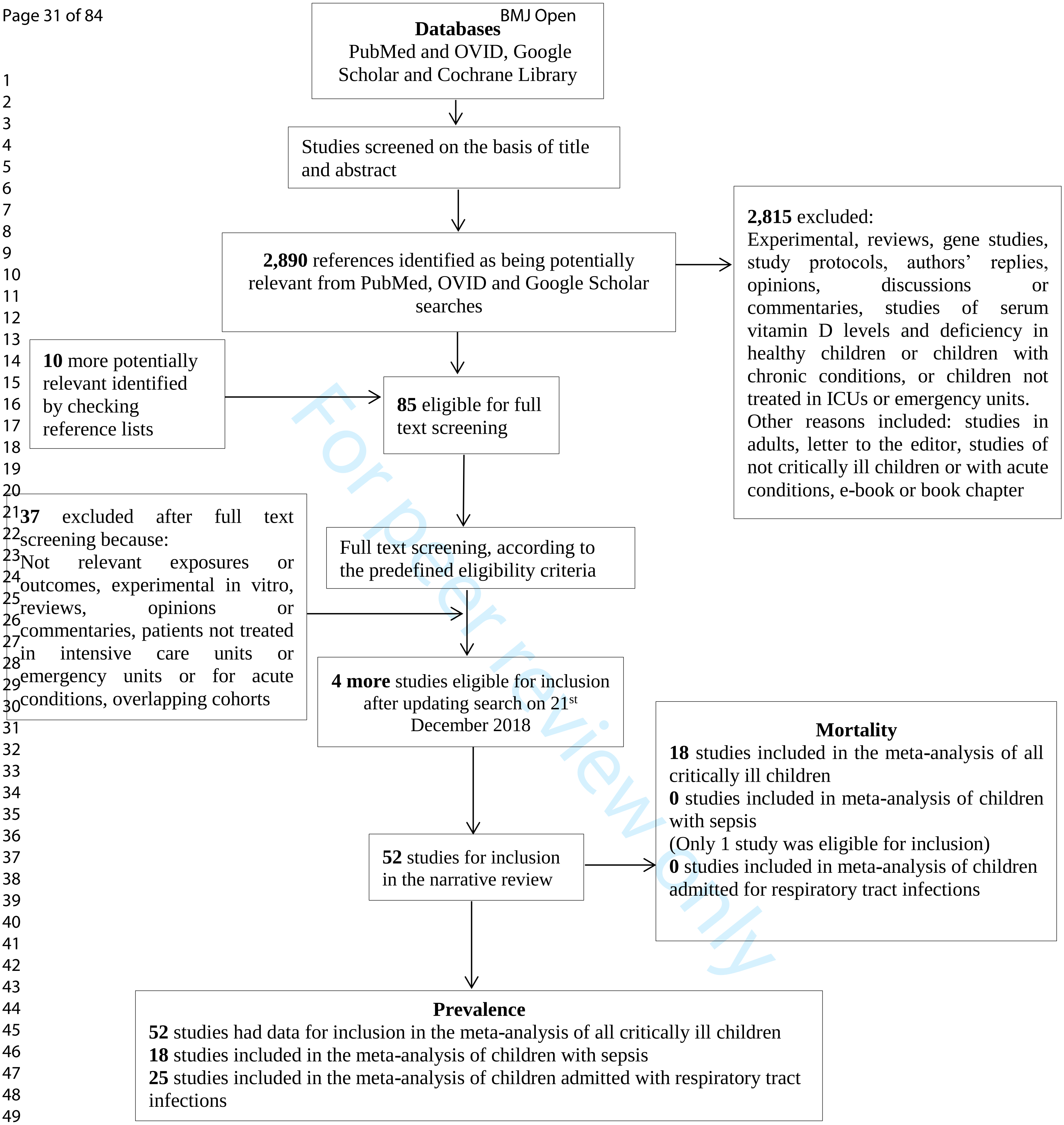


**Figure 1** Flow chart of study selection process

For peer review only

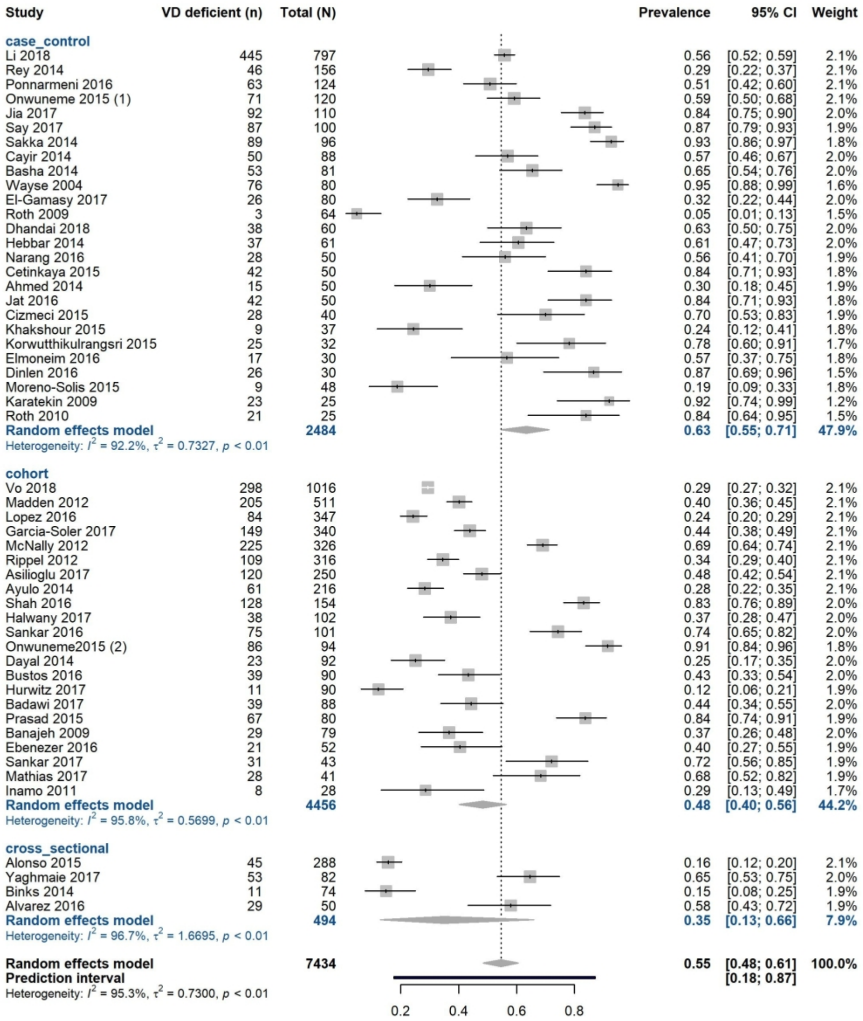


Figure 2 Pooled prevalence estimate for vitamin D deficiency in acute and critically ill children by study design. Forest plot shows results from the random effects model. Each diamond represents the pooled proportion of 25(OH)D deficiency for each of the subgroups (case-control, cohort, cross-sectional study designs). The diamond at the bottom represents the overall pooled proportion of all the 52 studies together. Each square shows the prevalence estimate of each study and the horizontal line across each square represents the 95% confidence interval (CI) of the prevalence estimate.

203x254mm (300 x 300 DPI)

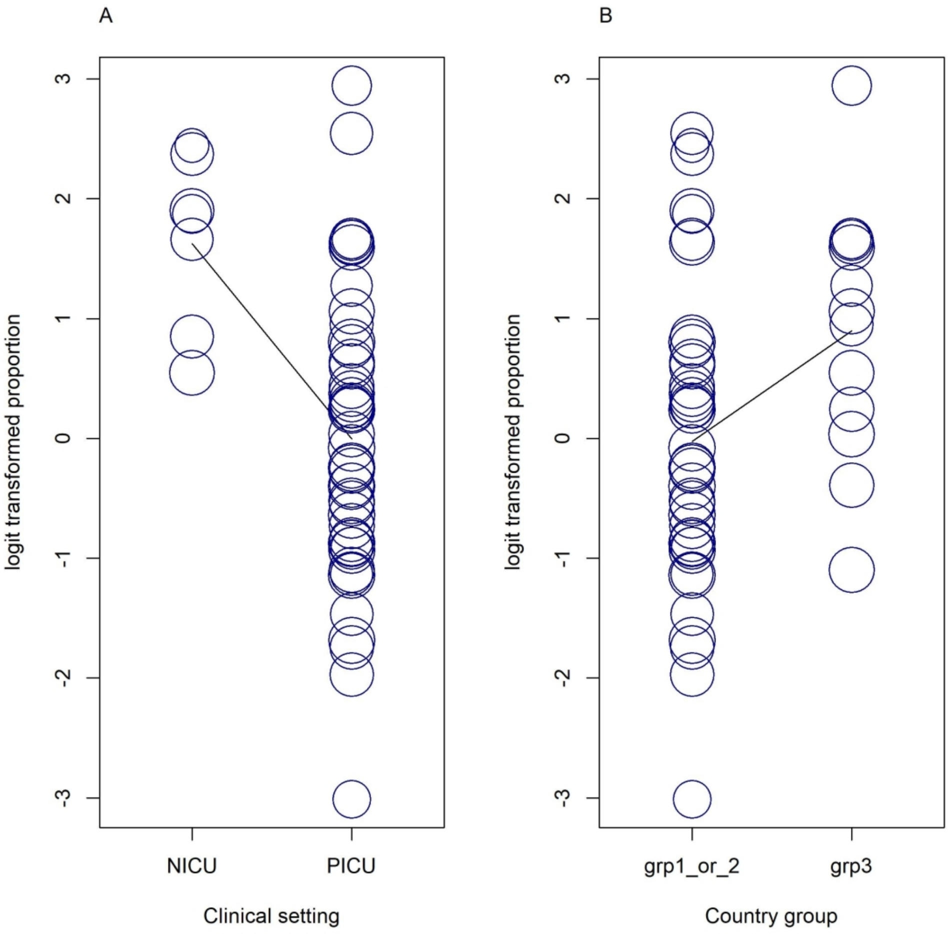


Figure 3 Bubble plots of univariate meta-regressions. Each study is represented by a circle. Predictor variables; A clinical setting and B country groups are shown on the x-axis and the effect measure logit transformed proportion shown on the vertical (y-axis). NICU = Neonatal Intensive Care Unit; PICU = Pediatric Intensive Care Unit; grp = country group; country group 1 = USA, Chile, Australia, Canada, Ireland, Japan, Spain; country group 2 = South Africa, China, Egypt, Iran, Turkey, Saudi Arabia; and country group 3 = Bangladesh, Thailand, and India

203x203mm (300 x 300 DPI)

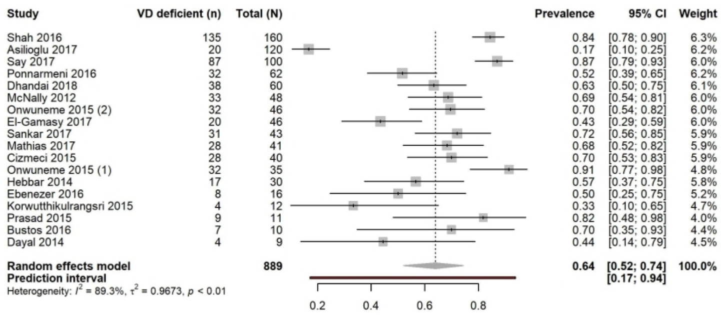


Figure 4 Pooled prevalence estimate for vitamin D deficiency in children with sepsis. Forest plot shows result from the random effects model. The diamond represents the overall pooled proportion of 25(OH)D deficiency from the meta-analysis of the 18 studies. Each square shows the prevalence estimate of each study and the horizontal line across each square represents the 95% confidence interval (CI) of the prevalence estimate.

254x152mm (300 x 300 DPI)

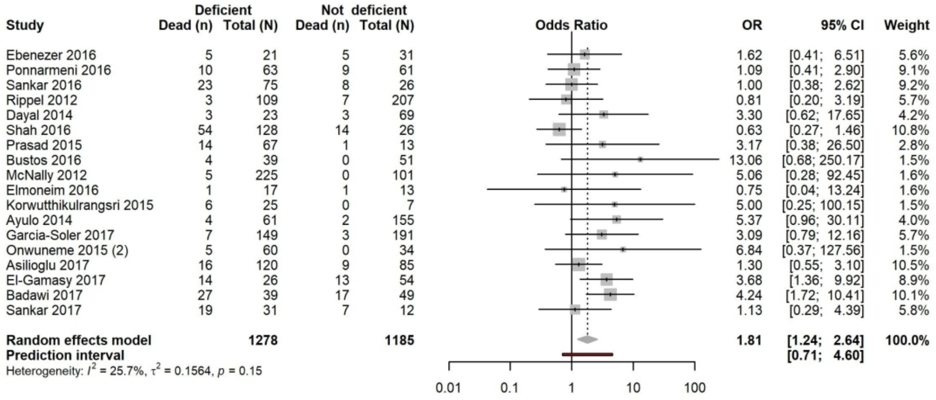


Figure 5 Pooled odds ratio (OR) of risk of mortality in vitamin D deficient versus vitamin D non-deficient acute and critically ill children. Forest plot shows result from the random effects model. Diamond represents the overall OR (with corresponding 95% Confidence Interval). Each square shows the odds ratio of each study and the horizontal line across each square represents the 95% confidence interval (CI) of the estimate.

203x127mm (300 x 300 DPI)

# Supplementary Material

## Table of Contents

Additional Table 1 PRISMA Checklist 2009 ........ 3
Additional Table 2A Search terms used in OVID ........ 5
Additional Table 2B Search terms used in PubMed ........ 6
Additional Table 3A Newcastle Ottawa study quality scoring system (cohort studies) ........ 7
Additional Table 3B Newcastle Ottawa study quality scoring system (case-control studies) ........ 8
Additional Table 3C Newcastle Ottawa study quality scoring system (cross-sectional studies) ........ 9
Additional Table 4A Circulating 25(OH)D threshold levels used in the selected studies ........ 10
Additional Table 4B Circulating 25(OH)D threshold levels used in the selected studies for prevalence in sepsis ........ 13
Additional Table 5 Studies with thresholds other than < 50 nmol/L ........ 14
Additional Table 6 Excluded studies ........ 15
Additional Table 7 Characteristics of the 52 included studies ........ 16
Additional Table 8 Objectives and outcomes of included studies ........ 22
Additional Table 9 Assay used in each study to measure Vitamin D levels ........ 26
Additional Table 10 Funding and ethical approval of included studies ........ 27
Additional Table 11 Age groups of children in each study ........ 30
Additional Table 12 Prevalence of vitamin D deficiency in each study of acute and critically ill children (sorted from highest to lowest) ........ 31
Additional Table 13 Characteristics of studies used in the meta-analysis of prevalence ........ 33
Additional Table 14 Sensitivity analyses for prevalence of vitamin D deficiency in acute and critically ill children ........ 35
Additional Table 15 Multivariate meta-regression model for prevalence ........ 36
Additional Table 16 Characteristics of studies included in the meta-analysis for prevalence in individuals with sepsis ........ 37
Additional Table 17 Sensitivity analyses for prevalence of vitamin D deficiency in acute and critically ill children with sepsis ........ 38
Additional Table 18 Sensitivity analyses for mortality. Pooled odds ratios for risk of mortality in deficient versus not deficient children ........ 39
Additional Figures ........ 40
Additional Figure 1 Funnel plot of studies of prevalence of vitamin D deficiency in acute and critically ill children. ........ 40



For peer review only - http://bmjopen.bmj.com/site/about/guidelines.xhtml

Additional Figure 2. Pooled prevalence estimate for vitamin D deficiency in acute and critically ill children (by country group). .......... 41

Additional Figure 3. Pooled prevalence estimates for vitamin D deficiency in acute and critically ill children (neonates versus all other age groups). .......... 42

Additional Figure 4 Bubble plots of univariate meta-regressions. .......... 43

Additional Figure 5 Funnel plot for prevalence of vitamin D deficiency in acute and critically ill children with sepsis. .......... 44

Additional Figure 6 Pooled prevalence estimate for vitamin D deficiency in acute and critically ill children with sepsis (subgroup analysis by study design). .......... 45

Additional Figure 7 Funnel plot of risk of mortality in vitamin D deficient versus vitamin D non-deficient acute and critically ill children. .......... 46

Additional Figure 8 Pooled odds ratio (OR) and 95% CI for risk of mortality in vitamin D deficient versus vitamin D non-deficient acute and critically ill children (fixed effects model). .......... 47

For peer review only



For peer review only - http://bmjopen.bmj.com/site/about/guidelines.xhtml

| Additional Table 1 PRISMA Checklist 2009 | | | |
|---|---|---|---|
| **Section/topic** | **#** | **Checklist item** | **Reported on page #** |
| **TITLE** | | | |
| Title | 1 | Identify the report as a systematic review, meta-analysis, or both. | 1 |
| **ABSTRACT** | | | |
| Structured summary | 2 | Provide a structured summary including, as applicable: background; objectives; data sources; study eligibility criteria, participants, and interventions; study appraisal and synthesis methods; results; limitations; conclusions and implications of key findings; systematic review registration number. | 2-3 |
| **INTRODUCTION** | | | |
| Rationale | 3 | Describe the rationale for the review in the context of what is already known. | 4-5 |
| Objectives | 4 | Provide an explicit statement of questions being addressed with reference to participants, interventions, comparisons, outcomes, and study design (PICOS). | 6-8 |
| **METHODS** | | | |
| Protocol and registration | 5 | Indicate if a review protocol exists, if and where it can be accessed (e.g., Web address), and, if available, provide registration information including registration number. | 9 |
| Eligibility criteria | 6 | Specify study characteristics (e.g., PICOS, length of follow-up) and report characteristics (e.g., years considered, language, publication status) used as criteria for eligibility, giving rationale. | 5-7 |
| Information sources | 7 | Describe all information sources (e.g., databases with dates of coverage, contact with study authors to identify additional studies) in the search and date last searched. | 6-7 |
| Search | 8 | Present full electronic search strategy for at least one database, including any limits used, such that it could be repeated. | 6 and supplementary material |
| Study selection | 9 | State the process for selecting studies (i.e., screening, eligibility, included in systematic review, and, if applicable, included in the meta-analysis). | 6-7 |
| Data collection process | 10 | Describe method of data extraction from reports (e.g., piloted forms, independently, in duplicate) and any processes for obtaining and confirming data from investigators. | 7 |
| Data items | 11 | List and define all variables for which data were sought (e.g., PICOS, funding sources) and any assumptions and simplifications made. | 7-8 |
| Risk of bias in individual studies | 12 | Describe methods used for assessing risk of bias of individual studies (including specification of whether this was done at the study or outcome level), and how this information is to be used in any data synthesis. | 8 |
| Summary measures | 13 | State the principal summary measures (e.g., risk ratio, difference in means). | 8 |
| Synthesis of results | 14 | Describe the methods of handling data and combining results of studies, if done, including measures of consistency (e.g., $I^2$) for each meta-analysis. | 8-10 |

**Additional Table 1 PRISMA Checklist 2009**



For peer review only - http://bmjopen.bmj.com/site/about/guidelines.xhtml

| Section/topic | # | Checklist item | Reported on page # |
|---|---|---|---|
| Risk of bias across studies | 15 | Specify any assessment of risk of bias that may affect the cumulative evidence (e.g., publication bias, selective reporting within studies). | 8 |
| Additional analyses | 16 | Describe methods of additional analyses (e.g., sensitivity or subgroup analyses, meta-regression), if done, indicating which were pre-specified. | 9-10 and 15 |
| **RESULTS** | | | |
| Study selection | 17 | Give numbers of studies screened, assessed for eligibility, and included in the review, with reasons for exclusions at each stage, ideally with a flow diagram. | 10 |
| Study characteristics | 18 | For each study, present characteristics for which data were extracted (e.g., study size, PICOS, follow-up period) and provide the citations. | 14-19 (supplementary material) |
| Risk of bias within studies | 19 | Present data on risk of bias of each study and, if available, any outcome level assessment (see item 12). | 5-7 (supplementary material) |
| Results of individual studies | 20 | For all outcomes considered (benefits or harms), present, for each study: (a) simple summary data for each intervention group (b) effect estimates and confidence intervals, ideally with a forest plot. | 13,15,18 and supplementary material |
| Synthesis of results | 21 | Present results of each meta-analysis done, including confidence intervals and measures of consistency. | 13 and supplementary material pages 32-36 |
| Risk of bias across studies | 22 | Present results of any assessment of risk of bias across studies (see Item 15). | 5-7 (supplementary material) |
| Additional analysis | 23 | Give results of additional analyses, if done (e.g., sensitivity or subgroup analyses, meta-regression [see Item 16]). | supplementary material pages 32-36 |
| **DISCUSSION** | | | |
| Summary of evidence | 24 | Summarize the main findings including the strength of evidence for each main outcome; consider their relevance to key groups (e.g., healthcare providers, users, and policy makers). | 19-21 |
| Limitations | 25 | Discuss limitations at study and outcome level (e.g., risk of bias), and at review-level (e.g., incomplete retrieval of identified research, reporting bias). | 20-22 |
| Conclusions | 26 | Provide a general interpretation of the results in the context of other evidence, and implications for future research. | 22 |
| **FUNDING** | | | |
| Funding | 27 | Describe sources of funding for the systematic review and other support (e.g., supply of data); role of funders for the systematic review. | 22 |

From: Moher D, Liberati A, Tetzlaff J, Altman DG, The PRISMA Group (2009). Preferred Reporting Items for Systematic Reviews and Meta-Analyses: The PRISMA Statement. PLoS Med 6(6): e1000097. doi:10.1371/journal.pmed1000097 For more information, visit: www.prisma-statement.org.



For peer review only - http://bmjopen.bmj.com/site/about/guidelines.xhtml

| Additional Table 2A Search terms used in OVID | |
|---|---|
| 1. | Vitamin D |
| 2. | Sepsis or septic shock |
| 3. | Vitamin D or cholecalciferol* or ergocalciferol or 25-hydroxyvitamin D or calcidiol or calcitriol |
| 4. | Intensive care unit* or critical care |
| 5. | |
| 6. | Multiple organ dysfunction syndrome or multiple organ failure |
| 7. | (1 and 2) or 5 |
| 8. | ((1 or 3) and 2 or 4 |
| 9. | (1 or 3) and 2 |
| 10. | (1 or 3) and 4 |
| 11. | (1 or 3) and 5 |
| 12. | Sepsis |
| 13. | multi* organ dysfunction syndrome or multiple organ failure |
| 14. | multi* organ dysfunction syndrome or multi* organ failure |
| 15. | (2 or 8) and 3 |
| 16. | critical* ill or acute condition* or intensive care unit |
| 17. | child* or pediatric* |
| 18. | vitamin D or cholecalciferol or ergocalciferol or 25-hydroxyvitamin D or calcidiol or calcitriol |
| 19. | 15 and 16 and 17 |
| 20. | Vitamin D blood levels or 25-hydroxyvitamin |
| 21. | 5 and 3 and 15 and 16 |
| 22. | 2 and 15 and 16 and 17 |
| 23. | 16 and 2 |
| 24. | 16 and 2 and 3 |
| 25. | Pediatric* |
| 26. | Pediatric* and 5 and 2 |
| 27. | 24 and 3 and 15 |

For peer review only

| Additional Table 2B Search terms used in PubMed | |
|---|---|
| **Term searched** | |
| 1. | Vitamin D.mp. |
| 2. | (sepsis or septic shock).mp. |
| 3. | (vitamin D or cholecalciferol* or ergocalciferol or 25-hydroxyvitamin D or calcidiol or calcitriol).mp. |
| 4. | (intensive care unit* or critical care).mp. |
| 5. | (multi* organ dysfunction syndrome or multiple organ failure).mp. |
| 6. | (critical* ill or acute condition* or intensive care unit).mp. |
| 7. | (toddler or infant or child* or neonate* or baby or teenager or pediatric* or paediatric*).mp. |
| 8. | 3 and 6 and 2 and 7 |

For peer review only

**Additional Table 3A Newcastle Ottawa study quality scoring system (cohort studies)**

| Study | Selection | | | | Comparability | Outcome | | | Number of stars (out of 9 total) |
|---|---|---|---|---|---|---|---|---|---|
| | Representativeness of the exposed cohort | Selection of the non-exposed cohort | Ascertainment of exposure | Demonstration that outcome of interest was not present at the start of the study | Comparability of cohorts on the basis of the design or analysis (maximum 2 stars) | Assessment of outcome | Was follow-up long enough for outcome to occur (≥28 days after admission to the ICU) | Adequacy of follow up of cohorts | |
| Ebenezer 2016 | 1 | 1 | 1 | 1 | 0 | 1 | 0 | 1 | 6 |
| Sankar 2016 | 1 | 1 | 1 | 1 | 2 | 1 | 0 | 1 | 8 |
| Rippel 2012 | 1 | 1 | 1 | 1 | 1 | 1 | 1 | 1 | 8 |
| Madden 2012 | 1 | 1 | 1 | 1 | 2 | 1 | 0 | 1 | 8 |
| McNally 2012 | 1 | 1 | 1 | 0 | 1 | 1 | 1 | 1 | 7 |
| Dayal 2014 | 1 | 1 | 1 | 1 | 0 | 1 | 0 | 1 | 6 |
| Ayulo 2014 | 1 | 1 | 1 | 1 | 2 | 1 | 0 | 0 | 7 |
| Bustos 2016 | 1 | 1 | 1 | 0 | 2 | 1 | 0 | 1 | 7 |
| Prasad 2015 | 1 | 1 | 1 | 0 | 2 | 1 | 0 | 1 | 7 |
| Onwuneme 2015 (2) | 1 | 1 | 1 | 1 | 2 | 1 | 0 | 1 | 8 |
| Inamo 2011 | 1 | 1 | 1 | 0 | 1 | 1 | 0 | 1 | 6 |
| Shah 2016 | 1 | 1 | 1 | 1 | 0 | 1 | 0 | 1 | 6 |
| Lopez 2016 | 1 | 1 | 1 | 1 | 2 | 1 | 0 | 1 | 8 |
| Garcia-Soler 2017 | 1 | 1 | 1 | 1 | 2 | 1 | 0 | 0 | 7 |
| Sankar 2017 | 1 | 1 | 1 | 1 | 2 | 1 | 0 | 1 | 8 |
| Asilioglu 2017 | 1 | 1 | 1 | 1 | 1 | 1 | 0 | 1 | 7 |
| Halwany 2017 | 1 | 1 | 1 | 1 | 1 | 1 | 0 | 0 | 6 |
| Hurwitz 2017 | 1 | 1 | 1 | 0 | 1 | 1 | 0 | 1 | 6 |
| Banajeh 2009 | 1 | 1 | 1 | 0 | 1 | 1 | 1 | 1 | 7 |
| Badawi 2017 | 1 | 1 | 1 | 1 | 1 | 1 | 1 | 1 | 8 |
| Vo 2018 | 1 | 1 | 1 | 1 | 2 | 1 | 0 | 1 | 8 |
| Mathias 2017 | 1 | 1 | 1 | 1 | 0 | 1 | 0 | 1 | 6 |

The Newcastle-Ottawa Scale (NOS) for assessing the quality of non-randomised studies in meta-analyses. Available at: http://www.ohri.ca/programs/clinical_epidemiology/oxford.asp.



For peer review only - http://bmjopen.bmj.com/site/about/guidelines.xhtml

**Additional Table 3B Newcastle Ottawa study quality scoring system (case-control studies)**

| Study | SELECTION | | | | COMPARABILITY | EXPOSURE | | | Number of stars (out of 9 total) |
|---|---|---|---|---|---|---|---|---|---|
| | Is the case definition adequate? | Representativeness of the cases | Selection of controls | Definition of controls | Comparability of cases and controls on the basis of the design or analysis (maximum 2 stars) | Ascertainment of exposure | Same method of ascertainment for cases and controls (yes or no or 0 star if no mention) | Non-response rate | |
| Wayse 2004 | 1 | 1 | 0 | 0 | 1 | 1 | 1 | 1 | 6 |
| Karatekin 2009 | 1 | 1 | 0 | 0 | 1 | 1 | 1 | 1 | 6 |
| Roth 2009 | 1 | 1 | 0 | 1 | 1 | 1 | 1 | 0 | 7 |
| Roth 2010 | 1 | 1 | 1 | 1 | 2 | 1 | 1 | 0 | 8 |
| Cetinkaya 2015 | 1 | 1 | 0 | 0 | 0 | 1 | 1 | 1 | 6 |
| Onwuneme 2015 (1) | 1 | 1 | 0 | 1 | 2 | 1 | 1 | 1 | 8 |
| Cizmeci 2015 | 1 | 1 | 0 | 0 | 2 | 1 | 1 | 1 | 7 |
| Korwutthikulrangsri 2015 | 1 | 1 | 0 | 0 | 0 | 1 | 1 | 1 | 5 |
| Elmoneim 2016 | 1 | 1 | 0 | 0 | 0 | 1 | 1 | 1 | 5 |
| Narang 2016 | 1 | 1 | 0 | 0 | 1 | 1 | 1 | 1 | 6 |
| Dinlen 2016 | 1 | 1 | 0 | 0 | 1 | 1 | 1 | 1 | 6 |
| Ahmed 2015 | 1 | 1 | 0 | 1 | 2 | 1 | 1 | 1 | 8 |
| Cayir 2014 | 1 | 1 | 0 | 0 | 2 | 1 | 1 | 1 | 7 |
| Say 2017 | 1 | 1 | 0 | 0 | 2 | 1 | 1 | 0 | 6 |
| Basha 2014 | 1 | 1 | 0 | 1 | 2 | 1 | 1 | 0 | 7 |
| Jia 2017 | 1 | 1 | 0 | 1 | 1 | 1 | 1 | 1 | 7 |
| Jat 2016 | 1 | 1 | 0 | 1 | 2 | 1 | 1 | 1 | 8 |
| Moreno-Solis 2015 | 1 | 1 | 0 | 1 | 1 | 1 | 0 | 1 | 6 |
| Sakka 2014 | 1 | 1 | 0 | 1 | 2 | 1 | 1 | 1 | 8 |
| Hebbar 2014 | 1 | 1 | 0 | 0 | 1 | 1 | 1 | 1 | 6 |
| Rey 2014 | 1 | 1 | 1 | 1 | 1 | 1 | 1 | 1 | 8 |
| Ponnarmeni 2016 | 1 | 1 | 0 | 0 | 0 | 1 | 1 | 1 | 6 |
| El-Gamasy 2017 | 1 | 1 | 1 | 0 | 2 | 1 | 1 | 0 | 7 |
| Khakshour 2015 | 1 | 1 | 0 | 0 | 1 | 1 | 1 | 0 | 5 |
| Dhandai 2018 | 1 | 1 | 0 | 0 | 1 | 1 | 1 | 1 | 6 |
| Li 2018 | 1 | 1 | 1 | 1 | 2 | 1 | 1 | 0 | 8 |

The Newcastle-Ottawa Scale (NOS) for assessing the quality of non-randomised studies in meta-analyses. Available at: http://www.ohri.ca/programs/clinical_epidemiology/oxford.asp.

For peer review only



For peer review only - http://bmjopen.bmj.com/site/about/guidelines.xhtml

**Additional Table 3C Newcastle Ottawa study quality scoring system (cross-sectional studies)**

| | SELECTION (Maximum 5 stars) | | | | COMPARABILITY (Maximum 2 stars) | OUTCOME (Maximum 3 stars) | | |
|---|---|---|---|---|---|---|---|---|
| **Study** | **Representativeness of the sample** | **Sample size** | **Non-respondents** | **Ascertainment of the exposure (risk factor)** | **Subjects in different outcome groups are comparable, based on the study design or analysis. Confounding factors are controlled.** | **Assessment of the outcome (max=2 stars)** | **Statistical test (max=1 star)** | **Number of stars (out of 10 total)** |
| Yaghmaie 2017 | 1 | 0 | 0 | 0 | 1 | 1 | 1 | 4 |
| Alvarez 2016 | 1 | 0 | 0 | 1 | 1 | 2 | 1 | 6 |
| Alonso 2015 | 1 | 0 | 0 | 1 | 1 | 1 | 1 | 5 |
| Binks 2014 | 1 | 0 | 0 | 1 | 1 | 2 | 1 | 6 |

The Newcastle-Ottawa Scale (NOS) for assessing the quality of non-randomised studies in meta-analyses. Available at: http://www.ohri.ca/programs/clinical_epidemiology/oxford.asp.

For peer review only



For peer review only - http://bmjopen.bmj.com/site/about/guidelines.xhtml

**Additional Table 4A Circulating 25(OH)D threshold levels used in the selected studies**

| Study | Number of cases | 25(OH)D categories (as given) | Number of cases in each category |
|---|---|---|---|
| Asilioglu 2017 | 250 | Deficiency: <20 ng/mL | 120 |
| | | Sufficiency: >=20 ng/mL | 85 |
| Halwany 2017 | 102 | Deficiency: <20 ng/ml | 38 |
| | | Insufficiency: 20-29.9 ng/ml | 39 |
| | | "Normal" levels: 30 ng/ml | 25 |
| Hurwitz 2017 | 90 | Deficiency: <20 ng/mL | 11 |
| Garcia-Soler 2017 | 340 | Deficiency: <20 ng/mL | 149 |
| | | Insufficiency: 20-30 ng/mL | 128 |
| | | 25(OH)D levels: 30-40 ng/mL | 41 |
| | | Optimal levels >40 ng/mL | 22 |
| Badawi 2017 | 88 | Deficiency: < 50nmol/l | 39 |
| | | Severe deficiency: <30 nmol/l | 30 |
| Jia 2017 | 110 | Severe deficiency: <10 ng/ml | 36 |
| | | Deficiency: 10-20 ng/ml | 56 |
| | | Insufficiency: 21-30 ng/ml | 17 |
| | | Sufficiency: >30 ng/ml | 1 |
| Yaghmaie 2017 | 82 | Deficiency: <30 ng/ml | 53 |
| | | Sufficiency: >=30 ng/ml | 29 |
| Say 2017 | 100 | Severe deficiency (group 1) <5 ng/mL. | 63 |
| | | Insufficiency (group2): 5 to 15 ng/mL | 24 |
| | | Sufficiency (group 3) ≥15 ng/mL | 13 |
| El-Gamasy 2017 | 80 | Serious deficiency: < 30 nmol/L | 26 |
| | | Insufficiency: 30-75 nmol/L | 27 |
| | | Adequate levels >75 nmol/L | 27 |
| Sankar 2017 | 43 | Severe deficiency: serum 25 (OH) D <10 ng/mL | 31 |
| Shah 2016 | 154 | 25(OH)D < 20 ng/mL | 128 |
| | | 25(OH)D ≥20 ng/mL | 26 |
| Ponnarmeni 2016 | 124 | Deficiency: < 50nmol/L | 63 |
| | | Insufficiency: 50-75 nmol/L | 31 |
| | | <75, insufficient and deficient | 94 |
| | | Sufficiency: >75 nmol/L | 30 |
| Sankar 2016 | 101 | Deficiency: ≤ 20 ng/mL | 75 |
| | | Severe deficiency: <15 ng/mL | 62 |
| | | 'No deficiency': > 20 ng/mL | 26 |
| Bustos 2016 | 90 | Deficiency: <20 ng/ml | 39 |
| | | Normal levels: >20 ng/ml | 51 |
| Ebenezer 2016 | 52 | Deficiency: < 20ng/mL | 21 |
| | | Insufficiency: 20–30 ng/ml | 12 |
| | | 'Normal levels: ≥ 20 ng/L | 31 |
| Elmoneim 2016 | 21 | Deficiency: < 20 ng/mL | 17 |
| | | Insufficiency: 20-30 ng/mL | 4 |
| | | Normal levels: > 30 ng/mL | 9 |
| Jat 2016 | 50 | Deficiency: <20 ng/ml | 42 |
| | | Insufficiency: 20-30 ng/ml | 2 |
| | | Sufficiency: >=30 ng/ml | 1 |
| Narang 2016 | 50 | "Severe deficiency": <20ng/ml | 28 |
| Dinlen 2016 | 30 | Deficiency: <=15 ng/mL | 26 |
| | | Severe deficiency: <=5 ng/mL | 3 |
| Lopez 2016 | 347 | 25-OHD levels: <10 ng/mL | 19 |
| | | 25-OHD levels: 10-20 ng/mL | 65 |
| | | 25-OHD levels: 20-30 ng/mL | 134 |
| | | | |
| Alvarez 2016 | 50 | 25(OH) D levels: <20 ng/mL | 29 |
| | | 25(OH) D levels: 20-30 ng/mL | 37 |
| Onwuneme 2015 (1) | 120 | 25(OH)D levels: <50nmol/L | 71 |
| | | 25(OH)D levels: ≥50nmol/L | 49 |
| Onwuneme 2015 (2) | 94 | 25(OH)D levels: <20 ng/mL | 86 |
| | | 25(OH)D levels: < 30 nmol/L | 60 |
| | | 25(OH)D levels: >=30 nmol/L | 34 |
| Prasad 2015 | 80 | Deficiency: < 20 ng/ml | 67 |
| | | Sufficiency: ≥ 20 ng/mL | 13 |
| Moreno-Solis 2015 | 48 | Deficiency: <20 ng/ml | 9 |



For peer review only - http://bmjopen.bmj.com/site/about/guidelines.xhtml

| | | | |
|---|---|---|---|
| | | Insufficiency: 21–29 ng/ml | 16 |
| | | Sufficiency: ≥30 ng/ml | 23 |
| Alonso 2015 | 288 | Deficiency:  <20 ng/ml | 45 |
| | | <10 ng/ml | 6 |
| Korwutthikulrangsri 2015 | 32 | Deficiency: < 20 ng/ml | 25 |
| | | Insufficiency: 20-29.9 ng/ml | 7 |
| | | Sufficiency: ≥ 30 ng/mL | 0 |
| Khakshour 2015 | 37 | Deficiency: <20 ng/mL | 9 |
| Cizmeci 2015 | 40 | Deficiency: ≤20 ng/ml | 28 |
| | | Insufficiency: 21–29 ng/ml | 7 |
| | | Normal levels: ≥30 ng/ml | 5 |
| Cetinkaya 2015 | 50 | Severe deficiency: <10 ng $ml^{-1}$ | 42 |
| | | Insufficiency: 11 to 32 ng $ml^{-1}$ | 8 |
| Ayulo 2014 | 216 | Deficient: < 15 ng/ml | 61 |
| | | Insufficient: 15-29 ng/mL | 102 |
| | | Sufficient: ≥ 30 ng/mL | 53 |
| Dayal 2014 | 92 | Deficiency:  < 50 nmol/L | 23 |
| | | Insufficiency: 50–75 nmol/L | 41 |
| | | Sufficiency:  > 75 nmol/L | 28 |
| | | 25(OH) D levels: < 75 nmol/L | 64 |
| | | 'Non-deficiency': > 50 nmol/L | 69 |
| Hebbar 2014 | 61 | Deficiency: ≤ 10 ng/mL | 10 |
| | | Insufficiency: 10 to 20 ng/mL | 27 |
| | | Sufficiency: ≥ 20 ng/mL | 24 |
| Rey 2014 | 156 | Deficiency: < 20 ng/mL | 46 |
| | | 25(OH)D levels: ≥ 20 ng/mL | 110 |
| Ahmed 2014 | 50 | Deficiency: <50 nmol/L | 15 |
| Basha 2014 | 81 | Deficiency: <50 nmol/L | 53 |
| | | Insufficiency: <75 nmol/L | 14 |
| | | Normal level: >75 nmol/L | 14 |
| Sakka 2014 | 96 | Severe deficiency: 0–5 ng/mL | 29 |
| | | Deficiency: 5–15 ng/mL | 49 |
| | | Insufficiency: 15– 20 ng/mL | 11 |
| | | Sufficiency: 20–100 ng/mL | 7 |
| Cayir 2014 | 88 | Normal levels:  20 ng/mL | 38 |
| | | Insufficiency: 15-20ng/mL | 18 |
| | | Deficiency: <15ng/mL | 32 |
| Binks 2014 | 74 | 25(OH) D levels: < 50 nmol/L | 11 |
| Madden 2012 | 511 | 25(OH) D levels: < 10 ng/mL | 36 |
| | | 25(OH) D levels: 10-19.9 ng/mL | 169 |
| | | Deficiency: < 20 ng/ml | 205 |
| | | Insufficiency: <30 ng/mL | 364 |
| Rippel 2012 | 316 | Deficiency: < 50 nmol/L | 109 |
| | | 'Normal levels': ≥50 nmol/L | 207 |
| Madden 2012 | 511 | 25(OH)D levels: < 10 ng/mL | 36 |
| | | 25(OH)D levels: 10-19.9 ng/mL | 169 |
| | | Deficiency: < 20 ng/mL | 205 |
| | | Insufficiency: <30 ng/mL | 364 |
| McNally 2012 | 326 | Deficiency: < 50 nmol/L | 225 |
| | | 25(OH)D levels: 50 to 75 nmol/L | 75 |
| | | 'Not deficient': > 50 nmol/L | 101 |
| Inamo 2011 | 28 | 25(OH)D levels: < 10 ng/mL | 4 |
| | | 25(OH)D levels: < 15 ng/mL | 8 |
| | | 25(OH)D levels: < 25 ng/mL | 12 |
| | | 25(OH)D levels: < 40 ng/mL | 28 |
| Roth 2010 | 25 | 25(OH)D levels:  < 40 nmol/L | 21 |
| Banajeh 2009 | 79 | Deficiency: <30 nmol/L | 29 |
| Karatekin 2009 | 25 | Serum 25(OH)D <10 (deficiency) | 19 |
| | | Serum 25(OH)D 11 to 20 (deficiency) | 4 |
| | | Serum 25(OH)D 21 to 32 (insufficiency) | 1 |

For peer review only



For peer review only - http://bmjopen.bmj.com/site/about/guidelines.xhtml

| | | | |
|---|---|---|---|
| | | Serum 25(OH)D 32 to 100 (sufficiency) | 1 |
| Roth 2009 | 64 | < 40 nmol/L | 3 |
| Wayse 2004 | 80 | Plasma 25(OH)D3 > 22.5 nmol/L | 26 |
| | | Plasma 25(OH)D3 > 50 nmol/L | 4 |
| Mathias 2017 | 41 | 25(OH)D < 20 ng/ml | 28 |
| | | 25(OH)D < 30 ng/ml | 36 |
| Dhandai 2018 | 60 | Deficiency: < 20 ng/ml | 38 |
| | | Insufficiency: <29 ng/ml | 17 |
| | | Optimum: 30-50 ng/ml | 5 |
| Vo 2018 | 1016 | 25(OH)D < 20 ng/ml | 298 |
| | | 20-29.9 ng/ml | 352 |
| | | >=30 ng/ml | 366 |
| Li 2018 | 797 | Severe deficiency: < 10 ng/mL | 159 |
| | | Deficiency: 10–20 ng/mL | 286 |
| | | Insufficiency: 20– 30 ng/mL | 223 |
| | | Sufficiency: > 30 ng/mL | 127 |

25(OH)D in nmol/L = 25(OH)D in ng/mL multiplied by 2.496

For peer review only



For peer review only - http://bmjopen.bmj.com/site/about/guidelines.xhtml

**Additional Table 4B Circulating 25(OH)D threshold levels used in the selected studies for prevalence in sepsis**

| Study | Number of cases | 25(OH)D categories (as provided by each study) | Number of cases |
|---|---|---|---|
| Asilioglu 2017 | 30 sepsis | Deficiency: <20 ng/mL | 20 |
| | | Sufficiency: >=20 ng/mL | 10 |
| Say 2017 | 100 neonatal sepsis | Severe deficiency (group 1) <5 ng/mL. | 63 |
| | | Insufficiency (group2): 5 to 15 ng/mL | 24 |
| | | Sufficiency (group 3) ≥15 ng/mL | 13 |
| El-Gamasy 2017 | 46 sepsis | Serious deficiency: < 30 nmol/L | 20 |
| | | Insufficiency: 30-75 nmol/L | 18 |
| | | Adequate levels >75 nmol/L | 8 |
| Sankar 2017 | 43 septic shock | Severe deficiency: serum 25 (OH) D <10 ng/mL | 31 |
| Shah 2016 | 100 sepsis | 25(OH)D <20 ng/mL | 84 |
| | | 25(OH)D >=20 ng/mL | 26 |
| Ponnarmeni 2016 | 124 sepsis | Deficiency: <50nmol/L | 63 |
| | | Insufficiency: 50-75 nmol/L | 31 |
| | | <75, insufficient + deficient | 94 |
| | | Sufficiency: >75 nmol/L | 30 |
| Bustos 2016 | 10 sepsis intraabdominal | Deficiency: < 20 ng/ml | 7 |
| | | Normal levels: > 20 ng/mL | 3 |
| Ebenezer 2016 | 16 shock | 25(OH)D <20 ng/mL | 8 |
| | | 25(OH)D >=20 ng/mL | 8 |
| Onwuneme 2015 (1) | 35 culture positive sepsis | 25(OH)D <50 ng/mL | 32 |
| | | 25(OH)D >=50 ng/mL | 3 |
| Onwuneme 2015 (2) | 46 culture positive sepsis and late-onset sepsis | Deficiency: < 30 nmol/L | 32 |
| | | Sufficiency: ≥ 30 nmol/L | 14 |
| Prasad 2015 | 11 positive blood culture | 25(OH)D <20 ng/mL | 9 |
| | | 25(OH)D >=20 ng/mL | 2 |
| Korwutthikulrangsri 2015 | 17 shock and septicaemia | 25(OH)D <20 ng/mL | 14 |
| | | 25(OH)D >=20 ng/mL | 3 |
| Cizmez 2015 | 40 suspected sepsis | Deficiency: ≤20 ng/ml | 28 |
| | | Insufficiency: 21–29 ng/ml | 7 |
| | | Normal levels: ≥30 ng/ml | 5 |
| Dayal 2014 | 9 nosocomial sepsis | 25(OH)D <20 ng/mL | 4 |
| | | 25(OH)D >=20 ng/mL | 5 |
| Hebbar 2014 | 30 shock and/or Sepsis | 25(OH)D <20 ng/mL | 17 |
| | | 25(OH)D >=20 ng/mL | 13 |
| McNally2012 | 48 septic | 25(OH) D levels: <50 nmol/L | 33 |
| | | 25(OH) D levels: ≥50 nmol/L | 15 |
| Mathias 2017 | 41 sepsis, severe sepsis or septic shock | 25(OH)D < 20 ng/ml | 28 |
| | | 25(OH)D < 30 ng/ml | 36 |
| Dhandai 2018 | 60 sepsis | Deficiency: < 20 ng/ml | 38 |
| | | Insufficiency: <29 ng/ml | 17 |
| | | Optimum: 30-50 ng/ml | 5 |

For peer review only



For peer review only - http://bmjopen.bmj.com/site/about/guidelines.xhtml

**Additional Table 5 Studies with thresholds other than < 50 nmol/L**

| Study | Threshold used by study |
|---|---|
| Roth 2009 | < 40 nmol/L |
| Roth 2010 | < 40 nmol/L |
| Say 2017 | <=15 ng/mL (37.4 nmol/L) |
| Inamo 2011 | <=15 ng/mL (37.4 nmol/L) |
| Ayulo 2014 | <=15 ng/mL (37.4 nmol/L) |
| Dinlen 2016 | <=15 ng/mL (37.4 nmol/L) |
| Onwuneme 2015 (2) | < 30 nmol/L |
| Yaghmaie 2017 | < 30 ng/mL (74.88 nmol/L) |
| El-Gamasy 2017 | < 30 nmol/L |
| Banajeh 2009 | < 30 nmol/L |
| Sankar 2017 | <= 10 ng/mL (24.9 nmol/L) |
| Cetinkaya 2015 | <= 10 ng/mL (24.9 nmol/L) |

25(OH) D values nmol/L = ng/mL * 2.496

For peer review only



For peer review only - http://bmjopen.bmj.com/site/about/guidelines.xhtml

**Additional Table 6 Excluded studies**

| Study | Design | Sample size | Characteristics of pediatric population | Country | Reasons for exclusion of paper |
|---|---|---|---|---|---|
| Seliem 2016 | Hospital-based case-control | Cases, n= 30<br>Controls, n= 30 | Cases: group 1, full-term neonates with EOS and their mothers.<br>Controls, group 2: 30 full-term healthy neonates and their mothers with no clinical or laboratory evidence of sepsis. | Egypt | Requested data without reply or data not available |
| Gamal 2017 | Case-control | Cases, n= 50<br>Control, n= 30 | Cases: neonates with early onset neonatal sepsis that occurred at <72 hours<br>Controls: Age and sex healthy neonates with no prenatal risk factor for early neonatal sepsis enrolled in the study as a control group. | Egypt | Requested data without reply or data not available |
| Aydemir 2014 | Hospital-based case control | Cases, n=40<br>Controls, n= 20 | Cases: children with sepsis between 1 and 16 years old<br>Controls: children without sepsis | Turkey | Requested data without reply or data not available |
| Garg 2016 | Hospital-based case-control | Cases, n= 40<br>Controls, n= 40 | Cases: children from 6 months to 5 years of age admitted or attending OPD in department of Pediatrics.<br>Controls: children receiving care at the Hospital's ambulatory, emergency or in-patient units and presenting with non-respiratory complaints also not having any clinical indication of vitamin D deficiency. | India | Requested data without reply or data not available |



For peer review only - http://bmjopen.bmj.com/site/about/guidelines.xhtml

**Additional Table 7 Characteristics of the 52 included studies**

| Study | Design | Number of cases (controls, where appropriate) | Characteristics of population | Country and setting | Vitamin D thresholds as defined by the study | Quality score (NOS) |
|---|---|---|---|---|---|---|
| Asilioglu 2017 | Historical cohort (single centre) | 250 | Cases: aged 1 month to ≤18 years | Turkey, PICU | Deficiency: <20 ng/mL | 7 |
| Halwany 2017 | Cohort (single centre) | 102 | Cases: children aged >1 month to ≤ 5 years | Egypt, PICU | Deficiency: <20 ng/ml<br>Insufficiency: 20-29.9 ng/ml<br>“Normal” levels: 30 ng/ml | 6 |
| Hurwitz 2017 | Prospective cohort (single centre) | 90 | Cases: aged <5 years hospitalized with LRTI and RSV and/or hMPV | USA, PICU | Deficiency: <20 ng/mL | 6 |
| Garcia-Soler 2017 | Cohort (single centre) | 340 | Cases: aged 6 months to 17 years<br>Critically ill with various conditions | Spain, PICU | Deficient: <20 ng/mL<br>Insufficiency: 20-30 ng/mL<br>25(OH)D levels: 30-40 ng/mL<br>Optimal levels >40 ng/mL | 7 |
| Badawi 2017 | Cohort (single centre) | 88 | Cases: 1 month to 12 years | Egypt, PICU | Deficiency: < 50nmol/l,<br>Severe deficiency: <30 nmol/l | 8 |
| Jia 2017 | Hospital-based case-control (single centre) | 110 (110) | Cases: infants (< 1 year of age) with ALRTI<br>Controls: Healthy from similar areas attending the hospital for vaccination during study period | China, PICU | Severe deficiency: <10 ng/ml<br>Deficiency: 10-20 ng/ml<br>Insufficiency: 21-30 ng/ml<br>Sufficiency: >30 ng/ml | 7 |
| Yaghmaie 2017 | Cross sectional (single centre) | 82 | Cases: hospitalized in PICU | Iran, PICU | Deficiency: <30 ng/ml | 4 |
| Say 2017 | Case-control (single centre) | 100 (13) | Cases: premature infants less than 37 weeks diagnosed with early or late -onset neonatal sepsis<br>Controls: From same population of neonates with sepsis but not vitamin D deficient | Turkey, NICU | Severe deficiency (group 1) <5 ng/mL.<br>Insufficiency (group2): 5 to 15 ng/mL<br>Sufficiency (group 3) ≥15 ng/mL | 6 |

For peer review only

| | | | | | | |
|---|---|---|---|---|---|---|
| El-Gamasy 2017 | Case-control (single centre) | 80 (20) | Cases: 3 months to 12 years hospitalized with acute kidney injury<br>Controls: completely healthy subjects | Egypt, PICU | Seriously deficient: < 30 nmol/L<br>Insufficient: 30-75 nmol/L<br>Adequate >75 nmol/L | 7 |
| Sankar 2017 | Cohort (single centre) | 43 | Cases: <=17 years of age | India, PICU | Severe vitamin D deficiency: serum 25 (OH) D <10 ng/mL | 8 |
| Shah 2016 | Cohort (single centre) | 154 | Cases: aged between 1 month and 15 years | India, PICU | Deficiency: <20 μg/mL | 6 |
| Ponnarmeni 2016 | Case-control (single centre) | 124 (338) | Cases: aged 1–12 years admitted with a diagnosis of sepsis<br>Controls: from previous prospective study, apparently healthy children of upper socioeconomic status who attended the out-patient department for immunization or with minor ailments | India, PICU | Deficiency: < 50 nmol/L<br>Insufficiency: 50-75 nmol/L<br>Sufficiency: > 75 nmol/L | 6 |
| Sankar 2016 | Cohort (single centre) | 101 | Cases: aged 1 month to 17 years | India, PICU | Deficiency: ≤ 20 ng/ml<br>Severe deficiency: <15 ng/mL | 8 |
| Bustos 2016 | Cohort (single centre) | 90 | Cases: critically ill, greater than 37 weeks and less than 15 years of age | Chile, PICU | Deficiency: < 20 ng/ml<br>Normal levels: > 20 ng/mL | 7 |
| Ebenezer 2016 | Cohort (single centre) | 52 | Cases: <18 years of age; medical and surgical diagnoses | India, PICU | Deficiency: < 20 ng/ml<br>Insufficiency: 20–30 ng/ml<br>Normal levels: ≥ 20 ng/L | 6 |
| Elmoneim 2016 | Case-control (single centre) | 30 | Cases: aged less than 14 years<br>Controls: with “normal” vitamin D levels | Saudi Arabia, PICU | Deficiency: < 20ng/ml<br>Insufficiency: 20-30 ng/mL<br>Normal: > 30 ng/mL | 5 |
| Jat 2016 | Hospital-based case-control (single centre) | 50 (50) | Cases: 1 month to 12 years of age admitted with pneumonia<br>Controls: admitted for reasons other than respiratory symptoms, required blood sampling | India, PICU | Deficiency: < 20 ng/ml<br>Insufficiency: 20-30 ng/ml Sufficiency: >= 30 ng/ml | 8 |
| Narang 2016 | Hospital-based case-control (single centre) | 50 (50) | Cases: 2 months to 5 years of age admitted as in-patients or seen in the outpatient department with ALRI<br>Controls: healthy, same age group, attending outpatients’ service for immunization or admitted for minor conditions other than ALRI | India, PICU | Severe deficiency: < 20ng/ml | 6 |



For peer review only - http://bmjopen.bmj.com/site/about/guidelines.xhtml

For peer review only

| | | | | | | |
|---|---|---|---|---|---|---|
| Dinlen 2016 | Hospital-based case-control (single centre) | 30 (30) | Cases: term neonates with ALRI<br>Controls: healthy neonates, same age as the study group. | Turkey, NICU | Deficient: <= 15 ng/mL<br>Severe deficiency: <= 5 ng/mL | 6 |
| Lopez 2016 | Prospective cohort (multi centre) | 347 | Cases: 0 to >48 months admitted to hospital with ALRI prospectively recruited through the GENDRES (GENetic,vitamin D and RESpiratory infections research network) | Spain, PICU | 25-OHD levels: <10 ng/mL<br>25-OHD levels: 10-20 ng/mL<br>25-OHD levels: 20-30 ng/mL | 8 |
| Alvarez 2016 | Cross-sectional (single centre) | 50 | Cases: patients aged 0 to 18 years | USA, PICU | 25(OH)D < 20 ng/mL<br>25(OH)D < 30 ng/mL | 6 |
| Onwuneme 2015 (1) | Case-control (single centre) | 120 (30) | Cases: with suspected sepsis (<12 years old)<br>Controls: paediatric controls admitted for elective day case surgery during the same study period and were not suspected of having sepsis | Ireland , PICU | 25(OH) D levels: <50 nmol/L<br>25(OH) D levels: ≥50 nmol/L | 8 |
| Onwuneme 2015 (2) | Cohort (single centre) | 94 | Cases: preterm infants <32 weeks gestation | Ireland, NICU | Deficiency: < 30 nmol/L<br>Sufficiency: ≥ 30 nmol/L | 8 |
| Prasad 2015 | Cohort (single centre) | 80 | Cases: 2 months to 12 years old | India, PICU | Deficiency: < 20 ng/ml<br>Sufficient: ≥ 20 ng/mL | 7 |
| Moreno-Solis 2015 | Hospital-based case-control (single centre) | 48 (30) | Cases: aged 1–11 months with acute bronchiolitis<br>Controls: healthy, <12 months, admitted to the outpatient clinic without respiratory symptoms or history of hospitalization for bronchiolitis or wheezing | Spain, PICU | Deficiency: < 20 ng/ml<br>Insufficiency: 21–29 ng/ml Sufficiency: ≥ 30 ng/ml | 6 |
| Alonso 2015 | Cross sectional (single centre) | 288 | Cases: aged 1 month to 13 years | Spain, PICU | Deficient: < 20 ng/ml and < 10 ng/ml | 5 |
| Korwutthikulrangsri M 2015 | Nested case-control (single centre) | 32 (36) | Cases: requiring PICU admission<br>Controls: Healthy, enrolled during the same period of time and served as the control group (age in months) | Thailand, PICU | Deficiency: < 20 ng/mLl<br>Insufficiency: 20-29.9 ng/ml<br>Sufficiency: ≥ 30 ng/mL | 5 |
| Khakshour 2015 | Case-control | 37 (53) | Cases: below 5 years of age and suffering from respiratory infections<br>Controls: those who were not suffering from respiratory infections | Iran, PICU | Deficiency: < 20 ng/mL | 5 |



For peer review only - http://bmjopen.bmj.com/site/about/guidelines.xhtml

| | | | | | | |
|---|---|---|---|---|---|---|
| Cizmeci 2015 | Case-control (single centre) | 40 (43) | Cases: infants with suspected early-onset neonatal sepsis.<br>Controls: For each newborn of group 1, one healthy infant selected as a control | Turkey, NICU | Deficiency: ≤ 20 ng/ml<br>Insufficiency: 21–29 ng/ml<br>Normal levels: ≥ 30 ng/ml | 7 |
| Cetinkaya 2015 | Hospital-based case-control (single centre) | 50 (50) | Cases: term infants with clinically suspected (probable) early infection (early-onset sepsis) within the first 3 postnatal days of life and were >37 weeks of gestational age<br>Controls: healthy infants with no signs of clinical/laboratory infection | Turkey, NICU | Severe deficiency: <10 ng $ml^{-1}$<br>Insufficiency: 11 to 32 ng $ml^{-1}$<br>Adequacy: 32 to 100 ng $ml^{-1}$ | 6 |
| Ayulo 2014 | Cohort (single centre) | 216 | Cases: between the ages of 1 and 21 years | Spain, PICU | Deficient: < 15 ng/ml<br>Insufficient: 15-29 ng/mL<br>Sufficient: ≥ 30 ng/mL | 7 |
| Dayal 2014 | Cohort (single centre) | 92 | Cases: Children aged 3 months to 12 years | India, PICU | Deficiency: < 50 nmol/L<br>Insufficiency: 50–75 nmol/L<br>Sufficiency: > 75 nmol/L<br>25(OH) D levels: < 75 nmol/L | 6 |
| Hebbar 2014 | Case-control (single centre) | 61 (46) | Cases: children 0 to 18 years<br>Controls: patients recruited among children in the magnetic resonance imaging suite. | USA, PICU | Deficient: ≤ 10 ng/ml<br>Insufficient: 10 to 20 ng/ml<br>Sufficient: ≥ 20 ng/mL | 6 |
| Rey 2014 | Case-control (single centre) | 156 (289) | Cases: heterogeneous group of critically ill children aged <16 years<br>Control group for comparison: population of healthy children | Spain, PICU | Deficient: < 20 ng/ml | 8 |
| Ahmed 2014 | Hospital-based case-control (single centre) | 50 (50) | Cases: aged 2–60 months hospitalized with ALRI<br>Controls: age-matched with cases within 1 or 2 months, attending well-child clinics or general clinics without evidence of respiratory infection or admitted to the hospital for elective surgery | Africa, PICU | Deficiency: <50 nmol/L | 8 |
| Basha 2014 | Hospital-based case-control (single centre) | 81 (89) | Cases: under 5 years old with severe pneumonia selected from the inpatient departments and emergency units of the hospital<br>Controls: healthy, selected from the outpatient surgical clinics of the hospital during their visit for umbilical or inguinal hernia repair and not suffering | Egypt, PICU | Deficiency: < 50 nmol/L<br>Insufficiency: < 75 nmol/L<br>Sufficiency: > 75 nmol/L | 7 |



For peer review only - http://bmjopen.bmj.com/site/about/guidelines.xhtml

| | | | | | | |
|---|---|---|---|---|---|---|
| | | | from upper or lower respiratory infections | | | |
| Sakka 2014 | Population based case-control (single centre) | 96 (96) | Cases: <2 years old with ALRI, 48 diagnosed with pneumonia and 48 with bronchiolitis<br>Controls: age and sex matched with no respiratory symptoms or signs from the Health office | Egypt, PICU | Severe deficiency: 0–5 ng/mL Deficiency: 5–15 ng/mL Insufficiency: 15– 20 ng/mL Sufficiency: 20–100 ng/mL | 8 |
| Cayir 2014 | Hospital-based case-control (single centre) | 88 (81) | Cases: 1 to 13 years diagnosed with acute otitis media<br>Controls: Healthy same age range | Turkey, PICU | Normal levels: 20 ng/mL<br>Insufficiency: 15-20ng/mL<br>Deficiency: <15ng/mL | 7 |
| Binks 2014 | Cross-sectional | 74 | Cases: aged <3 years admitted with acute lower respiratory infections (ALRIs) or other conditions | Australia, PICU | 25(OH) D levels: < 50 nmol/L | 6 |
| Madden 2012 | Cohort (single centre) | 511 | Cases: less than 21 years old | USA, PICU | 25(OH) D levels: < 10 ng/mL<br>25(OH) D levels: 10-19.9 ng/mL<br>Deficiency: < 20 ng/ml<br>Insufficiency: < 30 ng/mL | 8 |
| Rippel 2012 | Cohort (single centre) | 316 | Cases: children aged 16.5 (3.1–75.2) months | Australia, PICU | Deficiency: < 50 nmol/L<br>"Normal" levels: ≥ 50 nmol/L | 8 |
| McNally 2012 | Cohort (multi-centre) | 326 | Cases: Newborn to 17 years of age | Canada, PICUs | Deficiency: < 50 nmol/L<br>25(OH) D levels:<br>50 to 75 nmol/L | 7 |
| Inamo 2011 | Cohort (single centre) | 28 | Cases: between 1 and 48 months hospitalized with ALRI, 26 diagnosed with bronchiolitis and two as having pneumonia | Japan, PICU | Deficiency: ≤ 15ng/ml<br>Severe deficiency:<br>≤ 5ng/mL | 6 |
| Roth 2010 | Hospital-based case-control (single centre) | 25 (25) | Cases: 1–18 months hospitalized with ALRI<br>Controls: selected by population-based sampling. aged 1–23 months, and matched to cases on age (±2 months) and sex | Bangladesh, PICU | Deficiency: < 40 nmol/L | 8 |
| Banajeh 2009 | Prospective cohort (single centre) | 79 | Cases: 2–59 months with WHO-defined very severe community acquired (VSP) pneumonia | Iran, PICU | Deficiency: < 30 nmol/L | 7 |
| Karatekin 2009 | Hospital-based case- | 25 (15) | Cases: newborns with acute respiratory infections | Turkey, NICU | Deficiency: < 20 ng/ml | 6 |

For peer review only

| | | | | | | |
|---|---|---|---|---|---|---|
| | control, (single centre) | | Controls: healthy, age matched from outpatients' service where they went for immunization. | | | |
| Roth 2009 | Hospital-based case-control (single centre) | 64 (65) | Cases: aged 1-25 months admitted with ALRI<br>Controls: aged 1-25 months undergoing elective surgery, no history of hospitalization for ALRI | Canada, PICU | Deficiency: < 40 nmol/L | 7 |
| Wayse 2004 | Hospital-based case-control, (single centre) | 80 (70) | Cases: < 5 years with severe ALRI<br>Controls: healthy, attending outpatients service for immunization | India, PICU | Deficiency: Plasma 25(OH)D3 < 50 nmol/L | 6 |
| Mathias 2017 | Prospective cohort (single centre) | 41 | Cases: less than 18 years admitted with diagnosis of sepsis, severe sepsis or septic shock. | USA, PICU | 25(OH)D levels < 20 ng/ml<br>25(OH)D levels <30 ng/ml | 6 |
| Dhandai 2018 | Case-control (multi-centre) | 60 (60) | Cases: neonates admitted with late-onset sepsis (LOS)<br>Controls: neonates admitted during same period with clinically significant physiological hyperbilirubinaemia (without sepsis) | India, NICU | Deficiency: < 20 ng/ml<br>Insufficiency: <29 ng/ml<br>Optimum: 30-50 ng/ml | 6 |
| Vo 2018 | Prospective cohort (multi-centre) | 1016 | Cases: Infants less than twelve months old hospitalized for bronchiolitis | USA, PICU | 25(OH)D < 20 ng/ml<br>25(OH)D: 20-29.9 ng/ml<br>25(OH)D >= 30 ng/ml | 8 |
| Li 2018 | Case-control (single-centre) | 797 (785) | Cases: children with pneumonia or pneumonia-induced sepsis group | China, PICU | Severe deficiency: < 10 ng/mL<br>Deficiency: 10–20 ng/mL<br>Insufficiency: 20– 30 ng/mL<br>Sufficiency: > 30 ng/mL | 8 |

LRTI = lower respiratory tract infection; PICU = paediatric intensive care unit; RSV = respiratory syncytial virus; ALRI = acute lower respiratory infection; WHO = World Health Organization; VSP = very severe community acquired pneumonia; NICU = neonatal intensive care unit.



For peer review only - http://bmjopen.bmj.com/site/about/guidelines.xhtml

**Additional Table 8 Objectives and outcomes of included studies**

| Study | Objectives/aims of study | Main outcome(s) and conclusion(s) |
|---|---|---|
| Madden 2012 | Prevalence of vitamin D deficiency in critically ill children and factors influencing admission 25-hydroxyvitamin D (25(OH)D) levels | Identified high prevalence of vitamin D deficiency and insufficiency in critically ill children. Inverse association severity of illness on admission and 25(OH) levels |
| Lopez 2016 | Role of Vitamin D in Children hospitalized with Lower Tract Acute Respiratory Infections | 25-hydroxyvitamin D levels of study population below normal range. Correlation of higher disease severity with lower levels of 25-hydroxyvitamin D. |
| Garcia-Soler 2017 | Prevalence and risks factors of vitamin D deficiency, as well as its relationship with length of PICU stay morbidity and mortality in a PICU. | Vitamin D deficiency is frequent in paediatric critical patients. It also has an association with higher severity scores, season of year and parental educational attainment.<br>Vitamin D levels associated with various laboratory parameters of SIRS. Vitamin D deficiency associated with increased risk of morbidity and mortality. Inconclusive findings on its association with PICU length of stay were inconclusive |
| McNally 2012 | Prevalence of vitamin D deficiency, risk factors and potential association with clinically relevant outcomes in critically ill children in Canada. | Most of critically ill children vitamin D deficient at PICU admission. Lower 25(OH) D levels associated with increased catecholamine requirements, fluid bolus administration, hypocalcemia, and longer PICU admission. |
| Rippel 2012 | Prevalence of hypovitaminosis D and association with outcome in critically ill children requiring admitted in intensive care. | Hypovitaminosis D is frequent in critically ill children in PICU, especially in infants and children with heart disease. Hypovitaminosis D associated with hypocalcemia in non-cardiac population, and increased need for calcium replacement in the cardiac population.<br>No association between vitamin D status and survival or PICU length of stay. Strong association with early postoperative inotropic needs in the cardiac population. |
| Alonso 2015 | Investigate relationship of serum 25-hydroxyvitamin D concentrations with serum parathyroid hormone (PTH) levels, body mass index (BMI), and environmental factors in a population of Caucasian children living at latitude 43°N. | Results doubt the assumption that a serum 25OH D threshold indicates vitamin D deficiency in children. |
| Asilioglu 2017 | Measure occurrence of VDD in critically ill children. Assess determinants of vitamin D status and compare vitamin D deficient and sufficient cases in respect of severity of illness. | Hypovitaminosis D occurrence high in critically ill children and associated with higher vasopressor requirement. Not associated with other markers of illness severity including mortality. |
| Ayulo 2014 | Prevalence of vitamin D deficiency among children in PICU | Vitamin D deficiency common. No significant correlation between disease severity and vitamin D levels levels of vitamin D. Mortality associated with vitamin D levels |
| Rey 2014 | Identify prevalence of 25 hydroxivitamin D or 25(OH) vitamin D deficiency on pediatric intensive care unit (PICU) admission, and if associated with increased prediction of mortality risk scores. | Hypovitaminosis D incidence high in PICU patients. Hypovitaminosis D not associated with higher prediction of risk mortality scores. |
| Shah 2016 | Determine prevalence of vitamin D deficiency in critically ill children its association with illness severity, parathyroid response and clinical outcomes. | High prevalence of vitamin D deficiency. Parathyroid gland response secondary vitamin D deficiency or hypocalcemia impaired in critically ill. |
| Ponnarmeni 2016 | Vitamin D deficiency in critically ill children with sepsis admitted to PICU and its association with: mortality, length of stay, illness severity, requirement for ventilation and catecholamines | High prevalence of vitamin D deficiency<br>No significant association between vitamin D deficiency and other outcomes such as mortality |
| Onwuneme 2015 (1) | Assess vitamin D status, and its determinants, in chidren with suspected sepsis admitted to PICU. Also investigated association between vitamin D status and clinical outcomes. | Children admitted to the PICU with suspected sepsis lower 25OH D compared to controls. Inadequate 25 OH D levels associated with confirmed sepsis and poor outcomes. |
| Jia 2017 | Association of vitamin D with ALRTI in Chinese infants | Lowered plasma level of 25-OH Vitamin D makes children susceptible to ALRTI. |
| Halwany 2017 | Frequency of vitamin D deficiency in critically ill pediatric intensive care unit [PICU] patients and relation to state of serum 25(OH) D to disease severity. | High prevalence of vitamin D deficiency in critically ill children.<br>Negative correlation of Vitamin D level with PELOD score. Recommend screening of critically ill children for vitamin D deficiency to restore their serum levels. |



For peer review only - http://bmjopen.bmj.com/site/about/guidelines.xhtml

| | | |
|---|---|---|
| Sankar 2016 | Prevalence of vitamin D deficiency on admission and examine association with length of ICU stay | High prevalence of vitamin D deficiency. Vitamin D deficient children with longer ICU stay duration or mortality |
| Say 2017 | Evaluate effects of low vitamin D levels in cord blood on neonatal sepsis in preterm infants. | No significant relationship between the cord blood vitamin D levels and the risk of neonatal sepsis in premature infants. |
| Sakka 2014 | Determine the relation between vitamin D deficiency, anemia and the severity of ALRTIs in hospitalized children. | Vitamin D deficiency, low BMI, low hemoglobin level, rachitic signs were risk factors for the severity of ALRTIs. |
| Onwuneme2015 (2) | Investigate the association between serum 25-hydroxyvitamin D (25OHD) levels and outcomes in preterm infants (<32 weeks gestation). | High prevalence of low 25OHD. Association between vitamin D status and acute respiratory morbidity in preterm infants after birth. In none of the following outcomes was the difference statistically significant: surfactant use, inotrope requirement, RDS, pneumothorax, pulmonary hemorrhage, chronic lung disease, sepsis, retinopathy of prematurity, intraventricular hemorrhage, periventricular leucomalacia, patent ductus arteriosus, and mortality |
| Dayal 2014 | Prevalence of vitamin D deficiency<br>Association of serum vitamin D levels with duration of stay in hospital, mortality and requirement of ventilation | Reduced serum vitamin D levels in children |
| Bustos 2016 | Determine prevalence of vitamin D deficiency and its association with other clinically relevant outcomes in children admitted to Paediatric Intensive Care Unit | Vitamin D deficiency was prevalent in critically ill children and associated with adverse clinical outcomes. |
| Hurwitz 2017 | Measure retinol binding protein and vitamin D in children aged <5 years hospitalized with lower respiratory tract infection and respiratory syncytial virus and/or human meta pneumovirus detections | Low vitamin levels in 50% of the children and associated with significantly elevated risk of the need for intensive care unit admission and invasive mechanical ventilation. |
| Cayir 2014 | Investigate the relationship between Vitamin D deficiency and acute otitis media infection | Serum 25-hydroxy vitamin D levels significantly lower in children with acute otitis media compared to the controls. Vitamin D deficiency plays a role in otitis media infection. |
| Badawi 2017 | Investigated if VDD is related to higher severity scores and organ dysfunction. Primary objective of study was to estimate the prevalence of VDD in a group of critically ill children, and secondary objectives was to correlate vitamin D status with pediatric logistic organ dysfunction (PELOD) and pediatric risk ofmortality III (PRISM III) scores. | VDD prevalence was reported in about half of the critically ill patients, and it was observed to be related to multiple organ dysfunctions and rapid clinical deterioration. |
| Yaghmaie 2017 | Investigate relation of vitamin D deficiency with potential demographic and clinical factors. | Vitamin D deficiency among paediatric intensive care unit patients similar to western countries, also with similar age and BMI distribution. Significant relation observed between age and serum level of vitamin D. |
| Basha 2014 | Aimed to evaluate vitamin D status as a risk factor for severe pneumonia in Egyptian hospitalized children under 5 years | Significant association between vitamin D deficiency and severe pneumonia in Egyptian children below 5 years |
| Prasad 2015 | Prevalence of vitamin D deficiency in critically ill children and association with illness severity& other outcomes | High prevalence of vitamin D deficiency in critically ill children and association with PRISM III scores in a developing country.<br>Groups did not differ in terms of PICU stay, duration of hospital stay, culture positivity, biochemical parameters (serum calcium, serum phosphate), need of ventilation or steroids, presence of coagulopathy and mortality. |
| Wayse 2004 | If vitamin D deficiency in Indian children under 5 years old of age is risk factor for severe acute lower respiratory infection (ALRI). | Subclinical vitamin D deficiency & nonexclusive breastfeeding in four first months of life found to be significant risk factors for severe ALRI in Indian children. |
| El-Gamasy 2017 | Assess serum 25 (OH)D level in critically ill paediatric patients with AKI at PED of Tanta University Emergency Hospital (TUEH) within the first 24 hours of admission and evaluate its correlation with duration of hospital stay and mortality outcome. | Vitamin D deficiency associated with higher incidence of sepsis and mortality. |



For peer review only - http://bmjopen.bmj.com/site/about/guidelines.xhtml

| | | |
|---|---|---|
| Banajeh 2009 | Determine if rickets and VDD predict the outcomes in very severe pneumonia (VSP). | In WHO-defined VSP, nutritional rickets was strongly associated with a reduced successful treatment outcome, and VDD was a significant and independent predictor of reduced circulating PMNs and persistent hypoxemia. |
| Binks 2014 | Aimed to determine prevalence of vitamin D insufficiency among children hospitalised with ALRI in the Northern Territory. | Vitamin D insufficiency was observed in about one-third of these hospitalised children. Children hospitalised with an ALRI less likely to have vitamin D insufficiency compared with children hospitalised for other conditions (predominantly gastroenteritis). |
| Roth 2009 | Test the hypothesis that vitamin D status is associated with the risk of ALRI in Canadian children (1 month to 2 years old) | Among children aged 1 month to 2 years, vitamin D status not associated with ALRI requiring hospitalization |
| Hebbar 2014 | Prevalence of vitamin D insufficiency and relationship between vitamin D levels infection and innate immunity | High prevalence of vitamin D deficiency<br>Serum vitamin D levels - no correlation with illness severity score |
| Ebenezer 2016 | Determine vitamin D status and association with outcomes | Vitamin D deficiency common among pediatric patients<br>No association between vitamin D status and admission and mortality such as higher mortality and/or longer PICU stay<br>Deficiency associated with mechanical ventilation severity of illness, vasopressor need |
| Narang 2016 | Identify an effective nutritional agent that reduces the need for antibiotics, duration of pneumonia and length of hospitalization would be highly cost-beneficial. | Severe vitamin D deficiency (<20 ng/ml), nonexclusive breastfeeding in the first six months of life, inadequate sun exposure found to be significant risk factors for ALRTI in Indian children. |
| Alvarez 2016 | Study aimed to investigate relationship between vitamin D status and plasma markers of lutathione (GSH) and cysteine (Cys) redox and immunity in critically ill children | Vitamin D sufficiency was associated with more reduced plasma hCySS, indicative of lower oxidative stress, in critically ill children. Plasma GSH, GSSG, and glutamine, however, were lower in the vitamin D sufficient group. Vitamin D role in maintaining redox status during pediatric critical illness requires further study. |
| Cetinkaya 2015 | Evaluate the effect of vitamin D levels on early-onset sepsis (EOS) in term infants | Lower maternal and neonatal 25-OHD levels associated with EOS. Suggest that adequate vitamin D supplementation during pregnancy may be helpful to prevent EOS in term neonates. |
| Ahmed 2014 | Examine relationship between vitamin D status and hospitalization for ALRTI in Nigerian children. | ALRTI not associated with vitamin D status, but associated with less exposure to sunlight. Exposure to sunlight & vitamin D supplementation contributed to vitamin D status in this population. |
| Jat 2016 | Evaluate Vitamin D levels and its correlation with severity and outcome of pneumonia in children. | Majority (86.4%) of children were vitamin D deficient. Vitamin D levels were found different in cases and controls and were not related to severity and outcome of pneumonia. |
| Sankar 2017 | Evaluate association of severe vitamin D deficiency with clinically important outcomes in children with septic shock. | Prevalence of severe vitamin D deficiency is high in children with septic shock admitted to pediatric intensive care unit. Severe vitamin D deficiency at admission seems to be associated with lower rates of shock reversal at 24 hours of ICU stay. |
| Cizmeci 2015 | Investigate if neonates with early-onset neonatal sepsis (EONS) had lower levels of vitamin D | Cord-blood 25(OH) D levels of neonates with EONS significantly lower than that of the healthy controls, and a low level of cord-blood vitamin D was found to be associated with an increased risk of EONS. Further studies are warranted to confirm this association. |
| Khakshour 2015 | Clarify the association between vitamin D deficiency and acute respiratory infection in children below age 5 years. | The group of children with respiratory disorders, 9 (42.9%) exhibited vitamin D deficiency. No meaningful statistical relation vitamin D deficiency with acute respiratory infections (p>0.05) |
| Korwutthikulrangsri 2015 | Determine vitamin D status in critically ill children and its relationship with adrenal function | Higher prevalence of vitamin D deficiency in critically ill children in comparison to controls. Patients vitamin D deficiency had higher median (IQR) PRISM III score and higher proportion of mortality than those with serum 25-OHD of equal or more than 12 ng/mL. |
| Elmoneim 2016 | Association of the level of vitamin D on admission & length of stay in the PICU, or duration of mechanical ventilation. | High prevalence of vitamin D deficiency among PICU patients & significant association with increased LOS and need for mechanical ventilation. Not significant association with mortality rate. |



For peer review only - http://bmjopen.bmj.com/site/about/guidelines.xhtml

| | | |
|---|---|---|
| Dinlen 2016 | Determine the association between serum 25-hydroxy vitamin D [25(OH)D] levels and acute respiratory tract infections (ALRTI) in newborns | Lower blood 25(OH) D levels might be associated with increased risk of ALRTI in term newborn babies. Appropriate vitamin D supplementation during pregnancy and early childhood may enhance newborns' respiratory health. |
| Moreno-Solis 2015 | Examine prevalence of hypovitaminosis D in infants with acute bronchiolitis compared with control subjects and to evaluate the relationship between serum 25-hydroxyvitamin D (25(OH) D) and the severity of bronchiolitis. | Prevalence of hypovitaminosis D is high in Spanish infants with bronchiolitis. The severity of acute bronchiolitis increases with a decline in serum 25 (OH) D level. |
| Inamo 2011 | Relationship between serum vitamin D concentrations and severity of ALRI in hospitalized children in Japan. | Significantly more children with ALRI that required supplementary oxygen and ventilator management were vitamin D deficient. Findings suggest: immunomodulatory properties of vitamin D may influence the severity of ALRI. |
| Karatekin 2009 | Association of serum 25-hydroxy vitamin D concentrations in newborns with acute lower respiratory infection (ALRI) and without clinical signs of rickets, and their mothers | Newborns with subclinical vitamin D deficiency may have an increased risk of suffering from ALRI. Strong positive correlation between newborns' and mothers' 25(OH) D concentrations indicates that adequate vitamin D supplementation of mothers should be emphasized during pregnancy especially in winter months. |
| Roth 2010 | Investigate association between vitamin D status and ALRI | Vitamin D status associated with ALRI in early childhood.<br>Randomized trials needed to establish if interventions to improve vitamin D status could reduce burden of ALRI in early childhood. |
| Mathias 2017 | Determination of association of 25(OH)D with cathelicidin and DBP (D binding protein) in children with sepsis | No association between vitamin D and cathelicidin or DBP (D binding protein) levels |
| Dhandai 2018 | Assess vitamin D deficiency as possible risk factor for late-onset sepsis in term and late preterm neonates | Vitamin D deficient neonates are at greater risk for late onset sepsis compared to those with sufficient vitamin D levels |
| Vo 2018 | Investigate the association between circulating 25-hydroxyvitamin D status upon admission and disease severity in infants hospitalized for bronchiolitis. Also, to determine if the association differs by the form of 25(OH)D (total, bioavailable or free 25 (OH) D. | Infants with total 25 (OH) D < 20 ng/ml had higher risk of intensive care and longer hospital length-of-stay |
| Li 2018 | Assess vitamin D nutritional status of children of 3 days to 14 years and investigated the relationship between community-acquired pneumonia and serum 25(OH)D level | Children with low serum 25 (OH) D levels may be at higher risk of receiving mechanical ventilation and presenting with multiple organ dysfunction. Vitamin D supplementation could be beneficial for the treatment and prevention of CAP |



For peer review only - http://bmjopen.bmj.com/site/about/guidelines.xhtml

**Additional Table 9 Assay used in each study to measure Vitamin D levels**

| Assay | Paper | Total Number of studies |
|---|---|---|
| Radioimmunoassay | Madden 2012; Inamo 2011; Karatekin 2009; Roth 2009; Roth 2010; Sakka 2014; Moreno-Solis 2015; Khashour 2015 | 8 |
| Competitive binding enzyme linked immunoassay | Basha 2014 | 1 |
| Binding protein assay | Onwuneme 2015 (2) | 1 |
| Liquid chromatography-mass spectrometry | McNally 2012; Korwutthikulrangsri 2015; Binks 2014; Cetinkaya 2015; Cizmeci 2015; Asilioglu 2017; Onwuneme (1) | 7 |
| Chemiluminescence immunoassay or chemiluminescent tracer | Alonso 2015; Rey 2014; Sankar 2016; Shah 2016; Prasad 2015; Ahmed 2015; Lopez 2016; Alvarez 2016; Say 2017; Sankar 2017; Jat 2017; Mathias 2017; Li 2018 | 13 |
| ELISA | Ponnarmeni 2016; Hebbar 2014; Elmoneim 2016; Narang 2016; Jia 2017; El-Gamasy 2017; Halwany 2017; Banajeh 2009; Badawi 2017 | 9 |
| ELFA (enzyme linked fluorescent assay) | Bustos 2014 | 1 |
| Electrochemiluminescence Immunoassay (ECLIA) | Dayal 2014, Ebenezer 2016, Garcia Soler 2017; Cayir 2014; | 4 |
| Clinical Laboratory Improvement Amendments–approved Vitamin D assay (Elecsys; Roche Diagnostics, Indianapolis, Indiana) | Hurwitz 2017 | 1 |
| Commercial immunoassay lit (I RIA Diasorin UK) | Wayse 2004, Ayulo 2014, | 2 |
| Immunoassay analyzer | Rippel 2012 | 1 |
| APPLIED 3200 Biosystem | Dinlen 2016 | 1 |
| Not reported | Yaghmaie 2017; Dhandai 2018 | 2 |
| Abbott Architect assay (Abbott, Waukegan, Illinois) | Vo 2018 | 1 |

For peer review only

**Additional Table 10 Funding and ethical approval of included studies**

| Study | Funding | Approval of study and ethics |
|---|---|---|
| Madden 2012 | Reported | Children's Hospital Boston institutional review board. Informed consent obtained from family. |
| Lopez 2016 | Reported | Approved by the Ethical Committee of Clinical Investigation of Galicia (CEIC ref 010/015) and all of the regional ethics committees of the participant centres informed consent forms were obtained from either a parent or legal guardian for each subject before study inclusion. |
| Garcia-Soler 2017 | Not reported | Study protocol approved by regional research ethics committee. Obtained informed consent from family of patients. Data recorded anonymously (encrypted electronic database).<br>Project adhered to the principles of Declaration of Helsinki and standards for good clinical practice. |
| McNally 2012 | Reported | Research ethics board approval for vitamin D sub study obtained from 6 centers, representing 337 of the original 389 study participants. |
| Rippel 2012 | Not Reported | Approved by Ethics Committee of The Royal Children's Hospital Melbourne |
| Alonso 2015 | Reported | Regional Ethics Committee of the Principality of Asturias |
| Asilioglu 2017 | Not reported | Approval of study by the Local Ethics Committee of Ondokuz Mayıs University (Samsun, Turkey). |
| Ayulo 2014 | Not reported | Institutional Review Board: Montefiore Medical Centre |
| Rey 2014 | Reported | Hospital Ethics Committee |
| Shah 2016 | Reported | Ethical approval obtained from Institutional ethics committee. Parents of children satisfying criteria gave written informed consent for participation of their child in the study. |
| Ponnarmeni 2016 | Not reported | The institution's ethics committee. Informed consent obtained |
| Onwuneme 2015 (1) | Reported | Approval by ethics committees of: Children's University Hospital, Temple Street and Our<br>Lady's Children's Hospital, Dublin, Ireland. Participants were informed and provided written consent before recruitment. |
| Jia 2017 | Not reported | Approved by Human Ethical Committee of Affiliated Hospital of Yan'an University, Yan'an. Informed consent was taken from mothers and/or parent of infants. |
| Halwany 2017 | Not reported | Approval by the Medical Ethics Committee of the Faculty of Medicine, Alexandria University |
| Sankar 2016 | Not reported | Institutional Ethics committee. Informed consent obtained |
| Say 2017 | Not reported | Approval by the local Ethics Committee and informed parental consent was obtained for all infants. |
| Sakka 2014 | Not reported | Not reported |
| Onwuneme2015 (2) | Reported | Ethics Committee of National Maternity Hospital. Informed written consent obtained from parents before recruitment |
| Dayal 2014 | Not reported | Ethics Committee of the Institute. Informed consent obtained. |
| Bustos 2016 | Reported | Comité Ético Científico del Servicio de Salud de Concepción |
| Hurwitz 2017 | Reported | Informed consent obtained and study protocol approved by the Institutional Review Boards at the University of Tennessee, St Jude Research Hospital, and the Centers for Disease Control and Prevention. |
| Cayir 2014 | Not reported | Approval from the institutional ethics committee and consent from the parents of all children in the study. |
| Badawi 2017 | Not reported | Children's Cairo University institutional review board approved study. Informed consent obtained from parents |
| Yaghmaie 2017 | Not reported | Study carried out in accordance with the Declaration of Helsinki, and the ethics committee of the Tehran University of Medical Sciences approved the protocols of the study. Records of patients' were kept confidential. Patients' consent provided |



For peer review only - http://bmjopen.bmj.com/site/about/guidelines.xhtml

| | | |
|---|---|---|
| | | for blood sampling and vitamin D serum level was assessed from patients' files, so no invasive method or extra blood sampling was done. |
| Basha 2014 | Not reported | Oral consent from the mothers. |
| Prasad 2015 | Not reported | Institutional review board approved the protocol. Written informed consent obtained from parents or guardians. |
| Wayse 2004 | Reported | Information not provided |
| El-Gamasy 2017 | Not reported | Study approved from the Ethical Committee of the Faculty of Medicine, Tanta University. Informed verbal or written parental consents from all subjects involved in the study. |
| Banajeh 2009 | Reported | Protocol approved and described in previous paper |
| Binks 2014 | Reported | Testing performed after approval by the Human Research Ethics Committee of the Northern Territory Department of Health and Menzies School of Health Research |
| Roth 2009 | Reported | Caregiver of each participant provided written informed consent and completed a questionnaire Study approved by the Human Research Ethics Board of the University of Alberta Health Sciences Faculties |
| Hebbar 2014 | Reported | Institutional Review Boards of Emory University and Children's Healthcare Atlanta Informed consent obtained |
| Ebenezer 2016 | Reported | Institutional Review Board (IRB), Informed consent from parents |
| Narang 2016 | Not reported | Not reported |
| Alvarez 2016 | Reported | Study approved by both the Emory University and Children's Healthcare of Atlanta Institutional Review Boards, and informed consent was obtained from patients' guardians prior to any study procedures |
| Cetinkaya 2015 | Not reported | Study protocol approved by the local Ethics Committee. Informed parental consent was obtained for all infants |
| Ahmed 2014 | Not reported | Informed written or oral consent obtained from the parents, and the study was approved by the National Hospital Abuja Ethics Committee |
| Jat 2016 | Not reported | Ethics committee approval was taken before commencing the study. Parent's informed consent was taken before enrolling children into study. |
| Sankar 2017 | No funding | Study approved by the institutional ethics committee. |
| Cizmeci 2015 | Reported | Acquisition of cord-blood was approved by the local ethics committee of Fatih University Medical School. |
| Khakshour 2015 | Not reported | Obtained informed consent from parents, data collection done using demographic questionnaire and serum level of 25-dehydroxycalcciferol was measured. Samples taken by a trained nurse at admission based on physician's orders. |
| Korwutthikulrangsri 2015 | Not reported | Ramathibodi Hospital Ethics Committee. Written informed consent from legal guardians of all participants |
| Elmoneim 2016 | Reported | MCH hospital Ethics Committee approved study protocol |
| Dinlen 2016 | Not reported | Study approved by the Local Ethics Committee. All parents fully informed about this investigation as well as its aim. Written consent was obtained from all parents. |
| Moreno-Solis 2015 | Reported | Written informed consent obtained from parents or legal guardian of all enrolled children. Protocol of study approved by the Ethics Committee before the beginning of this study. |
| Inamo 2011 | Not reported | Ethics Committee of Nihon University Nerima-Hikarigaoka Hospital. Informed consent obtained from the parents of all patients before inclusion in the study. |
| Karatekin 2009 | Not reported | Study approved by the Institution's Ethics Committee, and informed consent was obtained from the study participants. |
| Roth 2010 | Reported | Approved by The Johns Hopkins Bloomberg School of Public Health Institutional Review Board and the ethics committee of the Bangladesh Institute for Child Health at the Dhaka Shishu Hospital, Bangladesh. Signed permission prior to enrolment from parents/guardians. |



For peer review only - http://bmjopen.bmj.com/site/about/guidelines.xhtml

| | | |
|---|---|---|
| Mathias 2017 | Reported | The institution's Human Investigation Committee approved the study after a full board review and informed consent obtained |
| Dhandai 2018 | Not reported | The institute's ethics committee approved the study and informed written consent had been given by their parents or guardians |
| Vo 2018 | Reported | The institutional review boards at all participating sites approved the protocol and informed consent obtained from the infants' parents/legal guardians |
| Li 2018 | Not reported | Protocols for the study and written consent approved by the ethics committee of the Capital Institute of Pediatrics at Beijing, China |

For peer review only

**Additional Table 11 Age groups of children in each study**

| First author, date | Age group |
|---|---|
| Wayse 2004 | <5 years |
| Karatekin 2009 | Neonates |
| Roth 2009 | <2 years |
| Roth 2010 | 1–18 months |
| Inamo 2011 | <4 years |
| Madden 2012 | <21 years |
| Rippel 2012 | 16.5 (3.1 to 75.2) months |
| McNally 2012 | Newborn to 17 years |
| Ayulo 2014 | 1 to 21 years |
| Dayal 2014 | 3 months to 12 years |
| Hebbar 2014 | 0 to 18 years |
| Rey 2014 | <16 years |
| Cetinkaya 2015 | >37 weeks |
| Onwuneme (1) 2015 | <12 years |
| Onwuneme (2) 2015 | <32 weeks gestation |
| Prasad 2015 | 2 months to 12 years |
| Alonso 2015 | 1 month to 13 years |
| Korwutthikulrangsri 2015 | 79 (61) cases; 92 (40) controls months |
| Cizmeci 2015 | Neonates |
| Shah 2016 | 1 month to 15 years |
| Ponnarmeni 2016 | 1 to 12 years |
| Sankar 2016 | 1 month to 17 years |
| Bustos 2016 | >37 weeks and <15 years |
| Ebenezer 2016 | <18 years |
| Elmoneim 2016 | <14 years |
| Narang 2016 | 2 months to 5 years |
| Dinlen 2016 | Neonates |
| Lopez 2016 | 0 to >48 months |
| Alvarez 2016 | 0 to 18 years |
| Garcia-Soler 2017 | 6 months to 17 years |
| Sankar 2017 | <17 years |
| Ahmed 2015 | 2 to 60 months |
| Cayir 2014 | 1 to 13 years |
| Say 2017 | <37 weeks |
| Asilioglu 2017 | <=18 years |
| Basha 2014 | <5 years |
| Jia 2017 | <1 year |
| Jat 2017 | 1 month to 12 years |
| Yaghmaie 2017 | Age range not stated |
| El-Gamasy 2017 | 3 months to 12 years |
| Binks 2014 | <3 years |
| Halwany 2017 | >1 month to <= 5 years |
| Badawi 2017 | 1 month to 12 years |
| Moreno-Solis 2015 | 1 to 11 months |
| Sakka 2014 | <2 years |
| Hurwitz 2017 | <5 years |
| Banajeh 2009 | 2 to 59 months |
| Khakshour 2015 | < 5 years |
| Mathias 2017 | <= 18 years |
| Dhandai 2018 | Neonates |
| Vo 2018 | < 12 months |
| Li 2018 | 3 days to 14 years |



For peer review only - http://bmjopen.bmj.com/site/about/guidelines.xhtml

**Additional Table 12 Prevalence of vitamin D deficiency in each study of acute and critically ill children (sorted from highest to lowest)**

| Study | Prevalence of vitamin D deficiency (%) | Number of vitamin D deficient children | Total number of children |
|---|---|---|---|
| Wayse 2004 | 95.00 | 76 | 80 |
| Sakka 2014 | 92.70 | 89 | 96 |
| Karatekin 2009 | 92.00 | 23 | 25 |
| Onwuneme2015 (2) | 91.48 | 86 | 94 |
| Say 2017 | 87.00 | 87 | 100 |
| Dinlen 2016 | 86.70 | 26 | 30 |
| Cetinkaya 2015 | 84.00 | 42 | 50 |
| Jat 2016 | 84.00 | 42 | 50 |
| Roth 2010 | 84.00 | 21 | 25 |
| Prasad 2015 | 83.75 | 67 | 80 |
| Jia 2017 | 83.64 | 92 | 110 |
| Shah 2016 | 83.11 | 128 | 154 |
| Korwutthikulrangsri 2015 | 78.12 | 25 | 32 |
| Sankar 2016 | 74.26 | 75 | 101 |
| Sankar 2017 | 72.09 | 31 | 43 |
| Cizmeci 2015 | 70.00 | 28 | 40 |
| Mathias 2017 | 70.00 | 28 | 41 |
| McNally 2012 | 69.02 | 225 | 326 |
| Basha 2014 | 65.43 | 53 | 81 |
| Yaghmaie 2017 | 64.63 | 53 | 82 |
| Dhandai 2018 | 63.30 | 38 | 60 |
| Hebbar 2014 | 60.66 | 37 | 61 |
| Onwuneme 2015 (1) | 59.16 | 71 | 120 |
| Alvarez 2016 | 58.00 | 29 | 50 |
| Cayir 2014 | 56.82 | 50 | 88 |
| Elmoneim 2016 | 56.67 | 17 | 30 |
| Narang 2016 | 56.00 | 28 | 50 |
| Li 2018 | 55.83 | 445 | 797 |
| Ponnarmeni 2016 | 50.81 | 63 | 124 |
| Asilioglu 2017 | 48.00 | 120 | 250 |
| Badawi 2017 | 44.30 | 39 | 88 |
| Garcia-Soler 2017 | 43.82 | 149 | 340 |



For peer review only - http://bmjopen.bmj.com/site/about/guidelines.xhtml

| | | | |
|---|---|---|---|
| Bustos 2016 | 43.30 | 39 | 90 |
| Ebenezer 2016 | 40.38 | 21 | 52 |
| Madden 2012 | 40.12 | 205 | 511 |
| Halwany 2017 | 37.25 | 38 | 102 |
| Banajeh 2009 | 36.71 | 29 | 79 |
| Rippel 2012 | 34.49 | 109 | 316 |
| El-Gamasy 2017 | 32.50 | 26 | 80 |
| Ahmed 2014 | 30.00 | 15 | 50 |
| Rey 2014 | 29.49 | 46 | 156 |
| Vo 2018 | 29.33 | 298 | 1016 |
| Inamo 2011 | 28.50 | 8 | 28 |
| Dayal 2014 | 25.00 | 23 | 92 |
| Khakshour 2015 | 24.32 | 9 | 37 |
| Lopez 2016 | 24.20 | 84 | 347 |
| Alonso 2015 | 15.63 | 45 | 288 |
| Ayulo 2014 | 15.63 | 61 | 216 |
| Binks 2014 | 14.86 | 11 | 74 |
| Moreno-Solis 2015 | 13.33 | 9 | 48 |
| Hurwitz 2017 | 12.22 | 11 | 90 |
| Roth 2009 | 4.69 | 3 | 64 |

For peer review only

**Additional Table 13 Characteristics of studies used in the meta-analysis of prevalence**

| Study | Total number of patients | Total number of vitamin D deficient patients | Country, setting | Age range | Design | Quality score (NOS) |
|---|---|---|---|---|---|---|
| Vo 2018 | 1016 | 298 | USA, PICU | < 12 months | cohort | **8** |
| Li 2018 | 797 | 445 | China, PICU | 3 days to 14 years | case-control | 8 |
| Madden 2012 | 511 | 205 | Boston US, PICU | < 21 years | cohort | 8 |
| Lopez 2016 | 347 | 84 | Spain, hospitalised | 0 to >48 months | cohort | 8 |
| Garcia-Soler 2017 | 340 | 149 | Spain, PICU | 6 months to 17 years | cohort | 7 |
| McNally 2012 | 326 | 225 | Canada, PICU | newborn to 17 years of age | cohort | 7 |
| Rippel 2012 | 316 | 109 | Australia, PICU | 16.5 (3.1–75.2) months | cohort | 8 |
| Alonso 2015 | 288 | 45 | Spain, PICU | 1 month to 13 years | cross sectional | 5 |
| Asilioglu 2017 | 250 | 120 | Turkey, PICUs | <= 18 years | cohort | 6 |
| Ayulo 2014 | 216 | 61 | Spain, PICU | 1 month to 13 years | cross sectional | 5 |
| Rey 2014 | 156 | 46 | Spain, PICU | <16 years | case-control | 8 |
| Shah 2016 | 154 | 128 | India,PICU | 1 month-15 years | cohort | 6 |
| Ponnarmeni 2016 | 124 | 63 | India, PICU | aged 1–12 years | case-control | 6 |
| Onwuneme 2015 (1) | 120 | 71 | Ireland, PICU | <12 years old | case-control | 8 |
| Jia 2017 | 110 | 92 | China, PICU | infants <1 years old | case-control | 7 |
| Halwany 2017 | 102 | 38 | Alexandria, PICU | >1 month to ≤ 5 years | cohort | 6 |
| Sankar 2016 | 101 | 75 | India, PICU | 1 month to 17 years | cohort | 8 |
| Say 2017 | 100 | 87 | Turkey, NICU | gestational age <37 weeks | case-control | 6 |
| Sakka 2014 | 96 | 89 | Egypt, hospitalized | infants (<2 years old) | case-control | 8 |
| Onwuneme 2015 (2) | 94 | 86 | Ireland, NICU | preterm infants at <32 weeks gestation | cohort | 8 |
| Dayal 2014 | 92 | 23 | India, PICU | 3 months to 12 years | cohort | 6 |
| Bustos 2016 | 90 | 39 | Chile, PICU | >37 weeks and <15 years | cohort | 7 |
| Hurwitz 2017 | 90 | 11 | USA, hospitalised | <5 years old | cohort | 6 |
| Cayir 2014 | 88 | 50 | Turkey, PICU | 1 to 13 years | case-control | 7 |
| Badawi 2017 | 88 | 39 | Cairo, Egypt PICU | 1 month to 12 years | cohort | 7 |
| Yaghmaie 2017 | 82 | 53 | Iran, PICU | children undefined | cross sectional | 4 |
| Basha 2014 | 81 | 53 | Cairo Egypt, PICU | <5 years old | case-control | 7 |
| Prasad 2015 | 80 | 67 | India, PICU | 2 months-12 years | cohort | 7 |
| Wayse 2004 | 80 | 76 | Indapur India, PICU | <5 years | case-control | 6 |
| El-Gamasy 2017 | 80 | 26 | Egypt, PICU | 3 months to 12 years | Case-control | 7 |
| Banajeh 2009 | 79 | 29 | Iran, hospitalised | aged 2–59 months | cohort | 7 |
| Binks 2014 | 74 | 11 | Australia, PICU | <3 years old | cross sectional | 6 |
| Roth 2009 | 64 | 3 | Canada, PICU | aged 1-25 months | case-control | 7 |
| Hebbar 2014 | 61 | 37 | Atlanta, PICU | 0 to 18 years | case-control | 6 |
| Dhandai 2018 | 60 | 38 | India, NICU | neonates | case-control | 6 |



For peer review only - http://bmjopen.bmj.com/site/about/guidelines.xhtml

| | | | | | | |
|---|---|---|---|---|---|---|
| Ebenezer 2016 | 52 | 21 | India, PICU | <18 years | cohort | 6 |
| Narang 2016 | 50 | 28 | Punjab, India, PICU | 2 months to 5 years | case-control | 6 |
| Alvarez 2016 | 50 | 29 | Atlanta, PICU | 0 to 18 years | cross sectional | 6 |
| Cetinkaya 2015 | 50 | 42 | Instabul/Turkey, NICU | neonates | case-control | 6 |
| Ahmed 2014 | 50 | 15 | Nigeria, PICU | 2–60 months | case-control | 8 |
| Jat 2016 | 50 | 42 | India, PICU | 1 month to 12 years | case-control | 8 |
| Sankar 2017 | 43 | 31 | India, PICU | <=17 years | cohort | 8 |
| Mathias 2017 | 41 | 28 | USA, PICU | <= 18 years | cohort | 6 |
| Cizmeci 2015 | 40 | 28 | Instabul/Turkey, NICU | neonates | case-control | 7 |
| Khakshour 2015 | 37 | 9 | Iran, hospitalized | <5 years | Case-control | 5 |
| Korwutthikulrangsri 2015 | 32 | 25 | Bangkok, PICU | months not more specific range | nested case-control | 5 |
| Elmoneim 2016 | 30 | 17 | Saudi Arabia, PICU | <14 years | case-control | 5 |
| Dinlen 2016 | 30 | 26 | Ankara Turkey, NICU | neonates | case-control | 6 |
| Moreno-Solis 2015 | 48 | 9 | Spain, PICU | infants 1–11 months | case-control | 6 |
| Inamo 2011 | 28 | 8 | Tokyo Japan, PICU | 1-48 months | cohort | 6 |
| Karatekin 2009 | 25 | 23 | Istanbul Turkey, NICU | neonates | case-control | 6 |
| Roth 2010 | 25 | 21 | Bangladesh | 1-18 months | case-control | 8 |

Studies arranged from largest to smallest total sample size. NOS = Newcastle Ottawa Score

For peer review only

**Additional Table 14 Sensitivity analyses for prevalence of vitamin D deficiency in acute and critically ill children**

| Patient category | Number of studies (Total number of individuals; number of vitamin D deficient individuals) | Pooled proportion % (95% CI) Random effects model | 95% PI | Pooled proportion % (95% CI) Fixed effects model | Heterogeneity ($I^2$) % (95% CI) | Q value, d.f. p-value for heterogeneity | Median % (IQR) of vitamin D deficiency |
|---|---|---|---|---|---|---|---|
| Acute and critically ill children, excluding studies that used other thresholds | 40 (6,543; 3,000) | 53.0 (46.4-59.5) | 17.7-85.5 | 45.1 (43.8-46.5) | 95.5 (94.5-96.2) | 859.8, 39, < 0.0001 | 55.9 (33.4-55.9) |
| Sample size >= 82 (large) | 26 (6,094; 2,731) | 51.5 (43.6-59.4) | 16.5-85.2 | 44.0 (42.6-45.3) | 96.8 (96.0-97.4) | 773.1, 25, < 0.0001 | 46.2 (30.7-67.9) |
| Sample size < 82 (small) | 26 (1,340; 742) | 58.2 (47.5-68.2) | 13.2-92.8 | 54.8 (51.7-58.0) | 90.9 (87.9-93.2) | 275.4, 25, < 0.0001 | 62.0 (33.6-82.3) |
| Cohort studies | 22 (4,456; 1,874) | 48.3 (40.2-56.5) | 15.7-82.4 | 41.0 (39.5-42.6) | 95.8 (94.6-96.7) | 499.6, 21, < 0.0001 | 41.9 (30.6-68.8) |
| Case-control | 26 (2,484; 1,461) | 63.4 (54.9-71.2) | 22.2-91.4 | 57.2 (55.1-59.4) | 92.2 (89.8-94.1) | 322.2, 25, < 0.0001 | 62.0 (52.1-84.0) |
| Cross sectional | 4 (494; 138) | 34.8 (12.8-66.0) | 0.10-99.6 | 30.3 (25.9-35.2) | 96.7 (94.0-98.2) | 90.4, 3, < 0.0001 | 36.8 (15.4-59.7) |
| Studies from India | 11 (886; 592) | 68.9 (54.9-80.1) | 18.3-95.6 | 64.0 (60.4-67.5) | 96.7 (94.0 98.2) | 140.2, 10, < 0.0001 | 72.1 (53.4-83.4) |
| Studies from Turkey | 7 (583; 376) | 76.3 (60.9-87.0) | 20.9-97.5 | 61.0 (56.6-65.2) | 91.1 (84.2-95.0) | 67.5, 6, < 0.0001 | 84 (63.4-86.8) |
| Studies that only recruited neonates | 7 (399; 330) | 83.0 (73.1-90.0) | 42.4-97.0 | 80.7 (76.1-84.5) | 76.6 (51.0-88.9) | 25.7, 6, 0.0003 | 86.7 (77.0-89.2) |
| Studies with children of all other ages except neonates | 45 (7,035; 3,143) | 49.7 (43.5-55.8) | 16.1-83.5 | 44.2 (43.0-45.5) | 95.2 (94.3-96.0) | 919.9, 44, < 0.0001 | 48.0 (29.5-68.3) |

CI = confidence intervals; $I^2$ = heterogeneity; df = degrees of freedom. Vitamin D deficiency defined in our study as <50 nmol/L (20 ng/mL)
$I^2$ statistic used to estimate heterogeneity between pooled studies: $I^2$ >= 75% was considered as high heterogeneity, PI= Prediction interval; IQR = interquartile range: Lower (Q1) to Upper (Q3) quartile



For peer review only - http://bmjopen.bmj.com/site/about/guidelines.xhtml

**Additional Table 15 Multivariate meta-regression model for prevalence**

| Predictors | k | b-coefficient | se | t-value | p-value | ci.lb | ci.ub | F-value | I^2 (%) | R^2 (%) | QE |
|---|---|---|---|---|---|---|---|---|---|---|---|
| Full model: year + clinical setting + quality score + design + country group + total sample size | 52 | | | | | | | 4.14 | 96.0 | 29.6 | 759.8<br>p < 0.0022 |
| year | | -0.024 | 0.059 | -0.419 | 0.677 | -0.143 | 0.094 | | | | |
| total study sample size | | -0.0005 | 0.001 | -0.545 | 0.588 | -0.002 | 0.001 | | | | |
| country group (group 1 or 2 versus group 3) | | 0.996 | 0.359 | 2.771 | 0.008 | 0.272 | 1.719 | | | | |
| clinical setting (NICU versus PICU) | | -1.645 | 0.471 | -3.496 | 0.001 | -2.593 | -0.698 | | | | |
| design group (cohort vs other i.e. case-control or cross sectional) | | 0.288 | 0.321 | 0.899 | 0.374 | -0.358 | 0.935 | | | | |
| quality score | | 0.214 | 0.152 | 1.410 | 0.165 | -0.092 | 0.521 | | | | |

k = number of outcomes included in the model fitting; se = standard errors of the coefficients; ci.lb = lower bound of the confidence intervals for the coefficients; ci.ub = upper bound of the confidence intervals for the coefficients; QE = test statistic for the test of (residual) heterogeneity; I^2 = residual heterogeneity / unaccounted variability; R^2 (amount of heterogeneity accounted for; PICU = pediatric intensive care units, NICU = neonatal intensive care units



For peer review only - http://bmjopen.bmj.com/site/about/guidelines.xhtml

**Additional Table 16 Characteristics of studies included in the meta-analysis for prevalence in individuals with sepsis**

| Study | Total number of patients with sepsis | Total number of vitamin D deficient patients with sepsis | Country, setting | Age | Design | Quality score (NOS) |
|---|---|---|---|---|---|---|
| Shah 2016 | 160 | 135 | India, PICU | 1 month to 16 years | cohort | 6 |
| Asilioglu 2017 | 120 | 20 | Turkey, PICU | 1 month to ≤18 years | cohort | 7 |
| Say 2017 | 100 | 87 | Turkey, NICU | preterm infants at <37 weeks gestation | case-control | 6 |
| Dhandai 2018 | 60 | 38 | India, NICU | neonates | case-control | 6 |
| Ponnarmeni 2016 | 62 | 32 | India, PICU | 1 to 12 years | case-control | 6 |
| McNally 2012 | 48 | 33 | Canada, PICU | newborn to 17 years | cohort | 7 |
| Onwuneme 2015 (2) | 46 | 32 | Ireland, NICU | preterm infants at <32 weeks gestation | cohort | 8 |
| El-Gamasy 2017 | 46 | 20 | Egypt, pediatric emergency department | 3 months to 12 years | case-control | 7 |
| Sankar 2017 | 43 | 31 | India, PICU | <=17 years | cohort | 8 |
| Mathias 2017 | 41 | 28 | USA, PICU | <=18 years | cohort | 6 |
| Cizmeci 2015 | 40 | 28 | Turkey, NICU | neonates | case-control | 7 |
| Onwuneme 2015 (1) | 35 | 32 | Ireland, PICU | <12 years old | case-control | 8 |
| Hebbar 2014 | 30 | 17 | Atlanta, PICU | 0 to 18 years | case-control | 6 |
| Ebenezer 2016 | 16 | 8 | India, PICU | <18 years | cohort | 6 |
| Korwutthikulrangsri 2015 | 12 | 4 | Bangkok, PICU | moths (<8/9) | nested-case control | 5 |
| Prasad 2015 | 11 | 9 | India, PICU | 2 months to 12 years | cohort | 7 |
| Bustos 2016 | 10 | 7 | Chile, PICU | >37 weeks and < than 15 years | cohort | 7 |
| Dayal 2014 | 9 | 4 | India, tertiary care hospital | 3 months to 12 years | cohort | 6 |



For peer review only - http://bmjopen.bmj.com/site/about/guidelines.xhtml

**Additional Table 17 Sensitivity analyses for prevalence of vitamin D deficiency in acute and critically ill children with sepsis**

| Patient category | Number of studies (Total number of individuals; number of vitamin D deficient individuals) | Pooled proportion (%, 95% CI) Random effects | 95% PI | Pooled proportion (%, 95% CI) Fixed effects | Heterogeneity ($I^2$) %, 95% CI | Q value, d.f. p-value for heterogeneity | Median % (IQR) of vitamin D deficiency |
|---|---|---|---|---|---|---|---|
| Excluding studies that used other thresholds | 14 (654; 395) | 62.0 (47.3-74.72) | 13.3-94.5 | 60.4 (56.0-64.7) | 89.7 (84.5-93.2) | 126.5, 13, < 0.0001 | 65.8 (50.4-70.0) |
| Sample size >= 42 (large) | 9 (685; 428) | 63.2 (44.6-78.5) | 9.4-96.6 | 62.5 (58.2-66.6) | 94.3 (91.1-96.3) | 139.2, 8 < 0.0001 | 68.8 (51.6-72.1) |
| Sample size < 42 (small) | 9 (204; 137) | 64.7 (52.5-75.3) | 29.5-89.0 | 64.7 (57.4-71.4) | 57.9 (11.8-79.9) | 19.0, 8, 0.0148 | 68.3 (50.0-70.0) |
| Cohort studies | 10 (504; 307) | 63.2 (43.7-79.1) | 8.57-96.9 | 62.5 (57.5-67.4) | 92.0 (87.3-94.9) | 112.2, 9 < 0.0001 | 69.2 (54.6-71.6) |
| Case-control | 8 (385; 285) | 64.9 (50.1-77.3) | 18.6-93.8 | 63.6 (58.2-68.7) | 84.9 (72.0-91.8) | 46.3, 7 < 0.0001 | 60 (49.6-74.3) |
| Studies from India | 7 (361; 257) | 66.0 (51.4-78.1) | 21.3-93.3 | 69.2 (63.9-74.1) | 81.1 (61.8-90.6) | 31.7, 6 < 0.0001 | 63.3 (50.8-77.0) |
| Studies from Turkey | 3 (260; 135) | 59.2 (13.6-93.1) | 0.00-100 | 51.2 (43.2-59.3) | 97.8 (95.8- 98.8) | 90.0, 2, < 0.0001 | 70.0 (43.3-78.5) |
| Studies that only included neonates | 4 (246; 185) | 73.7 (60.3-83.8) | 15.9-97.7 | 73.5 (67.3-78.9) | 76.0 (34.1-91.3) | 12.5, 3, 0.0058 | 69.8 (68.0-74.3) |
| Studies that included children of all other ages except neonates | 14 (643; 380) | 60.7 (45.5-74.0) | 11.8-94.7 | 58.7 (54.2-63.0) | 90.1 (85.2-93.4) | 131.8, 13 < 0.0001 | 62.5 (45.8-71.6) |

CI = confidence intervals; $I^2$ = heterogeneity; df = degrees of freedom. Vitamin D deficiency defined in our study as <50 nmol/L (20 ng/mL). $I^2$ statistic used to estimate heterogeneity between pooled studies: $I^2$ >= 75% was considered as high heterogeneity; PI= Prediction interval; IQR = interquartile range: Lower (Q1) to Upper (Q3) quartile

For peer review only

**Additional Table 18 Sensitivity analyses for mortality. Pooled odds ratios for risk of mortality in deficient versus not deficient children**

| Patient category | Number of studies (Total number of individuals) | Pooled OR (95% CI) Vitamin D deficient/ Vitamin D non-deficient p-value | | Heterogeneity ($I^2$) % (95% CI) | Q value, d.f. p-value for heterogeneity | Eggers p-value |
|---|---|---|---|---|---|---|
| | | Random effects | Fixed effects | | | |
| Excluding studies that used other thresholds | 14 (2,030) | 1.59 (1.05-2.41) p-value = 0.028 | 1.52 (1.08-2.13) p-value = 0.016 | 24.3 (0.0-59.9) | 17.18, 13, 0.1910 | p-value = 0.120 |
| Cohort studies only | 14 (2,197) | 1.80 (1.15-2.81) p-value = 0.009 | 1.65 (1.17-2.34) p-value = 0.004 | 31.3 (0.0-63.7) | 18.92, 13, 0.1255 | p-value = 0.042 |
| Case-control studies only | 7 (266) | 1.97 (0.88-4.42) p-value = 0.098 | 1.97 (1.02-3.82) p-value = 0.044 | 19.7 (0.0-87.7) | 3.73, 3, 0.2916 | Number of studies too small to test for small study effects (k.min=10) |
| Studies from India | 7 (646) | 1.08 (0.70-1.69) p-value = 0.710 | 1.08 (0.70-1.69) p-value = 0.710 | 0.0 (0.0-62.4) | 4.56, 6, 0.589 | Number of studies too small to test for small study effects (k.min=10) |

CI = Confidence Intervals; $I^2$ = I squared statistic used to estimate heterogeneity (inconsistency); df = degrees of freedom, results reported in 1 decimal place; OR= odds ratio. Vitamin D deficiency defined as < 50 nmol/L or 20 ng/ml. We used the $I^2$ statistic to estimate heterogeneity between pooled studies: $I^2$ >= 75% was considered as high heterogeneity.

For peer review only



For peer review only - http://bmjopen.bmj.com/site/about/guidelines.xhtml

## Additional Figures

**Additional Figure 1 Funnel plot of studies of prevalence of vitamin D deficiency in acute and critically ill children.** Horizontal axis shows logit transformed proportion and the standard error of the logit transformed proportion is plotted on the vertical axis. Each dot represents an individual study and the vertical line represents the pooled proportion from a random-effects meta-analysis. The diagonal lines represent pseudo 95% confidence limits (p = 0.01, Egger's test).

Plot A shows the funnel plot before trim and fill method was applied and B after. Solid circles in plot B represent original data and open circles represent imputed filled studies (11 studies added) on the left-hand side of the funnel plot.

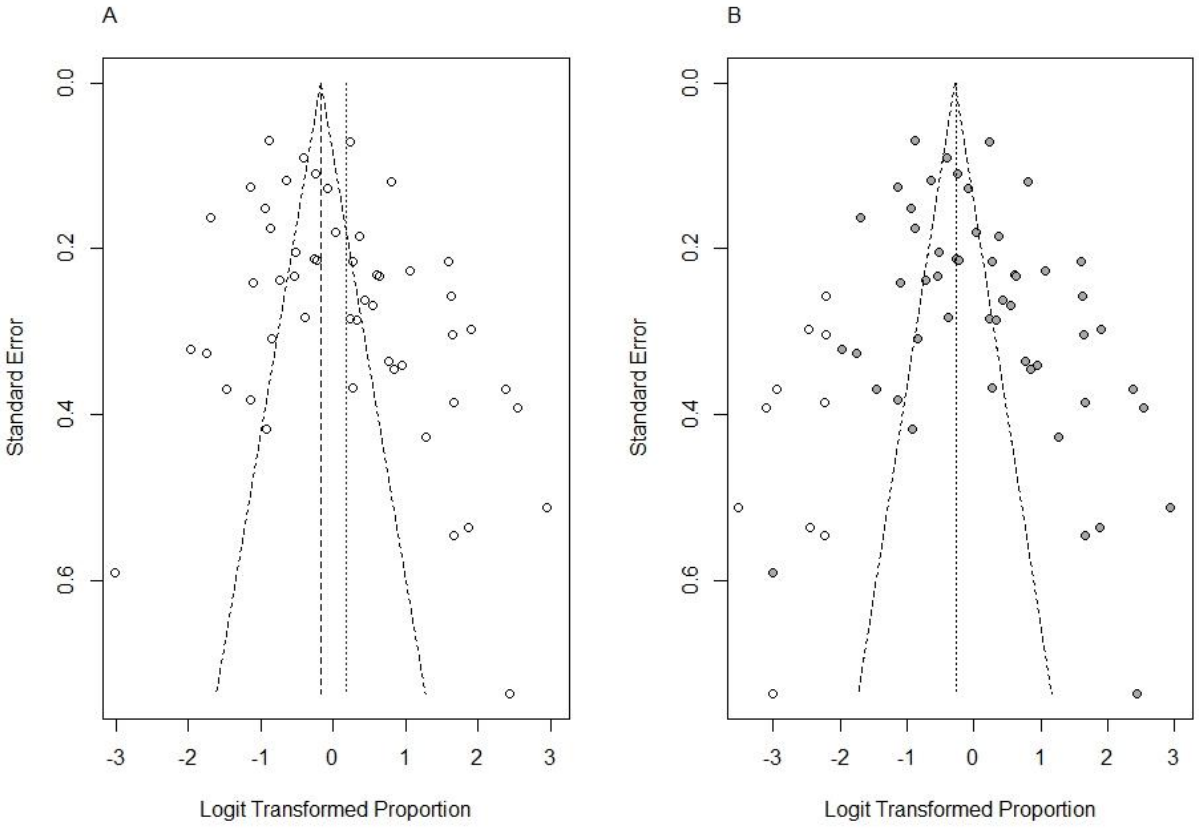

**Additional Figure 2. Pooled prevalence estimate for vitamin D deficiency in acute and critically ill children (by country group).** Forest plot shows results from the random effects model. Diamonds represent pooled proportion of vitamin D deficiency for the countries in each subgroup (group 1, group2, group 3). The diamond at the bottom shows the overall pooled estimate of all the 52 studies. Each square shows the prevalence estimate of each study and the horizontal line across each square represents the 95% confidence interval (CI) of the prevalence estimate.

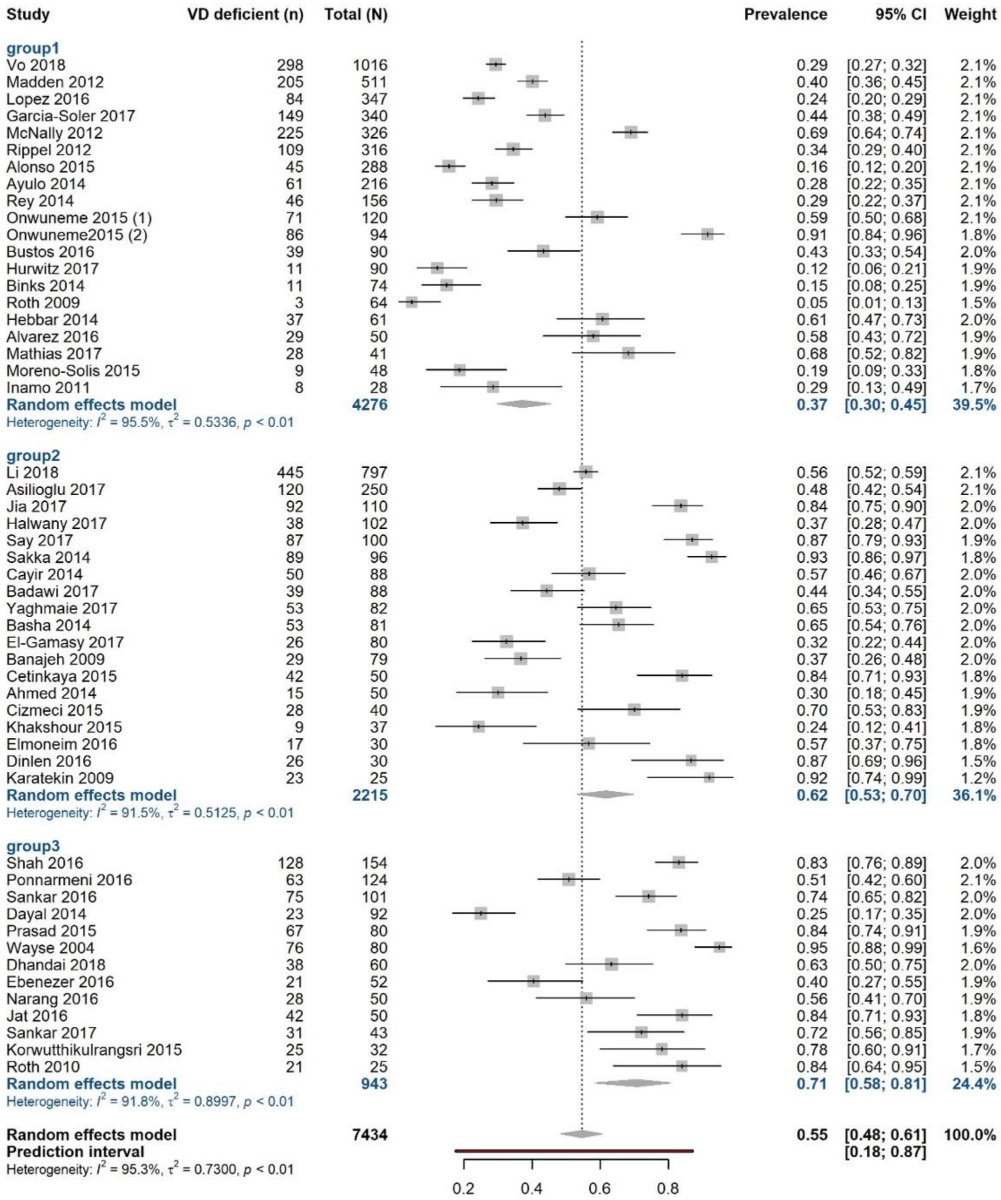

**Additional Figure 3. Pooled prevalence estimates for vitamin D deficiency in acute and critically ill children (neonates versus all other age groups).** Forest plot shows results from the random effects model. Diamonds represent pooled proportion of vitamin D deficiency for the studies in neonates and all other age groups. The diamond at the bottom shows the overall pooled estimate of all the 52 studies. Each square shows the prevalence estimate of each study and the horizontal line across each square represents the 95% confidence interval (CI) of the prevalence estimate.

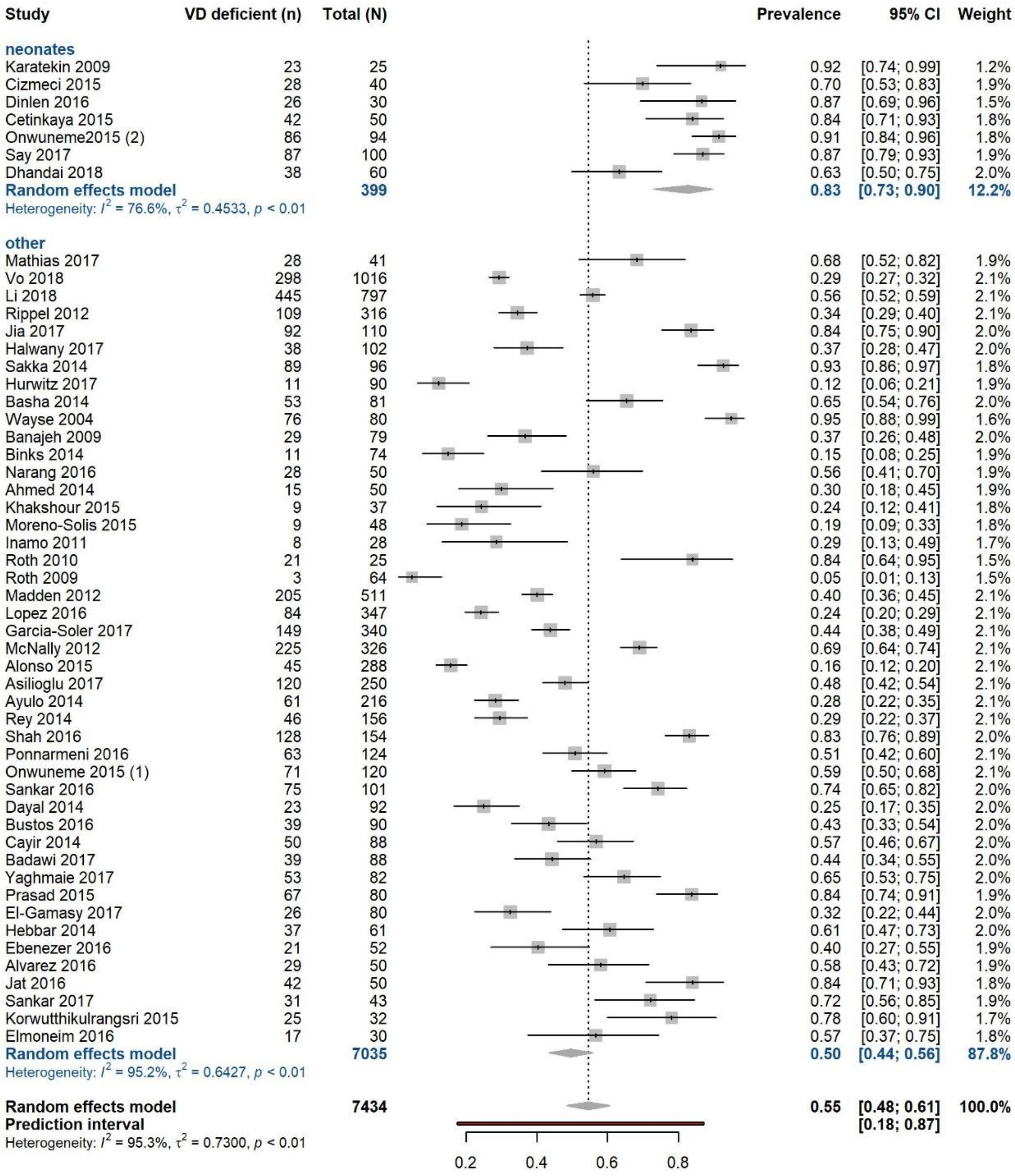

**Additional Figure 4 Bubble plots of univariate meta-regressions.** Each study is represented by a circle. Predictor variables: A study design, B year of publication, C quality score and D total children are plotted on the x-axis with the effect measure logit transformed proportion shown on the vertical (y-axis).

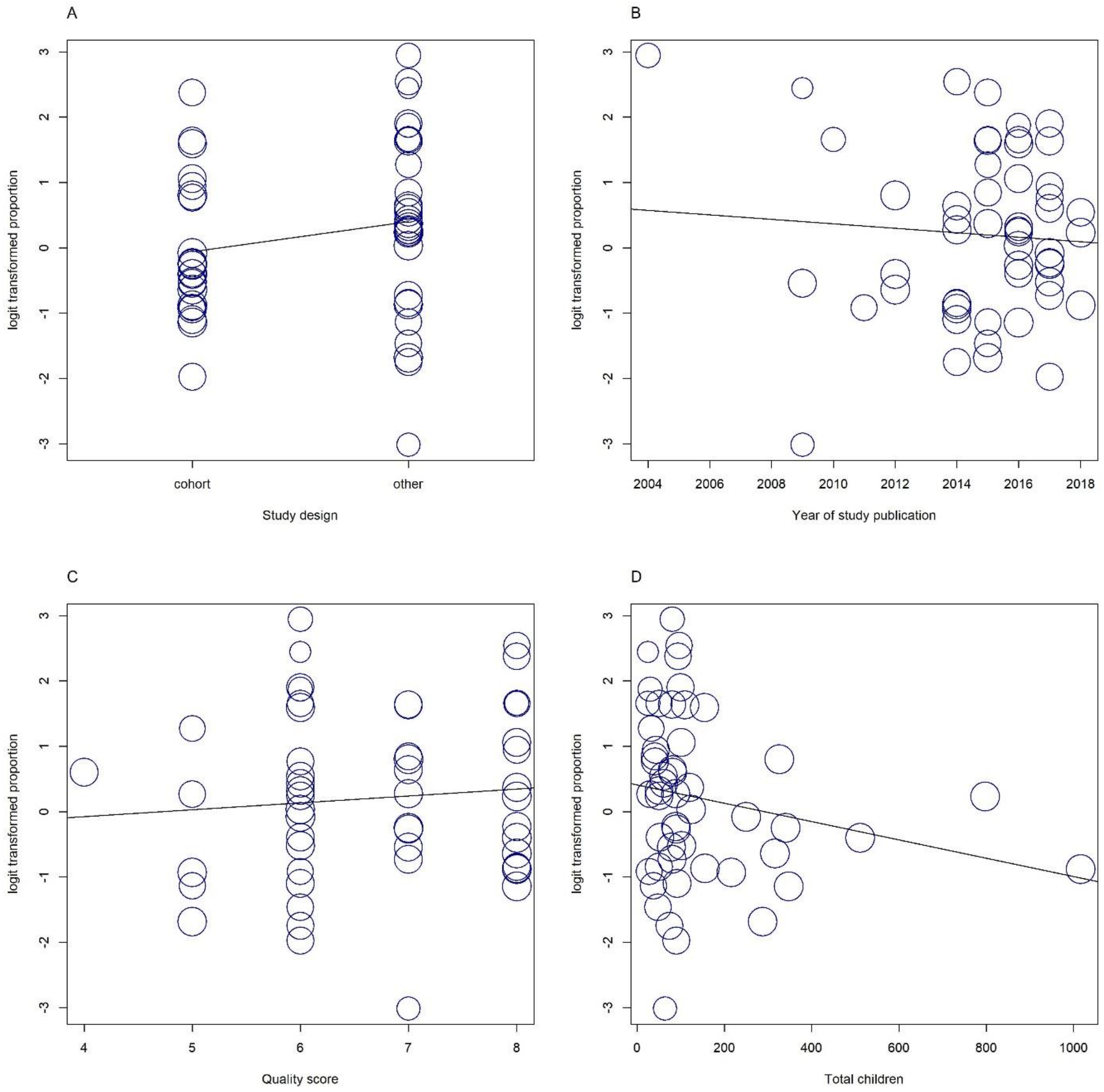

**Additional Figure 5 Funnel plot for prevalence of vitamin D deficiency in acute and critically ill children with sepsis.** Horizontal axis shows logit transformed proportion and the standard error of the logit transformed proportion is plotted on the vertical axis. Each dot represents an individual study and the vertical line represents the pooled proportion from a random-effects meta-analysis. The diagonal lines represent pseudo 95% confidence limits (p = 0.81, Egger's test).

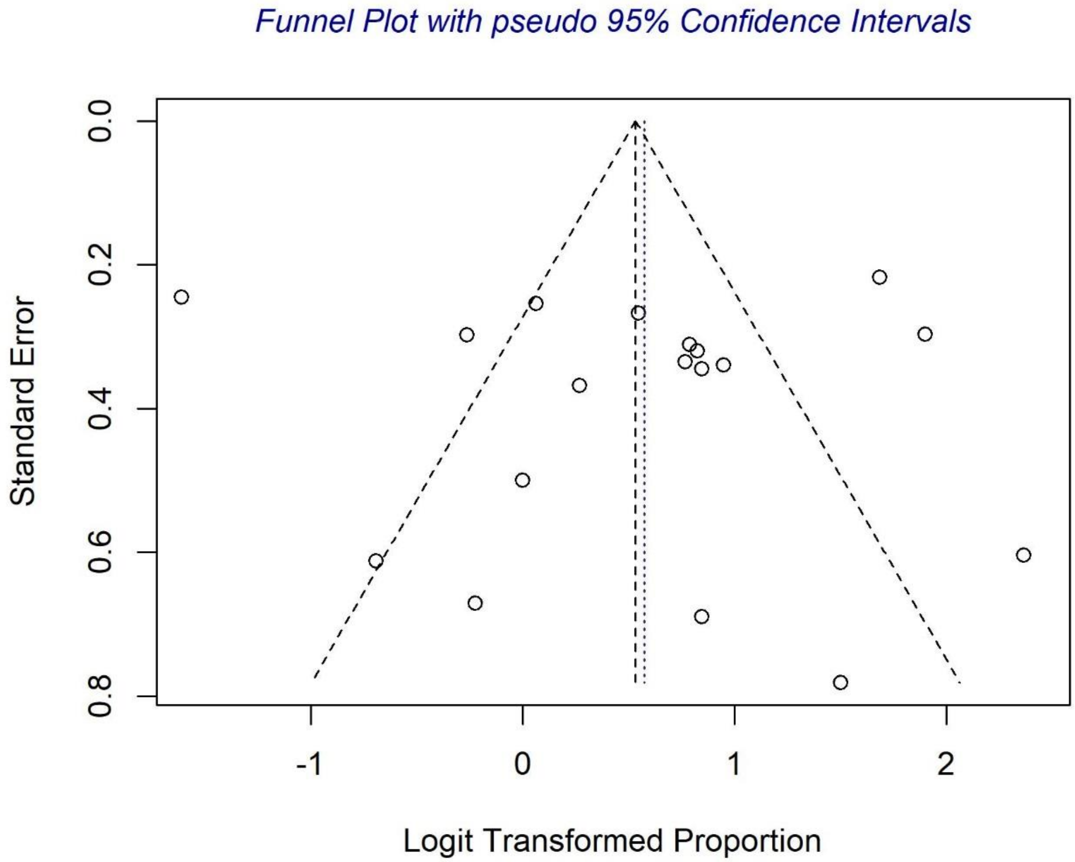




For peer review only - http://bmjopen.bmj.com/site/about/guidelines.xhtml

**Additional Figure 6 Pooled prevalence estimate for vitamin D deficiency in acute and critically ill children with sepsis (subgroup analysis by study design).** Forest plot shows results from the random effects model. Diamonds represent the pooled proportion of vitamin D deficiency for the studies in each subgroup (case-control and cohort). The diamond at the bottom shows the overall pooled estimate of all the 18 studies. Each square shows the prevalence estimate of each study and the horizontal line across each square represents the 95% confidence interval (CI) of the prevalence estimate.

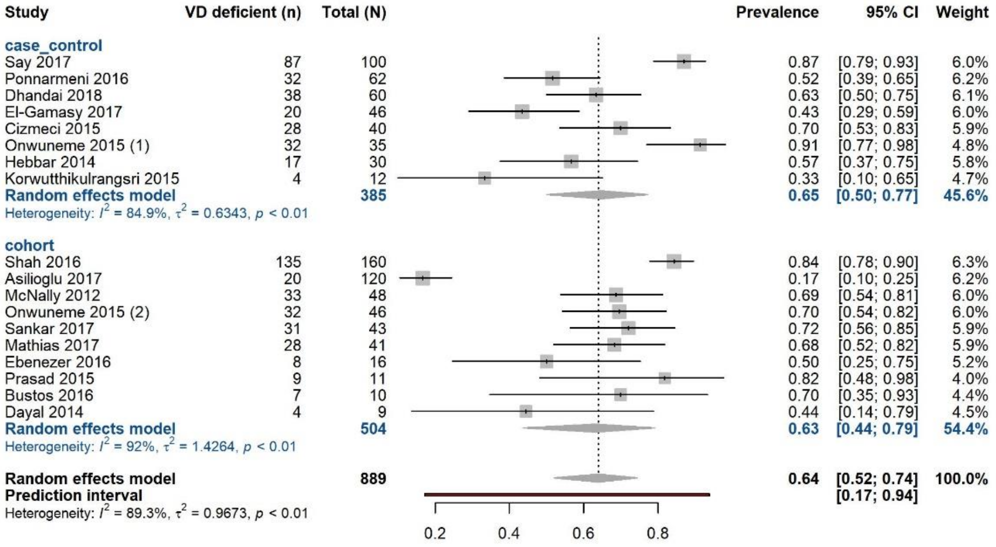


only



For peer review only - http://bmjopen.bmj.com/site/about/guidelines.xhtml

**Additional Figure 7 Funnel plot of risk of mortality in vitamin D deficient versus vitamin D non-deficient acute and critically ill children.** Horizontal axis shows logit transformed odds ratio and the standard error of the log odds ratio is plotted on the vertical axis. Each dot represents an individual study and the vertical line represents the overall pooled odds ratio from random-effects meta-analysis. The diagonal lines represent pseudo 95% confidence limits (p = 0.084, Egger's test).

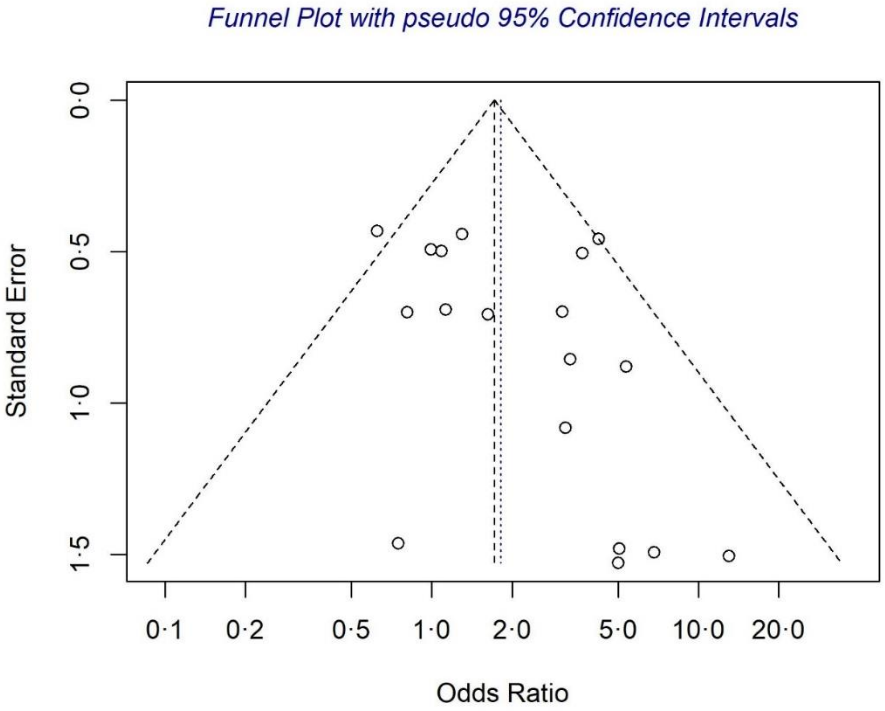


w only



For peer review only - http://bmjopen.bmj.com/site/about/guidelines.xhtml

**Additional Figure 8 Pooled odds ratio (OR) and 95% CI for risk of mortality in vitamin D deficient versus vitamin D non-deficient acute and critically ill children (fixed effects model).** Diamond represents the overall odds ratio (with corresponding 95% Confidence Interval). Each square shows the odds ratio of each study and the horizontal line across each square represents the 95% confidence interval (CI) of the estimate.

| Study | Deficient Dead (n) | Deficient Total (N) | Not deficient Dead (n) | Not deficient Total (N) | OR | 95% CI | Weight |
|---|---|---|---|---|---|---|---|
| Ebenezer 2016 | 5 | 21 | 5 | 31 | 1.62 | [0.41; 6.51] | 4.9% |
| Ponnarmeni 2016 | 10 | 63 | 9 | 61 | 1.09 | [0.41; 2.90] | 9.8% |
| Sankar 2016 | 23 | 75 | 8 | 26 | 1.00 | [0.38; 2.62] | 10.0% |
| Rippel 2012 | 3 | 109 | 7 | 207 | 0.81 | [0.20; 3.19] | 5.0% |
| Dayal 2014 | 3 | 23 | 3 | 69 | 3.30 | [0.62; 17.65] | 3.3% |
| Shah 2016 | 54 | 128 | 14 | 26 | 0.63 | [0.27; 1.46] | 13.0% |
| Prasad 2015 | 14 | 67 | 1 | 13 | 3.17 | [0.38; 26.50] | 2.1% |
| Bustos 2016 | 4 | 39 | 0 | 51 | 13.06 | [0.68; 250.17] | 1.1% |
| McNally 2012 | 5 | 225 | 0 | 101 | 5.06 | [0.28; 92.45] | 1.1% |
| Elmoneim 2016 | 1 | 17 | 1 | 13 | 0.75 | [0.04; 13.24] | 1.1% |
| Korwutthikulrangsri 2015 | 6 | 25 | 0 | 7 | 5.00 | [0.25; 100.15] | 1.0% |
| Ayulo 2014 | 4 | 61 | 2 | 155 | 5.37 | [0.96; 30.11] | 3.1% |
| Garcia-Soler 2017 | 7 | 149 | 3 | 191 | 3.09 | [0.79; 12.16] | 5.0% |
| Onwuneme 2015 (2) | 5 | 60 | 0 | 34 | 6.84 | [0.37; 127.56] | 1.1% |
| Asilioglu 2017 | 16 | 120 | 9 | 85 | 1.30 | [0.55; 3.10] | 12.4% |
| El-Gamasy 2017 | 14 | 26 | 13 | 54 | 3.68 | [1.36; 9.92] | 9.5% |
| Badawi 2017 | 27 | 39 | 17 | 49 | 4.24 | [1.72; 10.41] | 11.5% |
| Sankar 2017 | 19 | 31 | 7 | 12 | 1.13 | [0.29; 4.39] | 5.1% |
| **Fixed effect model** | | **1278** | | **1185** | **1.72** | **[1.27; 2.33]** | **100.0%** |

Heterogeneity: $I^2 = 25.7\%$, $\tau^2 = 0.1564$, $p = 0.15$

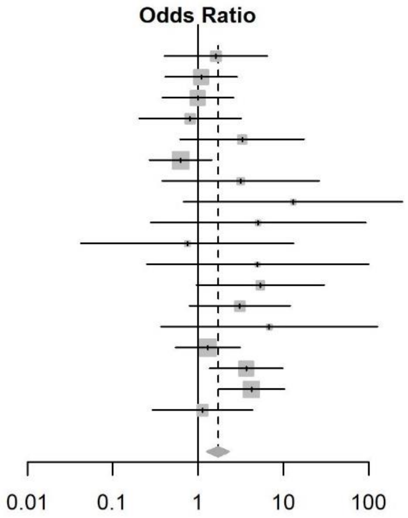




For peer review only - http://bmjopen.bmj.com/site/about/guidelines.xhtml

# MOOSE (Meta-analyses Of Observational Studies in Epidemiology) Checklist

A reporting checklist for Authors, Editors, and Reviewers of Meta-analyses of Observational Studies. You must report the page number in your manuscript where you consider each of the items listed in this checklist. If you have not included this information, either revise your manuscript accordingly before submitting or note N/A.

| Reporting Criteria | Reported (Yes/No) | Reported on Page No. |
|---|---|---|
| **Reporting of Background** | | |
| Problem definition | Yes | 4-5 |
| Hypothesis statement | Yes | 5 |
| Description of Study Outcome(s) | Yes | 7 |
| Type of exposure or intervention used | Yes | 1-2 |
| Type of study design used | Yes | 2 |
| Study population | Yes | 2 |
| **Reporting of Search Strategy** | | |
| Qualifications of searchers (eg, librarians and investigators) | Yes | 6 |
| Search strategy, including time period included in the synthesis and keywords | Yes | 6 |
| Effort to include all available studies, including contact with authors | Yes | 6-7 |
| Databases and registries searched | Yes | 6 |
| Search software used, name and version, including special features used (eg, explosion) | Yes | 6, 9 |
| Use of hand searching (eg, reference lists of obtained articles) | Yes | 7 |
| List of citations located and those excluded, including justification | Yes | 10 & Suppl. |
| Method for addressing articles published in languages other than English | Yes | 6 |
| Method of handling abstracts and unpublished studies | Yes | 7 |
| Description of any contact with authors | Yes | 7 |
| **Reporting of Methods** | | |
| Description of relevance or appropriateness of studies assembled for assessing the hypothesis to be tested | Yes | 6-7 |
| Rationale for the selection and coding of data (eg, sound clinical principles or convenience) | Yes | 6-7 |
| Documentation of how data were classified and coded (eg, multiple raters, blinding, and interrater reliability) | Yes | 7 |
| Assessment of confounding (eg, comparability of cases and controls in studies where appropriate | Yes | 7 |

For peer review only

| Reporting Criteria | Reported (Yes/No) | Reported on Page No. |
|---|---|---|
| Assessment of study quality, including blinding of quality assessors; stratification or regression on possible predictors of study results | Yes | 7 & 20 |
| Assessment of heterogeneity | Yes | 8-9 |
| Description of statistical methods (eg, complete description of fixed or random effects models, justification of whether the chosen models account for predictors of study results, dose-response models, or cumulative meta-analysis) in sufficient detail to be replicated | Yes | 8-9 |
| Provision of appropriate tables and graphics | Yes | 12 & Suppl. |
| **Reporting of Results** | | |
| Table giving descriptive information for each study included | Yes | 10 & Suppl. |
| Results of sensitivity testing (eg, subgroup analysis) | Yes | 13, 15-16 |
| Indication of statistical uncertainty of findings | Yes | 20-21 |
| **Reporting of Discussion** | | |
| Quantitative assessment of bias (eg, publication bias) | Yes | 8-9, 15-18 |
| Justification for exclusion (eg, exclusion of non–English-language citations) | Yes | 7 |
| Assessment of quality of included studies | Yes | 7 |
| **Reporting of Conclusions** | | |
| Consideration of alternative explanations for observed results | Yes | 21-22 |
| Generalization of the conclusions (ie, appropriate for the data presented and within the domain of the literature review) | Yes | 21-22 |
| Guidelines for future research | Yes | 21-22 |
| Disclosure of funding source | Yes | 9 & 22 |

**Once you have completed this checklist, please save a copy and upload it as part of your submission. DO NOT include this checklist as part of the main manuscript document. It must be uploaded as a separate file.**

For peer review only

BMJ Open

# Importance of vitamin D in acute and critically ill children with subgroup analyses of sepsis and respiratory tract infections: a systematic review and meta-analysis

| | |
|---|---|
| Journal: | *BMJ Open* |
| Manuscript ID | bmjopen-2018-027666.R2 |
| Article Type: | Research |
| Date Submitted by the Author: | 13-Mar-2019 |
| Complete List of Authors: | Cariolou, Margarita; Imperial College London School of Public Health, Epidemiology and Biostatistics<br>Cupp, Meghan; Imperial College London School of Public Health, Epidemiology and Biostatistics<br>Evangelou, Evangelos; Imperial College London School of Public Health, Epidemiology and Biostatistics; University of Ioannina School of Medicine, Department of Hygiene and Epidemiology<br>Tzoulaki, Ioanna; Imperial College London School of Public Health, Epidemiology and Biostatistics; University of Ioannina School of Medicine, Department of Hygiene and Epidemiology<br>Berlanga-Taylor, Antonio; Imperial College London School of Public Health, Epidemiology and Biostatistics |
| <b>Primary Subject Heading</b>: | Epidemiology |
| Secondary Subject Heading: | Paediatrics, Research methods |
| Keywords: | Vitamin D, EPIDEMIOLOGY, meta-analysis, sepsis, PAEDIATRICS, INTENSIVE & CRITICAL CARE |
| | |

SCHOLARONE™
Manuscripts

# Importance of vitamin D in acute and critically ill children with subgroup analyses of sepsis and respiratory tract infections: a systematic review and meta-analysis

Margarita Cariolou[1] MPH, Meghan A. Cupp[1] MPH, Evangelos Evangelou[1,2] PhD, Ioanna Tzoulaki[1,2] PhD and Antonio J. Berlanga-Taylor[1]* DPhil

[1]MRC-PHE Centre for Environment and Health, Department of Epidemiology & Biostatistics, School of Public Health, Faculty of Medicine, Imperial College London, St Mary's Campus, Norfolk Place, UK, LONDON W2 1PG

[2]Department of Hygiene and Epidemiology, University of Ioannina Medical School, Ioannina, Greece

*Correspondence should be addressed to Dr. Antonio J. Berlanga-Taylor (a.berlanga@imperial.ac.uk), MRC-PHE Centre for Environment and Health, Department of Epidemiology & Biostatistics, School of Public Health, Faculty of Medicine, Imperial College London, St Mary's Campus, Norfolk Place, LONDON W2 1PG.

For peer review only

## ABSTRACT

**Objectives:** To estimate the prevalence of 25-hydroxyvitamin D (25(OH)D) deficiency and investigate its association with mortality in children with acute or critical conditions.

**Design:** Systematic review and meta-analysis of observational studies.

**Data sources:** PubMed, OVID, Google Scholar and the Cochrane Library searched until 21 December 2018.

**Eligibility criteria:** Studies of children hospitalised with acute or critical conditions who had blood 25(OH)D levels measured.

**Data extraction and synthesis:** We obtained pooled prevalence estimates of 25(OH)D deficiency and odds ratios for mortality. We calculated 95% confidence and prediction intervals and investigated heterogeneity and evidence of small-study effects.

**Results:** Fifty-two studies were included. Of 7,434 children, 3,473 (47.0%) were 25(OH)D deficient (<50 nmol/L). The pooled prevalence estimate of 25(OH)D deficiency was 54.6% (95% CI 48.5-60.6, $I^2$=95.3%, p<0.0001). Prevalence was similar after excluding smaller studies (51.5%). In children with sepsis (18 studies, 889 total individuals) prevalence was 64.0% (95% CI 52.0-74.4, $I^2$=89.3%, p<0.0001) and 48.7% (95% CI 38.2-59.3; $I^2$=94.3%, p<0.0001) in those with respiratory tract infections (RTI) (25 studies, 2,699 total individuals). Overall, meta-analysis of mortality (18 cohort studies, 2,463 total individuals) showed increased risk of death in 25(OH)D deficient children (OR 1.81, 95% CI 1.24-2.64, p=0.002, $I^2$=25.7%, p=0.153). Four (22.0%) of the 18 studies statistically adjusted for confounders. There were insufficient studies to meta-analyse sepsis and RTI related mortality.




For peer review only - http://bmjopen.bmj.com/site/about/guidelines.xhtml

**Conclusions:** Our results suggest that 25(OH)D deficiency in acute and critically ill children is high and associated with increased mortality. Small-study effects, reverse causation and other biases may have confounded results. Larger, carefully designed studies in homogeneous populations with confounder adjustment are needed to clarify the association between 25(OH)D levels with mortality and other outcomes.

**Registration** PROSPERO (CRD42016050638)

**Copyright** Open access article under terms of CC BY

**Keywords** paediatric, vitamin D, intensive care, sepsis, meta-analysis, prevalence, mortality, systematic review, respiratory tract infections

**Strengths and limitations of this study**

- We comprehensively assessed the magnitude and relevance of vitamin D (25(OH)D) circulating levels in paediatric acute and critically ill patients using a large number of studies with large total sample size with pre-specified sub-group and sensitivity analyses.
- We used the currently recommended cut-off of less than 50 nmol/L for vitamin D deficiency.
- We did not find enough studies to perform meta-analyses for mortality from sepsis or respiratory tract infection in relation to vitamin D status.
- We did not identify longitudinal studies with multiple time-point, pre-admission or pre-disease vitamin D measurements.
- Most studies were single centre with heterogeneous patient groups and few controlled for important confounders that influence vitamin D levels such as age, BMI, gender, season of measurements, vitamin D supplementation and comorbidities.

For peer review only



For peer review only - http://bmjopen.bmj.com/site/about/guidelines.xhtml

## INTRODUCTION

Vitamin D is an essential nutrient[1, 2] representing a group of fat soluble secosteroids with key endocrine functions.[3] It is synthesized in the skin upon sunlight exposure[4] while dietary sources, such as oily fish, egg yolk, certain fungi and supplements, are usually secondary sources. Vitamin D is critical in bone metabolism[5] and calcium homeostasis,[6] as well as acting as an important regulator in extra-skeletal metabolic processes,[7] cardiovascular and immune systems.[8] Many observational and laboratory studies have observed the anti-inflammatory properties of vitamin D,[9] including direct regulation of endogenous anti-microbial peptide production.[10]

It is therefore crucial for humans to have sufficient vitamin D levels to maintain bone health and possibly improve response to infection.[6, 11, 12] Infants and children are especially dependent on vitamin D to achieve healthy bone development and growth.[13, 14] Well-known functional outcomes of adequate vitamin D levels in children include rickets prevention, higher bone mineral content and reduced bone fracture rates.[5, 14] In otherwise healthy children in the United States, the reported prevalence of vitamin D deficiency (25OHD levels of < 25 nmol/L) ranges from 9 to 18%.[15] The Endocrine Society Clinical Practice Guidelines and the Institute of Medicine (IOM) suggest that 25(OH)D levels less than 50 nmol/L (20 ng/mL) reflect a deficient state.[4, 16]

Studies in adults reflect a high prevalence of vitamin D deficiency both in general intensive care unit (ICU) and sepsis patients and strongly suggest an association between low vitamin D and poor clinical outcomes, including increased mortality, particularly in those suffering from sepsis.[2, 17] Recent clinical trials of vitamin D supplementation in adults appear promising in both general critical care[18, 19] and sepsis.[20]



For peer review only - http://bmjopen.bmj.com/site/about/guidelines.xhtml

Sepsis remains a challenging clinical entity with high social and economic costs.[21] Each year there are approximately 123,000 sepsis cases and around 37,000 deaths in England alone.[22] Recent reports show an increased prevalence of paediatric sepsis,[23] likely a reflection of an increased population with chronic comorbidities, higher rates of opportunistic infections and multi-drug resistant organisms.[24] Respiratory tract infections account for a large proportion of underlying diagnoses in acute and critical care conditions[24, 25] but remain understudied.[26]

The magnitude, relevance and quality of evidence of vitamin D deficiency in children receiving acute care is not clear. Several recent studies have addressed these questions with mixed results. We sought to summarise the evidence regarding the implications of vitamin D deficiency and its prevalence in general acute care, ICU, respiratory tract infection and sepsis patients in the paediatric population. We carried out a systematic review and meta-analysis of circulating vitamin D levels, as measured by 25(OH)D, to assess the prevalence of vitamin D deficiency (≤ 50 nmol/L) and its association with mortality in these conditions.

## METHODS

We used the Preferred Reporting Items for Systematic Reviews and Meta-Analyses (PRISMA) guidelines to report our review.[27] (*Additional Table 1*). We also followed the Meta-Analysis of Observational Studies in Epidemiology (MOOSE) guidelines[28] as no relevant randomized controlled trials have been reported.

### Search strategy and selection criteria



For peer review only - http://bmjopen.bmj.com/site/about/guidelines.xhtml

Our population of interest consists of paediatric patients with acute conditions and/or those treated in ICU or emergency units for acute conditions whose vitamin D status was assessed prior to or during admission. We included published cross sectional, case-control and cohort studies that measured circulating 25(OH)D levels and either reported prevalence, odds ratios (OR) or data to enable calculation of these measures. Studies were excluded if they were reviews, case reports, surveys, commentaries, replies, not original contributions, experimental *in vitro* or if they recruited patients who were not treated in emergency, neonatal intensive care units (NICUs), paediatric intensive care units (PICUs) or for acute conditions. Studies were also excluded if they only enrolled vitamin D deficient patients, investigated healthy populations only or did not measure circulating 25(OH)D levels as an indicator of vitamin D status. When we identified more than one publication utilising the same cohort, we included the publication which shared our review's objective to investigate vitamin D levels and prevalence of deficiency.

For purposes of our review, we classified vitamin D deficiency as being 25(OH)D less than 50 nmol/L (equivalent to 20 ng/mL), as suggested by the IOM.[16] Different age categories were used to designate patients as "children" in the studies reviewed. We therefore included all "children" (neonates up to 21 years) as defined by each treating facility and this included "neonates", "infants", "toddlers", "children" and "adolescents".

We searched PubMed, OVID, Google Scholar and the Cochrane Library from inception up until 21$^{st}$ December 2018, with no language restrictions. Search terms used across these databases included: "critical care", "vitamin D", "pediatric", "child", "neonate", "toddler", "intensive care unit", "sepsis" and "septic shock". Search terms used in OVID and PubMed are listed in the *Additional Tables 2A and 2B*. Literature searches were performed by two investigators independently (MC and AJBT) and included initial screening of titles and abstracts, followed by



For peer review only - http://bmjopen.bmj.com/site/about/guidelines.xhtml

full text screening. Any disagreements for study eligibility were resolved by discussion between the two investigators. Reference lists of the selected papers, including reviews, were also checked for relevant titles. Abstracts of relevant titles were then assessed for eligibility. Corresponding authors were contacted to obtain additional information if necessary. A data extraction form was designed *a priori* in Excel. Variables extracted from each study included year of publication, country of study, clinical setting, cut-off given to define vitamin D deficiency, total number of children, total number of cases, study design and age range.

**Study quality assessment**

The quality of each included study was assessed using the Newcastle-Ottawa Scale (NOS) adapted *a priori* for this review, for cohort, case-control and cross-sectional study designs (*Additional Tables 3A, 3B* and *3C*).[29] We classified studies as low (1-3), medium (4-6) or high quality (7-9) for purposes of sensitivity analysis.

**Prevalence and mortality outcomes**

In the majority of studies (n = 40), prevalence of vitamin D deficiency was extracted as reported with a threshold of ≤ 50 nmol/L. If prevalence was not reported directly, it was calculated using data provided in each study (cases ≤ 50 nmol/L / total number of study participants) (*Additional Tables 4A* and *4B*). Extracted or calculated prevalence values were then combined in a meta-analysis. For mortality, we calculated unadjusted odd ratios (OR) as:

OR = (vitamin D deficient patients who died * vitamin D non-deficient patients who did not die)/ (vitamin D deficient patients who did not die * vitamin D non-deficient patients who died)



For peer review only - http://bmjopen.bmj.com/site/about/guidelines.xhtml

We had sufficient information to calculate ORs < 50 nmol/L for 40 studies (77.0%). For the 12 studies with insufficient information, we used the lower cut-off values reported as a conservative approximation (*Additional Table 5*). We converted 25(OH)D values using: nmol/L = ng/mL * 2.496.

**Data analysis**

We obtained proportions of vitamin D deficiency with 95% confidence intervals (CI) using the Clopper-Pearson method[30] in R. We used a random effects model[31] to account for the variation observed within and between studies due to the different ages and acute conditions in the populations considered. For each meta-analysis we also obtained the 95% prediction interval (PI) to further account for between study heterogeneity. This helps to evaluate how consistent an observed effect would be in a future study that will investigate the same association.[32] We obtained pooled proportions and pooled ORs with fixed effect model for sensitivity analysis or in cases where heterogeneity was low.[33-35] For prevalence we also calculated median and interquartile range (IQR) for comparisons with pooled prevalence estimates.

We investigated possible sources of heterogeneity using sensitivity and subgroup analyses. Cochran's Q was used to assess the heterogeneity and the $I^2$ statistic was used to estimate the percentage of total variation across studies which can be attributed to heterogeneity. Confidence intervals of $I^2$ were calculated to aid interpretation.[36] A Q value of < 0.05 was considered significant and an $I^2$ statistic greater or equal to 75% indicated a high level of variation due to heterogeneity.[37, 38] We used Egger's regression test to present results of small-study effects and funnel plot asymmetry[39] and generated funnel plots for visual assessment and screening. A p-value < 0.05 indicated evidence of small-study effects. With few studies, Egger's test has low power to



For peer review only - http://bmjopen.bmj.com/site/about/guidelines.xhtml

detect such bias, therefore we only estimated small-study effects for analyses with more than ten studies.[40] When small-study effects were detected based on this threshold, we used trim-and-fill methods to add potentially missed studies and re-calculate an adjusted pooled estimate.[41]

To further assess heterogeneity, we utilised meta-regression to identify predictor variables that could explain variation in study prevalence estimates. We used restricted maximum likelihood (REML) estimations in the model to account for residual heterogeneity[42] and the Knapp-Hartung method to adjust confidence intervals and test statistics. This method estimates between study variance using a t-distribution, rather than a z-distribution, yielding a more conservative inference.[43] We tested the following continuous predictors: year of study publication, total sample size and quality score. Categorical variables included study setting (PICU, NICU), study design (case-control, cross-sectional and cohort) and country group by geographic region and economic development (group 1, group 2, and group 3) and were dummy coded.

We used R version 3.5.0 and Microsoft Excel 2010 for analyses and data collection. The R packages "meta"[44] and "metafor"[45] were used for analyses. Only results of the random effects model are reported for prevalence due to the expected heterogeneity between populations being considered. Our protocol is registered in PROSPERO (CRD42016050638).

**Role of the funding source**

The study received funding from the UK Medical Research Council. The funders had no role in data collection, analysis, interpretation or writing of the report. All authors had access to the data in the study.

**Patient involvement**



For peer review only - http://bmjopen.bmj.com/site/about/guidelines.xhtml

No patients were involved in this study. We only used data from previously published studies.

## RESULTS

### Screening and study characteristics

After title and abstract screening, we identified 2,890 potentially relevant studies (*Figure 1*) and eighty-five full text articles were assessed for eligibility. Rationale for study exclusion included: studies including adults, study populations other than critically ill children or with acute conditions, studies of circulating vitamin D levels and deficiency in healthy children or in children with chronic conditions. Four studies[46-49] were excluded due to insufficient data reporting (*Additional Table 6*). We also excluded three studies[50-52] that used the same cohort of children and included a single study to represent the cohort.[53] Ultimately, 52 studies met criteria for inclusion (*Additional Table 7*).

*Figure 1 Flow chart of study selection process*

The primary objective of most included studies was to determine circulating vitamin D concentration ("status") in children and/or prevalence of vitamin D deficiency. Secondary objectives included investigation of associations between deficiency of circulating vitamin D and various outcomes, such as hospital mortality length of stay, requirement of ventilation and/or illness severity (*Additional Table 8*).

All included studies reported vitamin D measurement assay methods used (*Additional Table 9*) and stated that samples were collected and analysed within the first 24 hours of hospital admission. Studies reported ethical approval and consent for participation from parents or guardians (*Additional Table 10*). Included studies were published between 2004 and 2018, with the majority



For peer review only - http://bmjopen.bmj.com/site/about/guidelines.xhtml

(n = 40, 77.0%) published between 2014 and 2017 (*Additional Table 7*). In total, 7,434 children were hospitalized in paediatric or neonatal intensive care units or emergency units or for acute conditions. Sample sizes of critically ill children ranged from 25[54] to 1,016.[55] In 18 studies the total number of cases was greater than 100.

Studies originated from 15 countries, with the majority from India[8, 56-65] (n = 11) or Turkey[54, 66-71] (n = 7) (*Additional Table 7*). All were of medium or high quality (NOS score median 7, range 4-8). The score range for cohort studies was 6 to 8 (n = 30), for case-control studies 5 to 8 (n = 18) and for cross sectional 4 to 6 (n = 4). Studies used a broad range of ages to classify patients as "children". Seven studies (13.5%)[54, 65, 67, 69-72] included only neonates. In two[67, 72] of these studies, neonates were preterm. The largest age range was seen in the study of Ayulo et al 2014, which included individuals between 1 and 21 years of age (*Additional Table 11*). Forty-two of the included studies (80.8%) included patients admitted for medical conditions and the other ten[53, 61, 66, 73-78] included both surgical and medical patients. Of the 52 included studies 26 used a control group and had a total number of 2,479 controls of which 773 (31.2%) were vitamin D deficient.

All studies included both female and male participants. For mortality, four of the 18 studies (22.0%) carried out multivariate regression analysis with adjustment for confounders. The remaining studies presented results using a variety of methods, including Spearman's correlation analysis, chi-square or Fisher's exact tests or descriptive statistics.

**Prevalence of vitamin D deficiency**

We included 52 studies representing a total of 7,434 children hospitalised with critical or acute conditions. Of these, 3,473 (47.0%) were classified as vitamin D deficient (< 50 nmol/L).



For peer review only - http://bmjopen.bmj.com/site/about/guidelines.xhtml

Prevalence of deficiency ranged from 5.0%[79] to 95.0%[60] , median (IQR) 56.3% (31.9 to 75.2%) (*Additional Table 12*). Sample sizes ranged from 25 to 1,016, with a median of 82 individuals (*Additional Table 13*). Using a random effects model, the pooled prevalence estimate of vitamin D deficiency was 54.6% (95% CI 48.5-60.6) with a high proportion of variation attributed to heterogeneity ($I^2$ = 95.3%, 95% CI 94.5-96.0, $p < 0.0001$) (*Figure 2*) and evidence of funnel plot asymmetry (p = 0.01, Egger's test) (*Table 1* and *Additional Figure 1*). Trim and fill analysis estimated 11 unpublished studies in the lower left-hand side of the funnel plot (*Additional Figure 1*). The re-calculated adjusted pooled estimate was lower 43.6% (95% CI 37.5-50.0) with significant heterogeneity ($p < 0.0001$).

**Table 1 - Pooled estimates of vitamin D (25(OH)D) deficiency in acute and critically ill children and those with sepsis or respiratory tract infections**

| **Patient category** | **Number of studies (Total number of individuals; number of deficient individuals)** | **Pooled proportion (%, 95% CI) Random effects** | **95% PI** | **Pooled proportion (%, 95% CI) Fixed effects** | **Heterogeneity ($I^2$) % (95% CI)** | **Q value, d.f. p-value for heterogeneity** | **Eggers p-value** |
|---|---|---|---|---|---|---|---|
| All children (includes those with sepsis and respiratory tract infections) | 52 (7,434; 3,473) | 54.6 (48.5-60.6) | 17.5-87.2 | 45.7 (44.4-46.9) | 95.3 (94.5-96.0) | 1086.6, 51, < 0.0001 | 0.01 |
| Critically ill children with sepsis only | 18 (889; 565) | 64.0 (52.0-74.4) | 17.1-93.9 | 63.0 (59.3-66.6) | 89.3 (84.6-92.5) | 158.52 17 < 0.0001 | 0.81 |
| Critically ill children with respiratory tract infections only | 25 (2,699; 1,076) | 48.7 (38.2-59.3) | 9.96-89.1 | 37.0 (35.0-39.1) | 94.3 (92.7-95.6) | 423.07 24 < 0.0001 | 0.05 |

CI = confidence intervals; $I^2$ = heterogeneity; df = degrees of freedom. Vitamin D deficiency defined in our study as 25(OH)D < 50 nmol/L (20 ng/mL). $I^2$ statistic used to estimate heterogeneity between pooled studies: $I^2 \geq 75\%$ was considered high heterogeneity; PI = Prediction Interval

*Figure 2 Pooled prevalence estimate for vitamin D deficiency in acute and critically ill children by study design.*

## Sensitivity analysis for prevalence



For peer review only - http://bmjopen.bmj.com/site/about/guidelines.xhtml

We did not detect material differences in prevalence after exclusion of the 12 studies which did not directly report prevalence < 50 nmol/L (53.0%, 95% CI 46.4-59.5; $I^2 = 95.5\%$, 95% CI 94.5-96.2, $p < 0.0001$) (*Additional Table 14*).

When examining results by median sample size (defining "large" as ≥ 82 and "small" as < 82), we found that the 26[8, 53, 55-58, 66-68, 72-74, 77, 78, 80-91] studies with larger sample size included 6,094 total individuals and gave a prevalence estimate of 51.5% (95% CI 43.6-59.4; $I^2 = 96.8\%$, 95% CI 96.0-97.4, $p < 0.0001$) . The remaining 26 studies with "smaller" sample sizes included 1,340 total children and estimated pooled prevalence as 58.2% (95% CI 47.5-68.2; $I^2 = 90.9\%$, 95% CI 87.9-93.2, $p < 0.0001$) (*Additional Table 14*).

We also conducted analysis by study design. Cohort studies (n = 30) yielded a prevalence estimate of 49.6% (95% CI 42.7-56.4; $I^2 = 94.9\%$, 95% CI 93.6-95.9, $p < 0.0001$). In case-control studies (n = 18) the estimate was 68.1% (95% CI 56.5-77.8; $I^2 = 93.0\%$, 95% CI 90.4-94.9, $p < 0.0001$) and in cross-sectional (n = 4) 34.8% (95% CI 12.8-66.0; $I^2 = 96.7\%$, 95% CI 94.0-98.2, $p < 0.0001$) (*Additional Table 14*, *Figure 2*).

We assessed whether studies' country of origin influenced results. Studies in India gave an estimate of 68.9% (95% CI 54.9-80.1; $I^2 = 96.7\%$ (95% CI 94.0-98.2, $p < 0.0001$). Similarly, we found higher pooled prevalence estimates for studies from Turkey (76.3%, 95% CI 60.9-87.0; $I^2 = 91.1\%$, 95% CI 84.2-95.0, $p < 0.0001$). We also grouped studies by geography and economic development. Group 1: USA, Chile, Australia, Canada, Ireland, Japan, Spain; group 2: South Africa, China, Egypt, Iran, Turkey, Saudi Arabia; and group 3: Bangladesh, Thailand, and India. Prevalence was 37.2% (95% CI 29.7-45.5) for group 1 (n = 20), 61.8% (95% CI 53.2-69.7) for



For peer review only - http://bmjopen.bmj.com/site/about/guidelines.xhtml

group 2 (n = 19) and 70.8% (95% CI 58.3-80.7) for group 3 (n = 13) *(Additional Figure 2).* Variation attributable to heterogeneity was still high in the three subgroups ($I^2 > 90.0\%$).

Given the broad age range in included studies, we combined studies with only neonates[54, 65, 67, 69-72] and observed a prevalence estimate of 83.0% (95% CI 73.1-89.8) with less variation attributable to heterogeneity ($I^2 = 76.6\%$, 95% CI 51.0-88.9, p = 0.0003). In all other studies (n = 45) that included children of other age ranges, estimated prevalence was lower at 49.7% (95% CI 43.5-55.8; $I^2 = 95.2\%$, 95% CI 94.3-96.0, p < 0.0001) (*Additional Table 14, Additional Figure 3*).

**Post-hoc investigation to determine sources of heterogeneity**

To investigate the substantial heterogeneity observed in prevalence estimates, we incorporated study-specific characteristics (year of publication, total study sample size, quality score, study design, country group and clinical setting) as covariates in a random effects meta-regression model. We identified clinical setting and country groups as significant predictors, p < 0.01 *(Figure 3).* We found that the model fitted with all available covariates can explain 32.9% of $I^2$ with F = 4.57, p = 0.001 *(Additional Table 15).* We also conducted univariate meta-regressions for each of the six predictors *(Additional Figure 4).*

*Figure 3 Bubble plots of univariate meta-regressions.*

**Prevalence of vitamin D deficiency in children with sepsis and in those with respiratory tract infections**

A total of 889 (median 42, range 9 -160) patients had a diagnosis of sepsis, of which 565 (63.5%) were vitamin D deficient. Sixteen of the eighteen studies including septic patients were cohort

For peer review only



For peer review only - http://bmjopen.bmj.com/site/about/guidelines.xhtml

(88.9%) and two (11.1%) case-control *(Additional Table 16)*. Most studies originated from India (n = 7) Turkey (n = 3) or Ireland (n = 2) and 16 were published between 2014 and 2017. Thirteen studies took place in a PICU and the remaining[65, 67, 70, 72] in NICUs. We found that all studies were of medium to high quality (median NOS score 7, range 6 – 8). Pooled prevalence of vitamin D deficiency was 64.0% (95% CI 52.0-74.4) *(Figure 4)* and median (IQR), 68.5% (50.4 to 71.6%). Variation attributable to heterogeneity was high ($I^2$ = 89.3%, 95% CI 84.6-92.5, p < 0.0001). Funnel plot was symmetric (p > 0.05) suggesting no small-study effects (p = 0.81, Egger's test) *(Additional Figure 5)*.

*Figure 4 Pooled prevalence estimate for vitamin D deficiency in children with sepsis.*

We also separately analysed studies of patients admitted for respiratory tract infections (n = 25) such as acute lower respiratory tract infection (ALRTI), pneumonia and bronchiolitis. Of these 2,699 total individuals (median 50), 1,076 (39.9%) were vitamin D deficient. These studies were of high to medium quality (median NOS score 7, range 6 - 8). Most originated from India (n = 6) and Spain (n = 4). We found a prevalence estimate of 48.7% (95% CI 38.2-59.3; $I^2$ = 94.3%, 95% CI 92.7-95.6, p < 0.0001) and median (IQR) at 36.7% (24.3 to 83.6%) with marginally non-significant evidence of bias (p = 0.05, Egger's test) (*Table 1)*. We therefore applied the trim and fill method and obtained an adjusted pooled estimate of 37.4% (95% CI 27.6-48.4) after four studies were added.

**Sensitivity analysis for prevalence in children with sepsis**

Exclusion of the studies[64, 67, 72, 92] utilising thresholds other than < 50 nmol/L for deficiency yielded a similar estimate of prevalence at 62.0% (95% CI 47.3-74.7; $I^2$ = 89.7%, 95% CI 84.5-93.2, p < 0.0001) *(Additional Table 17)*.



For peer review only - http://bmjopen.bmj.com/site/about/guidelines.xhtml

We examined pooled prevalence estimates according to median sample size (< 42 versus ≥ 42). Studies with a smaller sample size (n = 9; 204 total individuals) showed a pooled prevalence estimate of 64.7% (95% CI 52.5-75.3) with moderate variation attributable to heterogeneity ($I^2$ = 57.9%, 95% CI 11.8-79.9, p = 0.015). For the remaining nine studies (sample sizes ≥ 42, 685 total individuals) the estimate was 63.2% (95% CI 44.6-78.5) with high variation attributable to heterogeneity ($I^2$ = 94.3%, 95% CI 91.1-96.3, p < 0.0001).

There was no material change in prevalence estimates when analysed according to study design. The sixteen cohort studies (749 total individuals) gave an estimate of 61.4% (95% CI 48.6-72.8) with high variation attributable to heterogeneity ($I^2$ = 88.8%, 95% CI 83.5-92.4, p < 0.0001). Case-control studies (n = 2; 140 total individuals) showed a pooled prevalence of 80.0% (95% CI 58.8-91.8; $I^2$ = 81.3%, 95% CI 20.5-95.6, p < 0.0001) *(Additional Table 17, Additional Figure 6).*

Studies from India (n = 7) gave a prevalence estimate of 66.0% (95% CI 51.4-78.1); $I^2$ = 81.1%, 95% CI 61.8-90.6, p < 0.0001). The three studies from Turkey assessing septic patients gave a pooled estimate of 59.2% (95% CI 13.6-93.1; $I^2$ = 97.8%, 95% CI 95.8-98.8, p < 0.0001) *(Additional Table 17).*

The pooled prevalence estimate in the four studies[65, 67, 70, 72] including neonates with sepsis was 73.7% (95% CI 60.3-83.8, $I^2$ = 76.0% 34.1-91.3, p = 0.006). The fourteen studies with children of different ages, excluding neonates, gave a pooled estimate of 60.7% (95% CI 45.5-74.0); $I^2$ = 90.1%, 95% CI 85.2-93.4, p < 0.0001) *(Additional Table 17).* Four of the studies[56, 61, 87, 89] included children admitted with either sepsis or respiratory tract infections.

**Mortality in acute and critically ill children**



For peer review only - http://bmjopen.bmj.com/site/about/guidelines.xhtml

We identified 18 cohort studies[8, 53, 56-59, 61, 64, 66, 72, 74-78, 82, 89, 92] assessing vitamin D status and mortality. These studies included a total of 2,463 individuals, from which 220 deaths (17.2%) were observed in 1,278 (51.9%) individuals with vitamin D deficiency and 99 deaths (8.4%) were observed in 1,185 individuals without deficiency (48.1%).

All 18 studies took place in a PICU apart from one[72], which considered only NICU patients. Sixteen of these studies (89.0%) were published between 2014 and 2017. Almost half (n = 7) of the studies originated from India. Quality scores ranged from 5 to 8 with a median of 6.5.

Using a random effects model, we found that vitamin D deficiency in critically ill children significantly increased the risk of death (OR 1.81, 95% CI 1.24-2.64, p = 0.002) with low, non-significant heterogeneity ($I^2$ = 25.7%, 95% CI 0.0-58.0, p = 0.153) (*Figure 5*). However, small-study effects cannot be easily excluded (p = 0.084, Egger's test) (*Additional Figure 7*) and the 95% prediction interval (0.71-4.62) included the null value.

*Figure 5 Pooled odds ratio (OR) of risk of mortality in vitamin D deficient versus vitamin D non-deficient acute and critically ill children.*

**Sensitivity analysis for mortality in acute and critically ill children**

We obtained similar results through the fixed effects model (OR 1.72, 95% CI 1.27-2.33, p = 0.0005) (*Additional Figure 8*). When excluding studies with thresholds other than < 50 nmol/L indicating deficiency, we found the association between vitamin D deficiency and increased risk of mortality still significant but lower, both with the random (OR 1.59, 95% CI 1.05-2.41, p = 0.028; $I^2$ = 24.3%, 95% CI 0.00-59.9, p = 0.191) and fixed effect models (OR 1.52, 95% CI 1.08-



For peer review only - http://bmjopen.bmj.com/site/about/guidelines.xhtml

2.13, p = 0.016) without clear indication of small-study effects (p = 0.120, Egger's test) *(Additional Table 18)*.

The association was positive but not-significant when pooling the seven studies from India with the random effects model (OR 1.08, 95% CI 0.70-1.69, p = 0.710; $I^2$ = 0.0% 0.0-62.4, p = 0.589) and similar with fixed effects (OR 1.08, 95% CI 0.70-1.69, p = 0.710) *(Additional Table 18)*.

**Mortality in patients with sepsis and respiratory tract infections**

We were unable to identify a sufficient number of studies assessing vitamin D and mortality for meta-analysis in individuals with sepsis. Three studies[8, 64, 67] measured vitamin D levels in paediatric patients with sepsis. One study[8] assessed mortality and did not find a significant association in children from 1 to 12 years with sepsis (n=124). None of the studies with children admitted for respiratory tract infections looked at the association between vitamin D deficiency and childhood mortality.

## DISCUSSION

Vitamin D deficiency is highly prevalent worldwide, even in countries with abundant sunshine. Studies have shown high prevalence of vitamin D deficiency in otherwise healthy children from high-income countries (9 to 24%) but also from middle and low-income countries in the Indian subcontinent (36 to 90%).[8]

We identified 52 studies representing a total of 7,434 children treated in ICU or emergency units for acute conditions who had blood 25(OH)D levels measured close to or upon admission. Our analysis shows that prevalence of vitamin D deficiency is generally high but very variable (range



For peer review only - http://bmjopen.bmj.com/site/about/guidelines.xhtml

5%[79] to 95%[60]) across ICU and emergency units in the paediatric population, particularly in individuals with sepsis. Importantly, our analysis showed a significantly increased risk of mortality in critically ill children with vitamin D deficiency. We carried out several analyses for sensitivity including fixed effects models, by study design, country group, age and sample size and found generally consistent results. A recently published meta-analysis[93] also investigated prevalence of vitamin D deficiency in critically ill children and its association with risk of mortality and showed similar results to ours. The study did not clearly report heterogeneity and small-study effects however, which we found to be critical limitations that must be addressed.

Subgroup analyses in patients with sepsis or respiratory tract infections demonstrated a high prevalence of vitamin D deficiency, consistent with the increased risk of bacterial or nosocomial infection in vitamin D deficient individuals identified elsewhere.[93]

Although sepsis is a leading cause of paediatric mortality and morbidity worldwide,[94] we found few studies assessing the relationship between vitamin D status and mortality in this population. We were unable to identify sufficient studies including patients with sepsis to perform a meta-analysis of vitamin D status and mortality. Sepsis remains an area of unmet need with high social and financial costs.[24] Diagnostic criteria,[95] a lack of adequate biomarkers[96] and targeted treatment remain important challenges in research on sepsis. We did not find studies that assessed the risk of mortality in relation to vitamin D deficiency in children admitted for respiratory tract infections either.

Strengths of our review include the large number of studies and large total sample size, allowing a high-powered investigation to identify meaningful associations. For our systematic review and meta-analysis, we followed pre-specified eligibility criteria and used the PRISMA[27] and MOOSE



For peer review only - http://bmjopen.bmj.com/site/about/guidelines.xhtml

guidelines[28] for reporting. We carried out multiple sensitivity analyses with few material differences in results. However, we note that the relationship between vitamin D deficiency and mortality was sensitive to study design and studies from India, probably due to the smaller number of individuals in those analyses. As expected for prevalence estimates, heterogeneity across studies was high overall. Only the prevalence analysis with neonates indicated somewhat lower variation attributable to heterogeneity ($I^2$ = 76.6%) along with a higher prevalence estimate (83.0%) compared to other analyses. We utilised meta-regression to investigate this substantial heterogeneity. From the six variables in our multi-variable model, only clinical setting and country groups were found to be significant predictors of pooled prevalence estimates of vitamin D deficiency and the full model could explain 32.9% of heterogeneity ($I^2$). Studies in NICU yielded higher prevalence estimates compared to studies in PICU. Studies from group 3 countries were also associated with higher prevalence estimates compared to studies from countries of group 1 and 2. Other variables, mainly individual patient characteristics such as age and ethnicity, were not directly available to us and may account for significant heterogeneity.

Our systematic review did not identify longitudinal studies with multiple time-point, pre-disease or pre-admission vitamin D measurements. The majority of studies were single centre with heterogeneous patient groups and relatively small sample sizes. Few studies accounted for important confounders that influence vitamin D levels such as age, gender, BMI, season of measurements, vitamin D supplementation and comorbidities. The relationship observed between vitamin D deficiency and mortality could be due to reverse causation and future studies will need to control for covariates and other confounders. Low vitamin D levels could also represent a chronically deficient state due to reduced sunlight exposure, because of chronic illness, lifestyle factors or different country latitudes. In addition, we cannot rule out measurement bias such as



For peer review only - http://bmjopen.bmj.com/site/about/guidelines.xhtml

dilution from intravenous fluids. Our results should be interpreted with caution since our review is based on evidence from observational studies. More research is warranted to strengthen the evidence and investigate whether vitamin D could be causally linked to acute or critical illness and what its contribution might be through various mechanisms such as anti-inflammatory or anti-microbial peptide responses.

Although included studies were generally of good quality, sample sizes varied considerably and were typically small. Half of the studies included less than 100 cases and only 10 (19.2 %) had a total sample size of more than 200 individuals. In addition, studies used a variety of definitions and age ranges to designate individuals as children. Our analysis only included mortality as a clinical outcome. A further general limitation is the difference in thresholds for vitamin D deficiency, particularly in the levels which are considered normal for infants and young children. Our assessment used the currently recommended threshold for deficiency (25(OH)D $\leq$ 50 nmol/L)[16] and a conservative estimate for studies which used different criteria. Although our review included a large number of studies and individuals, all studies were observational, and results could be subject to small-study effects.

Vitamin D remains an attractive biomarker and potential therapeutic agent in acute and critical care patients. Our review suggests that high quality focussed studies in each relevant paediatric population are needed first, which could then be followed by trials to establish safety and appropriate treatment regimens in children with acute or critical illness.

**Availability of data and materials**



For peer review only - http://bmjopen.bmj.com/site/about/guidelines.xhtml

Data and computational code used for processing and analysis are available at https://github.com/margarc/VitaminD_children

## Author contributions

AJBT conceived the study. AJBT and IT designed the study. MC collected data and performed the analysis with input from MAC, IT, ABJT and EE. MC and AJBT wrote the manuscript with contributions from all authors.

## Declaration of interests

The authors declare no conflicts of interest.

**Funding** Medical Research Council UK

## Acknowledgements

AJBT was supported by the Medical Research Council (UK MED-BIO Programme Fellowship, MR/L01632X/1).

**Ethics committee approval:** Not applicable.

## Main Figures

**Figure 1 Flow chart of study selection process**

**Figure 2 Pooled prevalence estimate for vitamin D deficiency in acute and critically ill children by study design**. Forest plot shows results from the random effects model. Each diamond represents the pooled proportion of 25(OH)D deficiency for each of the subgroups (case-control, cohort, cross-sectional study designs). The diamond at the bottom represents the overall pooled proportion of all the 52 studies together. Each square shows the prevalence estimate of each study and the horizontal line across each square represents the 95% confidence interval (CI) of the prevalence estimate.



For peer review only - http://bmjopen.bmj.com/site/about/guidelines.xhtml

**Figure 3 Bubble plots of univariate meta-regressions. Each study is represented by a circle.** Predictor variables; A clinical setting and B country groups are shown on the x-axis and the effect measure logit transformed proportion shown on the vertical (y-axis). NICU = Neonatal Intensive Care Unit; PICU = Pediatric Intensive Care Unit; grp = country group; country group 1 = USA, Chile, Australia, Canada, Ireland, Japan, Spain; country group 2 = South Africa, China, Egypt, Iran, Turkey, Saudi Arabia; and country group 3 = Bangladesh, Thailand, and India

**Figure 4 Pooled prevalence estimate for vitamin D deficiency in children with sepsis.** Forest plot shows result from the random effects model. The diamond represents the overall pooled proportion of 25(OH)D deficiency from the meta-analysis of the 18 studies. Each square shows the prevalence estimate of each study and the horizontal line across each square represents the 95% confidence interval (CI) of the prevalence estimate.

**Figure 5 Pooled odds ratio (OR) of risk of mortality in vitamin D deficient versus vitamin D non-deficient acute and critically ill children.** Forest plot shows result from the random effects model. Diamond represents the overall OR (with corresponding 95% Confidence Interval). Each square shows the odds ratio of each study and the horizontal line across each square represents the 95% confidence interval (CI) of the estimate.

## Supplementary Material

**Additional Tables**

Additional Table 1 PRISMA Checklist 2009

Additional Table 2A Search terms used in OVID

Additional Table 2B Search terms used in PubMed

Additional Table 3A Newcastle Ottawa study quality scoring system (cohort studies)

Additional Table 3B Newcastle Ottawa study quality scoring system (case-control studies)

Additional Table 3C Newcastle Ottawa study quality scoring system (cross sectional studies)

Additional Table 4A Circulating 25(OH)D threshold levels used in the selected studies

Additional Table 4B Circulating 25(OH)D threshold levels used in the selected studies for prevalence in sepsis

Additional Table 5 Studies with thresholds other than <50 nmol/L

Additional Table 6 Excluded studies

Additional Table 7 Characteristics of the 52 included studies

Additional Table 8 Objectives and outcomes of included studies

Additional Table 9 Assay used in each study to measure Vitamin D levels

Additional Table 10 Funding and ethical approval of included studies



For peer review only - http://bmjopen.bmj.com/site/about/guidelines.xhtml

For peer review only

Additional Table 11 Age groups of children in each study

Additional Table 12 Prevalence of vitamin D deficiency in each study of acute and critically ill children (sorted from highest to lowest)

Additional Table 13 Characteristics of studies used in the meta-analysis for prevalence

Additional Table 14 Sensitivity analyses for prevalence of vitamin D deficiency in acute and critically ill children

Additional Table 15 Multivariate meta-regression model for prevalence

Additional Table 16 Characteristics of studies included in the meta-analysis for prevalence in individuals with sepsis

Additional Table 17 Sensitivity analyses for prevalence of vitamin D deficiency in acute and critically ill children with sepsis

Additional Table 18 Sensitivity analyses for mortality

## Additional Figures

Additional Figure 1 Funnel plot of studies of prevalence of vitamin D deficiency in acute and critically ill children

Additional Figure 2 Pooled prevalence estimate for vitamin D deficiency in acute and critically ill children (subgroup analysis by country group)

Additional Figure 3 Pooled prevalence estimate for vitamin D deficiency in acute and critically ill children (subgroup analysis of neonates versus all other age groups)

Additional Figure 4 Bubble plots of univariate meta-regressions.

Additional Figure 5 Funnel plot for prevalence of vitamin D deficiency in acute and critically ill children with sepsis

Additional Figure 6 Pooled prevalence estimate for vitamin D deficiency in acute and critically ill children with sepsis (subgroup analysis by study design)

Additional Figure 7 Funnel plot of risk of mortality in vitamin D deficient versus vitamin D non-deficient acute and critically ill children

Additional Figure 8 Pooled odds ratio and 95% CI for risk of mortality in vitamin D deficient versus vitamin D non-deficient acute and critically ill children (fixed effects model)



For peer review only - http://bmjopen.bmj.com/site/about/guidelines.xhtml

## References

1. Shuler FD, Wingate MK, Moore GH, *et al.* Sports health benefits of vitamin d. *Sports health* 2012;4(6):496-501.
2. Verceles AC, Weiler B, Koldobskiy D, *et al.* Association Between Vitamin D Status and Weaning From Prolonged Mechanical Ventilation in Survivors of Critical Illness. *Respir Care* 2015;60(7):1033-9.
3. Braegger C, Campoy C, Colomb V, *et al.* Vitamin D in the healthy European paediatric population. *J Pediatr Gastroenterol Nutr* 2013;56(6):692-701.
4. Holick MF, Binkley NC, Bischoff-Ferrari HA, *et al.* Evaluation, treatment, and prevention of vitamin D deficiency: an Endocrine Society clinical practice guideline. *J Clin Endocrinol Metab* 2011;96(7):1911-30.
5. Holick MF. Vitamin D deficiency. *N Engl J Med* 2007;357(3):266-81.
6. Aranow C. Vitamin D and the immune system. *J Investig Med* 2011;59(6):881-6.
7. Zhang YP, Wan YD, Sun TW, *et al.* Association between vitamin D deficiency and mortality in critically ill adult patients: a meta-analysis of cohort studies. *Crit Care* 2014;18(6):684.
8. Ponnarmeni S, Kumar Angurana S, Singhi S, *et al.* Vitamin D deficiency in critically ill children with sepsis. *Paediatr Int Child Health* 2016;36(1):15-21.
9. Kulie T, Groff A, Redmer J, *et al.* Vitamin D: an evidence-based review. *J Am Board Fam Med* 2009;22(6):698-706.
10. Hewison M. Antibacterial effects of vitamin D. *Nat Rev Endocrinol* 2011;7(6):337-45.
11. Kempker JA, West KG, Kempker RR, *et al.* Vitamin D status and the risk for hospital-acquired infections in critically ill adults: a prospective cohort study. *PLoS One* 2015;10(4):e0122136.
12. Martineau AR, Jolliffe DA, Hooper RL, *et al.* Vitamin D supplementation to prevent acute respiratory tract infections: systematic review and meta-analysis of individual participant data. *BMJ* 2017;356:i6583.
13. Absoud M, Cummins C, Lim MJ, *et al.* Prevalence and predictors of vitamin D insufficiency in children: a Great Britain population based study. *PLoS One* 2011;6(7):e22179.
14. Greer FR. Defining vitamin D deficiency in children: beyond 25-OH vitamin D serum concentrations. *Pediatrics* 2009;124(5):1471-3.
15. Abou-Zahr R, Kandil SB. A pediatric critical care perspective on vitamin D. *Pediatr Res* 2015;77(1-2):164-7.
16. Ross AC, Manson JE, Abrams SA, *et al.* The 2011 report on dietary reference intakes for calcium and vitamin D from the Institute of Medicine: what clinicians need to know. *J Clin Endocrinol Metab* 2011;96(1):53-8.
17. Ala-Kokko TI, Mutt SJ, Nisula S, *et al.* Vitamin D deficiency at admission is not associated with 90-day mortality in patients with severe sepsis or septic shock: Observational FINNAKI cohort study. *Ann Med* 2016;48(1-2):67-75.
18. Putzu A, Belletti A, Cassina T, *et al.* Vitamin D and outcomes in adult critically ill patients. A systematic review and meta-analysis of randomized trials. *J Crit Care* 2017;38:109-14.
19. Han JE, Jones JL, Tangpricha V, *et al.* High Dose Vitamin D Administration in Ventilated Intensive Care Unit Patients: A Pilot Double Blind Randomized Controlled Trial. *J Clin Transl Endocrinol* 2016;4:59-65.
20. Quraishi SA, De Pascale G, Needleman JS, *et al.* Effect of Cholecalciferol Supplementation on Vitamin D Status and Cathelicidin Levels in Sepsis: A Randomized, Placebo-Controlled Trial. *Crit Care Med* 2015;43(9):1928-37.
21. Singer M, Deutschman CS, Seymour CW, *et al.* The Third International Consensus Definitions for Sepsis and Septic Shock (Sepsis-3). *JAMA* 2016;315(8):801-10.



For peer review only - http://bmjopen.bmj.com/site/about/guidelines.xhtml

22. NHS-England. Improving outcomes for patients with sepsis: a cross-system action plan 2015 [Available from: https://www.england.nhs.uk/ourwork/part-rel/sepsis/.
23. Plunkett A, Tong J. Sepsis in children. *BMJ* 2015;350:h3017.
24. Weiss SL, Fitzgerald JC, Pappachan J, *et al.* Global epidemiology of pediatric severe sepsis: the sepsis prevalence, outcomes, and therapies study. *Am J Respir Crit Care Med* 2015;191(10):1147-57.
25. Mayr FB, Yende S, Angus DC. Epidemiology of severe sepsis. *Virulence* 2014;5(1):4-11.
26. De Pascale G, Ranzani OT, Nseir S, *et al.* Intensive care unit patients with lower respiratory tract nosocomial infections: the ENIRRIs project. *ERJ Open Res* 2017;3(4).
27. Moher D, Liberati A, Tetzlaff J, *et al.* Preferred reporting items for systematic reviews and meta-analyses: the PRISMA statement. *PloS Med* 2009;6.
28. Stroup DF, Berlin JA, Morton SC, *et al.* Meta-analysis of observational studies in epidemiology: a proposal for reporting. Meta-analysis Of Observational Studies in Epidemiology (MOOSE) group. *JAMA* 2000;283(15):2008-12.
29. Wells GA SB OCD, Peterson J, Welch V, Losos M,Tugwell P. The Newcastle-Ottawa Scale (NOS) for assessing the quality of nonrandomised studies in meta-analyses [Available from: http://www.ohri.ca/programs/clinical_epidemiology/oxford.asp.
30. Newcombe RG. Two-sided confidence intervals for the single proportion: comparison of seven methods. *Stat Med* 1998;17(8):857-72.
31. DerSimonian R, Laird N. Meta-analysis in clinical trials revisited. *Contemp Clin Trials* 2015;45(Pt A):139-45.
32. Higgins JP, Thompson SG, Spiegelhalter DJ. A re-evaluation of random-effects meta-analysis. *J R Stat Soc Ser A Stat Soc* 2009;172(1):137-59.
33. Borenstein M, Hedges LV, Higgins JP, *et al.* A basic introduction to fixed-effect and random-effects models for meta-analysis. *Res Synth Methods* 2010;1(2):97-111.
34. Haidich A-BJ. Meta-analysis in medical research. *Hippokratia* 2010;14(Suppl 1):29.
35. Lau J, Ioannidis JP, Schmid CH. Quantitative synthesis in systematic reviews. *Ann Intern Med* 1997;127(9):820-6.
36. Ioannidis JP, Patsopoulos NA, Evangelou E. Uncertainty in heterogeneity estimates in meta-analyses. *BMJ* 2007;335(7626):914-6.
37. Higgins JP, Thompson SG, Deeks JJ, *et al.* Measuring inconsistency in meta-analyses. *BMJ* 2003;327(7414):557-60.
38. Higgins JP, Thompson SG. Quantifying heterogeneity in a meta-analysis. *Stat Med* 2002;21(11):1539-58.
39. Egger M, Davey Smith G, Schneider M, *et al.* Bias in meta-analysis detected by a simple, graphical test. *BMJ* 1997;315(7109):629-34.
40. Sterne JA, Sutton AJ, Ioannidis JP, *et al.* Recommendations for examining and interpreting funnel plot asymmetry in meta-analyses of randomised controlled trials. *BMJ* 2011;343:d4002.
41. Duval S, Tweedie R. Trim and fill: A simple funnel-plot-based method of testing and adjusting for publication bias in meta-analysis. *Biometrics* 2000;56(2):455-63.
42. Panityakul T, Bumrungsup C, Knapp G. On estimating residual heterogeneity in random-effects meta-regression: a comparative study. *J Stat Theory Appl* 2013;12(3):253.
43. Knapp G, Hartung J. Improved tests for a random effects meta-regression with a single covariate. *Stat Med* 2003;22(17):2693-710.
44. Schwarzer G. meta: An R package for meta-analysis. *R news* 2007;7(3):40-5.
45. Viechtbauer W. Conducting meta-analyses in R with the metafor package. *J Stat Softw* 2010;36(3).



For peer review only - http://bmjopen.bmj.com/site/about/guidelines.xhtml

46. Garg D, Sharma VK, Karnawat B. Association of serum vitamin D with acute lower respiratory infection in Indian children under 5 years: a case control study. *Int J Contemp Pediatrics* 2016;3(4):1164-9.
47. Aydemir G, Cekmez F, Kalkan G*, et al.* High serum 25-hydroxyvitamin D levels are associated with pediatric sepsis. *Tohoku J Exp Med* 2014;234(4):295-8.
48. Gamal TS, Madiha AS, Hanan MK*, et al.* Neonatal and Maternal 25-OH Vitamin D Serum Levels in Neonates with Early-Onset Sepsis. *Children (Basel)* 2017;4(5).
49. Seliem MS, Abdel Haie OM, Mansour AI*, et al.* The relation between vitamin D level and increased risk for early-onset neonatal sepsis in full-term infants. *Medical Research Journal* 2016;15(1):16-21.
50. McNally JD, Leis K, Matheson LA*, et al.* Vitamin D deficiency in young children with severe acute lower respiratory infection. *Pediatr Pulmonol* 2009;44(10):981-8.
51. McNally JD, Doherty DR, Lawson ML*, et al.* The relationship between vitamin D status and adrenal insufficiency in critically ill children. *J Clin Endocrinol Metab* 2013;98(5):E877-81.
52. McNally JD, Iliriani K, Pojsupap S*, et al.* Rapid normalization of vitamin D levels: a meta-analysis. *Pediatrics* 2015;135(1):e152-66.
53. McNally JD, Menon K, Chakraborty P*, et al.* The association of vitamin D status with pediatric critical illness. *Pediatrics* 2012;130(3):429-36.
54. Karatekin G, Kaya A, Salihoglu O*, et al.* Association of subclinical vitamin D deficiency in newborns with acute lower respiratory infection and their mothers. *Eur J Clin Nutr* 2009;63(4):473-7.
55. Vo P, Koppel C, Espinola JA*, et al.* Vitamin D Status at the Time of Hospitalization for Bronchiolitis and Its Association with Disease Severity. *J Pediatr* 2018;203:416-22.e1.
56. Shah SK, Kabra SK, Gupta N*, et al.* Vitamin D Deficiency and Parathyroid Response in Critically-ill Children: Association with Illness Severity and Clinical Outcomes. *Indian Pediatr* 2016;53(6):479-84.
57. Sankar J, Lotha W, Ismail J*, et al.* Vitamin D deficiency and length of pediatric intensive care unit stay: a prospective observational study. *Ann Intensive Care* 2016;6(1):3.
58. Dayal D, Kumar S, Sachdeva N*, et al.* Fall in Vitamin D Levels during Hospitalization in Children. *Int J Pediatr* 2014;2014:291856.
59. Prasad S, Raj D, Warsi S*, et al.* Vitamin D Deficiency and Critical Illness. *Indian J Pediatr* 2015;82(11):991-5.
60. Wayse V, Yousafzai A, Mogale K*, et al.* Association of subclinical vitamin D deficiency with severe acute lower respiratory infection in Indian children under 5 y. *Eur J Clin Nutr* 2004;58(4):563-7.
61. Ebenezer K, Job V, Antonisamy B*, et al.* Serum Vitamin D Status and Outcome among Critically Ill Children Admitted to the Pediatric Intensive Care Unit in South India. *Indian J Pediatr* 2016;83(2):120-5.
62. Narang GS, Arora S, Kukreja S. Association of Vitamin D Deficiency with Acute Lower Respiratory Infection in Toddlers. *Journal of Nepal Paediatric Society* 2016;36(1):14-6.
63. Jat KR, Kaur J, Guglani V. Vitamin D and Pneumonia in Children: A Case Control Study. *J Pulm Med Respir Res* 2016;2(004).
64. Sankar J, Ismail J, Das R*, et al.* Effect of Severe Vitamin D Deficiency at Admission on Shock Reversal in Children With Septic Shock. *J Intensive Care Med* 2017.
65. Dhandai R, Jajoo M, Singh A*, et al.* Association of vitamin D deficiency with an increased risk of late-onset neonatal sepsis. *Paediatr Int Child Health* 2018;38(3):193-7.
66. Asilioglu N, Cigdem H, Paksu MS. Serum Vitamin D Status and Outcome in Critically Ill Children. *Indian J Crit Care Med* 2017;21(10):660-4.
67. Say B, Uras N, Sahin S*, et al.* Effects of cord blood vitamin D levels on the risk of neonatal sepsis in premature infants. *Korean J Pediatr* 2017;60(8):248-53.
68. Cayir A, Turan MI, Ozkan O*, et al.* Vitamin D levels in children diagnosed with acute otitis media. *J Pak Med Assoc* 2014;64(11):1274-7.



For peer review only - http://bmjopen.bmj.com/site/about/guidelines.xhtml

69. Cetinkaya M, Cekmez F, Buyukkale G, *et al.* Lower vitamin D levels are associated with increased risk of early-onset neonatal sepsis in term infants. *J Perinatol* 2015;35(1):39.
70. Cizmeci MN, Kanburoglu MK, Akelma AZ, *et al.* Cord-blood 25-hydroxyvitamin D levels and risk of early-onset neonatal sepsis: a case-control study from a tertiary care center in Turkey. *Eur J Pediatr* 2015;174(6):809-15.
71. Dinlen N, Zenciroglu A, Beken S, *et al.* Association of vitamin D deficiency with acute lower respiratory tract infections in newborns. *J Matern Fetal Neonatal Med* 2016;29(6):928-32.
72. Onwuneme C, Martin F, McCarthy R, *et al.* The Association of Vitamin D Status with Acute Respiratory Morbidity in Preterm Infants. *J Pediatr* 2015;166(5):1175-80.e1.
73. Madden K, Feldman HA, Smith EM, *et al.* Vitamin D deficiency in critically ill children. *Pediatrics* 2012;130(3):421-8.
74. Rippel C, South M, Butt WW, *et al.* Vitamin D status in critically ill children. *Intensive Care Med* 2012;38(12):2055-62.
75. Korwutthikulrangsri M, Mahachoklertwattana P, Lertbunrian R, *et al.* Vitamin D deficiency and adrenal function in critically ill children. *J Med Assoc Thai* 2015;98(4):365-72.
76. Elmoneim A, Rhill R, Rahalli M, *et al.* Vitamin D level in pediatric intensive care unit (PICU) patients: its relation to severity of illness. *Pediat Therapeut* 2016;6(293):2161-0665.
77. García-Soler P, Morales-Martínez A, Rosa-Camacho V, *et al.* Vitamin D deficiency and morbimortality in critically ill paediatric patients. *Anales de Pediatría (English Edition)* 2017;87(2):95-103.
78. Badawi NES, Algebaly HAF, El Sayed R, *et al.* Vitamin D deficiency in critically ill children. *Kasr Al Ainy Medical Journal* 2017;23(1):6.
79. Roth DE, Jones AB, Prosser C, *et al.* Vitamin D status is not associated with the risk of hospitalization for acute bronchiolitis in early childhood. *Eur J Clin Nutr* 2009;63(2):297-9.
80. Cebey-Lopez M, Pardo-Seco J, Gomez-Carballa A, *et al.* Role of Vitamin D in Hospitalized Children With Lower Tract Acute Respiratory Infections. *J Pediatr Gastroenterol Nutr* 2016;62(3):479-85.
81. Alonso MA, Pallavicini ZF, Rodriguez J, *et al.* Can vitamin D status be assessed by serum 25OHD in children? *Pediatr Nephrol* 2015;30(2):327-32.
82. Ayulo M, Jr., Katyal C, Agarwal C, *et al.* The prevalence of vitamin D deficiency and its relationship with disease severity in an urban pediatric critical care unit. *Endocr Regul* 2014;48(2):69-76.
83. Rey C, Sanchez-Arango D, Lopez-Herce J, *et al.* Vitamin D deficiency at pediatric intensive care admission. *J Pediatr (Rio J)* 2014;90(2):135-42.
84. Onwuneme C, Carroll A, Doherty D, *et al.* Inadequate vitamin D levels are associated with culture positive sepsis and poor outcomes in paediatric intensive care. *Acta Paediatr* 2015;104(10):e433-8.
85. Jia K-P, Zhao L-F, Feng N, *et al.* Lower level of vitamin D3 is associated with susceptibility to acute lower respiratory tract infection (ALRTI) and severity: a hospital based study in Chinese infants. *Int J Clin Exp Med* 2017;10(5):7997-8003.
86. Halwany AS, Deghady AAE, Moustafa AAA, *et al.* Study of Serum Vitamin D Level among Critically Ill Patients Admitted to Alexandria University Pediatric Intensive Care Unit. *JMSCR* 2017;5(4):20776-81.
87. Li W, Cheng X, Guo L, *et al.* Association between serum 25-hydroxyvitamin D concentration and pulmonary infection in children. *Medicine* 2018;97(1):e9060.
88. El Sakka AS, Imam SS, Amer HA, *et al.* Vitamin D deficiency and low hemoglobin level as risk factors for severity of acute lower respiratory tract infections in Egyptian children: A case-control study. *Egyptian Pediatric Association Gazette* 2014;62(1):1-7.
89. Bustos R, Rodriguez-Nunez I, Peña RZ, *et al.* Vitamin D deficiency in children admitted to the paediatric intensive care unit. *Revista chilena de pediatria* 2016;87(6):480-6.



For peer review only - http://bmjopen.bmj.com/site/about/guidelines.xhtml

90. Hurwitz JL, Jones BG, Penkert RR*, et al.* Low Retinol-Binding Protein and Vitamin D Levels Are Associated with Severe Outcomes in Children Hospitalized with Lower Respiratory Tract Infection and Respiratory Syncytial Virus or Human Metapneumovirus Detection. *J Pediatr* 2017;187:323-7.
91. Yaghmaie B, Sayarifard F, Modarresi M*, et al.* Investigation of serum 25 hydroxy vitamin D level in critically ill children and its relation with demographic and clinical risk factors. *Biomed Res* 2017;28(5).
92. El-Gamasy M, Eldeeb M, Abdelmageed M. Prognostic Value of Vitamin D Status in Pediatric Patients with Acute Kidney Injury in Tanta University Emergency Hospital, Egypt. *J Emerg Intern Med* 2017;1(1):5.
93. McNally JD, Nama N, O'Hearn K*, et al.* Vitamin D deficiency in critically ill children: a systematic review and meta-analysis. *Crit Care* 2017;21(1):287.
94. Randolph AG, McCulloh RJ. Pediatric sepsis: important considerations for diagnosing and managing severe infections in infants, children, and adolescents. *Virulence* 2014;5(1):179-89.
95. Vincent JL. The Clinical Challenge of Sepsis Identification and Monitoring. *PLoS Med* 2016;13(5):e1002022.
96. Mathias B, Mira JC, Larson SD. Pediatric sepsis. *Curr Opin Pediatr* 2016;28(3):380-7.

For peer review only



For peer review only - http://bmjopen.bmj.com/site/about/guidelines.xhtml

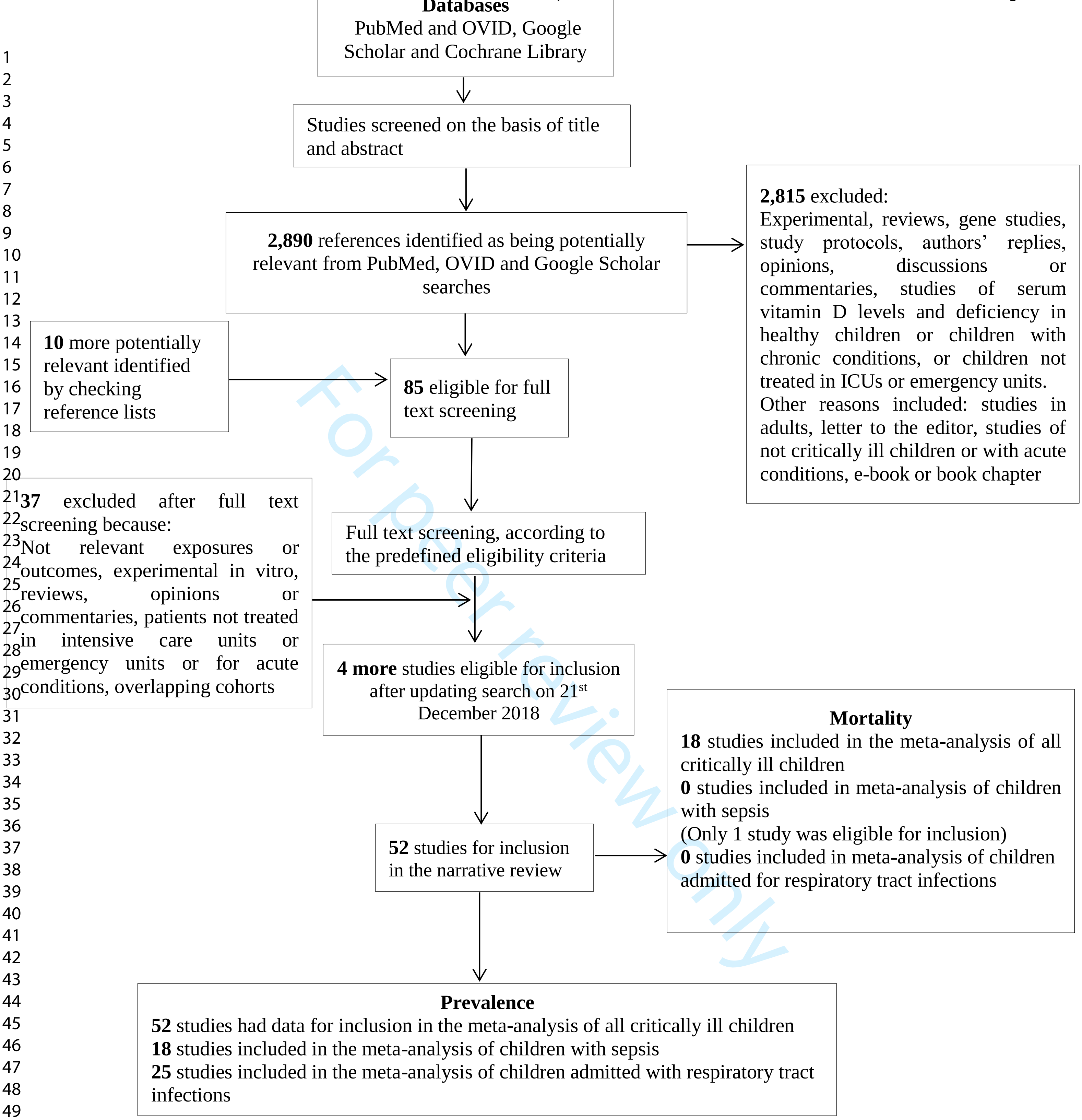


**Figure 1** Flow chart of study selection process

For peer review only

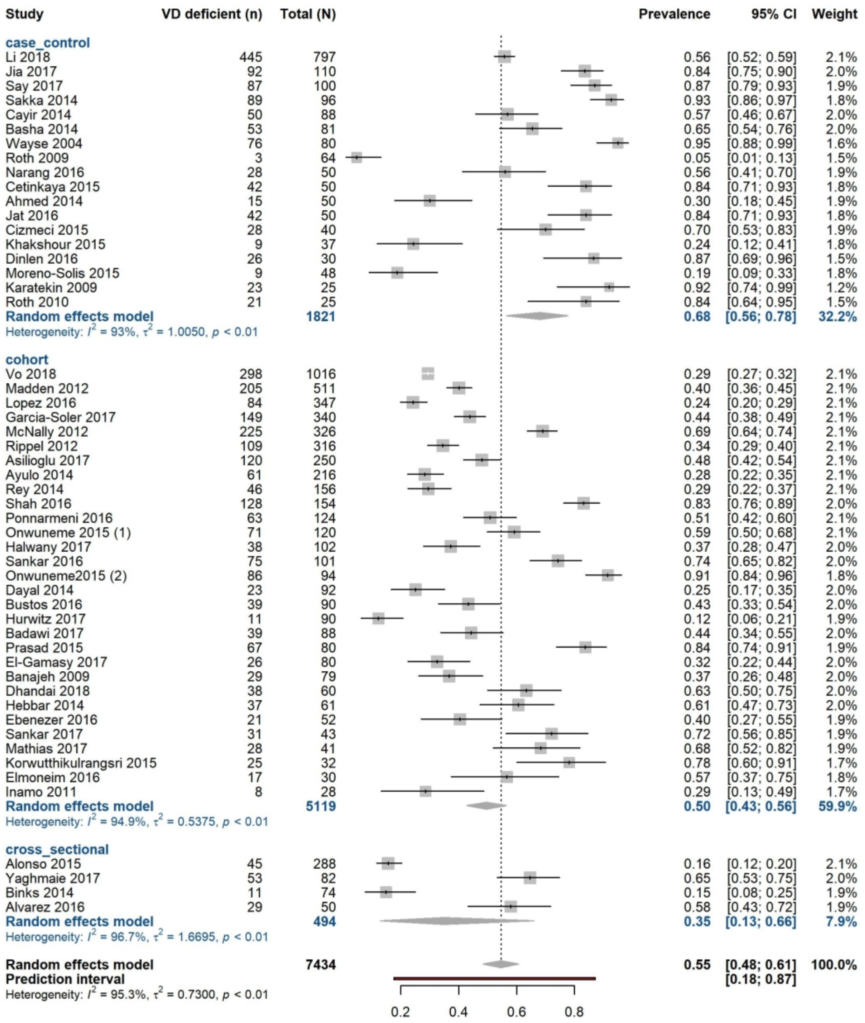


Figure 2 Pooled prevalence estimate for vitamin D deficiency in acute and critically ill children by study design. Forest plot shows results from the random effects model. Each diamond represents the pooled proportion of 25(OH)D deficiency for each of the subgroups (case-control, cohort, cross-sectional study designs). The diamond at the bottom represents the overall pooled proportion of all the 52 studies together. Each square shows the prevalence estimate of each study and the horizontal line across each square represents the 95% confidence interval (CI) of the prevalence estimate.

203x254mm (300 x 300 DPI)

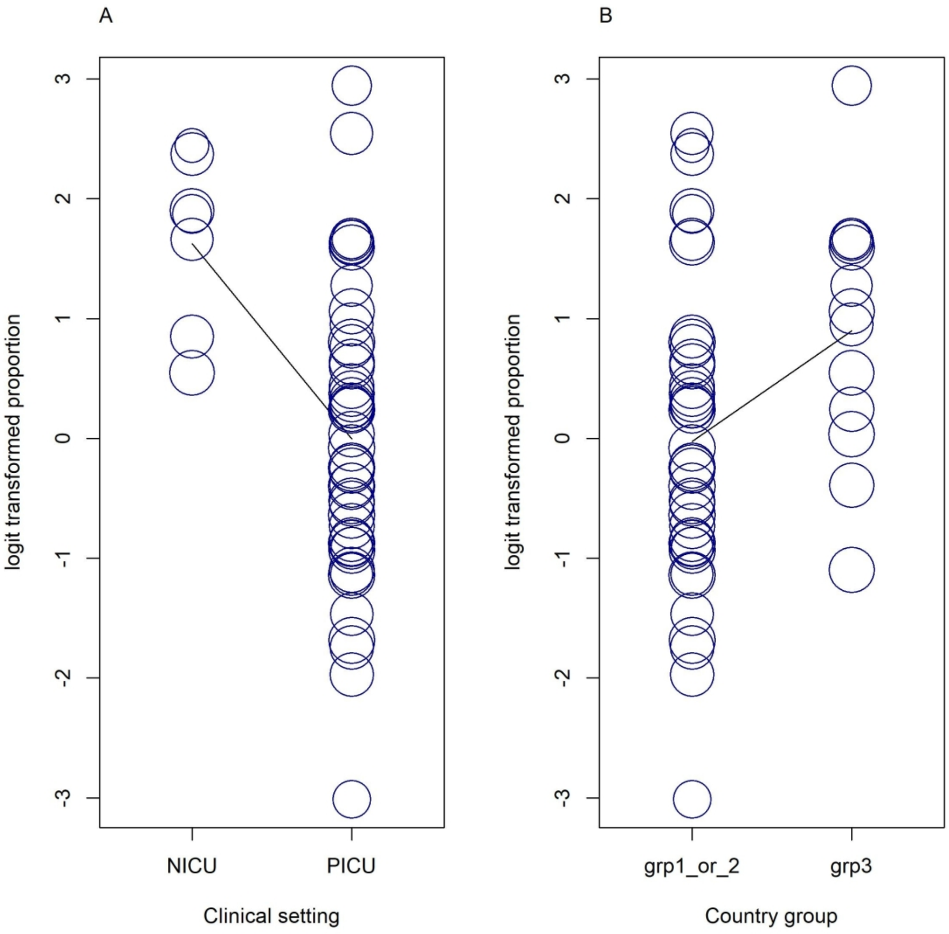


Figure 3 Bubble plots of univariate meta-regressions. Each study is represented by a circle. Predictor variables; A clinical setting and B country groups are shown on the x-axis and the effect measure logit transformed proportion shown on the vertical (y-axis). NICU = Neonatal Intensive Care Unit; PICU = Pediatric Intensive Care Unit; grp = country group; country group 1 = USA, Chile, Australia, Canada, Ireland, Japan, Spain; country group 2 = South Africa, China, Egypt, Iran, Turkey, Saudi Arabia; and country group 3 = Bangladesh, Thailand, and India

203x203mm (300 x 300 DPI)

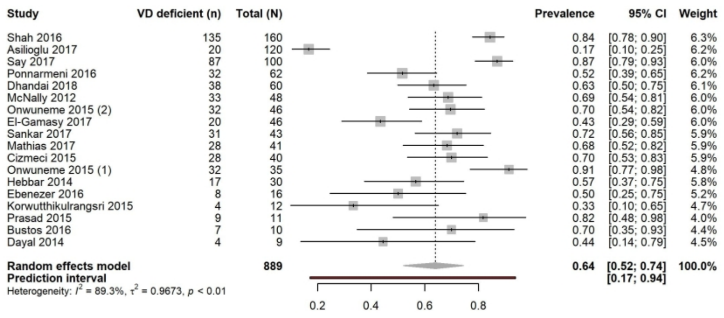


Figure 4 Pooled prevalence estimate for vitamin D deficiency in children with sepsis. Forest plot shows result from the random effects model. The diamond represents the overall pooled proportion of 25(OH)D deficiency from the meta-analysis of the 18 studies. Each square shows the prevalence estimate of each study and the horizontal line across each square represents the 95% confidence interval (CI) of the prevalence estimate.

254x152mm (300 x 300 DPI)

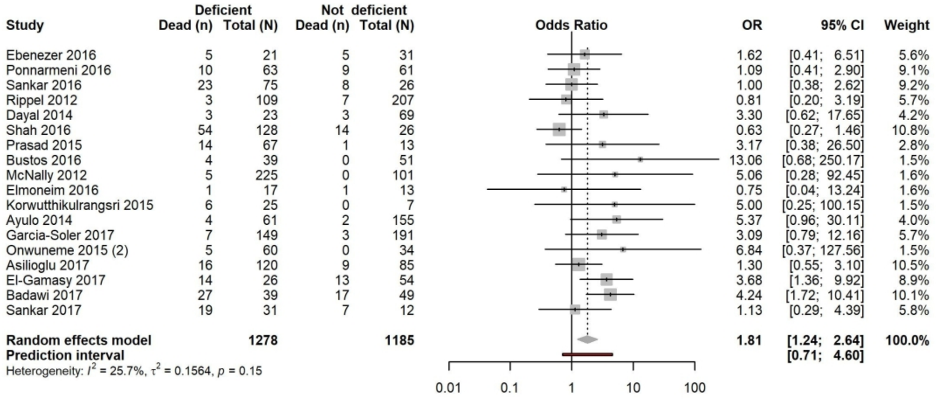


Figure 5 Pooled odds ratio (OR) of risk of mortality in vitamin D deficient versus vitamin D non-deficient acute and critically ill children. Forest plot shows result from the random effects model. Diamond represents the overall OR (with corresponding 95% Confidence Interval). Each square shows the odds ratio of each study and the horizontal line across each square represents the 95% confidence interval (CI) of the estimate.

203x127mm (300 x 300 DPI)

# Supplementary Material

## Table of Contents

Additional Table 1 PRISMA Checklist 2009 .......... 3
Additional Table 2A Search terms used in OVID .......... 5
Additional Table 2B Search terms used in PubMed .......... 6
Additional Table 3A Newcastle Ottawa study quality scoring system (cohort studies) .......... 7
Additional Table 3B Newcastle Ottawa study quality scoring system (case-control studies) .......... 8
Additional Table 3C Newcastle Ottawa study quality scoring system (cross-sectional studies) .......... 9
Additional Table 4A Circulating 25(OH)D threshold levels used in the selected studies .......... 10
Additional Table 4B Circulating 25(OH)D threshold levels used in the selected studies for prevalence in sepsis .......... 13
Additional Table 5 Studies with thresholds other than < 50 nmol/L .......... 14
Additional Table 6 Excluded studies .......... 15
Additional Table 7 Characteristics of the 52 included studies .......... 16
Additional Table 8 Objectives and outcomes of included studies .......... 22
Additional Table 9 Assay used in each study to measure Vitamin D levels .......... 26
Additional Table 10 Funding and ethical approval of included studies .......... 27
Additional Table 11 Age groups of children in each study .......... 30
Additional Table 12 Prevalence of vitamin D deficiency in each study of acute and critically ill children (sorted from highest to lowest) .......... 31
Additional Table 13 Characteristics of studies used in the meta-analysis of prevalence .......... 33
Additional Table 14 Sensitivity analyses for prevalence of vitamin D deficiency in acute and critically ill children .......... 35
Additional Table 15 Multivariate meta-regression model for prevalence .......... 36
Additional Table 16 Characteristics of studies included in the meta-analysis for prevalence in individuals with sepsis .......... 37
Additional Table 17 Sensitivity analyses for prevalence of vitamin D deficiency in acute and critically ill children with sepsis .......... 38
Additional Table 18 Sensitivity analyses for mortality. Pooled odds ratios for risk of mortality in deficient versus not deficient children .......... 39
Additional Figures .......... 40



For peer review only - http://bmjopen.bmj.com/site/about/guidelines.xhtml

Additional Figure 1 Funnel plot of studies of prevalence of vitamin D deficiency in acute and critically ill children. .......... 40

Additional Figure 2. Pooled prevalence estimate for vitamin D deficiency in acute and critically ill children (by country group). .......... 41

Additional Figure 3. Pooled prevalence estimates for vitamin D deficiency in acute and critically ill children (neonates versus all other age groups). .......... 42

Additional Figure 4 Bubble plots of univariate meta-regressions. .......... 43

Additional Figure 5 Funnel plot for prevalence of vitamin D deficiency in acute and critically ill children with sepsis. .......... 44

Additional Figure 6 Pooled prevalence estimate for vitamin D deficiency in acute and critically ill children with sepsis (subgroup analysis by study design). .......... 45

Additional Figure 7 Funnel plot of risk of mortality in vitamin D deficient versus vitamin D non-deficient acute and critically ill children. .......... 46

Additional Figure 8 Pooled odds ratio (OR) and 95% CI for risk of mortality in vitamin D deficient versus vitamin D non-deficient acute and critically ill children (fixed effects model). .......... 47

For peer review only

**Additional Table 1 PRISMA Checklist 2009**

| Section/topic | # | Checklist item | Reported on page # |
|---|---|---|---|
| **TITLE** | | | |
| Title | 1 | Identify the report as a systematic review, meta-analysis, or both. | 1 |
| **ABSTRACT** | | | |
| Structured summary | 2 | Provide a structured summary including, as applicable: background; objectives; data sources; study eligibility criteria, participants, and interventions; study appraisal and synthesis methods; results; limitations; conclusions and implications of key findings; systematic review registration number. | 2-3 |
| **INTRODUCTION** | | | |
| Rationale | 3 | Describe the rationale for the review in the context of what is already known. | 4-5 |
| Objectives | 4 | Provide an explicit statement of questions being addressed with reference to participants, interventions, comparisons, outcomes, and study design (PICOS). | 6-8 |
| **METHODS** | | | |
| Protocol and registration | 5 | Indicate if a review protocol exists, if and where it can be accessed (e.g., Web address), and, if available, provide registration information including registration number. | 9 |
| Eligibility criteria | 6 | Specify study characteristics (e.g., PICOS, length of follow-up) and report characteristics (e.g., years considered, language, publication status) used as criteria for eligibility, giving rationale. | 5-7 |
| Information sources | 7 | Describe all information sources (e.g., databases with dates of coverage, contact with study authors to identify additional studies) in the search and date last searched. | 6-7 |
| Search | 8 | Present full electronic search strategy for at least one database, including any limits used, such that it could be repeated. | 6 and supplementary material |
| Study selection | 9 | State the process for selecting studies (i.e., screening, eligibility, included in systematic review, and, if applicable, included in the meta-analysis). | 6-7 |
| Data collection process | 10 | Describe method of data extraction from reports (e.g., piloted forms, independently, in duplicate) and any processes for obtaining and confirming data from investigators. | 7 |
| Data items | 11 | List and define all variables for which data were sought (e.g., PICOS, funding sources) and any assumptions and simplifications made. | 7-8 |
| Risk of bias in individual studies | 12 | Describe methods used for assessing risk of bias of individual studies (including specification of whether this was done at the study or outcome level), and how this information is to be used in any data synthesis. | 8 |
| Summary measures | 13 | State the principal summary measures (e.g., risk ratio, difference in means). | 8 |
| Synthesis of results | 14 | Describe the methods of handling data and combining results of studies, if done, including measures of consistency (e.g., $I^2$) for each meta-analysis. | 8-10 |

**Additional Table 1 PRISMA Checklist 2009**



For peer review only - http://bmjopen.bmj.com/site/about/guidelines.xhtml

| Section/topic | # | Checklist item | Reported on page # |
|---|---|---|---|
| Risk of bias across studies | 15 | Specify any assessment of risk of bias that may affect the cumulative evidence (e.g., publication bias, selective reporting within studies). | 8 |
| Additional analyses | 16 | Describe methods of additional analyses (e.g., sensitivity or subgroup analyses, meta-regression), if done, indicating which were pre-specified. | 9-10 and 15 |
| **RESULTS** | | | |
| Study selection | 17 | Give numbers of studies screened, assessed for eligibility, and included in the review, with reasons for exclusions at each stage, ideally with a flow diagram. | 10 |
| Study characteristics | 18 | For each study, present characteristics for which data were extracted (e.g., study size, PICOS, follow-up period) and provide the citations. | 14-19 (supplementary material) |
| Risk of bias within studies | 19 | Present data on risk of bias of each study and, if available, any outcome level assessment (see item 12). | 5-7 (supplementary material) |
| Results of individual studies | 20 | For all outcomes considered (benefits or harms), present, for each study: (a) simple summary data for each intervention group (b) effect estimates and confidence intervals, ideally with a forest plot. | 13,15,18 and supplementary material |
| Synthesis of results | 21 | Present results of each meta-analysis done, including confidence intervals and measures of consistency. | 13 and supplementary material pages 32-36 |
| Risk of bias across studies | 22 | Present results of any assessment of risk of bias across studies (see Item 15). | 5-7 (supplementary material) |
| Additional analysis | 23 | Give results of additional analyses, if done (e.g., sensitivity or subgroup analyses, meta-regression [see Item 16]). | supplementary material pages 32-36 |
| **DISCUSSION** | | | |
| Summary of evidence | 24 | Summarize the main findings including the strength of evidence for each main outcome; consider their relevance to key groups (e.g., healthcare providers, users, and policy makers). | 19-21 |
| Limitations | 25 | Discuss limitations at study and outcome level (e.g., risk of bias), and at review-level (e.g., incomplete retrieval of identified research, reporting bias). | 20-22 |
| Conclusions | 26 | Provide a general interpretation of the results in the context of other evidence, and implications for future research. | 22 |
| **FUNDING** | | | |
| Funding | 27 | Describe sources of funding for the systematic review and other support (e.g., supply of data); role of funders for the systematic review. | 22 |

From: Moher D, Liberati A, Tetzlaff J, Altman DG, The PRISMA Group (2009). Preferred Reporting Items for Systematic Reviews and Meta-Analyses: The PRISMA Statement. PLoS Med 6(6): e1000097. doi:10.1371/journal.pmed1000097 For more information, visit: www.prisma-statement.org.



For peer review only - http://bmjopen.bmj.com/site/about/guidelines.xhtml

## Additional Table 2A Search terms used in OVID

| | |
|---|---|
| 1. | Vitamin D |
| 2. | Sepsis or septic shock |
| 3. | Vitamin D or cholecalciferol* or ergocalciferol or 25-hydroxyvitamin D or calcidiol or calcitriol |
| 4. | Intensive care unit* or critical care |
| 5. | Multiple organ dysfunction syndrome or multiple organ failure |
| 6. | (1 and 2) or 5 |
| 7. | ((1 or 3) and 2 or 4 |
| 8. | (1 or 3) and 2 |
| 9. | (1 or 3) and 4 |
| 10. | (1 or 3) and 5 |
| 11. | Sepsis |
| 12. | multi* organ dysfunction syndrome or multiple organ failure |
| 13. | multi* organ dysfunction syndrome or multi* organ failure |
| 14. | (2 or 8) and 3 |
| 15. | critical* ill or acute condition* or intensive care unit |
| 16. | child* or pediatric* |
| 17. | vitamin D or cholecalciferol or ergocalciferol or 25-hydroxyvitamin D or calcidiol or calcitriol |
| 18. | 15 and 16 and 17 |
| 19. | Vitamin D blood levels or 25-hydroxyvitamin |
| 20. | 5 and 3 and 15 and 16 |
| 21. | 2 and 15 and 16 and 17 |
| 22. | 16 and 2 |
| 23. | 16 and 2 and 3 |
| 24. | Pediatric* |
| 25. | Pediatric* and 5 and 2 |
| 26. | 24 and 3 and 15 |

For peer review only

| **Additional Table 2B Search terms used in PubMed** | |
|---|---|
| **Term searched** | |
| 1. | Vitamin D.mp. |
| 2. | (sepsis or septic shock).mp. |
| 3. | (vitamin D or cholecalciferol* or ergocalciferol or 25-hydroxyvitamin D or calcidiol or calcitriol).mp. |
| 4. | (intensive care unit* or critical care).mp. |
| 5. | (multi* organ dysfunction syndrome or multiple organ failure).mp. |
| 6. | (critical* ill or acute condition* or intensive care unit).mp. |
| 7. | (toddler or infant or child* or neonate* or baby or teenager or pediatric* or paediatric*).mp. |
| 8. | 3 and 6 and 2 and 7 |

For peer review only



For peer review only - http://bmjopen.bmj.com/site/about/guidelines.xhtml

| **Additional Table 3A Newcastle Ottawa study quality scoring system (cohort studies)** | | | | | | | | | |
|---|---|---|---|---|---|---|---|---|---|
| **Study** | **Selection** | | | | **Comparability** | **Outcome** | | | |
| | **Representativeness of the exposed cohort** | **Selection of the non-exposed cohort** | **Ascertainment of exposure** | **Demonstration that outcome of interest was not present at the start of the study** | **Comparability of cohorts on the basis of the design or analysis (maximum 2 stars)** | **Assessment of outcome** | **Was follow-up long enough for outcome to occur ( ≥ 28 days after admission to the ICU)** | **Adequacy of follow up of cohorts** | **Number of stars (out of 9 total)** |
| Ebenezer 2016 | 1 | 1 | 1 | 1 | 0 | 1 | 0 | 1 | 6 |
| Sankar 2016 | 1 | 1 | 1 | 1 | 2 | 1 | 0 | 1 | 8 |
| Rippel 2012 | 1 | 1 | 1 | 1 | 1 | 1 | 1 | 1 | 8 |
| Madden 2012 | 1 | 1 | 1 | 1 | 2 | 1 | 0 | 1 | 8 |
| McNally 2012 | 1 | 1 | 1 | 0 | 1 | 1 | 1 | 1 | 7 |
| Dayal 2014 | 1 | 1 | 1 | 1 | 0 | 1 | 0 | 1 | 6 |
| Ayulo 2014 | 1 | 1 | 1 | 1 | 2 | 1 | 0 | 0 | 7 |
| Bustos 2016 | 1 | 1 | 1 | 0 | 2 | 1 | 0 | 1 | 7 |
| Prasad 2015 | 1 | 1 | 1 | 0 | 2 | 1 | 0 | 1 | 7 |
| Onwuneme 2015 (2) | 1 | 1 | 1 | 1 | 2 | 1 | 0 | 1 | 8 |
| Inamo 2011 | 1 | 1 | 1 | 0 | 1 | 1 | 0 | 1 | 6 |
| Shah 2016 | 1 | 1 | 1 | 1 | 0 | 1 | 0 | 1 | 6 |
| Lopez 2016 | 1 | 1 | 1 | 1 | 2 | 1 | 0 | 1 | 8 |
| Garcia-Soler 2017 | 1 | 1 | 1 | 1 | 2 | 1 | 0 | 0 | 7 |
| Sankar 2017 | 1 | 1 | 1 | 1 | 2 | 1 | 0 | 1 | 8 |
| Asilioglu 2017 | 1 | 1 | 1 | 1 | 1 | 1 | 0 | 1 | 7 |
| Halwany 2017 | 1 | 1 | 1 | 1 | 1 | 1 | 0 | 0 | 6 |
| Hurwitz 2017 | 1 | 1 | 1 | 0 | 1 | 1 | 0 | 1 | 6 |
| Banajeh 2009 | 1 | 1 | 1 | 0 | 1 | 1 | 1 | 1 | 7 |
| Badawi 2017 | 1 | 1 | 1 | 1 | 1 | 1 | 1 | 1 | 8 |
| Rey 2014 | 1 | 1 | 1 | 1 | 2 | 0 | 0 | 1 | 7 |
| Ponnarmeni 2016 | 1 | 0 | 1 | 1 | 1 | 1 | 0 | 1 | 6 |
| Onwuneme 2015 (1) | 1 | 1 | 1 | 1 | 1 | 1 | 0 | 1 | 8 |
| El-Gamasy 2017 | 1 | 0 | 1 | 1 | 2 | 1 | 0 | 1 | 7 |
| Dhandai 2018 | 1 | 1 | 1 | 1 | 1 | 1 | 0 | 1 | 7 |
| Hebbar 2014 | 1 | 0 | 1 | 1 | 1 | 1 | 0 | 1 | 6 |
| Korwutthikulrangsri 2015 | 1 | 1 | 1 | 1 | 2 | 1 | 0 | 0 | 7 |
| Elmoneim 2016 | 1 | 1 | 1 | 1 | 0 | 1 | 0 | 1 | 7 |
| Vo 2018 | 1 | 1 | 1 | 1 | 2 | 1 | 0 | 1 | 8 |
| Mathias 2017 | 1 | 1 | 1 | 1 | 0 | 1 | 0 | 1 | 6 |

The Newcastle-Ottawa Scale (NOS) for assessing the quality of non-randomised studies in meta-analyses. Available at: http://www.ohri.ca/programs/clinical_epidemiology/oxford.asp.



For peer review only - http://bmjopen.bmj.com/site/about/guidelines.xhtml

**Additional Table 3B Newcastle Ottawa study quality scoring system (case-control studies)**

| Study | SELECTION | | | | COMPARABILITY | EXPOSURE | | | Number of stars (out of 9 total) |
|---|---|---|---|---|---|---|---|---|---|
| | Is the case definition adequate? | Representativeness of the cases | Selection of controls | Definition of controls | Comparability of cases and controls on the basis of the design or analysis (maximum 2 stars) | Ascertainment of exposure | Same method of ascertainment for cases and controls (yes or no or 0 star if no mention) | Non-response rate | |
| Wayse 2004 | 1 | 1 | 0 | 0 | 1 | 1 | 1 | 1 | 6 |
| Karatekin 2009 | 1 | 1 | 0 | 0 | 1 | 1 | 1 | 1 | 6 |
| Roth 2009 | 1 | 1 | 0 | 1 | 1 | 1 | 1 | 0 | 7 |
| Roth 2010 | 1 | 1 | 1 | 1 | 2 | 1 | 1 | 0 | 8 |
| Cetinkaya 2015 | 1 | 1 | 0 | 0 | 0 | 1 | 1 | 1 | 6 |
| Cizmeci 2015 | 1 | 1 | 0 | 0 | 2 | 1 | 1 | 1 | 7 |
| Narang 2016 | 1 | 1 | 0 | 0 | 1 | 1 | 1 | 1 | 6 |
| Dinlen 2016 | 1 | 1 | 0 | 0 | 1 | 1 | 1 | 1 | 6 |
| Ahmed 2015 | 1 | 1 | 0 | 1 | 2 | 1 | 1 | 1 | 8 |
| Cayir 2014 | 1 | 1 | 0 | 0 | 2 | 1 | 1 | 1 | 7 |
| Say 2017 | 1 | 1 | 0 | 0 | 2 | 1 | 1 | 0 | 6 |
| Basha 2014 | 1 | 1 | 0 | 1 | 2 | 1 | 1 | 0 | 7 |
| Jia 2017 | 1 | 1 | 0 | 1 | 1 | 1 | 1 | 1 | 7 |
| Jat 2016 | 1 | 1 | 0 | 1 | 2 | 1 | 1 | 1 | 8 |
| Moreno-Solis 2015 | 1 | 1 | 0 | 1 | 1 | 1 | 0 | 1 | 6 |
| Sakka 2014 | 1 | 1 | 0 | 1 | 2 | 1 | 1 | 1 | 8 |
| Khakshour 2015 | 1 | 1 | 0 | 0 | 1 | 1 | 1 | 0 | 5 |
| Li 2018 | 1 | 1 | 1 | 1 | 2 | 1 | 1 | 0 | 8 |

The Newcastle-Ottawa Scale (NOS) for assessing the quality of non-randomised studies in meta-analyses. Available at: http://www.ohri.ca/programs/clinical_epidemiology/oxford.asp.

For peer review only

**Additional Table 3C Newcastle Ottawa study quality scoring system (cross-sectional studies)**

| | SELECTION (Maximum 5 stars) | | | | COMPARABILITY (Maximum 2 stars) | OUTCOME (Maximum 3 stars) | | |
|---|---|---|---|---|---|---|---|---|
| Study | Representativeness of the sample | Sample size | Non-respondents | Ascertainment of the exposure (risk factor) | Subjects in different outcome groups are comparable, based on the study design or analysis. Confounding factors are controlled. | Assessment of the outcome (max=2 stars) | Statistical test (max=1 star) | Number of stars (out of 10 total) |
| Yaghmaie 2017 | 1 | 0 | 0 | 0 | 1 | 1 | 1 | 4 |
| Alvarez 2016 | 1 | 0 | 0 | 1 | 1 | 2 | 1 | 6 |
| Alonso 2015 | 1 | 0 | 0 | 1 | 1 | 1 | 1 | 5 |
| Binks 2014 | 1 | 0 | 0 | 1 | 1 | 2 | 1 | 6 |

The Newcastle-Ottawa Scale (NOS) for assessing the quality of non-randomised studies in meta-analyses. Available at: http://www.ohri.ca/programs/clinical_epidemiology/oxford.asp.

For peer review only

**Additional Table 4A Circulating 25(OH)D threshold levels used in the selected studies**

| Study | Number of cases | 25(OH)D categories (as given) | Number of cases in each category |
|---|---|---|---|
| Asilioglu 2017 | 250 | Deficiency: <20 ng/mL | 120 |
| | | Sufficiency: >=20 ng/mL | 85 |
| Halwany 2017 | 102 | Deficiency: <20 ng/ml | 38 |
| | | Insufficiency: 20-29.9 ng/ml | 39 |
| | | "Normal" levels: 30 ng/ml | 25 |
| Hurwitz 2017 | 90 | Deficiency: <20 ng/mL | 11 |
| Garcia-Soler 2017 | 340 | Deficiency: <20 ng/mL | 149 |
| | | Insufficiency: 20-30 ng/mL | 128 |
| | | 25(OH)D levels: 30-40 ng/mL | 41 |
| | | Optimal levels >40 ng/mL | 22 |
| Badawi 2017 | 88 | Deficiency: < 50nmol/l | 39 |
| | | Severe deficiency: <30 nmol/l | 30 |
| Jia 2017 | 110 | Severe deficiency: <10 ng/ml | 36 |
| | | Deficiency: 10-20 ng/ml | 56 |
| | | Insufficiency: 21-30 ng/ml | 17 |
| | | Sufficiency: >30 ng/ml | 1 |
| Yaghmaie 2017 | 82 | Deficiency: <30 ng/ml | 53 |
| | | Sufficiency: >=30 ng/ml | 29 |
| Say 2017 | 100 | Severe deficiency (group 1) <5 ng/mL. | 63 |
| | | Insufficiency (group2): 5 to 15 ng/mL | 24 |
| | | Sufficiency (group 3) ≥15 ng/mL | 13 |
| El-Gamasy 2017 | 80 | Serious deficiency: < 30 nmol/L | 26 |
| | | Insufficiency: 30-75 nmol/L | 27 |
| | | Adequate levels >75 nmol/L | 27 |
| Sankar 2017 | 43 | Severe deficiency: serum 25 (OH) D <10 ng/mL | 31 |
| Shah 2016 | 154 | 25(OH)D < 20 ng/mL | 128 |
| | | 25(OH)D ≥20 ng/mL | 26 |
| Ponnarmeni 2016 | 124 | Deficiency: < 50nmol/L | 63 |
| | | Insufficiency: 50-75 nmol/L | 31 |
| | | <75, insufficient and deficient | 94 |
| | | Sufficiency: >75 nmol/L | 30 |
| Sankar 2016 | 101 | Deficiency: ≤ 20 ng/mL | 75 |
| | | Severe deficiency: <15 ng/mL | 62 |
| | | 'No deficiency': > 20 ng/mL | 26 |
| Bustos 2016 | 90 | Deficiency: <20 ng/ml | 39 |
| | | Normal levels: >20 ng/ml | 51 |
| Ebenezer 2016 | 52 | Deficiency: < 20ng/mL | 21 |
| | | Insufficiency: 20–30 ng/ml | 12 |
| | | 'Normal levels: ≥ 20 ng/L | 31 |
| Elmoneim 2016 | 21 | Deficiency: < 20 ng/mL | 17 |
| | | Insufficiency: 20-30 ng/mL | 4 |
| | | Normal levels: > 30 ng/mL | 9 |
| Jat 2016 | 50 | Deficiency: <20 ng/ml | 42 |
| | | Insufficiency: 20-30 ng/ml | 2 |
| | | Sufficiency: >=30 ng/ml | 1 |
| Narang 2016 | 50 | "Severe deficiency": <20ng/ml | 28 |
| Dinlen 2016 | 30 | Deficiency: <=15 ng/mL | 26 |
| | | Severe deficiency: <=5 ng/mL | 3 |
| Lopez 2016 | 347 | 25-OHD levels: <10 ng/mL | 19 |
| | | 25-OHD levels: 10-20 ng/mL | 65 |
| | | 25-OHD levels: 20-30 ng/mL | 134 |
| Alvarez 2016 | 50 | 25(OH) D levels: <20 ng/mL | 29 |
| | | 25(OH) D levels: 20-30 ng/mL | 37 |
| Onwuneme 2015 (1) | 120 | 25(OH)D levels: <50nmol/L | 71 |
| | | 25(OH)D levels: ≥50nmol/L | 49 |
| Onwuneme 2015 (2) | 94 | 25(OH)D levels: <20 ng/mL | 86 |
| | | 25(OH)D levels: < 30 nmol/L | 60 |
| | | 25(OH)D levels: >=30 nmol/L | 34 |
| Prasad 2015 | 80 | Deficiency: < 20 ng/ml | 67 |
| | | Sufficiency: ≥ 20 ng/mL | 13 |
| Moreno-Solis 2015 | 48 | Deficiency: <20 ng/ml | 9 |
| | | Insufficiency: 21–29 ng/ml | 16 |
| | | Sufficiency: ≥30 ng/ml | 23 |



For peer review only - http://bmjopen.bmj.com/site/about/guidelines.xhtml

For peer review only

| | | | |
|---|---|---|---|
| Alonso 2015 | 288 | Deficiency: <20 ng/ml | 45 |
| | | <10 ng/ml | 6 |
| Korwutthikulrangsri 2015 | 32 | Deficiency: < 20 ng/ml | 25 |
| | | Insufficiency: 20-29.9 ng/ml | 7 |
| | | Sufficiency: ≥ 30 ng/mL | 0 |
| Khakshour 2015 | 37 | Deficiency: <20 ng/mL | 9 |
| Cizmeci 2015 | 40 | Deficiency: ≤20 ng/ml | 28 |
| | | Insufficiency: 21–29 ng/ml | 7 |
| | | Normal levels: ≥30 ng/ml | 5 |
| Cetinkaya 2015 | 50 | Severe deficiency: <10 ng $ml^{-1}$ | 42 |
| | | Insufficiency: 11 to 32 ng $ml^{-1}$ | 8 |
| Ayulo 2014 | 216 | Deficient: < 15 ng/ml | 61 |
| | | Insufficient: 15-29 ng/mL | 102 |
| | | Sufficient: ≥ 30 ng/mL | 53 |
| Dayal 2014 | 92 | Deficiency: < 50 nmol/L | 23 |
| | | Insufficiency: 50–75 nmol/L | 41 |
| | | Sufficiency: > 75 nmol/L | 28 |
| | | 25(OH) D levels: < 75 nmol/L | 64 |
| | | 'Non-deficiency': > 50 nmol/L | 69 |
| Hebbar 2014 | 61 | Deficiency: ≤ 10 ng/mL | 10 |
| | | Insufficiency: 10 to 20 ng/mL | 27 |
| | | Sufficiency: ≥ 20 ng/mL | 24 |
| Rey 2014 | 156 | Deficiency: < 20 ng/mL | 46 |
| | | 25(OH)D levels: ≥ 20 ng/mL | 110 |
| Ahmed 2014 | 50 | Deficiency: <50 nmol/L | 15 |
| Basha 2014 | 81 | Deficiency: <50 nmol/L | 53 |
| | | Insufficiency: <75 nmol/L | 14 |
| | | Normal level: >75 nmol/L | 14 |
| Sakka 2014 | 96 | Severe deficiency: 0–5 ng/mL | 29 |
| | | Deficiency: 5–15 ng/mL | 49 |
| | | Insufficiency: 15– 20 ng/mL | 11 |
| | | Sufficiency: 20–100 ng/mL | 7 |
| Cayir 2014 | 88 | Normal levels: 20 ng/mL | 38 |
| | | Insufficiency: 15-20ng/mL | 18 |
| | | Deficiency: <15ng/mL | 32 |
| Binks 2014 | 74 | 25(OH) D levels: < 50 nmol/L | 11 |
| Madden 2012 | 511 | 25(OH) D levels: < 10 ng/mL | 36 |
| | | 25(OH) D levels: 10-19.9 ng/mL | 169 |
| | | Deficiency: < 20 ng/ml | 205 |
| | | Insufficiency: <30 ng/mL | 364 |
| Rippel 2012 | 316 | Deficiency: < 50 nmol/L | 109 |
| | | 'Normal levels': ≥50 nmol/L | 207 |
| Madden 2012 | 511 | 25(OH)D levels: < 10 ng/mL | 36 |
| | | 25(OH)D levels: 10-19.9 ng/mL | 169 |
| | | Deficiency: < 20 ng/mL | 205 |
| | | Insufficiency: <30 ng/mL | 364 |
| McNally 2012 | 326 | Deficiency: < 50 nmol/L | 225 |
| | | 25(OH)D levels: 50 to 75 nmol/L | 75 |
| | | 'Not deficient': > 50 nmol/L | 101 |
| Inamo 2011 | 28 | 25(OH)D levels: < 10 ng/mL | 4 |
| | | 25(OH)D levels: < 15 ng/mL | 8 |
| | | 25(OH)D levels: < 25 ng/mL | 12 |
| | | 25(OH)D levels: < 40 ng/mL | 28 |
| Roth 2010 | 25 | 25(OH)D levels: < 40 nmol/L | 21 |
| Banajeh 2009 | 79 | Deficiency: <30 nmol/L | 29 |
| Karatekin 2009 | 25 | Serum 25(OH)D <10 (deficiency) | 19 |
| | | Serum 25(OH)D 11 to 20 (deficiency) | 4 |
| | | Serum 25(OH)D 21 to 32 (insufficiency) | 1 |
| | | Serum 25(OH)D 32 to 100 (sufficiency) | 1 |
| Roth 2009 | 64 | < 40 nmol/L | 3 |
| Wayse 2004 | 80 | Plasma 25(OH)D3 > 22.5 nmol/L | 26 |
| | | Plasma 25(OH)D3 > 50 nmol/L | 4 |
| Mathias 2017 | 41 | 25(OH)D < 20 ng/ml | 28 |



For peer review only - http://bmjopen.bmj.com/site/about/guidelines.xhtml

For peer review only

| | | | |
|---|---|---|---|
| | | 25(OH)D < 30 ng/ml | 36 |
| Dhandai 2018 | 60 | Deficiency: < 20 ng/ml | 38 |
| | | Insufficiency: <29 ng/ml | 17 |
| | | Optimum: 30-50 ng/ml | 5 |
| Vo 2018 | 1016 | 25(OH)D < 20 ng/ml | 298 |
| | | 20-29.9 ng/ml | 352 |
| | | >=30 ng/ml | 366 |
| Li 2018 | 797 | Severe deficiency: < 10 ng/mL | 159 |
| | | Deficiency: 10–20 ng/mL | 286 |
| | | Insufficiency: 20– 30 ng/mL | 223 |
| | | Sufficiency: > 30 ng/mL | 127 |

25(OH)D in nmol/L = 25(OH)D in ng/mL multiplied by 2.496

For peer review only

**Additional Table 4B Circulating 25(OH)D threshold levels used in the selected studies for prevalence in sepsis**

| Study | Number of cases | 25(OH)D categories (as provided by each study) | Number of cases |
|---|---|---|---|
| Asilioglu 2017 | 30 sepsis | Deficiency: <20 ng/mL | 20 |
| | | Sufficiency: >=20 ng/mL | 10 |
| Say 2017 | 100 neonatal sepsis | Severe deficiency (group 1) <5 ng/mL. | 63 |
| | | Insufficiency (group2): 5 to 15 ng/mL | 24 |
| | | Sufficiency (group 3) ≥15 ng/mL | 13 |
| El-Gamasy 2017 | 46 sepsis | Serious deficiency: < 30 nmol/L | 20 |
| | | Insufficiency: 30-75 nmol/L | 18 |
| | | Adequate levels >75 nmol/L | 8 |
| Sankar 2017 | 43 septic shock | Severe deficiency: serum 25 (OH) D <10 ng/mL | 31 |
| Shah 2016 | 100 sepsis | 25(OH)D <20 ng/mL | 84 |
| | | 25(OH)D >=20 ng/mL | 26 |
| Ponnarmeni 2016 | 124 sepsis | Deficiency: <50nmol/L | 63 |
| | | Insufficiency: 50-75 nmol/L | 31 |
| | | <75, insufficient + deficient | 94 |
| | | Sufficiency: >75 nmol/L | 30 |
| Bustos 2016 | 10 sepsis intraabdominal | Deficiency: < 20 ng/ml | 7 |
| | | Normal levels: > 20 ng/mL | 3 |
| Ebenezer 2016 | 16 shock | 25(OH)D <20 ng/mL | 8 |
| | | 25(OH)D >=20 ng/mL | 8 |
| Onwuneme 2015 (1) | 35 culture positive sepsis | 25(OH)D <50 ng/mL | 32 |
| | | 25(OH)D >=50 ng/mL | 3 |
| Onwuneme 2015 (2) | 46 culture positive sepsis and late-onset sepsis | Deficiency: < 30 nmol/L | 32 |
| | | Sufficiency: ≥ 30 nmol/L | 14 |
| Prasad 2015 | 11 positive blood culture | 25(OH)D <20 ng/mL | 9 |
| | | 25(OH)D >=20 ng/mL | 2 |
| Korwutthikulrangsri 2015 | 17 shock and septicaemia | 25(OH)D <20 ng/mL | 14 |
| | | 25(OH)D >=20 ng/mL | 3 |
| Cizmez 2015 | 40 suspected sepsis | Deficiency: ≤20 ng/ml | 28 |
| | | Insufficiency: 21–29 ng/ml | 7 |
| | | Normal levels: ≥30 ng/ml | 5 |
| Dayal 2014 | 9 nosocomial sepsis | 25(OH)D <20 ng/mL | 4 |
| | | 25(OH)D >=20 ng/mL | 5 |
| Hebbar 2014 | 30 shock and/or Sepsis | 25(OH)D <20 ng/mL | 17 |
| | | 25(OH)D >=20 ng/mL | 13 |
| McNally2012 | 48 septic | 25(OH) D levels: <50 nmol/L | 33 |
| | | 25(OH) D levels: ≥50 nmol/L | 15 |
| Mathias 2017 | 41 sepsis, severe sepsis or septic shock | 25(OH)D < 20 ng/ml | 28 |
| | | 25(OH)D < 30 ng/ml | 36 |
| Dhandai 2018 | 60 sepsis | Deficiency: < 20 ng/ml | 38 |
| | | Insufficiency: <29 ng/ml | 17 |
| | | Optimum: 30-50 ng/ml | 5 |



For peer review only - http://bmjopen.bmj.com/site/about/guidelines.xhtml

**Additional Table 5 Studies with thresholds other than < 50 nmol/L**

| Study | Threshold used by study |
|---|---|
| Roth 2009 | < 40 nmol/L |
| Roth 2010 | < 40 nmol/L |
| Say 2017 | <=15 ng/mL (37.4 nmol/L) |
| Inamo 2011 | <=15 ng/mL (37.4 nmol/L) |
| Ayulo 2014 | <=15 ng/mL (37.4 nmol/L) |
| Dinlen 2016 | <=15 ng/mL (37.4 nmol/L) |
| Onwuneme 2015 (2) | < 30 nmol/L |
| Yaghmaie 2017 | < 30 ng/mL (74.88 nmol/L) |
| El-Gamasy 2017 | < 30 nmol/L |
| Banajeh 2009 | < 30 nmol/L |
| Sankar 2017 | <= 10 ng/mL (24.9 nmol/L) |
| Cetinkaya 2015 | <= 10 ng/mL (24.9 nmol/L) |

25(OH) D values nmol/L = ng/mL * 2.496

For peer review only



For peer review only - http://bmjopen.bmj.com/site/about/guidelines.xhtml

**Additional Table 6 Excluded studies**

| Study | Design | Sample size | Characteristics of pediatric population | Country | Reasons for exclusion of paper |
|---|---|---|---|---|---|
| Seliem 2016 | Hospital-based case-control | Cases, n= 30<br>Controls, n= 30 | Cases: group 1, full-term neonates with EOS and their mothers.<br>Controls, group 2: 30 full-term healthy neonates and their mothers with no clinical or laboratory evidence of sepsis. | Egypt | Requested data without reply or data not available |
| Gamal 2017 | Case-control | Cases, n= 50<br>Control, n= 30 | Cases: neonates with early onset neonatal sepsis that occurred at <72 hours<br>Controls: Age and sex healthy neonates with no prenatal risk factor for early neonatal sepsis enrolled in the study as a control group. | Egypt | Requested data without reply or data not available |
| Aydemir 2014 | Hospital-based case control | Cases, n=40<br>Controls, n= 20 | Cases: children with sepsis between 1 and 16 years old<br>Controls: children without sepsis | Turkey | Requested data without reply or data not available |
| Garg 2016 | Hospital-based case-control | Cases, n= 40<br>Controls, n= 40 | Cases: children from 6 months to 5 years of age admitted or attending OPD in department of Pediatrics.<br>Controls: children receiving care at the Hospital's ambulatory, emergency or in-patient units and presenting with non-respiratory complaints also not having any clinical indication of vitamin D deficiency. | India | Requested data without reply or data not available |

For peer review only



For peer review only - http://bmjopen.bmj.com/site/about/guidelines.xhtml

**Additional Table 7 Characteristics of the 52 included studies**

| Study | Design | Number of cases (controls, where appropriate) | Characteristics of population | Country and setting | Vitamin D thresholds as defined by the study | Quality score (NOS) |
|---|---|---|---|---|---|---|
| Asilioglu 2017 | Historical cohort (single centre) | 250 | Cases: aged 1 month to ≤18 years | Turkey, PICU | Deficiency: <20 ng/mL | 7 |
| Halwany 2017 | Cohort (single centre) | 102 | Cases: children aged >1 month to ≤ 5 years | Egypt, PICU | Deficiency: <20 ng/ml<br>Insufficiency: 20-29.9 ng/ml<br>"Normal" levels: 30 ng/ml | 6 |
| Hurwitz 2017 | Prospective cohort (single centre) | 90 | Cases: aged <5 years hospitalized with LRTI and RSV and/or hMPV | USA, PICU | Deficiency: <20 ng/mL | 6 |
| Garcia-Soler 2017 | Cohort (single centre) | 340 | Cases: aged 6 months to 17 years<br>Critically ill with various conditions | Spain, PICU | Deficient: <20 ng/mL<br>Insufficiency: 20-30 ng/mL<br>25(OH)D levels: 30-40 ng/mL<br>Optimal levels >40 ng/mL | 7 |
| Badawi 2017 | Cohort (single centre) | 88 | Cases: 1 month to 12 years | Egypt, PICU | Deficiency: < 50nmol/l,<br>Severe deficiency: <30 nmol/l | 8 |
| Jia 2017 | Hospital-based case-control (single centre) | 110 (110) | Cases: infants (< 1 year of age) with ALRTI<br>Controls: Healthy from similar areas attending the hospital for vaccination during study period | China, PICU | Severe deficiency: <10 ng/ml<br>Deficiency: 10-20 ng/ml<br>Insufficiency: 21-30 ng/ml<br>Sufficiency: >30 ng/ml | 7 |
| Yaghmaie 2017 | Cross sectional (single centre) | 82 | Cases: hospitalized in PICU | Iran, PICU | Deficiency: <30 ng/ml | 4 |
| Say 2017 | Case-control (single centre) | 100 (13) | Cases: premature infants less than 37 weeks diagnosed with early or late -onset neonatal sepsis<br>Controls: From same population of neonates with sepsis but not vitamin D deficient | Turkey, NICU | Severe deficiency (group 1) <5 ng/mL.<br>Insufficiency (group2): 5 to 15 ng/mL<br>Sufficiency (group 3) ≥15 ng/mL | 6 |



For peer review only - http://bmjopen.bmj.com/site/about/guidelines.xhtml

| | | | | | | |
|---|---|---|---|---|---|---|
| El-Gamasy 2017 | Cohort (single centre) | 80 (20) | Cases: 3 months to 12 years hospitalized with acute kidney injury<br>Controls: completely healthy subjects | Egypt, PICU | Seriously deficient: < 30 nmol/L<br>Insufficient: 30-75 nmol/L<br>Adequate >75 nmol/L | 7 |
| Sankar 2017 | Cohort (single centre) | 43 | Cases: <=17 years of age | India, PICU | Severe vitamin D deficiency: serum 25 (OH) D <10 ng/mL | 8 |
| Shah 2016 | Cohort (single centre) | 154 | Cases: aged between 1 month and 15 years | India, PICU | Deficiency: <20 µg/mL | 6 |
| Ponnarmeni 2016 | Cohort (single centre) | 124 (338) | Cases: aged 1–12 years admitted with a diagnosis of sepsis<br>Controls: from previous prospective study, apparently healthy children of upper socioeconomic status who attended the out-patient department for immunization or with minor ailments | India, PICU | Deficiency: < 50 nmol/L<br>Insufficiency: 50-75 nmol/L<br>Sufficiency: > 75 nmol/L | 6 |
| Sankar 2016 | Cohort (single centre) | 101 | Cases: aged 1 month to 17 years | India, PICU | Deficiency: ≤ 20 ng/ml<br>Severe deficiency: <15 ng/mL | 8 |
| Bustos 2016 | Cohort (single centre) | 90 | Cases: critically ill, greater than 37 weeks and less than 15 years of age | Chile, PICU | Deficiency: < 20 ng/ml<br>Normal levels: > 20 ng/mL | 7 |
| Ebenezer 2016 | Cohort (single centre) | 52 | Cases: <18 years of age; medical and surgical diagnoses | India, PICU | Deficiency: < 20 ng/ml<br>Insufficiency: 20–30 ng/ml<br>Normal levels: ≥ 20 ng/L | 6 |
| Elmoneim 2016 | Cohort (single centre) | 30 | Cases: aged less than 14 years<br>Controls: with “normal” vitamin D levels | Saudi Arabia, PICU | Deficiency: < 20ng/ml<br>Insufficiency: 20-30 ng/mL<br>Normal: > 30 ng/mL | 7 |
| Jat 2016 | Hospital-based case-control (single centre) | 50 (50) | Cases: 1 month to 12 years of age admitted with pneumonia<br>Controls: admitted for reasons other than respiratory symptoms, required blood sampling | India, PICU | Deficiency: < 20 ng/ml<br>Insufficiency: 20-30 ng/ml Sufficiency: >= 30 ng/ml | 8 |
| Narang 2016 | Hospital-based case-control (single centre) | 50 (50) | Cases: 2 months to 5 years of age admitted as in-patients or seen in the outpatient department with ALRI<br>Controls: healthy, same age group, attending outpatients’ service for immunization or admitted for minor conditions other than ALRI | India, PICU | Severe deficiency: < 20ng/ml | 6 |



For peer review only - http://bmjopen.bmj.com/site/about/guidelines.xhtml

| | | | | | | |
|---|---|---|---|---|---|---|
| Dinlen 2016 | Hospital-based case-control (single centre) | 30 (30) | Cases: term neonates with ALRI<br>Controls: healthy neonates, same age as the study group. | Turkey, NICU | Deficient: <= 15 ng/mL<br>Severe deficiency: <= 5 ng/mL | 6 |
| Lopez 2016 | Prospective cohort (multi centre) | 347 | Cases: 0 to >48 months admitted to hospital with ALRI prospectively recruited through the GENDRES (GENetic,vitamin D and RESpiratory infections research network) | Spain, PICU | 25-OHD levels: <10 ng/mL<br>25-OHD levels: 10-20 ng/mL<br>25-OHD levels: 20-30 ng/mL | 8 |
| Alvarez 2016 | Cross-sectional (single centre) | 50 | Cases: patients aged 0 to 18 years | USA, PICU | 25(OH)D < 20 ng/mL<br>25(OH)D < 30 ng/mL | 6 |
| Onwuneme 2015 (1) | Cohort (single centre) | 120 (30) | Cases: with suspected sepsis (<12 years old)<br>Controls: paediatric controls admitted for elective day case surgery during the same study period and were not suspected of having sepsis | Ireland , PICU | 25(OH) D levels: <50 nmol/L<br>25(OH) D levels: ≥50 nmol/L | 8 |
| Onwuneme 2015 (2) | Cohort (single centre) | 94 | Cases: preterm infants <32 weeks gestation | Ireland, NICU | Deficiency: < 30 nmol/L<br>Sufficiency: ≥ 30 nmol/L | 8 |
| Prasad 2015 | Cohort (single centre) | 80 | Cases: 2 months to 12 years old | India, PICU | Deficiency: < 20 ng/ml<br>Sufficient: ≥ 20 ng/mL | 7 |
| Moreno-Solis 2015 | Hospital-based case-control (single centre) | 48 (30) | Cases: aged 1–11 months with acute bronchiolitis<br>Controls: healthy, <12 months, admitted to the outpatient clinic without respiratory symptoms or history of hospitalization for bronchiolitis or wheezing | Spain, PICU | Deficiency: < 20 ng/ml<br>Insufficiency: 21–29 ng/ml Sufficiency: ≥ 30 ng/ml | 6 |
| Alonso 2015 | Cross sectional (single centre) | 288 | Cases: aged 1 month to 13 years | Spain, PICU | Deficient: < 20 ng/ml and < 10 ng/ml | 5 |
| Korwutthikulrangsri 2015 | Cohort (single centre) | 32 (36) | Cases: requiring PICU admission<br>Controls: Healthy, enrolled during the same period of time and served as the control group (age in months) | Thailand, PICU | Deficiency: < 20 ng/mLl<br>Insufficiency: 20-29.9 ng/ml<br>Sufficiency: ≥ 30 ng/mL | 7 |
| Khakshour 2015 | Case-control | 37 (53) | Cases: below 5 years of age and suffering from respiratory infections<br>Controls: those who were not suffering from respiratory infections | Iran, PICU | Deficiency: < 20 ng/mL | 5 |



For peer review only - http://bmjopen.bmj.com/site/about/guidelines.xhtml

| | | | | | | |
|---|---|---|---|---|---|---|
| Cizmeci 2015 | Case-control (single centre) | 40 (43) | Cases: infants with suspected early-onset neonatal sepsis.<br>Controls: For each newborn of group 1, one healthy infant selected as a control | Turkey, NICU | Deficiency: ≤ 20 ng/ml<br>Insufficiency: 21–29 ng/ml<br>Normal levels: ≥ 30 ng/ml | 7 |
| Cetinkaya 2015 | Hospital-based case-control (single centre) | 50 (50) | Cases: term infants with clinically suspected (probable) early infection (early-onset sepsis) within the first 3 postnatal days of life and were >37 weeks of gestational age<br>Controls: healthy infants with no signs of clinical/laboratory infection | Turkey, NICU | Severe deficiency: <10 ng ml$^{-1}$<br>Insufficiency: 11 to 32 ng ml$^{-1}$<br>Adequacy: 32 to 100 ng ml$^{-1}$ | 6 |
| Ayulo 2014 | Cohort (single centre) | 216 | Cases: between the ages of 1 and 21 years | Spain, PICU | Deficient: < 15 ng/ml<br>Insufficient: 15-29 ng/mL<br>Sufficient: ≥ 30 ng/mL | 7 |
| Dayal 2014 | Cohort (single centre) | 92 | Cases: Children aged 3 months to 12 years | India, PICU | Deficiency: < 50 nmol/L<br>Insufficiency: 50–75 nmol/L<br>Sufficiency: > 75 nmol/L<br>25(OH) D levels: < 75 nmol/L | 6 |
| Hebbar 2014 | Cohort (single centre) | 61 (46) | Cases: children 0 to 18 years<br>Controls: patients recruited among children in the magnetic resonance imaging suite. | USA, PICU | Deficient: ≤ 10 ng/ml<br>Insufficient: 10 to 20 ng/ml<br>Sufficient: ≥ 20 ng/mL | 6 |
| Rey 2014 | Cohort (single centre) | 156 (289) | Cases: heterogeneous group of critically ill children aged <16 years<br>Control group for comparison: population of healthy children | Spain, PICU | Deficient: < 20 ng/ml | 7 |
| Ahmed 2014 | Hospital-based case-control (single centre) | 50 (50) | Cases: aged 2–60 months hospitalized with ALRI<br>Controls: age-matched with cases within 1 or 2 months, attending well-child clinics or general clinics without evidence of respiratory infection or admitted to the hospital for elective surgery | Africa, PICU | Deficiency: <50 nmol/L | 8 |
| Basha 2014 | Hospital-based case-control (single centre) | 81 (89) | Cases: under 5 years old with severe pneumonia selected from the inpatient departments and emergency units of the hospital<br>Controls: healthy, selected from the outpatient surgical clinics of the hospital during their visit for umbilical or inguinal hernia repair and not suffering | Egypt, PICU | Deficiency: < 50 nmol/L<br>Insufficiency: < 75 nmol/L<br>Sufficiency: > 75 nmol/L | 7 |



For peer review only - http://bmjopen.bmj.com/site/about/guidelines.xhtml

| | | | | | | |
|---|---|---|---|---|---|---|
| | | | from upper or lower respiratory infections | | | |
| Sakka 2014 | Population based case-control (single centre) | 96 (96) | Cases: <2 years old with ALRI, 48 diagnosed with pneumonia and 48 with bronchiolitis<br>Controls: age and sex matched with no respiratory symptoms or signs from the Health office | Egypt, PICU | Severe deficiency: 0–5 ng/mL Deficiency: 5–15 ng/mL Insufficiency: 15– 20 ng/mL Sufficiency: 20–100 ng/mL | 8 |
| Cayir 2014 | Hospital-based case-control (single centre) | 88 (81) | Cases: 1 to 13 years diagnosed with acute otitis media<br>Controls: Healthy same age range | Turkey, PICU | Normal levels:  20 ng/mL<br>Insufficiency: 15-20ng/mL<br>Deficiency: <15ng/mL | 7 |
| Binks 2014 | Cross-sectional | 74 | Cases: aged <3 years admitted with acute lower respiratory infections (ALRIs) or other conditions | Australia, PICU | 25(OH) D levels: < 50 nmol/L | 6 |
| Madden 2012 | Cohort (single centre) | 511 | Cases: less than 21 years old | USA, PICU | 25(OH) D levels: < 10 ng/mL<br>25(OH) D levels: 10-19.9 ng/mL<br>Deficiency: < 20 ng/ml<br>Insufficiency: < 30 ng/mL | 8 |
| Rippel 2012 | Cohort (single centre) | 316 | Cases: children aged 16.5 (3.1–75.2) months | Australia, PICU | Deficiency: < 50 nmol/L<br>"Normal" levels: ≥ 50 nmol/L | 8 |
| McNally 2012 | Cohort (multi-centre) | 326 | Cases: Newborn to 17 years of age | Canada, PICUs | Deficiency: < 50 nmol/L<br>25(OH) D levels:<br>50 to 75 nmol/L | 7 |
| Inamo 2011 | Cohort (single centre) | 28 | Cases: between 1 and 48 months hospitalized with ALRI, 26 diagnosed with bronchiolitis and two as having pneumonia | Japan, PICU | Deficiency: ≤ 15ng/ml<br>Severe deficiency:<br>≤ 5ng/mL | 6 |
| Roth 2010 | Hospital-based case-control (single centre) | 25 (25) | Cases: 1–18 months hospitalized with ALRI<br>Controls: selected by population-based sampling. aged 1–23 months, and matched to cases on age (±2 months) and sex | Bangladesh, PICU | Deficiency: < 40 nmol/L | 8 |
| Banajeh 2009 | Prospective cohort (single centre) | 79 | Cases: 2–59 months with WHO-defined very severe community acquired (VSP) pneumonia | Iran, PICU | Deficiency: < 30 nmol/L | 7 |
| Karatekin 2009 | Hospital-based case- | 25 (15) | Cases: newborns with acute respiratory infections | Turkey, NICU | Deficiency: < 20 ng/ml | 6 |



For peer review only - http://bmjopen.bmj.com/site/about/guidelines.xhtml

| | | | | | | |
|---|---|---|---|---|---|---|
| | control, (single centre) | | Controls: healthy, age matched from outpatients' service where they went for immunization. | | | |
| Roth 2009 | Hospital-based case-control (single centre) | 64 (65) | Cases: aged 1-25 months admitted with ALRI<br>Controls: aged 1-25 months undergoing elective surgery, no history of hospitalization for ALRI | Canada, PICU | Deficiency: < 40 nmol/L | 7 |
| Wayse 2004 | Hospital-based case-control, (single centre) | 80 (70) | Cases: < 5 years with severe ALRI<br>Controls: healthy, attending outpatients service for immunization | India, PICU | Deficiency: Plasma 25(OH)D3 < 50 nmol/L | 6 |
| Mathias 2017 | Prospective cohort (single centre) | 41 | Cases: less than 18 years admitted with diagnosis of sepsis, severe sepsis or septic shock. | USA, PICU | 25(OH)D levels < 20 ng/ml<br>25(OH)D levels <30 ng/ml | 6 |
| Dhandai 2018 | Cohort (multi-centre) | 60 (60) | Cases: neonates admitted with late-onset sepsis (LOS)<br>Controls: neonates admitted during same period with clinically significant physiological hyperbilirubinaemia (without sepsis) | India, NICU | Deficiency: < 20 ng/ml<br>Insufficiency: <29 ng/ml<br>Optimum: 30-50 ng/ml | 7 |
| Vo 2018 | Prospective cohort (multi-centre) | 1016 | Cases: Infants less than twelve months old hospitalized for bronchiolitis | USA, PICU | 25(OH)D < 20 ng/ml<br>25(OH)D: 20-29.9 ng/ml<br>25(OH)D >= 30 ng/ml | 8 |
| Li 2018 | Case-control (single-centre) | 797 (785) | Cases: children with pneumonia or pneumonia-induced sepsis group | China, PICU | Severe deficiency: < 10 ng/mL<br>Deficiency: 10–20 ng/mL<br>Insufficiency: 20– 30 ng/mL<br>Sufficiency: > 30 ng/mL | 8 |

LRTI = lower respiratory tract infection; PICU = paediatric intensive care unit; RSV = respiratory syncytial virus; ALRI = acute lower respiratory infection; WHO = World Health Organization; VSP = very severe community acquired pneumonia; NICU = neonatal intensive care unit.



For peer review only - http://bmjopen.bmj.com/site/about/guidelines.xhtml

**Additional Table 8 Objectives and outcomes of included studies**

| Study | Objectives/aims of study | Main outcome(s) and conclusion(s) |
|---|---|---|
| Madden 2012 | Prevalence of vitamin D deficiency in critically ill children and factors influencing admission 25-hydroxyvitamin D (25(OH)D) levels | Identified high prevalence of vitamin D deficiency and insufficiency in critically ill children. Inverse association severity of illness on admission and 25(OH) levels |
| Lopez 2016 | Role of Vitamin D in Children hospitalized with Lower Tract Acute Respiratory Infections | 25-hydroxyvitamin D levels of study population below normal range. Correlation of higher disease severity with lower levels of 25-hydroxyvitamin D. |
| Garcia-Soler 2017 | Prevalence and risks factors of vitamin D deficiency, as well as its relationship with length of PICU stay morbidity and mortality in a PICU. | Vitamin D deficiency is frequent in paediatric critical patients. It also has an association with higher severity scores, season of year and parental educational attainment.<br>Vitamin D levels associated with various laboratory parameters of SIRS. Vitamin D deficiency associated with increased risk of morbidity and mortality. Inconclusive findings on its association with PICU length of stay were inconclusive |
| McNally 2012 | Prevalence of vitamin D deficiency, risk factors and potential association with clinically relevant outcomes in critically ill children in Canada. | Most of critically ill children vitamin D deficient at PICU admission. Lower 25(OH) D levels associated with increased catecholamine requirements, fluid bolus administration, hypocalcemia, and longer PICU admission. |
| Rippel 2012 | Prevalence of hypovitaminosis D and association with outcome in critically ill children requiring admitted in intensive care. | Hypovitaminosis D is frequent in critically ill children in PICU, especially in infants and children with heart disease. Hypovitaminosis D associated with hypocalcemia in non-cardiac population, and increased need for calcium replacement in the cardiac population.<br>No association between vitamin D status and survival or PICU length of stay. Strong association with early postoperative inotropic needs in the cardiac population. |
| Alonso 2015 | Investigate relationship of serum 25-hydroxyvitamin D concentrations with serum parathyroid hormone (PTH) levels, body mass index (BMI), and environmental factors in a population of Caucasian children living at latitude 43°N. | Results doubt the assumption that a serum 25OH D threshold indicates vitamin D deficiency in children. |
| Asilioglu 2017 | Measure occurrence of VDD in critically ill children. Assess determinants of vitamin D status and compare vitamin D deficient and sufficient cases in respect of severity of illness. | Hypovitaminosis D occurrence high in critically ill children and associated with higher vasopressor requirement. Not associated with other markers of illness severity including mortality. |
| Ayulo 2014 | Prevalence of vitamin D deficiency among children in PICU | Vitamin D deficiency common. No significant correlation between disease severity and vitamin D levels levels of vitamin D. Mortality associated with vitamin D levels |
| Rey 2014 | Identify prevalence of 25 hydroxivitamin D or 25(OH) vitamin D deficiency on pediatric intensive care unit (PICU) admission, and if associated with increased prediction of mortality risk scores. | Hypovitaminosis D incidence high in PICU patients. Hypovitaminosis D not associated with higher prediction of risk mortality scores. |
| Shah 2016 | Determine prevalence of vitamin D deficiency in critically ill children its association with illness severity, parathyroid response and clinical outcomes. | High prevalence of vitamin D deficiency. Parathyroid gland response secondary vitamin D deficiency or hypocalcemia impaired in critically ill. |
| Ponnarmeni 2016 | Vitamin D deficiency in critically ill children with sepsis admitted to PICU and its association with: mortality, length of stay, illness severity, requirement for ventilation and catecholamines | High prevalence of vitamin D deficiency<br>No significant association between vitamin D deficiency and other outcomes such as mortality |
| Onwuneme 2015 (1) | Assess vitamin D status, and its determinants, in chidren with suspected sepsis admitted to PICU. Also investigated association between vitamin D status and clinical outcomes. | Children admitted to the PICU with suspected sepsis lower 25OH D compared to controls. Inadequate 25 OH D levels associated with confirmed sepsis and poor outcomes. |
| Jia 2017 | Association of vitamin D with ALRTI in Chinese infants | Lowered plasma level of 25-OH Vitamin D makes children susceptible to ALRTI. |
| Halwany 2017 | Frequency of vitamin D deficiency in critically ill pediatric intensive care unit [PICU] patients and relation to state of serum 25(OH) D to disease severity. | High prevalence of vitamin D deficiency in critically ill children.<br>Negative correlation of Vitamin D level with PELOD score. Recommend screening of critically ill children for vitamin D deficiency to restore their serum levels. |



For peer review only - http://bmjopen.bmj.com/site/about/guidelines.xhtml

| | | |
|---|---|---|
| Sankar 2016 | Prevalence of vitamin D deficiency on admission and examine association with length of ICU stay | High prevalence of vitamin D deficiency. Vitamin D deficient children with longer ICU stay duration or mortality |
| Say 2017 | Evaluate effects of low vitamin D levels in cord blood on neonatal sepsis in preterm infants. | No significant relationship between the cord blood vitamin D levels and the risk of neonatal sepsis in premature infants. |
| Sakka 2014 | Determine the relation between vitamin D deficiency, anemia and the severity of ALRTIs in hospitalized children. | Vitamin D deficiency, low BMI, low hemoglobin level, rachitic signs were risk factors for the severity of ALRTIs. |
| Onwuneme2015 (2) | Investigate the association between serum 25-hydroxyvitamin D (25OHD) levels and outcomes in preterm infants (<32 weeks gestation). | High prevalence of low 25OHD. Association between vitamin D status and acute respiratory morbidity in preterm infants after birth. In none of the following outcomes was the difference statistically significant: surfactant use, inotrope requirement, RDS, pneumothorax, pulmonary hemorrhage, chronic lung disease, sepsis, retinopathy of prematurity, intraventricular hemorrhage, periventricular leucomalacia, patent ductus arteriosus, and mortality |
| Dayal 2014 | Prevalence of vitamin D deficiency<br>Association of serum vitamin D levels with duration of stay in hospital, mortality and requirement of ventilation | Reduced serum vitamin D levels in children |
| Bustos 2016 | Determine prevalence of vitamin D deficiency and its association with other clinically relevant outcomes in children admitted to Paediatric Intensive Care Unit | Vitamin D deficiency was prevalent in critically ill children and associated with adverse clinical outcomes. |
| Hurwitz 2017 | Measure retinol binding protein and vitamin D in children aged <5 years hospitalized with lower respiratory tract infection and respiratory syncytial virus and/or human meta pneumovirus detections | Low vitamin levels in 50% of the children and associated with significantly elevated risk of the need for intensive care unit admission and invasive mechanical ventilation. |
| Cayir 2014 | Investigate the relationship between Vitamin D deficiency and acute otitis media infection | Serum 25-hydroxy vitamin D levels significantly lower in children with acute otitis media compared to the controls. Vitamin D deficiency plays a role in otitis media infection. |
| Badawi 2017 | Investigated if VDD is related to higher severity scores and organ dysfunction. Primary objective of study was to estimate the prevalence of VDD in a group of critically ill children, and secondary objectives was to correlate vitamin D status with pediatric logistic organ dysfunction (PELOD) and pediatric risk ofmortality III (PRISM III) scores. | VDD prevalence was reported in about half of the critically ill patients, and it was observed to be related to multiple organ dysfunctions and rapid clinical deterioration. |
| Yaghmaie 2017 | Investigate relation of vitamin D deficiency with potential demographic and clinical factors. | Vitamin D deficiency among paediatric intensive care unit patients similar to western countries, also with similar age and BMI distribution. Significant relation observed between age and serum level of vitamin D. |
| Basha 2014 | Aimed to evaluate vitamin D status as a risk factor for severe pneumonia in Egyptian hospitalized children under 5 years | Significant association between vitamin D deficiency and severe pneumonia in Egyptian children below 5 years |
| Prasad 2015 | Prevalence of vitamin D deficiency in critically ill children and association with illness severity& other outcomes | High prevalence of vitamin D deficiency in critically ill children and association with PRISM III scores in a developing country.<br>Groups did not differ in terms of PICU stay, duration of hospital stay, culture positivity, biochemical parameters (serum calcium, serum phosphate), need of ventilation or steroids, presence of coagulopathy and mortality. |
| Wayse 2004 | If vitamin D deficiency in Indian children under 5 years old of age is risk factor for severe acute lower respiratory infection (ALRI). | Subclinical vitamin D deficiency & nonexclusive breastfeeding in four first months of life found to be significant risk factors for severe ALRI in Indian children. |
| El-Gamasy 2017 | Assess serum 25 (OH)D level in critically ill paediatric patients with AKI at PED of Tanta University Emergency Hospital (TUEH) within the first 24 hours of admission and evaluate its correlation with duration of hospital stay and mortality outcome. | Vitamin D deficiency associated with higher incidence of sepsis and mortality. |



For peer review only - http://bmjopen.bmj.com/site/about/guidelines.xhtml

| | | |
|---|---|---|
| Banajeh 2009 | Determine if rickets and VDD predict the outcomes in very severe pneumonia (VSP). | In WHO-defined VSP, nutritional rickets was strongly associated with a reduced successful treatment outcome, and VDD was a significant and independent predictor of reduced circulating PMNs and persistent hypoxemia. |
| Binks 2014 | Aimed to determine prevalence of vitamin D insufficiency among children hospitalised with ALRI in the Northern Territory. | Vitamin D insufficiency was observed in about one-third of these hospitalised children. Children hospitalised with an ALRI less likely to have vitamin D insufficiency compared with children hospitalised for other conditions (predominantly gastroenteritis). |
| Roth 2009 | Test the hypothesis that vitamin D status is associated with the risk of ALRI in Canadian children (1 month to 2 years old) | Among children aged 1 month to 2 years, vitamin D status not associated with ALRI requiring hospitalization |
| Hebbar 2014 | Prevalence of vitamin D insufficiency and relationship between vitamin D levels infection and innate immunity | High prevalence of vitamin D deficiency<br>Serum vitamin D levels - no correlation with illness severity score |
| Ebenezer 2016 | Determine vitamin D status and association with outcomes | Vitamin D deficiency common among pediatric patients<br>No association between vitamin D status and admission and mortality such as higher mortality and/or longer PICU stay<br>Deficiency associated with mechanical ventilation severity of illness, vasopressor need |
| Narang 2016 | Identify an effective nutritional agent that reduces the need for antibiotics, duration of pneumonia and length of hospitalization would be highly cost-beneficial. | Severe vitamin D deficiency (<20 ng/ml), nonexclusive breastfeeding in the first six months of life, inadequate sun exposure found to be significant risk factors for ALRTI in Indian children. |
| Alvarez 2016 | Study aimed to investigate relationship between vitamin D status and plasma markers of lutathione (GSH) and cysteine (Cys) redox and immunity in critically ill children | Vitamin D sufficiency was associated with more reduced plasma hCySS, indicative of lower oxidative stress, in critically ill children. Plasma GSH, GSSG, and glutamine, however, were lower in the vitamin D sufficient group. Vitamin D role in maintaining redox status during pediatric critical illness requires further study. |
| Cetinkaya 2015 | Evaluate the effect of vitamin D levels on early-onset sepsis (EOS) in term infants | Lower maternal and neonatal 25-OHD levels associated with EOS. Suggest that adequate vitamin D supplementation during pregnancy may be helpful to prevent EOS in term neonates. |
| Ahmed 2014 | Examine relationship between vitamin D status and hospitalization for ALRTI in Nigerian children. | ALRTI not associated with vitamin D status, but associated with less exposure to sunlight. Exposure to sunlight & vitamin D supplementation contributed to vitamin D status in this population. |
| Jat 2016 | Evaluate Vitamin D levels and its correlation with severity and outcome of pneumonia in children. | Majority (86.4%) of children were vitamin D deficient. Vitamin D levels were found different in cases and controls and were not related to severity and outcome of pneumonia. |
| Sankar 2017 | Evaluate association of severe vitamin D deficiency with clinically important outcomes in children with septic shock. | Prevalence of severe vitamin D deficiency is high in children with septic shock admitted to pediatric intensive care unit. Severe vitamin D deficiency at admission seems to be associated with lower rates of shock reversal at 24 hours of ICU stay. |
| Cizmeci 2015 | Investigate if neonates with early-onset neonatal sepsis (EONS) had lower levels of vitamin D | Cord-blood 25(OH) D levels of neonates with EONS significantly lower than that of the healthy controls, and a low level of cord-blood vitamin D was found to be associated with an increased risk of EONS. Further studies are warranted to confirm this association. |
| Khakshour 2015 | Clarify the association between vitamin D deficiency and acute respiratory infection in children below age 5 years. | The group of children with respiratory disorders, 9 (42.9%) exhibited vitamin D deficiency. No meaningful statistical relation vitamin D deficiency with acute respiratory infections (p>0.05) |
| Korwutthikulrangsri 2015 | Determine vitamin D status in critically ill children and its relationship with adrenal function | Higher prevalence of vitamin D deficiency in critically ill children in comparison to controls. Patients vitamin D deficiency had higher median (IQR) PRISM III score and higher proportion of mortality than those with serum 25-OHD of equal or more than 12 ng/mL. |
| Elmoneim 2016 | Association of the level of vitamin D on admission & length of stay in the PICU, or duration of mechanical ventilation. | High prevalence of vitamin D deficiency among PICU patients & significant association with increased LOS and need for mechanical ventilation. Not significant association with mortality rate. |



For peer review only - http://bmjopen.bmj.com/site/about/guidelines.xhtml

| | | |
|---|---|---|
| Dinlen 2016 | Determine the association between serum 25-hydroxy vitamin D [25(OH)D] levels and acute respiratory tract infections (ALRTI) in newborns | Lower blood 25(OH) D levels might be associated with increased risk of ALRTI in term newborn babies. Appropriate vitamin D supplementation during pregnancy and early childhood may enhance newborns' respiratory health. |
| Moreno-Solis 2015 | Examine prevalence of hypovitaminosis D in infants with acute bronchiolitis compared with control subjects and to evaluate the relationship between serum 25-hydroxyvitamin D (25(OH) D) and the severity of bronchiolitis. | Prevalence of hypovitaminosis D is high in Spanish infants with bronchiolitis. The severity of acute bronchiolitis increases with a decline in serum 25 (OH) D level. |
| Inamo 2011 | Relationship between serum vitamin D concentrations and severity of ALRI in hospitalized children in Japan. | Significantly more children with ALRI that required supplementary oxygen and ventilator management were vitamin D deficient. Findings suggest: immunomodulatory properties of vitamin D may influence the severity of ALRI. |
| Karatekin 2009 | Association of serum 25-hydroxy vitamin D concentrations in newborns with acute lower respiratory infection (ALRI) and without clinical signs of rickets, and their mothers | Newborns with subclinical vitamin D deficiency may have an increased risk of suffering from ALRI. Strong positive correlation between newborns' and mothers' 25(OH) D concentrations indicates that adequate vitamin D supplementation of mothers should be emphasized during pregnancy especially in winter months. |
| Roth 2010 | Investigate association between vitamin D status and ALRI | Vitamin D status associated with ALRI in early childhood.<br>Randomized trials needed to establish if interventions to improve vitamin D status could reduce burden of ALRI in early childhood. |
| Mathias 2017 | Determination of association of 25(OH)D with cathelicidin and DBP (D binding protein) in children with sepsis | No association between vitamin D and cathelicidin or DBP (D binding protein) levels |
| Dhandai 2018 | Assess vitamin D deficiency as possible risk factor for late-onset sepsis in term and late preterm neonates | Vitamin D deficient neonates are at greater risk for late onset sepsis compared to those with sufficient vitamin D levels |
| Vo 2018 | Investigate the association between circulating 25-hydroxyvitamin D status upon admission and disease severity in infants hospitalized for bronchiolitis. Also, to determine if the association differs by the form of 25(OH)D (total, bioavailable or free 25 (OH) D. | Infants with total 25 (OH) D < 20 ng/ml had higher risk of intensive care and longer hospital length-of-stay |
| Li 2018 | Assess vitamin D nutritional status of children of 3 days to 14 years and investigated the relationship between community-acquired pneumonia and serum 25(OH)D level | Children with low serum 25 (OH) D levels may be at higher risk of receiving mechanical ventilation and presenting with multiple organ dysfunction. Vitamin D supplementation could be beneficial for the treatment and prevention of CAP |

For peer review only



For peer review only - http://bmjopen.bmj.com/site/about/guidelines.xhtml

**Additional Table 9 Assay used in each study to measure Vitamin D levels**

| Assay | Paper | Total Number of studies |
|---|---|---|
| Radioimmunoassay | Madden 2012; Inamo 2011; Karatekin 2009; Roth 2009; Roth 2010; Sakka 2014; Moreno-Solis 2015; Khashour 2015 | 8 |
| Competitive binding enzyme linked immunoassay | Basha 2014 | 1 |
| Binding protein assay | Onwuneme 2015 (2) | 1 |
| Liquid chromatography-mass spectrometry | McNally 2012; Korwutthikulrangsri 2015; Binks 2014; Cetinkaya 2015; Cizmeci 2015; Asilioglu 2017; Onwuneme (1) | 7 |
| Chemiluminescence immunoassay or chemiluminescent tracer | Alonso 2015; Rey 2014; Sankar 2016; Shah 2016; Prasad 2015; Ahmed 2015; Lopez 2016; Alvarez 2016; Say 2017; Sankar 2017; Jat 2017; Mathias 2017; Li 2018 | 13 |
| ELISA | Ponnarmeni 2016; Hebbar 2014; Elmoneim 2016; Narang 2016; Jia 2017; El-Gamasy 2017; Halwany 2017; Banajeh 2009; Badawi 2017 | 9 |
| ELFA (enzyme linked fluorescent assay) | Bustos 2014 | 1 |
| Electrochemiluminescence Immunoassay (ECLIA) | Dayal 2014, Ebenezer 2016, Garcia Soler 2017; Cayir 2014; | 4 |
| Clinical Laboratory Improvement Amendments–approved Vitamin D assay (Elecsys; Roche Diagnostics, Indianapolis, Indiana) | Hurwitz 2017 | 1 |
| Commercial immunoassay lit (I RIA Diasorin UK) | Wayse 2004, Ayulo 2014, | 2 |
| Immunoassay analyzer | Rippel 2012 | 1 |
| APPLIED 3200 Biosystem | Dinlen 2016 | 1 |
| Not reported | Yaghmaie 2017; Dhandai 2018 | 2 |
| Abbott Architect assay (Abbott, Waukegan, Illinois) | Vo 2018 | 1 |

For peer review only

**Additional Table 10 Funding and ethical approval of included studies**

| Study | Funding | Approval of study and ethics |
|---|---|---|
| Madden 2012 | Reported | Children's Hospital Boston institutional review board. Informed consent obtained from family. |
| Lopez 2016 | Reported | Approved by the Ethical Committee of Clinical Investigation of Galicia (CEIC ref 010/015) and all of the regional ethics committees of the participant centres informed consent forms were obtained from either a parent or legal guardian for each subject before study inclusion. |
| Garcia-Soler 2017 | Not reported | Study protocol approved by regional research ethics committee. Obtained informed consent from family of patients. Data recorded anonymously (encrypted electronic database).<br>Project adhered to the principles of Declaration of Helsinki and standards for good clinical practice. |
| McNally 2012 | Reported | Research ethics board approval for vitamin D sub study obtained from 6 centers, representing 337 of the original 389 study participants. |
| Rippel 2012 | Not Reported | Approved by Ethics Committee of The Royal Children's Hospital Melbourne |
| Alonso 2015 | Reported | Regional Ethics Committee of the Principality of Asturias |
| Asilioglu 2017 | Not reported | Approval of study by the Local Ethics Committee of Ondokuz Mayıs University (Samsun, Turkey). |
| Ayulo 2014 | Not reported | Institutional Review Board: Montefiore Medical Centre |
| Rey 2014 | Reported | Hospital Ethics Committee |
| Shah 2016 | Reported | Ethical approval obtained from Institutional ethics committee. Parents of children satisfying criteria gave written informed consent for participation of their child in the study. |
| Ponnarmeni 2016 | Not reported | The institution's ethics committee. Informed consent obtained |
| Onwuneme 2015 (1) | Reported | Approval by ethics committees of: Children's University Hospital, Temple Street and Our Lady's Children's Hospital, Dublin, Ireland. Participants were informed and provided written consent before recruitment. |
| Jia 2017 | Not reported | Approved by Human Ethical Committee of Affiliated Hospital of Yan'an University, Yan'an. Informed consent was taken from mothers and/or parent of infants. |
| Halwany 2017 | Not reported | Approval by the Medical Ethics Committee of the Faculty of Medicine, Alexandria University |
| Sankar 2016 | Not reported | Institutional Ethics committee. Informed consent obtained |
| Say 2017 | Not reported | Approval by the local Ethics Committee and informed parental consent was obtained for all infants. |
| Sakka 2014 | Not reported | Not reported |
| Onwuneme2015 (2) | Reported | Ethics Committee of National Maternity Hospital. Informed written consent obtained from parents before recruitment |
| Dayal 2014 | Not reported | Ethics Committee of the Institute. Informed consent obtained. |
| Bustos 2016 | Reported | Comité Ético Científico del Servicio de Salud de Concepción |
| Hurwitz 2017 | Reported | Informed consent obtained and study protocol approved by the Institutional Review Boards at the University of Tennessee, St Jude Research Hospital, and the Centers for Disease Control and Prevention. |
| Cayir 2014 | Not reported | Approval from the institutional ethics committee and consent from the parents of all children in the study. |
| Badawi 2017 | Not reported | Children's Cairo University institutional review board approved study. Informed consent obtained from parents |
| Yaghmaie 2017 | Not reported | Study carried out in accordance with the Declaration of Helsinki, and the ethics committee of the Tehran University of Medical Sciences approved the protocols of the study. Records of patients' were kept confidential. Patients' consent provided |



For peer review only - http://bmjopen.bmj.com/site/about/guidelines.xhtml

| | | |
|---|---|---|
| | | for blood sampling and vitamin D serum level was assessed from patients' files, so no invasive method or extra blood sampling was done. |
| Basha 2014 | Not reported | Oral consent from the mothers. |
| Prasad 2015 | Not reported | Institutional review board approved the protocol. Written informed consent obtained from parents or guardians. |
| Wayse 2004 | Reported | Information not provided |
| El-Gamasy 2017 | Not reported | Study approved from the Ethical Committee of the Faculty of Medicine, Tanta University. Informed verbal or written parental consents from all subjects involved in the study. |
| Banajeh 2009 | Reported | Protocol approved and described in previous paper |
| Binks 2014 | Reported | Testing performed after approval by the Human Research Ethics Committee of the Northern Territory Department of Health and Menzies School of Health Research |
| Roth 2009 | Reported | Caregiver of each participant provided written informed consent and completed a questionnaire Study approved by the Human Research Ethics Board of the University of Alberta Health Sciences Faculties |
| Hebbar 2014 | Reported | Institutional Review Boards of Emory University and Children's Healthcare Atlanta Informed consent obtained |
| Ebenezer 2016 | Reported | Institutional Review Board (IRB), Informed consent from parents |
| Narang 2016 | Not reported | Not reported |
| Alvarez 2016 | Reported | Study approved by both the Emory University and Children's Healthcare of Atlanta Institutional Review Boards, and informed consent was obtained from patients' guardians prior to any study procedures |
| Cetinkaya 2015 | Not reported | Study protocol approved by the local Ethics Committee. Informed parental consent was obtained for all infants |
| Ahmed 2014 | Not reported | Informed written or oral consent obtained from the parents, and the study was approved by the National Hospital Abuja Ethics Committee |
| Jat 2016 | Not reported | Ethics committee approval was taken before commencing the study. Parent's informed consent was taken before enrolling children into study. |
| Sankar 2017 | No funding | Study approved by the institutional ethics committee. |
| Cizmeci 2015 | Reported | Acquisition of cord-blood was approved by the local ethics committee of Fatih University Medical School. |
| Khakshour 2015 | Not reported | Obtained informed consent from parents, data collection done using demographic questionnaire and serum level of 25-dehydroxycalcciferol was measured. Samples taken by a trained nurse at admission based on physician's orders. |
| Korwutthikulrangsri 2015 | Not reported | Ramathibodi Hospital Ethics Committee. Written informed consent from legal guardians of all participants |
| Elmoneim 2016 | Reported | MCH hospital Ethics Committee approved study protocol |
| Dinlen 2016 | Not reported | Study approved by the Local Ethics Committee. All parents fully informed about this investigation as well as its aim. Written consent was obtained from all parents. |
| Moreno-Solis 2015 | Reported | Written informed consent obtained from parents or legal guardian of all enrolled children. Protocol of study approved by the Ethics Committee before the beginning of this study. |
| Inamo 2011 | Not reported | Ethics Committee of Nihon University Nerima-Hikarigaoka Hospital. Informed consent obtained from the parents of all patients before inclusion in the study. |
| Karatekin 2009 | Not reported | Study approved by the Institution's Ethics Committee, and informed consent was obtained from the study participants. |
| Roth 2010 | Reported | Approved by The Johns Hopkins Bloomberg School of Public Health Institutional Review Board and the ethics committee of the Bangladesh Institute for Child Health at the Dhaka Shishu Hospital, Bangladesh. Signed permission prior to enrolment from parents/guardians. |



For peer review only - http://bmjopen.bmj.com/site/about/guidelines.xhtml

| | | |
|---|---|---|
| Mathias 2017 | Reported | The institution's Human Investigation Committee approved the study after a full board review and informed consent obtained |
| Dhandai 2018 | Not reported | The institute's ethics committee approved the study and informed written consent had been given by their parents or guardians |
| Vo 2018 | Reported | The institutional review boards at all participating sites approved the protocol and informed consent obtained from the infants' parents/legal guardians |
| Li 2018 | Not reported | Protocols for the study and written consent approved by the ethics committee of the Capital Institute of Pediatrics at Beijing, China |

For peer review only



For peer review only - http://bmjopen.bmj.com/site/about/guidelines.xhtml

**Additional Table 11 Age groups of children in each study**

| First author, date | Age group |
|---|---|
| Wayse 2004 | <5 years |
| Karatekin 2009 | Neonates |
| Roth 2009 | <2 years |
| Roth 2010 | 1–18 months |
| Inamo 2011 | <4 years |
| Madden 2012 | <21 years |
| Rippel 2012 | 16.5 (3.1 to 75.2) months |
| McNally 2012 | Newborn to 17 years |
| Ayulo 2014 | 1 to 21 years |
| Dayal 2014 | 3 months to 12 years |
| Hebbar 2014 | 0 to 18 years |
| Rey 2014 | <16 years |
| Cetinkaya 2015 | >37 weeks |
| Onwuneme (1) 2015 | <12 years |
| Onwuneme (2) 2015 | <32 weeks gestation |
| Prasad 2015 | 2 months to 12 years |
| Alonso 2015 | 1 month to 13 years |
| Korwutthikulrangsri 2015 | 79 (61) cases; 92 (40) controls months |
| Cizmeci 2015 | Neonates |
| Shah 2016 | 1 month to 15 years |
| Ponnarmeni 2016 | 1 to 12 years |
| Sankar 2016 | 1 month to 17 years |
| Bustos 2016 | >37 weeks and <15 years |
| Ebenezer 2016 | <18 years |
| Elmoneim 2016 | <14 years |
| Narang 2016 | 2 months to 5 years |
| Dinlen 2016 | Neonates |
| Lopez 2016 | 0 to >48 months |
| Alvarez 2016 | 0 to 18 years |
| Garcia-Soler 2017 | 6 months to 17 years |
| Sankar 2017 | <17 years |
| Ahmed 2015 | 2 to 60 months |
| Cayir 2014 | 1 to 13 years |
| Say 2017 | <37 weeks |
| Asilioglu 2017 | <=18 years |
| Basha 2014 | <5 years |
| Jia 2017 | <1 year |
| Jat 2017 | 1 month to 12 years |
| Yaghmaie 2017 | Age range not stated |
| El-Gamasy 2017 | 3 months to 12 years |
| Binks 2014 | <3 years |
| Halwany 2017 | >1 month to <= 5 years |
| Badawi 2017 | 1 month to 12 years |
| Moreno-Solis 2015 | 1 to 11 months |
| Sakka 2014 | <2 years |
| Hurwitz 2017 | <5 years |
| Banajeh 2009 | 2 to 59 months |
| Khakshour 2015 | < 5 years |
| Mathias 2017 | <= 18 years |
| Dhandai 2018 | Neonates |
| Vo 2018 | < 12 months |
| Li 2018 | 3 days to 14 years |

For peer review only



For peer review only - http://bmjopen.bmj.com/site/about/guidelines.xhtml

**Additional Table 12 Prevalence of vitamin D deficiency in each study of acute and critically ill children (sorted from highest to lowest)**

| Study | Prevalence of vitamin D deficiency (%) | Number of vitamin D deficient children | Total number of children |
|---|---|---|---|
| Wayse 2004 | 95.00 | 76 | 80 |
| Sakka 2014 | 92.70 | 89 | 96 |
| Karatekin 2009 | 92.00 | 23 | 25 |
| Onwuneme2015 (2) | 91.48 | 86 | 94 |
| Say 2017 | 87.00 | 87 | 100 |
| Dinlen 2016 | 86.70 | 26 | 30 |
| Cetinkaya 2015 | 84.00 | 42 | 50 |
| Jat 2016 | 84.00 | 42 | 50 |
| Roth 2010 | 84.00 | 21 | 25 |
| Prasad 2015 | 83.75 | 67 | 80 |
| Jia 2017 | 83.64 | 92 | 110 |
| Shah 2016 | 83.11 | 128 | 154 |
| Korwutthikulrangsri 2015 | 78.12 | 25 | 32 |
| Sankar 2016 | 74.26 | 75 | 101 |
| Sankar 2017 | 72.09 | 31 | 43 |
| Cizmeci 2015 | 70.00 | 28 | 40 |
| Mathias 2017 | 70.00 | 28 | 41 |
| McNally 2012 | 69.02 | 225 | 326 |
| Basha 2014 | 65.43 | 53 | 81 |
| Yaghmaie 2017 | 64.63 | 53 | 82 |
| Dhandai 2018 | 63.30 | 38 | 60 |
| Hebbar 2014 | 60.66 | 37 | 61 |
| Onwuneme 2015 (1) | 59.16 | 71 | 120 |
| Alvarez 2016 | 58.00 | 29 | 50 |
| Cayir 2014 | 56.82 | 50 | 88 |
| Elmoneim 2016 | 56.67 | 17 | 30 |
| Narang 2016 | 56.00 | 28 | 50 |
| Li 2018 | 55.83 | 445 | 797 |
| Ponnarmeni 2016 | 50.81 | 63 | 124 |
| Asilioglu 2017 | 48.00 | 120 | 250 |
| Badawi 2017 | 44.30 | 39 | 88 |
| Garcia-Soler 2017 | 43.82 | 149 | 340 |



For peer review only - http://bmjopen.bmj.com/site/about/guidelines.xhtml

| | | | |
|---|---|---|---|
| Bustos 2016 | 43.30 | 39 | 90 |
| Ebenezer 2016 | 40.38 | 21 | 52 |
| Madden 2012 | 40.12 | 205 | 511 |
| Halwany 2017 | 37.25 | 38 | 102 |
| Banajeh 2009 | 36.71 | 29 | 79 |
| Rippel 2012 | 34.49 | 109 | 316 |
| El-Gamasy 2017 | 32.50 | 26 | 80 |
| Ahmed 2014 | 30.00 | 15 | 50 |
| Rey 2014 | 29.49 | 46 | 156 |
| Vo 2018 | 29.33 | 298 | 1,016 |
| Inamo 2011 | 28.50 | 8 | 28 |
| Dayal 2014 | 25.00 | 23 | 92 |
| Khakshour 2015 | 24.32 | 9 | 37 |
| Lopez 2016 | 24.20 | 84 | 347 |
| Alonso 2015 | 15.63 | 45 | 288 |
| Ayulo 2014 | 15.63 | 61 | 216 |
| Binks 2014 | 14.86 | 11 | 74 |
| Moreno-Solis 2015 | 13.33 | 9 | 48 |
| Hurwitz 2017 | 12.22 | 11 | 90 |
| Roth 2009 | 4.69 | 3 | 64 |

For peer review only

**Additional Table 13 Characteristics of studies used in the meta-analysis of prevalence**

| Study | Total number of patients | Total number of vitamin D deficient patients | Country, setting | Age range | Design | Quality score (NOS) |
|---|---|---|---|---|---|---|
| Vo 2018 | 1,016 | 298 | USA, PICU | < 12 months | cohort | 8 |
| Li 2018 | 797 | 445 | China, PICU | 3 days to 14 years | case-control | 8 |
| Madden 2012 | 511 | 205 | Boston US, PICU | < 21 years | cohort | 8 |
| Lopez 2016 | 347 | 84 | Spain, hospitalised | 0 to >48 months | cohort | 8 |
| Garcia-Soler 2017 | 340 | 149 | Spain, PICU | 6 months to 17 years | cohort | 7 |
| McNally 2012 | 326 | 225 | Canada, PICU | newborn to 17 years of age | cohort | 7 |
| Rippel 2012 | 316 | 109 | Australia, PICU | 16.5 (3.1–75.2) months | cohort | 8 |
| Alonso 2015 | 288 | 45 | Spain, PICU | 1 month to 13 years | cross sectional | 5 |
| Asilioglu 2017 | 250 | 120 | Turkey, PICUs | <= 18 years | cohort | 6 |
| Ayulo 2014 | 216 | 61 | Spain, PICU | 1 month to 13 years | cross sectional | 5 |
| Rey 2014 | 156 | 46 | Spain, PICU | <16 years | cohort | 7 |
| Shah 2016 | 154 | 128 | India,PICU | 1 month-15 years | cohort | 6 |
| Ponnarmeni 2016 | 124 | 63 | India, PICU | aged 1–12 years | cohort | 6 |
| Onwuneme 2015 (1) | 120 | 71 | Ireland, PICU | <12 years old | cohort | 8 |
| Jia 2017 | 110 | 92 | China, PICU | infants <1 years old | case-control | 7 |
| Halwany 2017 | 102 | 38 | Alexandria, PICU | >1 month to ≤ 5 years | cohort | 6 |
| Sankar 2016 | 101 | 75 | India, PICU | 1 month to 17 years | cohort | 8 |
| Say 2017 | 100 | 87 | Turkey, NICU | gestational age <37 weeks | case-control | 6 |
| Sakka 2014 | 96 | 89 | Egypt, hospitalized | infants (<2 years old) | case-control | 8 |
| Onwuneme 2015 (2) | 94 | 86 | Ireland, NICU | preterm infants at <32 weeks gestation | cohort | 8 |
| Dayal 2014 | 92 | 23 | India, PICU | 3 months to 12 years | cohort | 6 |
| Bustos 2016 | 90 | 39 | Chile, PICU | >37 weeks and <15 years | cohort | 7 |
| Hurwitz 2017 | 90 | 11 | USA, hospitalised | <5 years old | cohort | 6 |
| Cayir 2014 | 88 | 50 | Turkey, PICU | 1 to 13 years | case-control | 7 |
| Badawi 2017 | 88 | 39 | Cairo, Egypt PICU | 1 month to 12 years | cohort | 7 |
| Yaghmaie 2017 | 82 | 53 | Iran, PICU | children undefined | cross sectional | 4 |
| Basha 2014 | 81 | 53 | Cairo Egypt, PICU | <5 years old | case-control | 7 |
| Prasad 2015 | 80 | 67 | India, PICU | 2 months-12 years | cohort | 7 |
| Wayse 2004 | 80 | 76 | Indapur India, PICU | <5 years | case-control | 6 |
| El-Gamasy 2017 | 80 | 26 | Egypt, PICU | 3 months to 12 years | cohort | 7 |
| Banajeh 2009 | 79 | 29 | Iran, hospitalised | aged 2–59 months | cohort | 7 |
| Binks 2014 | 74 | 11 | Australia, PICU | <3 years old | cross sectional | 6 |
| Roth 2009 | 64 | 3 | Canada, PICU | aged 1-25 months | case-control | 7 |
| Hebbar 2014 | 61 | 37 | Atlanta, PICU | 0 to 18 years | cohort | 6 |
| Dhandai 2018 | 60 | 38 | India, NICU | neonates | cohort | 7 |



For peer review only - http://bmjopen.bmj.com/site/about/guidelines.xhtml

| | | | | | | |
|---|---|---|---|---|---|---|
| Ebenezer 2016 | 52 | 21 | India, PICU | <18 years | cohort | 6 |
| Narang 2016 | 50 | 28 | Punjab, India, PICU | 2 months to 5 years | case-control | 6 |
| Alvarez 2016 | 50 | 29 | Atlanta, PICU | 0 to 18 years | cross sectional | 6 |
| Cetinkaya 2015 | 50 | 42 | Instabul/Turkey, NICU | neonates | case-control | 6 |
| Ahmed 2014 | 50 | 15 | Nigeria, PICU | 2–60 months | case-control | 8 |
| Jat 2016 | 50 | 42 | India, PICU | 1 month to 12 years | case-control | 8 |
| Sankar 2017 | 43 | 31 | India, PICU | <=17 years | cohort | 8 |
| Mathias 2017 | 41 | 28 | USA, PICU | <= 18 years | cohort | 6 |
| Cizmeci 2015 | 40 | 28 | Instabul/Turkey, NICU | neonates | case-control | 7 |
| Khakshour 2015 | 37 | 9 | Iran, hospitalized | <5 years | case-control | 5 |
| Korwutthikulrangsri 2015 | 32 | 25 | Bangkok, PICU | months not more specific range | cohort | 7 |
| Elmoneim 2016 | 30 | 17 | Saudi Arabia, PICU | <14 years | cohort | 7 |
| Dinlen 2016 | 30 | 26 | Ankara Turkey, NICU | neonates | case-control | 6 |
| Moreno-Solis 2015 | 48 | 9 | Spain, PICU | infants 1–11 months | case-control | 6 |
| Inamo 2011 | 28 | 8 | Tokyo Japan, PICU | 1-48 months | cohort | 6 |
| Karatekin 2009 | 25 | 23 | Istanbul Turkey, NICU | neonates | case-control | 6 |
| Roth 2010 | 25 | 21 | Bangladesh | 1-18 months | case-control | 8 |

Studies arranged from largest to smallest total sample size. NOS = Newcastle Ottawa Score



For peer review only - http://bmjopen.bmj.com/site/about/guidelines.xhtml

**Additional Table 14 Sensitivity analyses for prevalence of vitamin D deficiency in acute and critically ill children**

| Patient category | Number of studies (Total number of individuals; number of vitamin D deficient individuals) | Pooled proportion % (95% CI) Random effects model | 95% PI | Pooled proportion % (95% CI) Fixed effects model | Heterogeneity ($I^2$) % (95% CI) | Q value, d.f. p-value for heterogeneity | Median % (IQR) of vitamin D deficiency |
|---|---|---|---|---|---|---|---|
| Acute and critically ill children, excluding studies that used other thresholds | 40 (6,543; 3,000) | 53.0 (46.4-59.5) | 17.7-85.5 | 45.1 (43.8-46.5) | 95.5 (94.5-96.2) | 859.8, 39, < 0.0001 | 55.9 (33.4-55.9) |
| Sample size >= 82 (large) | 26 (6,094; 2,731) | 51.5 (43.6-59.4) | 16.5-85.2 | 44.0 (42.6-45.3) | 96.8 (96.0-97.4) | 773.1, 25, < 0.0001 | 46.2 (30.7-67.9) |
| Sample size < 82 (small) | 26 (1,340; 742) | 58.2 (47.5-68.2) | 13.2-92.8 | 54.8 (51.7-58.0) | 90.9 (87.9-93.2) | 275.4, 25, < 0.0001 | 62.0 (33.6-82.3) |
| Cohort studies | 30 (5,119; 2,197) | 49.6 (42.7-56.4) | 17.6-81.9 | 42.1 (40.7-43.6) | 94.9 (93.6-95.9) | 567.0, 29, < 0.0001 | 44.1 (33.0-67.1) |
| Case-control | 18 (1,821; 1,138) | 68.1 (56.5-77.8) | 19.3-95.0 | 60.8 (58.2-63.3) | 93.0 (90.4-94.9) | 243.9, 17, 0.0001 | 76.8 (55.9-86.0) |
| Cross sectional | 4 (494; 138) | 34.8 (12.8-66.0) | 0.10-99.6 | 30.3 (25.9-35.2) | 96.7 (94.0-98.2) | 90.4, 3, < 0.0001 | 36.8 (15.4-59.7) |
| Studies from India | 11 (886; 592) | 68.9 (54.9-80.1) | 18.3-95.6 | 64.0 (60.4-67.5) | 96.7 (94.0 98.2) | 140.2, 10, < 0.0001 | 72.1 (53.4-83.4) |
| Studies from Turkey | 7 (583; 376) | 76.3 (60.9-87.0) | 20.9-97.5 | 61.0 (56.6-65.2) | 91.1 (84.2-95.0) | 67.5, 6, < 0.0001 | 84 (63.4-86.8) |
| Studies that only recruited neonates | 7 (399; 330) | 83.0 (73.1-90.0) | 42.4-97.0 | 80.7 (76.1-84.5) | 76.6 (51.0-88.9) | 25.7, 6, 0.0003 | 86.7 (77.0-89.2) |
| Studies with children of all other ages except neonates | 45 (7,035; 3,143) | 49.7 (43.5-55.8) | 16.1-83.5 | 44.2 (43.0-45.5) | 95.2 (94.3-96.0) | 919.9, 44, < 0.0001 | 48.0 (29.5-68.3) |

CI = confidence intervals; $I^2$ = heterogeneity; df = degrees of freedom. Vitamin D deficiency defined in our study as <50 nmol/L (20 ng/mL)
$I^2$ statistic used to estimate heterogeneity between pooled studies: $I^2$ >= 75% was considered as high heterogeneity, PI= Prediction interval; IQR = interquartile range: Lower (Q1) to Upper (Q3) quartile



For peer review only - http://bmjopen.bmj.com/site/about/guidelines.xhtml

**Additional Table 15 Multivariate meta-regression model for prevalence**

| Predictors | k | b-coefficient | se | t-value | p-value | ci.lb | ci.ub | F-value | I^2 (%) | R^2 (%) | QE |
|---|---|---|---|---|---|---|---|---|---|---|---|
| Full model: year + clinical setting + quality score + design + country group + total sample size | 52 | | | | | | | 4.57 | 95.7 | 32.9 | 702.6, p < 0.0001 |
| year | | -0.015 | 0.058 | -0.256 | 0.799 | -0.132 | 0.103 | | | | |
| total study sample size | | -0.001 | 0.001 | -0.731 | 0.469 | -0.002 | 0.001 | | | | |
| country group (group 1 or 2 versus group 3) | | 1.009 | 0.356 | 2.831 | 0.007 | 0.291 | 1.726 | | | | |
| clinical setting (NICU versus PICU) | | -1.573 | 0.465 | -3.384 | 0.002 | -2.510 | -0.637 | | | | |
| design group (cohort vs other i.e. case-control or cross sectional) | | 0.482 | 0.324 | 1.488 | 0.144 | -0.170 | 1.135 | | | | |
| quality score | | 0.296 | 0.170 | 1.744 | 0.088 | -0.046 | 0.637 | | | | |

k = number of outcomes included in the model fitting; se = standard errors of the coefficients; ci.lb = lower bound of the confidence intervals for the coefficients; ci.ub = upper bound of the confidence intervals for the coefficients; QE = test statistic for the test of (residual) heterogeneity; I^2 = residual heterogeneity / unaccounted variability; R^2 (amount of heterogeneity accounted for; PICU = pediatric intensive care units, NICU = neonatal intensive care units



For peer review only - http://bmjopen.bmj.com/site/about/guidelines.xhtml

**Additional Table 16 Characteristics of studies included in the meta-analysis for prevalence in individuals with sepsis**

| Study | Total number of patients with sepsis | Total number of vitamin D deficient patients with sepsis | Country, setting | Age | Design | Quality score (NOS) |
|---|---|---|---|---|---|---|
| Shah 2016 | 160 | 135 | India, PICU | 1 month to 16 years | cohort | 6 |
| Asilioglu 2017 | 120 | 20 | Turkey, PICU | 1 month to ≤18 years | cohort | 7 |
| Say 2017 | 100 | 87 | Turkey, NICU | preterm infants at <37 weeks gestation | case-control | 6 |
| Dhandai 2018 | 60 | 38 | India, NICU | neonates | cohort | 7 |
| Ponnarmeni 2016 | 62 | 32 | India, PICU | 1 to 12 years | cohort | 6 |
| McNally 2012 | 48 | 33 | Canada, PICU | newborn to 17 years | cohort | 7 |
| Onwuneme 2015 (2) | 46 | 32 | Ireland, NICU | preterm infants at <32 weeks gestation | cohort | 8 |
| El-Gamasy 2017 | 46 | 20 | Egypt, pediatric emergency department | 3 months to 12 years | cohort | 7 |
| Sankar 2017 | 43 | 31 | India, PICU | <=17 years | cohort | 8 |
| Mathias 2017 | 41 | 28 | USA, PICU | <=18 years | cohort | 6 |
| Cizmeci 2015 | 40 | 28 | Turkey, NICU | neonates | case-control | 7 |
| Onwuneme 2015 (1) | 35 | 32 | Ireland, PICU | <12 years old | cohort | 8 |
| Hebbar 2014 | 30 | 17 | Atlanta, PICU | 0 to 18 years | cohort | 6 |
| Ebenezer 2016 | 16 | 8 | India, PICU | <18 years | cohort | 6 |
| Korwutthikulrangsri 2015 | 12 | 4 | Bangkok, PICU | moths (<8/9) | cohort | 7 |
| Prasad 2015 | 11 | 9 | India, PICU | 2 months to 12 years | cohort | 7 |
| Bustos 2016 | 10 | 7 | Chile, PICU | >37 weeks and < than 15 years | cohort | 7 |
| Dayal 2014 | 9 | 4 | India, tertiary care hospital | 3 months to 12 years | cohort | 6 |



For peer review only - http://bmjopen.bmj.com/site/about/guidelines.xhtml

**Additional Table 17 Sensitivity analyses for prevalence of vitamin D deficiency in acute and critically ill children with sepsis**

| Patient category | Number of studies (Total number of individuals; number of vitamin D deficient individuals) | Pooled proportion (%, 95% CI) Random effects | 95% PI | Pooled proportion (%, 95% CI) Fixed effects | Heterogeneity ($I^2$) %, 95% CI | Q value, d.f. p-value for heterogeneity | Median % (IQR) of vitamin D deficiency |
|---|---|---|---|---|---|---|---|
| Excluding studies that used other thresholds | 14 (654; 395) | 62.0 (47.3-74.7) | 13.3-94.5 | 60.4 (56.0-64.7) | 89.7 (84.5-93.2) | 126.5, 13, < 0.0001 | 65.8 (50.4-70.0) |
| Sample size >= 42 (large) | 9 (685; 428) | 63.2 (44.6-78.5) | 9.4-96.6 | 62.5 (58.2-66.6) | 94.3 (91.1-96.3) | 139.2, 8 < 0.0001 | 68.8 (51.6-72.1) |
| Sample size < 42 (small) | 9 (204; 137) | 64.7 (52.5-75.3) | 29.5-89.0 | 64.7 (57.4-71.4) | 57.9 (11.8-79.9) | 19.0, 8, 0.0148 | 68.3 (50.0-70.0) |
| Cohort studies | 16 (749; 450) | 61.4 (48.6-72.8) | 15.5-93,2 | 60.0 (55.9-63.9) | 88.8 (83.5-92.4) | 134.2, 15, < 0.0001 | 65.8 (48.6-70.5) |
| Case-control | 2 (140; 115) | 80.0 (58.8-91.8) | NA | 81.0 (73.3-86.9) | 81.3 (20.5-95.6) | 5.35, 1, 0.0207 | 78.5 (74.2-82.8) |
| Studies from India | 7 (361; 257) | 66.0 (51.4-78.1) | 21.3-93.3 | 69.2 (63.9-74.1) | 81.1 (61.8-90.6) | 31.7, 6 < 0.0001 | 63.3 (50.8-77.0) |
| Studies from Turkey | 3 (260; 135) | 59.2 (13.6-93.1) | 0.00-100 | 51.2 (43.2-59.3) | 97.8 (95.8- 98.8) | 90.0, 2, < 0.0001 | 70.0 (43.3-78.5) |
| Studies that only included neonates | 4 (246; 185) | 73.7 (60.3-83.8) | 15.9-97.7 | 73.5 (67.3-78.9) | 76.0 (34.1-91.3) | 12.5, 3, 0.0058 | 69.8 (68.0-74.3) |
| Studies that included children of all other ages except neonates | 14 (643; 380) | 60.7 (45.5-74.0) | 11.8-94.7 | 58.7 (54.2-63.0) | 90.1 (85.2-93.4) | 131.8, 13 < 0.0001 | 62.5 (45.8-71.6) |

CI = confidence intervals; $I^2$ = heterogeneity; df = degrees of freedom. Vitamin D deficiency defined in our study as <50 nmol/L (20 ng/mL). $I^2$ statistic used to estimate heterogeneity between pooled studies: $I^2$ >= 75% was considered as high heterogeneity; PI= Prediction interval; IQR = interquartile range: Lower (Q1) to Upper (Q3) quartile; NA= Not available

For peer review only

**Additional Table 18 Sensitivity analyses for mortality. Pooled odds ratios for risk of mortality in deficient versus not deficient children**

| Patient category | Number of studies (Total number of individuals) | Pooled OR (95% CI) Vitamin D deficient/ Vitamin D non-deficient p-value | | Heterogeneity ($I^2$) % (95% CI) | Q value, d.f. p-value for heterogeneity | Eggers p-value |
|---|---|---|---|---|---|---|
| | | Random effects | Fixed effects | | | |
| Excluding studies that used other thresholds | 14 (2,030) | 1.59 (1.05-2.41) p-value = 0.028 | 1.52 (1.08-2.13) p-value = 0.016 | 24.3 (0.0-59.9) | 17.18, 13, 0.1910 | p-value = 0.120 |
| Studies from India | 7 (646) | 1.08 (0.70-1.69) p-value = 0.710 | 1.08 (0.70-1.69) p-value = 0.710 | 0.0 (0.0-62.4) | 4.56, 6, 0.589 | Number of studies too small to test for small study effects (k.min=10) |

CI = Confidence Intervals; $I^2$ = I squared statistic used to estimate heterogeneity (inconsistency); df = degrees of freedom, results reported in 1 decimal place; OR= odds ratio. Vitamin D deficiency defined as < 50 nmol/L or 20 ng/ml. We used the $I^2$ statistic to estimate heterogeneity between pooled studies: $I^2$ >= 75% was considered as high heterogeneity.



For peer review only - http://bmjopen.bmj.com/site/about/guidelines.xhtml

## Additional Figures

**Additional Figure 1 Funnel plot of studies of prevalence of vitamin D deficiency in acute and critically ill children.** Horizontal axis shows logit transformed proportion and the standard error of the logit transformed proportion is plotted on the vertical axis. Each dot represents an individual study and the vertical line represents the pooled proportion from a random-effects meta-analysis. The diagonal lines represent pseudo 95% confidence limits (p=0.01, Egger's test).

Plot A shows the funnel plot before trim and fill method was applied and B after. Solid circles in plot B represent original data and open circles represent imputed filled studies (11 studies added) on the left-hand side of the funnel plot.

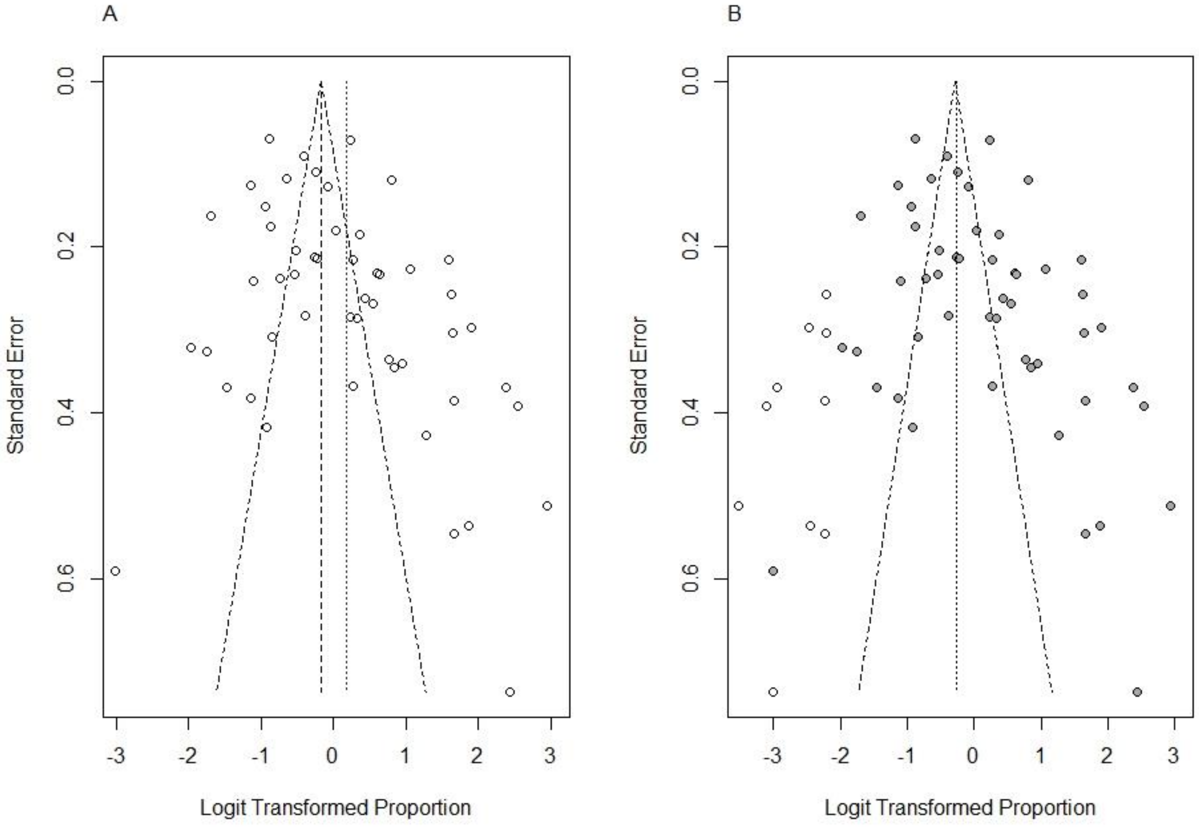




For peer review only - http://bmjopen.bmj.com/site/about/guidelines.xhtml

**Additional Figure 2. Pooled prevalence estimate for vitamin D deficiency in acute and critically ill children (by country group).** Forest plot shows results from the random effects model. Diamonds represent pooled proportion of vitamin D deficiency for the countries in each subgroup (group 1, group2, group 3). The diamond at the bottom shows the overall pooled estimate of all the 52 studies. Each square shows the prevalence estimate of each study and the horizontal line across each square represents the 95% confidence interval (CI) of the prevalence estimate.

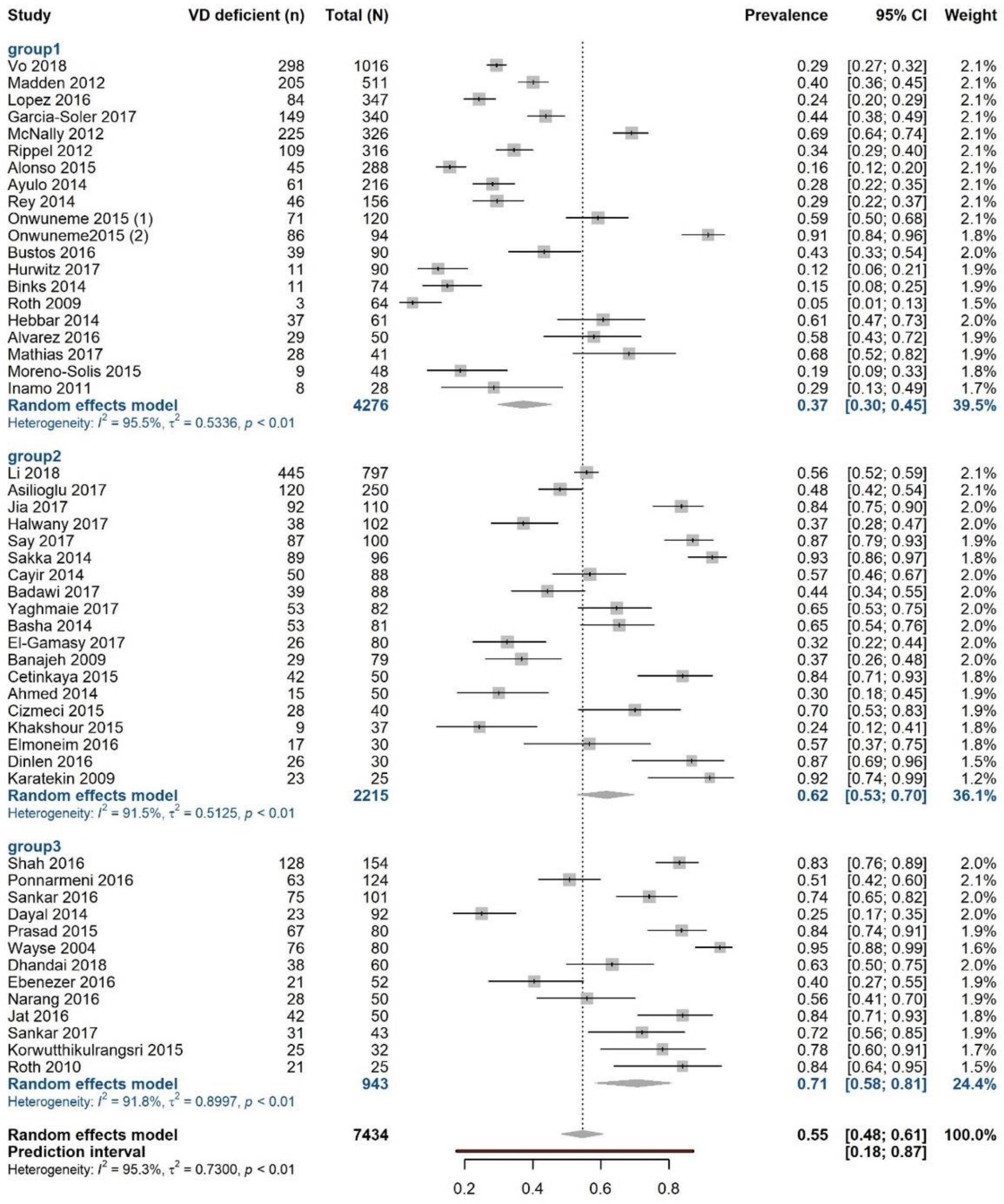

**Additional Figure 3. Pooled prevalence estimates for vitamin D deficiency in acute and critically ill children (neonates versus all other age groups).** Forest plot shows results from the random effects model. Diamonds represent pooled proportion of vitamin D deficiency for the studies in neonates and all other age groups. The diamond at the bottom shows the overall pooled estimate of all the 52 studies. Each square shows the prevalence estimate of each study and the horizontal line across each square represents the 95% confidence interval (CI) of the prevalence estimate.

| Study | VD deficient (n) | Total (N) | Prevalence | 95% CI | Weight |
|---|---|---|---|---|---|
| **neonates** | | | | | |
| Karatekin 2009 | 23 | 25 | 0.92 | [0.74; 0.99] | 1.2% |
| Cizmeci 2015 | 28 | 40 | 0.70 | [0.53; 0.83] | 1.9% |
| Dinlen 2016 | 26 | 30 | 0.87 | [0.69; 0.96] | 1.5% |
| Cetinkaya 2015 | 42 | 50 | 0.84 | [0.71; 0.93] | 1.8% |
| Onwuneme2015 (2) | 86 | 94 | 0.91 | [0.84; 0.96] | 1.8% |
| Say 2017 | 87 | 100 | 0.87 | [0.79; 0.93] | 1.9% |
| Dhandai 2018 | 38 | 60 | 0.63 | [0.50; 0.75] | 2.0% |
| **Random effects model** | | **399** | **0.83** | **[0.73; 0.90]** | **12.2%** |
| Heterogeneity: $I^2 = 76.6\%$, $\tau^2 = 0.4533$, $p < 0.01$ | | | | | |
| **other** | | | | | |
| Mathias 2017 | 28 | 41 | 0.68 | [0.52; 0.82] | 1.9% |
| Vo 2018 | 298 | 1016 | 0.29 | [0.27; 0.32] | 2.1% |
| Li 2018 | 445 | 797 | 0.56 | [0.52; 0.59] | 2.1% |
| Rippel 2012 | 109 | 316 | 0.34 | [0.29; 0.40] | 2.1% |
| Jia 2017 | 92 | 110 | 0.84 | [0.75; 0.90] | 2.0% |
| Halwany 2017 | 38 | 102 | 0.37 | [0.28; 0.47] | 2.0% |
| Sakka 2014 | 89 | 96 | 0.93 | [0.86; 0.97] | 1.8% |
| Hurwitz 2017 | 11 | 90 | 0.12 | [0.06; 0.21] | 1.9% |
| Basha 2014 | 53 | 81 | 0.65 | [0.54; 0.76] | 2.0% |
| Wayse 2004 | 76 | 80 | 0.95 | [0.88; 0.99] | 1.6% |
| Banajeh 2009 | 29 | 79 | 0.37 | [0.26; 0.48] | 2.0% |
| Binks 2014 | 11 | 74 | 0.15 | [0.08; 0.25] | 1.9% |
| Narang 2016 | 28 | 50 | 0.56 | [0.41; 0.70] | 1.9% |
| Ahmed 2014 | 15 | 50 | 0.30 | [0.18; 0.45] | 1.9% |
| Khakshour 2015 | 9 | 37 | 0.24 | [0.12; 0.41] | 1.8% |
| Moreno-Solis 2015 | 9 | 48 | 0.19 | [0.09; 0.33] | 1.8% |
| Inamo 2011 | 8 | 28 | 0.29 | [0.13; 0.49] | 1.7% |
| Roth 2010 | 21 | 25 | 0.84 | [0.64; 0.95] | 1.5% |
| Roth 2009 | 3 | 64 | 0.05 | [0.01; 0.13] | 1.5% |
| Madden 2012 | 205 | 511 | 0.40 | [0.36; 0.45] | 2.1% |
| Lopez 2016 | 84 | 347 | 0.24 | [0.20; 0.29] | 2.1% |
| Garcia-Soler 2017 | 149 | 340 | 0.44 | [0.38; 0.49] | 2.1% |
| McNally 2012 | 225 | 326 | 0.69 | [0.64; 0.74] | 2.1% |
| Alonso 2015 | 45 | 288 | 0.16 | [0.12; 0.20] | 2.1% |
| Asilioglu 2017 | 120 | 250 | 0.48 | [0.42; 0.54] | 2.1% |
| Ayulo 2014 | 61 | 216 | 0.28 | [0.22; 0.35] | 2.1% |
| Rey 2014 | 46 | 156 | 0.29 | [0.22; 0.37] | 2.1% |
| Shah 2016 | 128 | 154 | 0.83 | [0.76; 0.89] | 2.0% |
| Ponnarmeni 2016 | 63 | 124 | 0.51 | [0.42; 0.60] | 2.1% |
| Onwuneme 2015 (1) | 71 | 120 | 0.59 | [0.50; 0.68] | 2.1% |
| Sankar 2016 | 75 | 101 | 0.74 | [0.65; 0.82] | 2.0% |
| Dayal 2014 | 23 | 92 | 0.25 | [0.17; 0.35] | 2.0% |
| Bustos 2016 | 39 | 90 | 0.43 | [0.33; 0.54] | 2.0% |
| Cayir 2014 | 50 | 88 | 0.57 | [0.46; 0.67] | 2.0% |
| Badawi 2017 | 39 | 88 | 0.44 | [0.34; 0.55] | 2.0% |
| Yaghmaie 2017 | 53 | 82 | 0.65 | [0.53; 0.75] | 2.0% |
| Prasad 2015 | 67 | 80 | 0.84 | [0.74; 0.91] | 1.9% |
| El-Gamasy 2017 | 26 | 80 | 0.32 | [0.22; 0.44] | 2.0% |
| Hebbar 2014 | 37 | 61 | 0.61 | [0.47; 0.73] | 2.0% |
| Ebenezer 2016 | 21 | 52 | 0.40 | [0.27; 0.55] | 1.9% |
| Alvarez 2016 | 29 | 50 | 0.58 | [0.43; 0.72] | 1.9% |
| Jat 2016 | 42 | 50 | 0.84 | [0.71; 0.93] | 1.8% |
| Sankar 2017 | 31 | 43 | 0.72 | [0.56; 0.85] | 1.9% |
| Korwutthikulrangsri 2015 | 25 | 32 | 0.78 | [0.60; 0.91] | 1.7% |
| Elmoneim 2016 | 17 | 30 | 0.57 | [0.37; 0.75] | 1.8% |
| **Random effects model** | | **7035** | **0.50** | **[0.44; 0.56]** | **87.8%** |
| Heterogeneity: $I^2 = 95.2\%$, $\tau^2 = 0.6427$, $p < 0.01$ | | | | | |
| **Random effects model** | | **7434** | **0.55** | **[0.48; 0.61]** | **100.0%** |
| **Prediction interval** | | | | **[0.18; 0.87]** | |
| Heterogeneity: $I^2 = 95.3\%$, $\tau^2 = 0.7300$, $p < 0.01$ | | | | | |

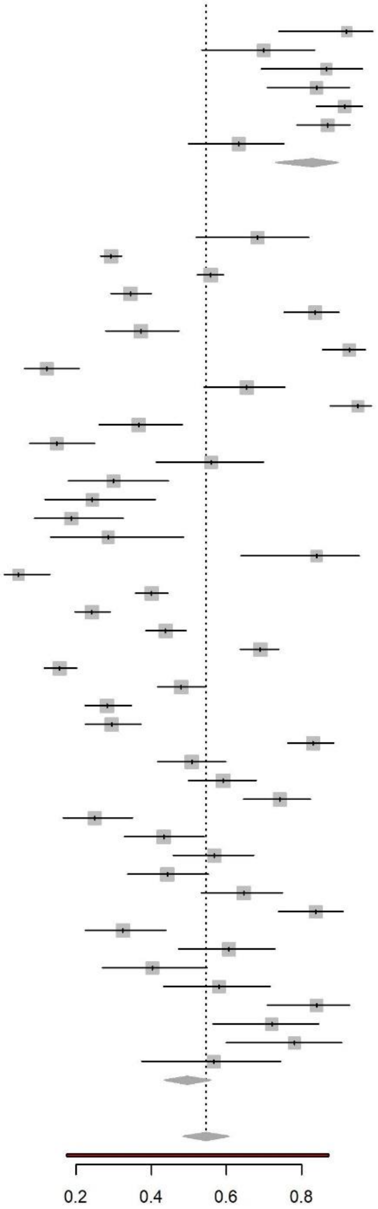

**Additional Figure 4 Bubble plots of univariate meta-regressions.** Each study is represented by a circle. Predictor variables: A study design, B year of publication, C quality score and D total children are plotted on the x-axis with the effect measure logit transformed proportion shown on the vertical (y-axis).

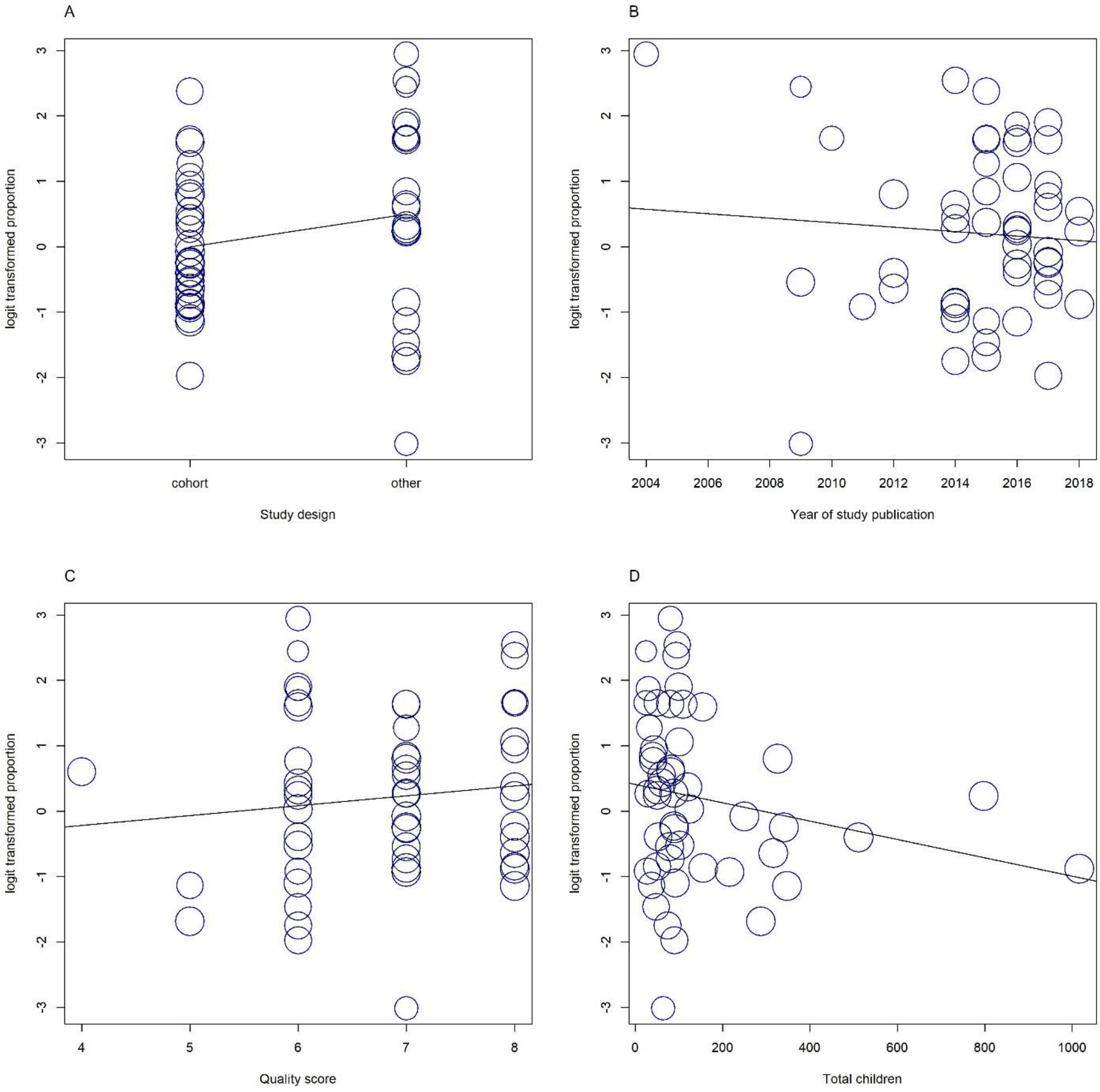

**Additional Figure 5 Funnel plot for prevalence of vitamin D deficiency in acute and critically ill children with sepsis.** Horizontal axis shows logit transformed proportion and the standard error of the logit transformed proportion is plotted on the vertical axis. Each dot represents an individual study and the vertical line represents the pooled proportion from a random-effects meta-analysis. The diagonal lines represent pseudo 95% confidence limits (p=0.81, Egger's test).

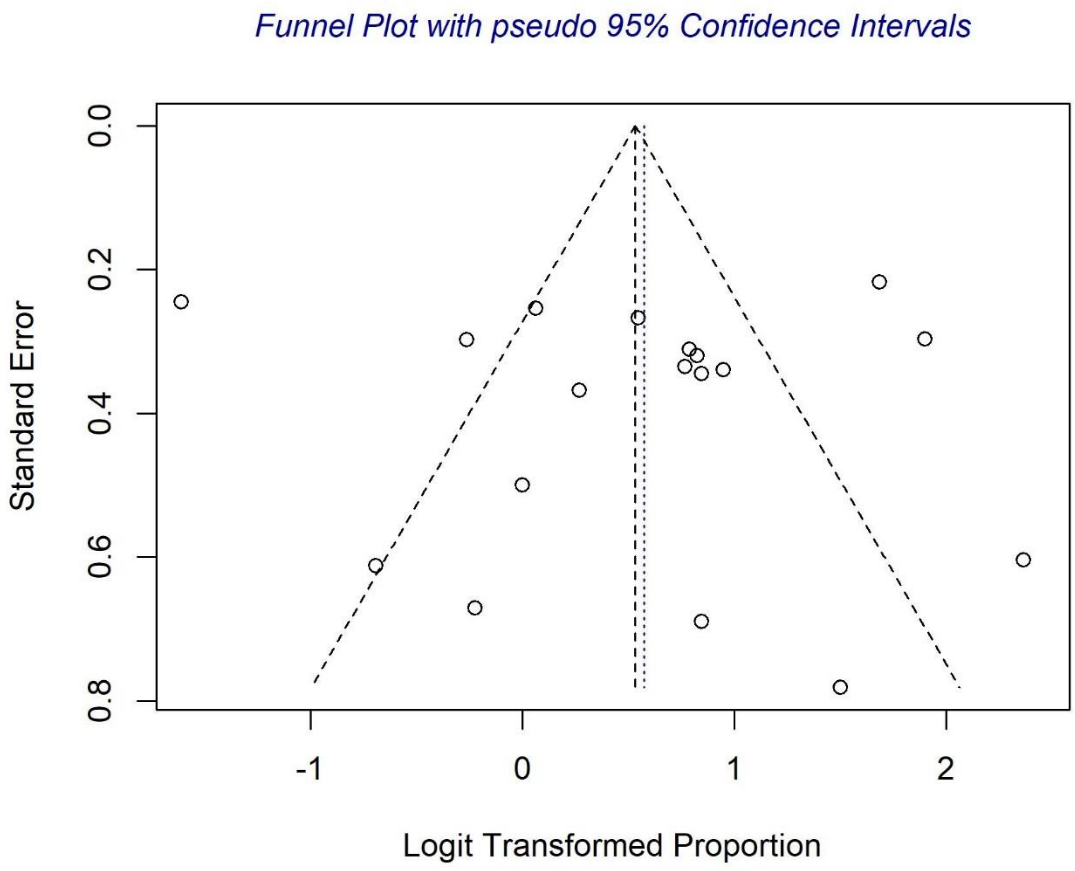




For peer review only - http://bmjopen.bmj.com/site/about/guidelines.xhtml

**Additional Figure 6 Pooled prevalence estimate for vitamin D deficiency in acute and critically ill children with sepsis (subgroup analysis by study design).** Forest plot shows results from the random effects model. Diamonds represent the pooled proportion of vitamin D deficiency for the studies in each subgroup (case-control and cohort). The diamond at the bottom shows the overall pooled estimate of all the 18 studies. Each square shows the prevalence estimate of each study and the horizontal line across each square represents the 95% confidence interval (CI) of the prevalence estimate.

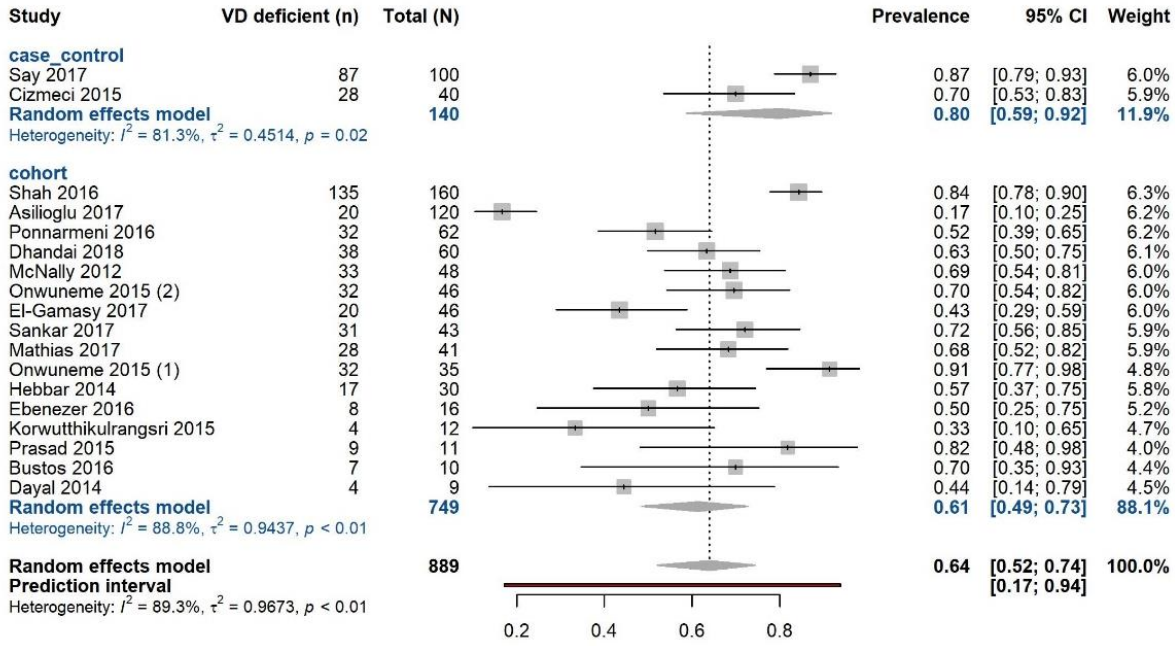

**Additional Figure 7 Funnel plot of risk of mortality in vitamin D deficient versus vitamin D non-deficient acute and critically ill children.** Horizontal axis shows logit transformed odds ratio and the standard error of the log odds ratio is plotted on the vertical axis. Each dot represents an individual study and the vertical line represents the overall pooled odds ratio from random-effects meta-analysis. The diagonal lines represent pseudo 95% confidence limits (p=0.084, Egger's test).

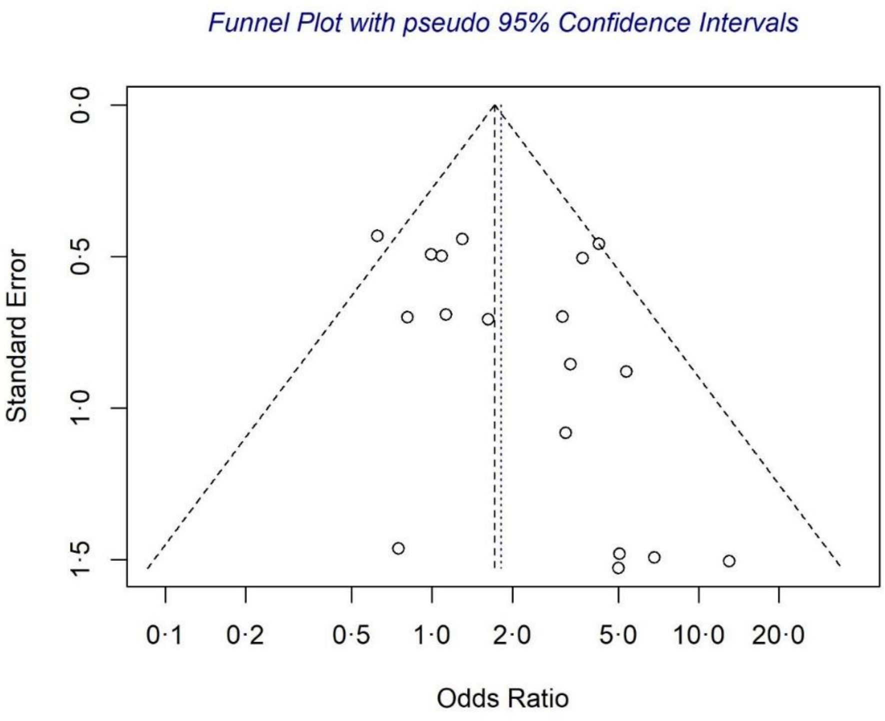


w only

**Additional Figure 8 Pooled odds ratio (OR) and 95% CI for risk of mortality in vitamin D deficient versus vitamin D non-deficient acute and critically ill children (fixed effects model).** Diamond represents the overall odds ratio (with corresponding 95% Confidence Interval). Each square shows the odds ratio of each study and the horizontal line across each square represents the 95% confidence interval (CI) of the estimate.

| Study | Deficient Dead (n) | Deficient Total (N) | Not deficient Dead (n) | Not deficient Total (N) | OR | 95% CI | Weight |
|---|---|---|---|---|---|---|---|
| Ebenezer 2016 | 5 | 21 | 5 | 31 | 1.62 | [0.41; 6.51] | 4.9% |
| Ponnarmeni 2016 | 10 | 63 | 9 | 61 | 1.09 | [0.41; 2.90] | 9.8% |
| Sankar 2016 | 23 | 75 | 8 | 26 | 1.00 | [0.38; 2.62] | 10.0% |
| Rippel 2012 | 3 | 109 | 7 | 207 | 0.81 | [0.20; 3.19] | 5.0% |
| Dayal 2014 | 3 | 23 | 3 | 69 | 3.30 | [0.62; 17.65] | 3.3% |
| Shah 2016 | 54 | 128 | 14 | 26 | 0.63 | [0.27; 1.46] | 13.0% |
| Prasad 2015 | 14 | 67 | 1 | 13 | 3.17 | [0.38; 26.50] | 2.1% |
| Bustos 2016 | 4 | 39 | 0 | 51 | 13.06 | [0.68; 250.17] | 1.1% |
| McNally 2012 | 5 | 225 | 0 | 101 | 5.06 | [0.28; 92.45] | 1.1% |
| Elmoneim 2016 | 1 | 17 | 1 | 13 | 0.75 | [0.04; 13.24] | 1.1% |
| Korwutthikulrangsri 2015 | 6 | 25 | 0 | 7 | 5.00 | [0.25; 100.15] | 1.0% |
| Ayulo 2014 | 4 | 61 | 2 | 155 | 5.37 | [0.96; 30.11] | 3.1% |
| Garcia-Soler 2017 | 7 | 149 | 3 | 191 | 3.09 | [0.79; 12.16] | 5.0% |
| Onwuneme 2015 (2) | 5 | 60 | 0 | 34 | 6.84 | [0.37; 127.56] | 1.1% |
| Asilioglu 2017 | 16 | 120 | 9 | 85 | 1.30 | [0.55; 3.10] | 12.4% |
| El-Gamasy 2017 | 14 | 26 | 13 | 54 | 3.68 | [1.36; 9.92] | 9.5% |
| Badawi 2017 | 27 | 39 | 17 | 49 | 4.24 | [1.72; 10.41] | 11.5% |
| Sankar 2017 | 19 | 31 | 7 | 12 | 1.13 | [0.29; 4.39] | 5.1% |
| **Fixed effect model** | | **1278** | | **1185** | **1.72** | **[1.27; 2.33]** | **100.0%** |

Heterogeneity: $I^2 = 25.7\%$, $\tau^2 = 0.1564$, $p = 0.15$

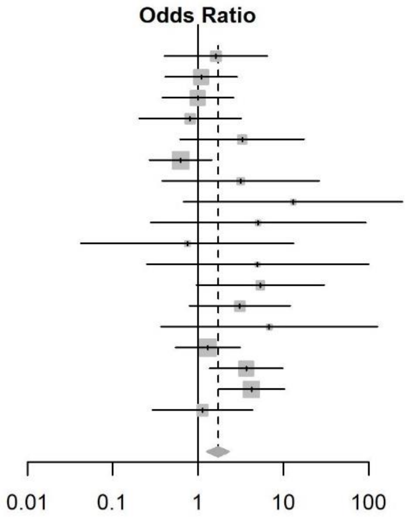


review only

# MOOSE (Meta-analyses Of Observational Studies in Epidemiology) Checklist

A reporting checklist for Authors, Editors, and Reviewers of Meta-analyses of Observational Studies. You must report the page number in your manuscript where you consider each of the items listed in this checklist. If you have not included this information, either revise your manuscript accordingly before submitting or note N/A.

| Reporting Criteria | Reported (Yes/No) | Reported on Page No. |
|---|---|---|
| **Reporting of Background** | | |
| Problem definition | Yes | 4-5 |
| Hypothesis statement | Yes | 5 |
| Description of Study Outcome(s) | Yes | 7 |
| Type of exposure or intervention used | Yes | 1-2 |
| Type of study design used | Yes | 2 |
| Study population | Yes | 2 |
| **Reporting of Search Strategy** | | |
| Qualifications of searchers (eg, librarians and investigators) | Yes | 6 |
| Search strategy, including time period included in the synthesis and keywords | Yes | 6 |
| Effort to include all available studies, including contact with authors | Yes | 6-7 |
| Databases and registries searched | Yes | 6 |
| Search software used, name and version, including special features used (eg, explosion) | Yes | 6, 9 |
| Use of hand searching (eg, reference lists of obtained articles) | Yes | 7 |
| List of citations located and those excluded, including justification | Yes | 10 & Suppl. |
| Method for addressing articles published in languages other than English | Yes | 6 |
| Method of handling abstracts and unpublished studies | Yes | 7 |
| Description of any contact with authors | Yes | 7 |
| **Reporting of Methods** | | |
| Description of relevance or appropriateness of studies assembled for assessing the hypothesis to be tested | Yes | 6-7 |
| Rationale for the selection and coding of data (eg, sound clinical principles or convenience) | Yes | 6-7 |
| Documentation of how data were classified and coded (eg, multiple raters, blinding, and interrater reliability) | Yes | 7 |
| Assessment of confounding (eg, comparability of cases and controls in studies where appropriate | Yes | 7 |

For peer review only

| Reporting Criteria | Reported (Yes/No) | Reported on Page No. |
|---|---|---|
| Assessment of study quality, including blinding of quality assessors; stratification or regression on possible predictors of study results | Yes | 7 & 20 |
| Assessment of heterogeneity | Yes | 8-9 |
| Description of statistical methods (eg, complete description of fixed or random effects models, justification of whether the chosen models account for predictors of study results, dose-response models, or cumulative meta-analysis) in sufficient detail to be replicated | Yes | 8-9 |
| Provision of appropriate tables and graphics | Yes | 12 & Suppl. |
| **Reporting of Results** | | |
| Table giving descriptive information for each study included | Yes | 10 & Suppl. |
| Results of sensitivity testing (eg, subgroup analysis) | Yes | 13, 15-16 |
| Indication of statistical uncertainty of findings | Yes | 20-21 |
| **Reporting of Discussion** | | |
| Quantitative assessment of bias (eg, publication bias) | Yes | 8-9, 15-18 |
| Justification for exclusion (eg, exclusion of non–English-language citations) | Yes | 7 |
| Assessment of quality of included studies | Yes | 7 |
| **Reporting of Conclusions** | | |
| Consideration of alternative explanations for observed results | Yes | 21-22 |
| Generalization of the conclusions (ie, appropriate for the data presented and within the domain of the literature review) | Yes | 21-22 |
| Guidelines for future research | Yes | 21-22 |
| Disclosure of funding source | Yes | 9 & 22 |

**Once you have completed this checklist, please save a copy and upload it as part of your submission. DO NOT include this checklist as part of the main manuscript document. It must be uploaded as a separate file.**

For peer review only
